# Supplementary material for: A Bacterial Platform for Studying Ubiquitination Cascades Anchored by SCF-Type E3 Ubiquitin Ligases
Source: Biomolecules. 2024 Sep 25;14(10):1209. doi: 10.3390/biom14101209 (PMC11505812; doi:10.3390/biom14101209)
Supplement: Supplementary file 1 [file biomolecules-14-01209-s001.zip › Attached files.pdf]

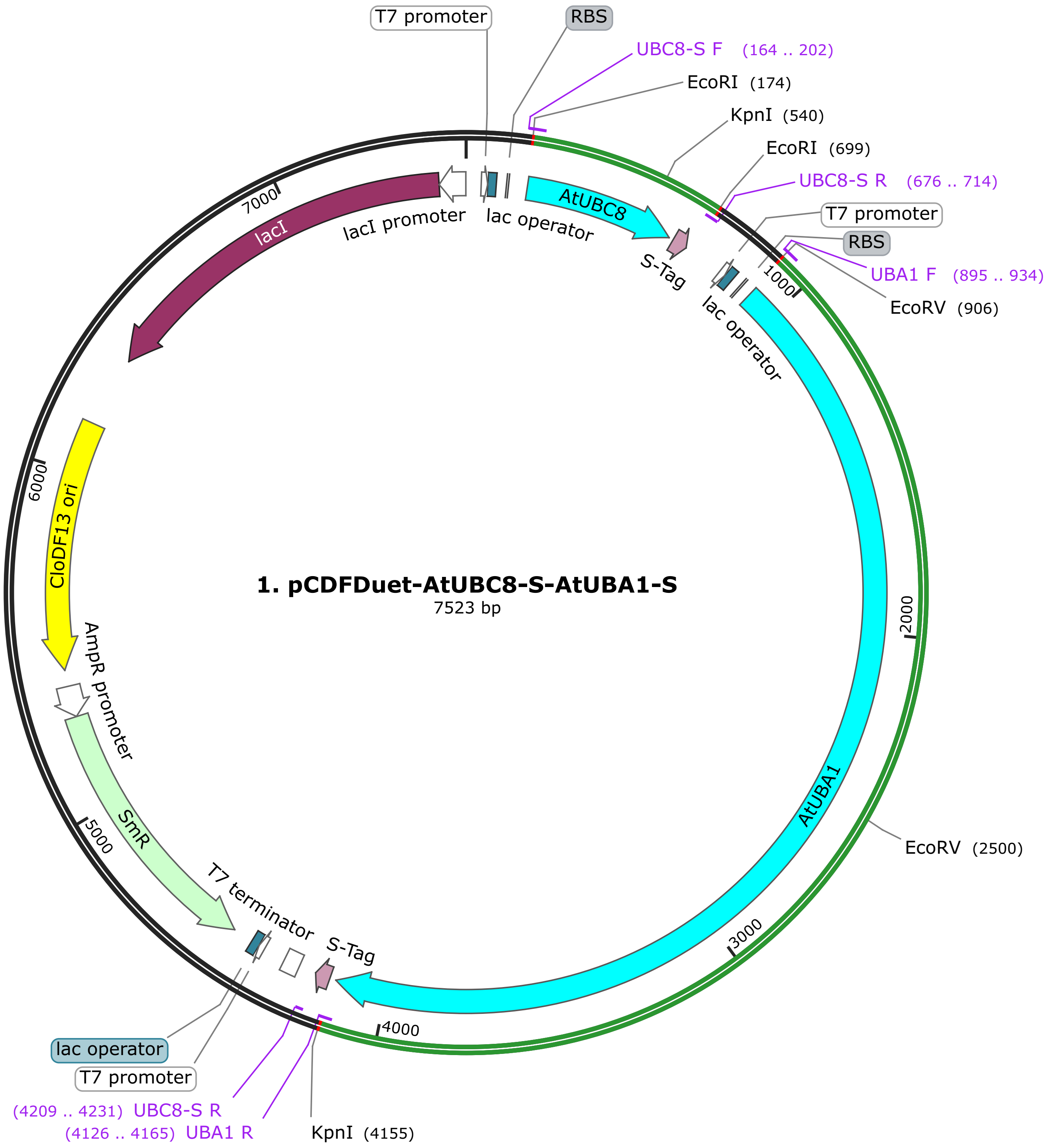
Created with SnapGene®
1. pCDFDuet-AtUBC8-S-AtUBA1-S
7523 bp
T7 promoter
RBS
UBC8-S F (164 .. 202)
EcoRI (174)
KpnI (540)
EcoRI (699)
UBC8-S R (676 .. 714)
T7 promoter
RBS
UBA1 F (895 .. 934)
EcoRV (906)
EcoRV (2500)
KpnI (4155)
(4126 .. 4165) UBA1 R
(4209 .. 4231) UBC8-S R
T7 promoter
lac operator
lacI promoter
lac operator
AtUBC8
S-Tag
lac operator
AtUBA1
S-Tag
T7 terminator
SmR
AmpR promoter
CloDF13 ori
lacI
1000
2000
3000
4000
5000
6000
7000

Sequence: 1. pCDFDuet-AtUBC8-S-AtUBA1-S.dna (Circular / 7523 bp)
Enzymes: < Unsaved Enzyme Set > (3 of 678 total)
Features: 20 total
Primers: 7 total

Unique Cutters **Bold**

5′ gggatctcgacgctctcccttatgcgactcctgcattaggaaattaatacgactcactataggggaattgtgagcggataacaattcccc 90

3′ ccctagagctgcgagagggaatacgctgaggacgtaatcctttaattatgctgagtgatatccccttaacactcgcctattgttaagggg

T7 promoter | lac operator

EcoRI

UBC8-S F

ccaggGatccgaattcA

tgtagaaataattttgtttaactttaataaggagatataccatgggcagcagccatcaccatcatcaccacagccaggGatccgaattcA 180

acatctttattaaaacaaattgaaattattcctctatatggtacccgtcgtcggtagtggtagtagtggtgtcggtccCtaggcttaagT

RBS | AtUBC8

UBC8-S F

TGGCTTCGAAACGGATCTTGAA

TGGCTTCGAAACGGATCTTGAAGGAGCTGAAGGATCTCCAGAAAGACCCTCCAACCTCCTGCAGTGCAGGTCCAGTTGCTGAAGACATGT 270

ACCGAAGCTTTGCCTAGAACTTCCTCGACTTCCTAGAGGTCTTTCTGGGAGGTTGGAGGACGTCACGTCCAGGTCAACGACTTCTGTACA

1 Met Ala Ser Lys 5 Arg Ile Leu Lys Glu 10 Leu Lys Asp Leu Gln 15 Lys Asp Pro Pro Thr 20 Ser Cys Ser Ala Gly 25 Pro Val Ala Glu Asp 30 Met

AtUBC8

TTCATTGGCAAGCTACAATTATGGGTCCTGCAGAGAGTCCGTATTCAGGCGGTGTGTTTCTCGTTACCATTCACTTCCCTCCGGACTATC 360

AAGTAACCGTTCGATGTTAATACCCAGGACGTCTCTCAGGCATAAGTCCGCCACACAAAGAGCAATGGTAAGTGAAGGGAGGCCTGATAG

Phe His Trp Gln 35 Ala Thr Ile Met Gly 40 Pro Ala Glu Ser Pro 45 Tyr Ser Gly Gly Val 50 Phe Leu Val Thr Ile 55 His Phe Pro Pro Asp 60 Tyr

AtUBC8

CATTCAAACCACCAAAGGTTGCATTTAGGACGAAGGTGTTTCACCCTAATATCAACAGCAACGGAAGCATTTGCCTTGACATTTTGAAAG 450

GTAAGTTTGGTGGTTTCCAACGTAAATCCTGCTTCCACAAAGTGGGATTATAGTTGTCGTTGCCTTCGTAAACGGAACTGTAAAACTTTC

Pro Phe Lys Pro 65 Pro Lys Val Ala Phe 70 Arg Thr Lys Val Phe 75 His Pro Asn Ile Asn 80 Ser Asn Gly Ser Ile 85 Cys Leu Asp Ile Leu 90 Lys

AtUBC8

KpnI

AACAATGGAGCCCTGCCCTCACCATTTCCAAGGTGTTGCTCTCGATATGTTCGCTGTTAACAGATCCAAATCCAGATGACCCTTTGGTAC 540

TTGTTACCTCGGGACGGGAGTGGTAAAGGTTCCACAACGAGAGCTATACAAGCGACAATTGTCTAGGTTTAGGTCTACTGGGAAACCATG

Glu Gln Trp Ser 95 Pro Ala Leu Thr Ile 100 Ser Lys Val Leu Leu 105 Ser Ile Cys Ser Leu 110 Leu Thr Asp Pro Asn 115 Pro Asp Asp Pro Leu 120 Val

AtUBC8

CAGAGATTGCACACATGTACAAAACCGACAGAGCCAAATACGAGGCTACTGCAAGAAACTGGACTCAGAAGTATGCCATGGGcctcgagt 630

GTCTCTAACGTGTGTACATGTTTTGGCTGTCTCGGTTTATGCTCCGATGACGTTCTTTGACCTGAGTCTTCATACGGTACCCggagctca

Pro Glu Ile Ala 125 His Met Tyr Lys Thr 130 Asp Arg Ala Lys Tyr 135 Glu Ala Thr Ala Arg 140 Asn Trp Thr Gln Lys 145 Tyr Ala Met Gly Leu Glu

AtUBC8

EcoRI

ctggtaaagaaaccgctgctgcgaaatttgaacgccagcacatggactcgtctactagcgcagcttaagaattcgagctcggcgcgcctg 720

gaccatttctttggcgacgacgctttaaacttgcggtcgtgtacctgagcagatgatcgcgtcgaattcttaagctcgagccgcgcggac

Ser Gly 1 Lys Glu Thr Ala 5 Ala Ala Lys Phe Glu 10 Arg Gln His Met Asp 15 Ser Ser Thr Ser Ala Ala *

S-Tag (in frame with S-Tag)

tgagcagatgatcgcgtcgaattcttaagctcgagccgc

UBC8-S R

caggtcgacaagcttgcggccgcataatgcttaagtcgaacagaaagtaatcgtattgtacacggccgcataatcgaaattaatacgact 810

gtccagctgttcgaacgccggcgtattacgaattcagcttgtctttcattagcataacatgtgccggcgtattagctttaattatgctga

T7 promoter

UBA1 F
tctcaa

cactataggggaattgtgagcggataacaattccccatcttagtatattagttaagtataagaaggagatatacatatggcagatctcaa 900
gtgatatccccttaacactcgcctattgttaaggggtagaatcatataatcaattcatattcttcctctatatgtataccgtctagagtt

T7 promoter | lac operator | RBS

EcoRV

UBA1 F
ttggatatcgATGCTTCACAAGCGAGCTAGTGAA

ttggatatcgATGCTTCACAAGCGAGCTAGTGAAGCTAACGATAAGAACGATAATACCATCATCGGTTCCGATTTAGCATCATCTAAGAA 990
aacctatagcTACGAAGTGTTCGCTCGATCACTTCGATTGCTATTCTTGCTATTATGGTAGTAGCCAAGGCTAAATCGTAGTAGATTCTT

1 5 10 15 20 25
Met Leu His Lys Arg Ala Ser Glu Ala Asn Asp Lys Asn Asp Asn Thr Ile Ile Gly Ser Asp Leu Ala Ser Ser Lys Lys
AtUBA1

ACGCCGTATCGATTTCACTGAATCTTCGTCTGATAAGTCTTCTTCGATTTTAGCTTCTGGTAGTAGCAGGGGTTTTCACGGCGATAGCGT 1080
TGCGGCATAGCTAAAGTGACTTAGAAGCAGACTATTCAGAAGAAGCTAAAATCGAAGACCATCATCGTCCCCAAAAGTGCCGCTATCGCA

30 35 40 45 50 55
Arg Arg Ile Asp Phe Thr Glu Ser Ser Ser Asp Lys Ser Ser Ser Ile Leu Ala Ser Gly Ser Ser Arg Gly Phe His Gly Asp Ser Val
AtUBA1

CGTTCAGCAAATCGACATGGCTTTTGGTAATTCGAACCGTCAGGAGATTGATGAAGATCTGCACAGTCGCCAGCTCGCCGTCTATGGTCG 1170
GCAAGTCGTTTAGCTGTACCGAAAACCATTAAGCTTGGCAGTCCTCTAACTACTTCTAGACGTGTCAGCGGTCGAGCGGCAGATACCAGC

60 65 70 75 80 85
Val Gln Gln Ile Asp Met Ala Phe Gly Asn Ser Asn Arg Gln Glu Ile Asp Glu Asp Leu His Ser Arg Gln Leu Ala Val Tyr Gly Arg
AtUBA1

TGAGACTATGAGGCGTCTCTTTGCTTCCAATGTTCTCATCTCGGGGATGCACGGTCTTGGTGCCGAGATTGCCAAGAATCTTATACTTGC 1260
ACTCTGATACTCCGCAGAGAAACGAAGGTTACAAGAGTAGAGCCCCTACGTGCCAGAACCACGGCTCTAACGGTTCTTAGAATATGAACG

90 95 100 105 110 115
Glu Thr Met Arg Arg Leu Phe Ala Ser Asn Val Leu Ile Ser Gly Met His Gly Leu Gly Ala Glu Ile Ala Lys Asn Leu Ile Leu Ala
AtUBA1

TGGTGTGAAGTCTGTGACCTTGCATGATGAAAGAGTTGTAGAGCTATGGGACTTATCAAGCAACTTTGTTTTCTCGGAGGATGATGTTGG 1350
ACCACACTTCAGACACTGGAACGTACTACTTTCTCAACATCTCGATACCCTGAATAGTTCGTTGAAACAAAAGAGCCTCCTACTACAACC

120 125 130 135 140 145
Gly Val Lys Ser Val Thr Leu His Asp Glu Arg Val Val Glu Leu Trp Asp Leu Ser Ser Asn Phe Val Phe Ser Glu Asp Asp Val Gly
AtUBA1

CAAGAATAGAGCAGATGCTTCTGTTCAGAAGTTACAGGATCTTAACAATGCTGTGGTTGTCTCTAGCTTGACCAAAAGCTTAAACAAAGA 1440
GTTCTTATCTCGTCTACGAAGACAAGTCTTCAATGTCCTAGAATTGTTACGACACCAACAGAGATCGAACTGGTTTTCGAATTTGTTTCT

150 155 160 165 170 175
Lys Asn Arg Ala Asp Ala Ser Val Gln Lys Leu Gln Asp Leu Asn Asn Ala Val Val Val Ser Ser Leu Thr Lys Ser Leu Asn Lys Glu
AtUBA1

GGATCTTTCTGGCTTCCAGGTTGTTGTATTCTCTGACATAAGCATGGAAAGAGCAATCGAATTTGATGACTATTGTCACAGCCATCAGCC 1530
CCTAGAAAGACCGAAGGTCCAACAACATAAGAGACTGTATTCGTACCTTTCTCGTTAGCTTAAACTACTGATAACAGTGTCGGTAGTCGG

180 185 190 195 200 205
Asp Leu Ser Gly Phe Gln Val Val Val Phe Ser Asp Ile Ser Met Glu Arg Ala Ile Glu Phe Asp Asp Tyr Cys His Ser His Gln Pro
AtUBA1

TCCTATCGCTTTTGTCAAGGCTGATGTCAGGGGTCTTTTCGGCTCTGTCTTTTGTGATTTTGGGCCTGAATTTGCGGTTCTTGATGTTGA 1620
AGGATAGCGAAAACAGTTCCGACTACAGTCCCCAGAAAAGCCGAGACAGAAAACACTAAAACCCGGACTTAAACGCCAAGAACTACAACT

210 215 220 225 230 235
Pro Ile Ala Phe Val Lys Ala Asp Val Arg Gly Leu Phe Gly Ser Val Phe Cys Asp Phe Gly Pro Glu Phe Ala Val Leu Asp Val Asp
AtUBA1

TGGAGAGGAACCCCATACAGGCATCATTGCGTCTATCTCTAATGAGAACCAGGCATTTATTTCCTGTGTCGACGATGAGAGGCTTGAGTT 1710
ACCTCTCCTTGGGGTATGTCCGTAGTAACGCAGATAGAGATTACTCTTGGTCCGTAAATAAAGGACACAGCTGCTACTCTCCGAACTCAA

240 245 250 255 260 265
Gly Glu Glu Pro His Thr Gly Ile Ile Ala Ser Ile Ser Asn Glu Asn Gln Ala Phe Ile Ser Cys Val Asp Asp Glu Arg Leu Glu Phe
AtUBA1

```
TGAAGATGGTGACCTAGTTGTTTTCTCAGAAGTTGAGGGTATGACAGAATTGAATGATGGGAAACCAAGGAAGATAAAAAGTACACGGCC   1800
ACTTCTACCACTGGATCAACAAAAGAGTCTTCAACTCCCATACTGTCTTAACTTACTACCCTTTGGTTCCTTCTATTTTTCATGTGCCGG
270 275 280 285 290 295
Glu Asp Gly Asp Leu Val Val Phe Ser Glu Val Glu Gly Met Thr Glu Leu Asn Asp Gly Lys Pro Arg Lys Ile Lys Ser Thr Arg Pro
AtUBA1

ATATTCATTCACCCTTGATGAGGACACTACTAACTATGGAACATATGTGAAGGGTGGTATTGTCACTCAAGTGAAACAGCCAAAGTTACT   1890
TATAAGTAAGTGGGAACTACTCCTGTGATGATTGATACCTTGTATACACTTCCCACCATAACAGTGAGTTCACTTTGTCGGTTTCAATGA
300 305 310 315 320 325
Tyr Ser Phe Thr Leu Asp Glu Asp Thr Thr Asn Tyr Gly Thr Tyr Val Lys Gly Gly Ile Val Thr Gln Val Lys Gln Pro Lys Leu Leu
AtUBA1

GAATTTCAAGCCATTGAGGGAAGCGCTTAAGGATCCAGGGGATTTTCTATTTAGTGATTTCTCTAAGTTTGATCGGCCTCCACTCCTTCA   1980
CTTAAAGTTCGGTAACTCCCTTCGCGAATTCCTAGGTCCCCTAAAAGATAAATCACTAAAGAGATTCAAACTAGCCGGAGGTGAGGAAGT
330 335 340 345 350 355
Asn Phe Lys Pro Leu Arg Glu Ala Leu Lys Asp Pro Gly Asp Phe Leu Phe Ser Asp Phe Ser Lys Phe Asp Arg Pro Pro Leu Leu His
AtUBA1

CTTAGCATTCCAGGCACTTGATCATTTTAAAGCTGAAGCTGGCAGATTTCCTGTTGCTGGCTCAGAAGAGGACGCTCAGAAGCTTATATC   2070
GAATCGTAAGGTCCGTGAACTAGTAAAATTTCGACTTCGACCGTCTAAAGGACAACGACCGAGTCTTCTCCTGCGAGTCTTCGAATATAG
360 365 370 375 380 385
Leu Ala Phe Gln Ala Leu Asp His Phe Lys Ala Glu Ala Gly Arg Phe Pro Val Ala Gly Ser Glu Glu Asp Ala Gln Lys Leu Ile Ser
AtUBA1

GATTGCCACAGCCATCAATACCGGGCAGGGTGATTTAAAGGTGGAGAATGTTGATCAGAAGCTTCTAAGACACTTTTCCTTTGGAGCCAA   2160
CTAACGGTGTCGGTAGTTATGGCCCGTCCCACTAAATTTCCACCTCTTACAACTAGTCTTCGAAGATTCTGTGAAAAGGAAACCTCGGTT
390 395 400 405 410 415
Ile Ala Thr Ala Ile Asn Thr Gly Gln Gly Asp Leu Lys Val Glu Asn Val Asp Gln Lys Leu Leu Arg His Phe Ser Phe Gly Ala Lys
AtUBA1

GGCTGTCCTGAATCCTATGGCTGCAATGTTTGGCGGTATTGTTGGACAGGAGGTTGTTAAAGCTTGCTCTGGAAAATTTCATCCTCTCTT   2250
CCGACAGGACTTAGGATACCGACGTTACAAACCGCCATAACAACCTGTCCTCCAACAATTTCGAACGAGACCTTTTAAAGTAGGAGAGAA
420 425 430 435 440 445
Ala Val Leu Asn Pro Met Ala Ala Met Phe Gly Gly Ile Val Gly Gln Glu Val Val Lys Ala Cys Ser Gly Lys Phe His Pro Leu Phe
AtUBA1

CCAGTTTTTCTACTTTGATTCAGTGGAGTCACTCCCCTCTGAACCTGTGGACTCGAGTGACTTTGCACCAAGAAACAGCCGGTATGATGC   2340
GGTCAAAAAGATGAAACTAAGTCACCTCAGTGAGGGGAGACTTGGACACCTGAGCTCACTGAAACGTGGTTCTTTGTCGGCCATACTACG
450 455 460 465 470 475
Gln Phe Phe Tyr Phe Asp Ser Val Glu Ser Leu Pro Ser Glu Pro Val Asp Ser Ser Asp Phe Ala Pro Arg Asn Ser Arg Tyr Asp Ala
AtUBA1

CCAAATATCTGTATTTGGGGCCAAGTTCCAGAAGAAACTTGAAGATGCTAAAGTTTTCACAGTAGGGTCTGGTGCTCTTGGCTGTGAGTT   2430
GGTTTATAGACATAAACCCCGGTTCAAGGTCTTCTTTGAACTTCTACGATTTCAAAAGTGTCATCCCAGACCACGAGAACCGACACTCAA
480 485 490 495 500 505
Gln Ile Ser Val Phe Gly Ala Lys Phe Gln Lys Lys Leu Glu Asp Ala Lys Val Phe Thr Val Gly Ser Gly Ala Leu Gly Cys Glu Phe
AtUBA1

EcoRV
CTTGAAAAATTTGGCTCTGATGGGGGTTTCTTGTGGAAGCCAAGGGAAGCTGACAGTGACAGATGATGATATCATTGAGAAGAGTAACCT   2520
GAACTTTTTAAACCGAGACTACCCCCAAAGAACACCTTCGGTTCCCTTCGACTGTCACTGTCTACTACTATAGTAACTCTTCTCATTGGA
510 515 520 525 530 535
Leu Lys Asn Leu Ala Leu Met Gly Val Ser Cys Gly Ser Gln Gly Lys Leu Thr Val Thr Asp Asp Asp Ile Ile Glu Lys Ser Asn Leu
AtUBA1

CAGTCGTCAATTTCTGTTCCGTGACTGGAACATTGGACAGGCTAAATCCACAGTTGCTGCTTCGGCTGCTGCTGTTATAAATCCTCGCTT   2610
GTCAGCAGTTAAAGACAAGGCACTGACCTTGTAACCTGTCCGATTTAGGTGTCAACGACGAAGCCGACGACGACAATATTTAGGAGCGAA
540 545 550 555 560 565
Ser Arg Gln Phe Leu Phe Arg Asp Trp Asn Ile Gly Gln Ala Lys Ser Thr Val Ala Ala Ser Ala Ala Ala Val Ile Asn Pro Arg Phe
AtUBA1
```

CAACATTGAGGCCCTGCAAAACCGTGTTGGCGCGGAGACTGAGAATGTATTTGACGATGCCTTCTGGGAGAACTTAACTGTTGTCGTCAA 2700
GTTGTAACTCCGGGACGTTTTGGCACAACCGCGCCTCTGACTCTTACATAAACTGCTACGGAAGACCCTCTTGAATTGACAACAGCAGTT

570 575 580 585 590 595
Asn Ile Glu Ala Leu Gln Asn Arg Val Gly Ala Glu Thr Glu Asn Val Phe Asp Asp Ala Phe Trp Glu Asn Leu Thr Val Val Val Asn
AtUBA1

TGCACTGGATAATGTTAATGCGAGGCTCTATGTTGATTCGAGGTGCTTGTATTTCCAGAAGCCTCTCCTTGAGTCTGGAACTCTTGGTAC 2790
ACGTGACCTATTACAATTACGCTCCGAGATACAACTAAGCTCCACGAACATAAAGGTCTTCGGAGAGGAACTCAGACCTTGAGAACCATG

600 605 610 615 620 625
Ala Leu Asp Asn Val Asn Ala Arg Leu Tyr Val Asp Ser Arg Cys Leu Tyr Phe Gln Lys Pro Leu Leu Glu Ser Gly Thr Leu Gly Thr
AtUBA1

AAAGTGCAACACACAGTCGGTTATCCCACATCTAACTGAAAATTACGGTGCCTCAAGGGACCCACCAGAGAAACAGGCCCCCATGTGTAC 2880
TTTCACGTTGTGTGTCAGCCAATAGGGTGTAGATTGACTTTTAATGCCACGGAGTTCCCTGGGTGGTCTCTTTGTCCGGGGGTACACATG

630 635 640 645 650 655
Lys Cys Asn Thr Gln Ser Val Ile Pro His Leu Thr Glu Asn Tyr Gly Ala Ser Arg Asp Pro Pro Glu Lys Gln Ala Pro Met Cys Thr
AtUBA1

AGTGCATTCCTTCCCGCATAACATTGATCACTGTTTAACTTGGGCTCGCTCTGAGTTTGAGGGTCTGCTGGAGAAGACTCCCGCTGAAGT 2970
TCACGTAAGGAAGGGCGTATTGTAACTAGTGACAAATTGAACCCGAGCGAGACTCAAACTCCCAGACGACCTCTTCTGAGGGCGACTTCA

660 665 670 675 680 685
Val His Ser Phe Pro His Asn Ile Asp His Cys Leu Thr Trp Ala Arg Ser Glu Phe Glu Gly Leu Leu Glu Lys Thr Pro Ala Glu Val
AtUBA1

GAATGCATATCTTTCTAGCCCAGTTGAGTACACTAACTCAATGATGAGTGCTGGTGATGCTCAGGCGAGGGACACATTGGAGAGGATTGT 3060
CTTACGTATAGAAAGATCGGGTCAACTCATGTGATTGAGTTACTACTCACGACCACTACGAGTCCGCTCCCTGTGTAACCTCTCCTAACA

690 695 700 705 710 715
Asn Ala Tyr Leu Ser Ser Pro Val Glu Tyr Thr Asn Ser Met Met Ser Ala Gly Asp Ala Gln Ala Arg Asp Thr Leu Glu Arg Ile Val
AtUBA1

TGAGTGCCTTGAAAAGGAGAAGTGTGAGACCTTCCAAGACTGCTTAACCTGGGCACGACTGAGGTTTGAGGATTACTTTGTGAACCGTGT 3150
ACTCACGGAACTTTTCCTCTTCACACTCTGGAAGGTTCTGACGAATTGGACCCGTGCTGACTCCAAACTCCTAATGAAACACTTGGCACA

720 725 730 735 740 745
Glu Cys Leu Glu Lys Glu Lys Cys Glu Thr Phe Gln Asp Cys Leu Thr Trp Ala Arg Leu Arg Phe Glu Asp Tyr Phe Val Asn Arg Val
AtUBA1

CAAGCAATTGATATACACATTTCCTGAAGATGCTGCGACTAGTACCGGAGCTCCATTCTGGTCTGCTCCAAAAAGATTCCCTCGTCCACT 3240
GTTCGTTAACTATATGTGTAAAGGACTTCTACGACGCTGATCATGGCCTCGAGGTAAGACCAGACGAGGTTTTTCTAAGGGAGCAGGTGA

750 755 760 765 770 775
Lys Gln Leu Ile Tyr Thr Phe Pro Glu Asp Ala Ala Thr Ser Thr Gly Ala Pro Phe Trp Ser Ala Pro Lys Arg Phe Pro Arg Pro Leu
AtUBA1

CCAGTACTCGTCGTCCGACCCAAGTCTCCTTAACTTTATCACAGCGACTGCTATTTTACGAGCAGAGACATTTGGGATCCCGATACCTGA 3330
GGTCATGAGCAGCAGGCTGGGTTCAGAGGAATTGAAATAGTGTCGCTGACGATAAAATGCTCGTCTCTGTAAACCCTAGGGCTATGGACT

780 785 790 795 800 805
Gln Tyr Ser Ser Ser Asp Pro Ser Leu Leu Asn Phe Ile Thr Ala Thr Ala Ile Leu Arg Ala Glu Thr Phe Gly Ile Pro Ile Pro Glu
AtUBA1

GTGGACTAAGAACCCAAAGGAAGCAGCAGAAGCTGTTGACAGAGTGATAGTCCCAGACTTTGAGCCAAGGCAAGATGCAAAGATTGTGAC 3420
CACCTGATTCTTGGGTTTCCTTCGTCGTCTTCGACAACTGTCTCACTATCAGGGTCTGAAACTCGGTTCCGTTCTACGTTTCTAACACTG

810 815 820 825 830 835
Trp Thr Lys Asn Pro Lys Glu Ala Ala Glu Ala Val Asp Arg Val Ile Val Pro Asp Phe Glu Pro Arg Gln Asp Ala Lys Ile Val Thr
AtUBA1

TGATGAGAAAGCTACCACTTTAACCACTGCTTCGGTGGACGACGCTGCAGTCATCGACGATCTCATTGCTAAGATCGACCAGTGTAGGCA 3510
ACTACTCTTTCGATGGTGAAATTGGTGACGAAGCCACCTGCTGCGACGTCAGTAGCTGCTAGAGTAACGATTCTAGCTGGTCACATCCGT

840 845 850 855 860 865
Asp Glu Lys Ala Thr Thr Leu Thr Thr Ala Ser Val Asp Asp Ala Ala Val Ile Asp Asp Leu Ile Ala Lys Ile Asp Gln Cys Arg His
AtUBA1

TAACTTGTCGCCAGACTTCAGGATGAAACCTATTCAGTTCGAAAAGGATGATGATACAAACTATCACATGGATGTGATAGCAGGACTAGC 3600
ATTGAACAGCGGTCTGAAGTCCTACTTTGGATAAGTCAAGCTTTTCCTACTACTATGTTTGATAGTGTACCTACACTATCGTCCTGATCG

870 875 880 885 890 895
Asn Leu Ser Pro Asp Phe Arg Met Lys Pro Ile Gln Phe Glu Lys Asp Asp Asp Thr Asn Tyr His Met Asp Val Ile Ala Gly Leu Ala
AtUBA1

CAACATGAGGGCGAGAAATTACAGCATCCCTGAAGTGGACAAGCTGAAAGCAAAGTTCATAGCAGGGAGAATCATCCCAGCCATTGCGAC 3690
GTTGTACTCCCGCTCTTTAATGTCGTAGGGACTTCACCTGTTCGACTTTCGTTTCAAGTATCGTCCCTCTTAGTAGGGTCGGTAACGCTG

900 905 910 915 920 925
Asn Met Arg Ala Arg Asn Tyr Ser Ile Pro Glu Val Asp Lys Leu Lys Ala Lys Phe Ile Ala Gly Arg Ile Ile Pro Ala Ile Ala Thr
AtUBA1

TTCAACAGCCATGGCTACTGGGCTGGTCTGCCTCGAGCTGTACAAGGTCCTTGATGGAGGACATAAAGTGGAAGCTTACAGAAACACATT 3780
AAGTTGTCGGTACCGATGACCCGACCAGACGGAGCTCGACATGTTCCAGGAACTACCTCCTGTATTTCACCTTCGAATGTCTTTGTGTAA

930 935 940 945 950 955
Ser Thr Ala Met Ala Thr Gly Leu Val Cys Leu Glu Leu Tyr Lys Val Leu Asp Gly Gly His Lys Val Glu Ala Tyr Arg Asn Thr Phe
AtUBA1

TGCCAACCTGGCACTTCCACTATTCTCCATGGCTGAACCACTTCCACCAAAGGTGGTGAAGCACCGTGACATGGCTTGGACAGTTTGGGA 3870
ACGGTTGGACCGTGAAGGTGATAAGAGGTACCGACTTGGTGAAGGTGGTTTCCACCACTTCGTGGCACTGTACCGAACCTGTCAAACCCT

960 965 970 975 980 985
Ala Asn Leu Ala Leu Pro Leu Phe Ser Met Ala Glu Pro Leu Pro Pro Lys Val Val Lys His Arg Asp Met Ala Trp Thr Val Trp Asp
AtUBA1

CAGATGGGTTCTCAAGGGAAACCCAACTCTTCGTGAGGTGTTGCAGTGGCTGGAGGACAAAGGGCTTAGCGCATACAGCATCTCTTGTGG 3960
GTCTACCCAAGAGTTCCCTTTGGGTTGAGAAGCACTCCACAACGTCACCGACCTCCTGTTTCCCGAATCGCGTATGTCGTAGAGAACACC

990 995 1000 1005 1010 1015
Arg Trp Val Leu Lys Gly Asn Pro Thr Leu Arg Glu Val Leu Gln Trp Leu Glu Asp Lys Gly Leu Ser Ala Tyr Ser Ile Ser Cys Gly
AtUBA1

AAGCTGTCTTCTGTTCAACAGTATGTTCACAAGGCACAAGGAGAGGATGGACAAGAAGGTTGTGGATCTCGCTCGGGATGTTGCGAAAGT 4050
TTCGACAGAAGACAAGTTGTCATACAAGTGTTCCGTGTTCCTCTCCTACCTGTTCTTCCAACACCTAGAGCGAGCCCTACAACGCTTTCA

1020 1025 1030 1035 1040 1045
Ser Cys Leu Leu Phe Asn Ser Met Phe Thr Arg His Lys Glu Arg Met Asp Lys Lys Val Val Asp Leu Ala Arg Asp Val Ala Lys Val
AtUBA1

CGAGTTACCGCCTTATCGCAACCATCTTGATGTAGTGGTGGCTTGTGAGGATGAAGATGACAATGACGTCGATATTCCTCTCGTCTCTAT 4140
GCTCAATGGCGGAATAGCGTTGGTAGAACTACATCACCACCGAACACTCCTACTTCTACTGTTACTGCAGCTATAAGGAGAGCAGAGATA

1050 1055 1060 1065 1070 1075
Glu Leu Pro Pro Tyr Arg Asn His Leu Asp Val Val Val Ala Cys Glu Asp Glu Asp Asp Asn Asp Val Asp Ile Pro Leu Val Ser Ile
AtUBA1

AGGAGAGCAGAGATA
UBA1 R

KpnI
CTACTTCAGGggtaccctcgagtctggtaaagaaaccgctgctgcgaaatttgaacgccagcacatggactcgtctactagcgcagctta 4230
GATGAAGTCCccatgggagctcagaccatttctttggcgacgacgctttaaacttgcggtcgtgtacctgagcagatgatcgcgtcgaat

1080 / 1 5 10 15
Tyr Phe Arg Gly Thr Leu Glu Ser Gly Lys Glu Thr Ala Ala Ala Lys Phe Glu Arg Gln His Met Asp Ser Ser Thr Ser Ala Ala ■
AtUBA1 (in frame with AtUBA1) S-Tag (in frame with S-Tag)

GATGAAGTCCcatgggagctcaga
UBA1 R

tgagcagatgatcgcgtcgaat
UBC8-S R

attaacctaggctgctgccaccgctgagcaataactagcataaccccttggggcctctaaacgggtcttgaggggttttttgctgaaacc 4320
taattggatccgacgacggtggcgactcgttattgatcgtattggggaaccccggagatttgcccagaactccccaaaaaacgactttgg

T7 terminator

t
cttaagctcgagccgc
UBC8-S R

tcaggcatttgagaagcacacggtcacactgcttccggtagtcaataaaccggtaaaccagcaatagacataagcggctatttaacgacc 4410
agtccgtaaactcttcgtgtgccagtgtgacgaaggccatcagttatttggccatttggtcgttatctgtattcgccgataaattgctgg
T7 promoter | lac operator

ctgccctgaaccgacgaccgggtcatcgtggccggatcttgcggcccctcggcttgaacgaattgttagacattatttgccgactacctt 4500
gacgggacttggctgctggcccagtagcaccggcctagaacgccggggagccgaacttgcttaacaatctgtaataaacggctgatggaa
260
* Lys Gly Val Val Lys
lac operator | SmR

ggtgatctcgcctttcacgtagtggacaaattcttccaactgatctgcgcgcgaggccaagcgatcttcttcttgtccaagataagcctg 4590
ccactagagcggaaagtgcatcacctgtttaagaaggttgactagacgcgcgctccggttcgctagaagaagaacaggttctattcggac
255 250 245 240 235 230
Thr Ile Glu Gly Lys Val Tyr His Val Phe Glu Glu Leu Gln Asp Ala Arg Ser Ala Leu Arg Asp Glu Glu Gln Gly Leu Tyr Ala Gln
SmR

tctagcttcaagtatgacgggctgatactgggccggcaggcgctccattgcccagtcggcagcgacatccttcggcgcgattttgccggt 4680
agatcgaagttcatactgcccgactatgacccggccgtccgcgaggtaacgggtcagccgtcgctgtaggaagccgcgctaaaacggcca
225 220 215 210 205 200
Arg Ala Glu Leu Ile Val Pro Gln Tyr Gln Ala Pro Leu Arg Glu Met Ala Trp Asp Ala Ala Val Asp Lys Pro Ala Ile Lys Gly Thr
SmR

tactgcgctgtaccaaatgcgggacaacgtaagcactacatttcgctcatcgccagcccagtcgggcggcgagttccatagcgttaaggt 4770
atgacgcgacatggtttacgccctgttgcattcgtgatgtaaagcgagtagcggtcgggtcagcccgccgctcaaggtatcgcaattcca
195 190 185 180 175 170
Val Ala Ser Tyr Trp Ile Arg Ser Leu Thr Leu Val Val Asn Arg Glu Asp Gly Ala Trp Asp Pro Pro Ser Asn Trp Leu Thr Leu Thr
SmR

ttcatttagcgcctcaaatagatcctgttcaggaaccggatcaaagagttcctccgccgctggacctaccaaggcaacgctatgttctct 4860
aagtaaatcgcggagtttatctaggacaagtccttggcctagtttctcaaggaggcggcgacctggatggttccgttgcgatacaagaga
165 160 155 150 145 140
Glu Asn Leu Ala Glu Phe Leu Asp Gln Glu Pro Val Pro Asp Phe Leu Glu Glu Ala Ala Pro Gly Val Leu Ala Val Ser His Glu Arg
SmR

tgcttttgtcagcaagatagccagatcaatgtcgatcgtggctggctcgaagatacctgcaagaatgtcattgcgctgccattctccaaa 4950
acgaaaacagtcgttctatcggtctagttacagctagcaccgaccgagcttctatggacgttcttacagtaacgcgacggtaagaggttt
135 130 125 120 115 110
Ala Lys Thr Leu Leu Ile Ala Leu Asp Ile Asp Ile Thr Ala Pro Glu Phe Ile Gly Ala Leu Ile Asp Asn Arg Gln Trp Glu Gly Phe
SmR

ttgcagttcgcgcttagctggataacgccacggaatgatgtcgtcgtgcacaacaatggtgacttctacagcgcggagaatctcgctctc 5040
aacgtcaagcgcgaatcgacctattgcggtgccttactacagcagcacgtgttgttaccactgaagatgtcgcgcctcttagagcgagag
105 100 95 90 85 80
Gln Leu Glu Arg Lys Ala Pro Tyr Arg Trp Pro Ile Ile Asp Asp His Val Val Ile Thr Val Glu Val Ala Arg Leu Ile Glu Ser Glu
SmR

tccaggggaagccgaagtttccaaaaggtcgttgatcaaagctcgccgcgttgtttcatcaagccttacggtcaccgtaaccagcaaatc 5130
aggtccccttcggcttcaaaggttttccagcaactagtttcgagcggcgcaacaaagtagttcggaatgccagtggcattggtcgtttag
75 70 65 60 55 50
Gly Pro Ser Ala Ser Thr Glu Leu Leu Asp Asn Ile Leu Ala Arg Arg Thr Thr Glu Asp Leu Arg Val Thr Val Thr Val Leu Leu Asp
SmR

aatatcactgtgtggcttcaggccgccatccactgcggagccgtacaaatgtacggccagcaacgtcggttcgagatggcgctcgatgac 5220
ttatagtgacacaccgaagtccggcggtaggtgacgcctcggcatgtttacatgccggtcgttgcagccaagctctaccgcgagctactg
45 40 35 30 25 20
Ile Asp Ser His Pro Lys Leu Gly Gly Asp Val Ala Ser Gly Tyr Leu His Val Ala Leu Leu Thr Pro Glu Leu His Arg Glu Ile Val
SmR

gccaactacctctgatagttgagtcgatacttcggcgatcaccgcttccctcatactcttcctttttcaatattattgaagcatttatca 5310
cggttgatggagactatcaactcagctatgaagccgctagtggcgaagggagtatgagaaggaaaaagttataataacttcgtaaatagt
15 10 5 1
Gly Val Val Glu Ser Leu Gln Thr Ser Val Glu Ala Ile Val Ala Glu Arg Met
SmR | AmpR promoter

gggttattgtctcatgagcggatacatatttgaatgtatttagaaaaataaacaaatagctagctcactcggtcgctacgctccgggcgt 5400
cccaataacagagtactcgcctatgtataaacttacataaatctttttatttgtttatcgatcgagtgagccagcgatgcgaggcccgca
AmpR promoter

gagactgcggcgggcgctgcggacacatacaaagttacccacagattccgtggataagcaggggactaacatgtgaggcaaaacagcagg 5490
ctctgacgccgcccgcgacgcctgtgtatgtttcaatgggtgtctaaggcacctattcgtcccctgattgtacactccgttttgtcgtcc
CloDF13 ori

gccgcgccggtggcgtttttccataggctccgccctcctgccagagttcacataaacagacgctttttccggtgcatctgtgggagccgtg 5580
cggcgcggccaccgcaaaaaggtatccgaggcgggaggacggtctcaagtgtatttgtctgcgaaaaggccacgtagacaccctcggcac
CloDF13 ori

aggctcaaccatgaatctgacagtacgggcgaaacccgacaggacttaaagatccccaccgtttccggcgggtcgctccctcttgcgctc 5670
tccgagttggtacttagactgtcatgcccgctttgggctgtcctgaatttctaggggtggcaaaggccgcccagcgagggagaacgcgag
CloDF13 ori

tcctgttccgaccctgccgtttaccggatacctgttccgcctttctcccttacgggaagtgtggcgctttctcatagctcacacactggt 5760
aggacaaggctgggacggcaaatggcctatggacaaggcggaaagagggaatgcccttcacaccgcgaaagagtatcgagtgtgtgacca
CloDF13 ori

atctcggctcggtgtaggtcgttcgctccaagctgggctgtaagcaagaactccccgttcagcccgactgctgcgccttatccggtaact 5850
tagagccgagccacatccagcaagcgaggttcgacccgacattcgttcttgaggggcaagtcgggctgacgacgcggaataggccattga
CloDF13 ori

gttcacttgagtccaacccggaaaagcacggtaaaacgccactggcagcagccattggtaactgggagttcgcagaggatttgtttagct 5940
caagtgaactcaggttgggccttttcgtgccattttgcggtgaccgtcgtcggtaaccattgaccctcaagcgtctcctaaacaaatcga
CloDF13 ori

aaacacgcggttgctcttgaagtgtgcgccaaagtccggctacactggaaggacagatttggttgctgtgctctgcgaaagccagttacc 6030
tttgtgcgccaacgagaacttcacacgcggtttcaggccgatgtgaccttcctgtctaaaccaacgacacgagacgctttcggtcaatgg
CloDF13 ori

acggttaagcagttccccaactgacttaaccttcgatcaaaccacctccccaggtggtttttcgtttacagggcaaaagattacgcgca 6120
tgccaattcgtcaaggggttgactgaattggaagctagtttggtggaggggtccaccaaaaaagcaaatgtcccgttttctaatgcgcgt
CloDF13 ori

gaaaaaaaggatctcaagaagatcctttgatcttttctactgaaccgctctagatttcagtgcaatttatctcttcaaatgtagcacctg 6210
ctttttttcctagagttcttctaggaaactagaaaagatgacttggcgagatctaaagtcacgttaaatagagaagtttacatcgtggac
CloDF13 ori

aagtcagccccatacgatataagttgtaattctcatgttagtcatgccccgcgcccaccggaaggagctgactgggttgaaggctctcaa 6300
ttcagtcggggtatgctatattcaacattaagagtacaatcagtacggggcgcgggtggccttcctcgactgacccaacttccgagagtt

gggcatcggtcgagatcccggtgcctaatgagtgagctaacttacattaattgcgttgcgctcactgcccgctttccagtcgggaaacct 6390
cccgtagccagctctagggccacggattactcactcgattgaatgtaattaacgcaacgcgagtgacgggcgaaaggtcagccctttgga
360 355
* Gln Gly Ser Glu Leu Arg Ser Val Gln
lacI

gtcgtgccagctgcattaatgaatcggccaacgcgcggggagaggcggtttgcgtattgggcgccagggtggtttttcttttcaccagtg 6480
cagcacggtcgacgtaattacttagccggttgcgcgcccctctccgccaaacgcataacccgcggtcccaccaaaaagaaaagtggtcac
350 345 340 335 330 325
Arg Ala Leu Gln Met Leu Ser Asp Ala Leu Ala Arg Pro Ser Ala Thr Gln Thr Asn Pro Ala Leu Thr Thr Lys Arg Lys Val Leu Ser
lacI

agacgggcaacagctgattgcccttcaccgcctggccctgagagagttgcagcaagcggtccacgctggtttgccccagcaggcgaaaat 6570
tctgcccgttgtcgactaacgggaagtggcggaccgggactctctcaacgtcgttcgccaggtgcgaccaaacggggtcgtccgctttta
320 315 310 305 300 295
Val Pro Leu Leu Gln Asn Gly Lys Val Ala Gln Gly Gln Ser Leu Gln Leu Leu Arg Asp Val Ser Thr Gln Gly Leu Leu Arg Phe Asp
lacI

cctgtttgatggtggttaacggcgggatataacatgagctgtcttcggtatcgtcgtatcccactaccgagatgtccgcaccaacgcgca 6660
ggacaaactaccaccaattgccgccctatattgtactcgacagaagccatagcagcatagggtgatggctctacaggcgtggttgcgcgt
290 285 280 275 270 265
Gln Lys Ile Thr Thr Leu Pro Pro Ile Tyr Cys Ser Ser Asp Glu Thr Asp Asp Tyr Gly Val Val Ser Ile Asp Ala Gly Val Arg Leu
lacI

gcccggactcggtaatggcgcgcattgcgcccagcgccatctgatcgttggcaaccagcatcgcagtgggaacgatgccctcattcagca 6750
cgggcctgagccattaccgcgcgtaacgcgggtcgcggtagactagcaaccgttggtcgtagcgtcacccttgctacgggagtaagtcgt
260 255 250 245 240 235
Gly Ser Glu Thr Ile Ala Arg Met Ala Gly Leu Ala Met Gln Asp Asn Ala Val Leu Met Ala Thr Pro Val Ile Gly Glu Asn Leu Met
lacI

tttgcatggtttgttgaaaaccggacatggcactccagtcgccttcccgttccgctatcggctgaatttgattgcgagtgagatatttat 6840
aaacgtaccaaacaacttttggcctgtaccgtgaggtcagcggaagggcaaggcgatagccgacttaaactaacgctcactctataaata
230 225 220 215 210 205
Gln Met Thr Gln Gln Phe Gly Ser Met Ala Ser Trp Asp Gly Glu Arg Glu Ala Ile Pro Gln Ile Gln Asn Arg Thr Leu Tyr Lys His
lacI

gccagccagccagacgcagacgcgccgagacagaacttaatgggcccgctaacagcgcgatttgctggtgacccaatgcgaccagatgct 6930
cggtcggtcggtctgcgtctgcgcggctctgtcttgaattacccgggcgattgtcgcgctaaacgaccactgggttacgctggtctacga
200 195 190 185 180 175
Trp Gly Ala Leu Arg Leu Arg Ala Ser Val Ser Ser Leu Pro Gly Ala Leu Leu Ala Ile Gln Gln His Gly Leu Ala Val Leu His Glu
lacI

ccacgcccagtcgcgtaccgtcttcatgggagaaaataatactgttgatgggtgtctggtcagagacatcaagaaataacgccggaacat 7020
ggtgcgggtcagcgcatggcagaagtaccctcttttattatgacaactacccacagaccagtctctgtagttctttattgcggccttgta
170 165 160 155 150 145
Val Gly Leu Arg Thr Gly Asp Glu His Ser Phe Ile Ile Ser Asn Ile Pro Thr Gln Asp Ser Val Asp Leu Phe Leu Ala Pro Val Asn
lacI

tagtgcaggcagcttccacagcaatggcatcctggtcatccagcggatagttaatgatcagcccactgacgcgttgcgcgagaagattgt 7110
atcacgtccgtcgaaggtgtcgttaccgtaggaccagtaggtcgcctatcaattactagtcgggtgactgcgcaacgcgctcttctaaca
140 135 130 125 120 115
Thr Cys Ala Ala Glu Val Ala Ile Ala Asp Gln Asp Asp Leu Pro Tyr Asn Ile Ile Leu Gly Ser Val Arg Gln Ala Leu Leu Asn His
lacI

gcaccgccgctttacaggcttcgacgccgcttcgttctaccatcgacaccaccacgctggcacccagttgatcggcgcgagatttaatcg 7200
cgtggcggcgaaatgtccgaagctgcggcgaagcaagatggtagctgtggtggtgcgaccgtgggtcaactagccgcgctctaaattagc
110 105 100 95 90 85
Val Ala Ala Lys Cys Ala Glu Val Gly Ser Arg Glu Val Met Ser Val Val Val Ser Ala Gly Leu Gln Asp Ala Arg Ser Lys Ile Ala
lacI

ccgcgacaatttgcgacggcgcgtgcagggccagactggaggtggcaacgccaatcagcaacgactgtttgcccgccagttgttgtgcca 7290
ggcgctgttaaacgctgccgcgcacgtcccggtctgacctccaccgttgcggttagtcgttgctgacaaacgggcggtcaacaacacggt
80 75 70 65 60 55
Ala Val Ile Gln Ser Pro Ala His Leu Ala Leu Ser Ser Thr Ala Val Gly Ile Leu Leu Ser Gln Lys Gly Ala Leu Gln Gln Ala Val
lacI

cgcggttgggaatgtaattcagctccgccatcgccgcttccactttttcccgcgttttcgcagaaacgtggctggcctggttcaccacgc 7380
gcgccaacccttacattaagtcgaggcggtagcggcgaaggtgaaaaagggcgcaaaagcgtctttgcaccgaccggaccaagtggtgcg
50 45 40 35 30 25
Arg Asn Pro Ile Tyr Asn Leu Glu Ala Met Ala Ala Glu Val Lys Glu Arg Thr Lys Ala Ser Val His Ser Ala Gln Asn Val Val Arg
lacI

```
gggaaacggtctgataagagacaccggcatactctgcgacatcgtataacgttactggtttcacattcaccaccctgaattgactcttt
                                                                                          7470
ccctttgccagactattctctgtggccgtatgagacgctgtagcatattgcaatgaccaaagtgtaagtggtgggacttaactgagagaa

   20               15                  10                  5             1
Ser Val Thr Gln Tyr Ser Val Gly Ala Tyr Glu Ala Val Asp Tyr Leu Thr Val Pro Lys Met
lacI                                                                      lacI promoter

ccgggcgctatcatgccataccgcgaaaggttttgcgccattcgatggtgtcc      3'
                                                       ...    7523
ggcccgcgatagtacggtatggcgctttccaaaacgcggtaagctaccacagg      5'
lacI promoter
```

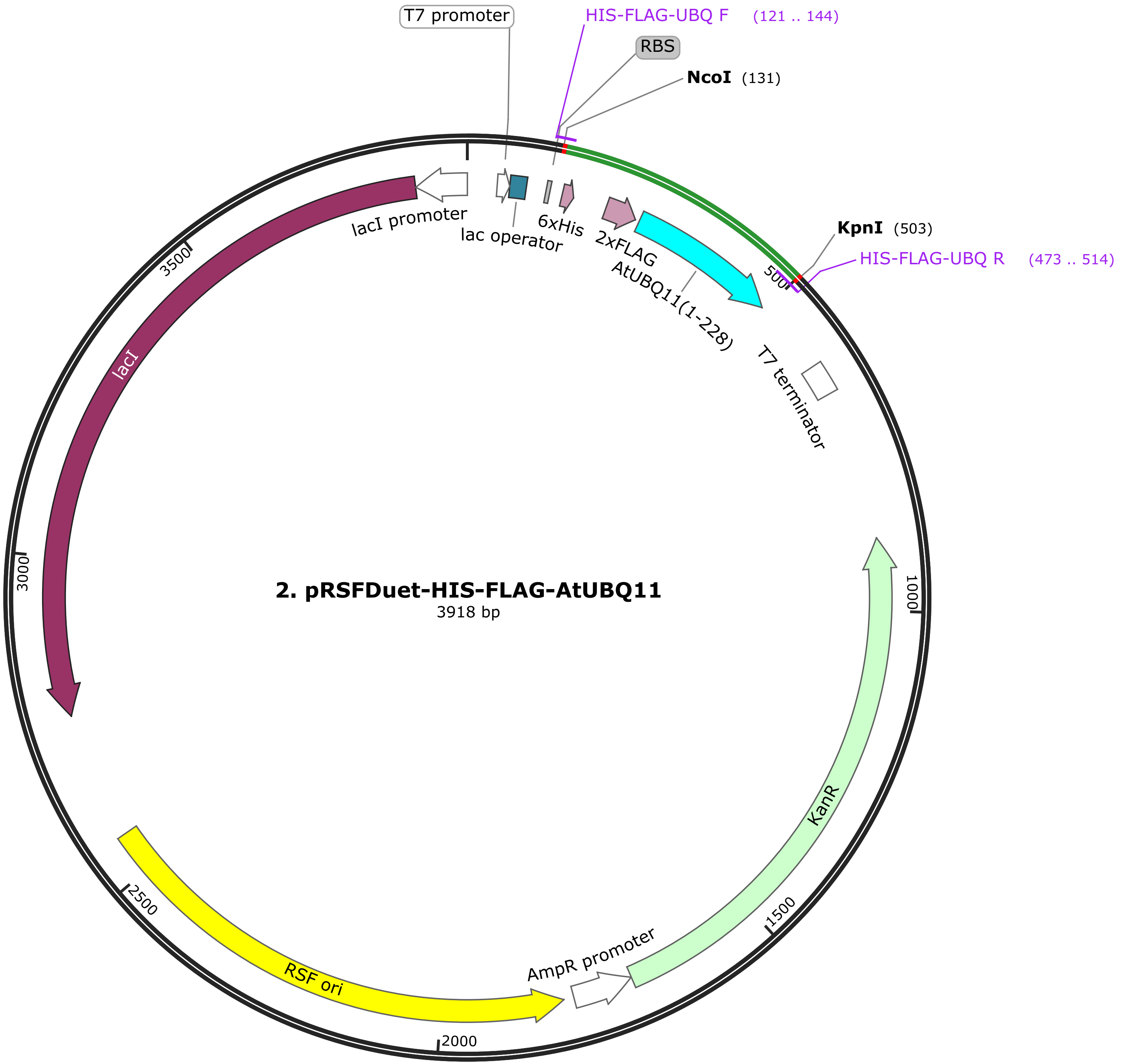

Created with SnapGene®
T7 promoter
HIS-FLAG-UBQ F (121 .. 144)
RBS
NcoI (131)
KpnI (503)
HIS-FLAG-UBQ R (473 .. 514)
lacI promoter
lac operator
6xHis
2xFLAG
AtUBQ11(1-228)
T7 terminator
lacI
KanR
AmpR promoter
RSF ori
3500
3000
2500
2000
1500
1000
500
2. pRSFDuet-HIS-FLAG-AtUBQ11
3918 bp

Sequence: 2. pRSFDuet-HIS-FLAG-AtUBQ11.dna (Circular / 3918 bp)
Enzymes: < Unsaved Enzyme Set > (2 of 678 total)
Features: 14 total
Primers: 3 total

Unique Cutters **Bold**

```
5' cgggatctcgacgctctcccttatgcgactcctgcattaggaaattaatacgactcactataggggaattgtgagcggataacaattccc   90
3' gccctagagctgcgagagggaatacgctgaggacgtaatcctttaattatgctgagtgatatccccttaacactcgcctattgttaaggg
```

T7 promoter | lac operator

NcoI

HIS-FLAG-UBQ F: aggagatataccatgggcagcagc

```
ctgtagaaataattttgtttaactttaatAAGGAGatataccatgggcagcagccatcatcatcatcatcacagcagcggcctggtgccg   180
gacatctttattaaaacaaattgaaattaTTCCTCtatatggtacccgtcgtcggtagtagtagtagtagtgtcgtcgccggaccacggc
```

1 His His His His 5 His His Ser Ser Gly Leu Val Pro

RBS | 6xHis | (in frame with 6xHis)

```
cgcggcagccatatggctagcTCTAGAATGTCTGACTACAAGGACGACGATGACAAGGACTACAAGGACGACGATGACAAGGGATCCATG   270
gcgccgtcggtataccgatcgAGATCTTACAGACTGATGTTCCTGCTGCTACTGTTCCTGATGTTCCTGCTGCTACTGTTCCCTAGGTAC
```

Arg Gly Ser His Met Ala Ser Ser Arg Met Ser | 1 Asp Tyr Lys Asp 5 Asp Asp Asp Lys Asp 10 Tyr Lys Asp Asp Asp 15 Asp Lys | Gly Ser | 1 Met

(in frame with 6xHis) | 2xFLAG | AtUBQ11(1-228)

```
CAGATCTTTGTTAAGACTCTCACCGGAAAGACTATCACCCTCGAGGTGGAAAGCTCTGACACCATCGACAACGTTAAGGCCAAGATCCAG   360
GTCTAGAAACAATTCTGAGAGTGGCCTTTCTGATAGTGGGAGCTCCACCTTTCGAGACTGTGGTAGCTGTTGCAATTCCGGTTCTAGGTC
```

Gln Ile Phe 5 Val Lys Thr Leu Thr 10 Gly Lys Thr Ile Thr 15 Leu Glu Val Glu Ser 20 Ser Asp Thr Ile Asp 25 Asn Val Lys Ala Lys 30 Ile Gln

AtUBQ11(1-228)

```
GATAAGGAAGGTATTCCTCCGGATCAGCAGAGGCTTATCTTCGCCGGAAAGCAGTTGGAGGATGGCCGCACGTTGGCGGATTACAATATC   450
CTATTCCTTCCATAAGGAGGCCTAGTCGTCTCCGAATAGAAGCGGCCTTTCGTCAACCTCCTACCGGCGTGCAACCGCCTAATGTTATAG
```

Asp Lys Glu 35 Gly Ile Pro Pro Asp 40 Gln Gln Arg Leu Ile 45 Phe Ala Gly Lys Gln 50 Leu Glu Asp Gly Arg 55 Thr Leu Ala Asp Tyr 60 Asn Ile

AtUBQ11(1-228)

KpnI

```
CAGAAGGAATCCACCCTCCACTTGGTCCTCAGGCTCCGTGGTGGTTAAggtaccctcgagtctggtaaagaaaccgctgctgcgaaattt   540
GTCTTCCTTAGGTGGGAGGTGAACCAGGAGTCCGAGGCACCACCAATTccatgggagctcagaccatttctttggcgacgacgctttaaa
```

Gln Lys Glu 65 Ser Thr Leu His Leu 70 Val Leu Arg Leu Arg 75 Gly Gly

AtUBQ11(1-228)

ACCAGGAGTCCGAGGCACCACCAATTccatgggagctcagac

HIS-FLAG-UBQ R

```
gaacgccagcacatggactcgtctactagcgcagcttaattaacctaggctgctgccaccgctgagcaataactagcataaccccttggg   630
cttgcggtcgtgtacctgagcagatgatcgcgtcgaattaattggatccgacgacggtggcgactcgttattgatcgtattggggaaccc
```

T7 terminator

```
gcctctaaacgggtcttgaggggttttttgctgaaacctcaggcatttgagaagcacacggtcacactgcttccggtagtcaataaaccg   720
cggagatttgcccagaactccccaaaaaacgactttggagtccgtaaactcttcgtgtgccagtgtgacgaaggccatcagttatttggc
```

T7 terminator

```
gtaaaccagcaatagacataagcggctatttaacgaccctgccctgaaccgacgacaagctgacgaccgggtctccgcaagtggcacttt   810
catttggtcgttatctgtattcgccgataaattgctgggacgggacttggctgctgttcgactgctggcccagaggcgttcaccgtgaaa
```

```
tcggggaaatgtgcgcggaacccctatttgtttatttttctaaatacattcaaatatgtatccgctcatgaattaattcttagaaaaact   900
agccccttttacacgcgccttggggataaacaaataaaaagatttatgtaagtttatacataggcgagtacttaattaagaatcttttga
```

270 Phe Phe Glu

KanR

```
catcgagcatcaaatgaaactgcaatttattcatatcaggattatcaataccatatttttgaaaaagccgtttctgtaatgaaggagaaa   990
gtagctcgtagtttactttgacgttaaataagtatagtcctaatagttatggtataaaaactttttcggcaaagacattacttcctcttt
   265        260        255        250        245        240
Asp Leu Met Leu His Phe Gln Leu Lys Asn Met Asp Pro Asn Asp Ile Gly Tyr Lys Gln Phe Leu Arg Lys Gln Leu Ser Pro Ser Phe
KanR

actcaccgaggcagttccataggatggcaagatcctggtatcggtctgcgattccgactcgtccaacatcaatacaacctattaatttcc   1080
tgagtggctccgtcaaggtatcctaccgttctaggaccatagccagacgctaaggctgagcaggttgtagttatgttggataattaaagg
   235        230        225        220        215        210
Glu Gly Leu Cys Asn Trp Leu Ile Ala Leu Asp Gln Tyr Arg Asp Ala Ile Gly Val Arg Gly Val Asp Ile Cys Gly Ile Leu Lys Gly
KanR

cctcgtcaaaaataaggttatcaagtgagaaatcaccatgagtgacgactgaatccggtgagaatggcaaaagtttatgcatttctttcc   1170
ggagcagtttttattccaatagttcactctttagtggtactcactgctgacttaggccactcttaccgttttcaaatacgtaaagaaagg
   205        200        195        190        185        180
Glu Asp Phe Ile Leu Asn Asp Leu Ser Phe Asp Gly His Thr Val Val Ser Asp Pro Ser Phe Pro Leu Leu Lys His Met Glu Lys Trp
KanR

agacttgttcaacaggccagccattacgctcgtcatcaaaatcactcgcatcaaccaaaccgttattcattcgtgattgcgcctgagcga   1260
tctgaacaagttgtccggtcggtaatgcgagcagtagttttagtgagcgtagttggtttggcaataagtaagcactaacgcggactcgct
   175        170        165        160        155        150
Val Gln Glu Val Pro Trp Gly Asn Arg Glu Asp Asp Phe Asp Ser Ala Asp Val Leu Gly Asn Asn Met Arg Ser Gln Ala Gln Ala Leu
KanR

gacgaaatacgcggtcgctgttaaaaggacaattacaaacaggaatcgaatgcaaccggcgcaggaacactgccagcgcatcaacaatat   1350
ctgctttatgcgccagcgacaattttcctgttaatgtttgtccttagcttacgttggccgcgtccttgtgacggtcgcgtagttgttata
   145        140        135        130        125        120
Arg Phe Val Arg Asp Ser Asn Phe Pro Cys Asn Cys Val Pro Ile Ser His Leu Arg Arg Leu Phe Val Ala Leu Ala Asp Val Ile Asn
KanR

tttcacctgaatcaggatattcttctaatacctggaatgctgttttcccggggatcgcagtggtgagtaaccatgcatcatcaggagtac   1440
aaagtggacttagtcctataagaagattatggaccttacgacaaaagggcccctagcgtcaccactcattggtacgtagtagtcctcatg
   115        110        105        100        95         90
Glu Gly Ser Asp Pro Tyr Glu Glu Leu Val Gln Phe Ala Thr Lys Gly Pro Ile Ala Thr Thr Leu Leu Trp Ala Asp Asp Pro Thr Arg
KanR

ggataaaatgcttgatggtcggaagaggcataaattccgtcagccagtttagtctgaccatctcatctgtaacatcattggcaacgctac   1530
cctattttacgaactaccagccttctccgtatttaaggcagtcggtcaaatcagactggtagagtagacattgtagtaaccgttgcgatg
   85         80         75         70         65         60
Ile Phe His Lys Ile Thr Pro Leu Pro Met Phe Glu Thr Leu Trp Asn Leu Arg Val Met Glu Asp Thr Val Asp Asn Ala Val Ser Gly
KanR

ctttgccatgtttcagaaacaactctggcgcatcgggcttcccatacaatcgatagattgtcgcacctgattgcccgacattatcgcgag   1620
gaaacggtacaaagtctttgttgagaccgcgtagcccgaagggtatgttagctatctaacagcgtggactaacgggctgtaatagcgctc
   55         50         45         40         35         30
Lys Gly His Lys Leu Phe Leu Glu Pro Ala Asp Pro Lys Gly Tyr Leu Arg Tyr Ile Thr Ala Gly Ser Gln Gly Val Asn Asp Arg Ala
KanR

cccatttatacccatataaatcagcatccatgttggaatttaatcgcggcctagagcaagacgtttcccgttgaatatggctcatactct   1710
gggtaaatatgggtatatttagtcgtaggtacaaccttaaattagcgccggatctcgttctgcaaagggcaacttataccgagtatgaga
   25         20         15         10         5          1
Trp Lys Tyr Gly Tyr Leu Asp Ala Asp Met Asn Ser Asn Leu Arg Pro Arg Ser Cys Ser Thr Glu Arg Gln Ile His Ser Met
KanR
AmpR promoter

tcctttttcaatattattgaagcatttatcagggttattgtctcatgagcggatacatatttgaatgtatttagaaaaataaacaaatag   1800
aggaaaaagttataataacttcgtaaatagtcccaataacagagtactcgcctatgtataaacttacataaatctttttatttgtttatc
AmpR promoter

gcatgcagcgctcttccgcttcctcgctcactgactcgctacgctcggtcgttcgactgcggcgagcggtgtcagctcactcaaaagcgg   1890
cgtacgtcgcgagaaggcgaaggagcgagtgactgagcgatgcgagccagcaagctgacgccgctcgccacagtcgagtgagttttcgcc
RSF ori
```

taatacggttatccacagaatcaggggataaagccggaaagaacatgtgagcaaaaagcaaagcaccggaagaagccaacgccgcaggcg 1980
attatgccaataggtgtcttagtcccctatttcggcctttcttgtacactcgtttttcgtttcgtggccttcttcggttgcggcgtccgc
RSF ori

tttttccataggctccgcccccctgacgagcatcacaaaaatcgacgctcaagccagaggtggcgaaacccgacaggactataaagatac 2070
aaaaaggtatccgaggcggggggactgctcgtagtgtttttagctgcgagttcggtctccaccgctttgggctgtcctgatatttctatg
RSF ori

caggcgtttccccctggaagctccctcgtgcgctctcctgttccgaccctgccgcttaccggatacctgtccgcctttctcccttcggga 2160
gtccgcaaagggggaccttcgagggagcacgcgagaggacaaggctgggacggcgaatggcctatggacaggcggaaagagggaagccct
RSF ori

agcgtggcgctttctcatagctcacgctgttggtatctcagttcggtgtaggtcgttcgctccaagctgggctgtgtgcacgaacccccc 2250
tcgcaccgcgaaagagtatcgagtgcgacaaccatagagtcaagccacatccagcaagcgaggttcgacccgacacacgtgcttgggggg
RSF ori

gttcagcccgaccgctgcgccttatccggtaactatcgtcttgagtccaacccggtaagacacgacttatcgccactggcagcagccatt 2340
caagtcgggctggcgacgcggaataggccattgatagcagaactcaggttgggccattctgtgctgaatagcggtgaccgtcgtcggtaa
RSF ori

ggtaactgatttagaggactttgtcttgaagttatgcacctgttaaggctaaactgaaagaacagatttggtgagtgcggtcctccaac 2430
ccattgactaaatctcctgaaacagaacttcaatacgtggacaattccgatttgactttcttgtctaaaccactcacgccaggaggttg
RSF ori

ccacttaccttggttcaaagagttggtagctcagcgaaccttgagaaaaccaccgttggtagcggtggtttttctttatttatgagatga 2520
ggtgaatggaaccaagtttctcaaccatcgagtcgcttggaactcttttggtggcaaccatcgccaccaaaaagaaataaatactctact
RSF ori

tgaatcaatcggtctatcaagtcaacgaacagctattccgttactctagatttcagtgcaatttatctcttcaaatgtagcacctgaagt 2610
acttagttagccagatagttcagttgcttgtcgataaggcaatgagatctaaagtcacgttaaatagagaagtttacatcgtggacttca
RSF ori

cagccccatacgatataagttgtaattctcatgttagtcatgccccgcgcccaccggaaggagctgactgggttgaaggctctcaagggc 2700
gtcggggtatgctatattcaacattaagagtacaatcagtacggggcgcgggtggccttcctcgactgacccaacttccgagagttcccg

atcggtcgagatcccggtgcctaatgagtgagctaacttacattaattgcgttgcgctcactgcccgctttccagtcgggaaacctgtcg 2790
tagccagctctagggccacggattactcactcgattgaatgtaattaacgcaacgcgagtgacgggcgaaaggtcagccctttggacagc
360 355
* Gln Gly Ser Glu Leu Arg Ser Val Gln Arg
lacI

tgccagctgcattaatgaatcggccaacgcgcggggagaggcggtttgcgtattgggcgccagggtggtttttcttttcaccagtgagac 2880
acggtcgacgtaattacttagccggttgcgcgcccctctccgccaaacgcataacccgcggtcccaccaaaaagaaaagtggtcactctg
350 345 340 335 330 325
Ala Leu Gln Met Leu Ser Asp Ala Leu Ala Arg Pro Ser Ala Thr Gln Thr Asn Pro Ala Leu Thr Thr Lys Arg Lys Val Leu Ser Val
lacI

gggcaacagctgattgcccttcaccgcctggccctgagagagttgcagcaagcggtccacgctggtttgccccagcaggcgaaaatcctg 2970
cccgttgtcgactaacgggaagtggcggaccgggactctctcaacgtcgttcgccaggtgcgaccaaacggggtcgtccgcttttaggac
320 315 310 305 300 295
Pro Leu Leu Gln Asn Gly Lys Val Ala Gln Gly Gln Ser Leu Gln Leu Leu Arg Asp Val Ser Thr Gln Gly Leu Leu Arg Phe Asp Gln
lacI

tttgatggtggttaacggcgggatataacatgagctgtcttcggtatcgtcgtatcccactaccgagatgtccgcaccaacgcgcagccc 3060
aaactaccaccaattgccgccctatattgtactcgacagaagccatagcagcatagggtgatggctctacaggcgtggttgcgcgtcggg
290 285 280 275 270 265
Lys Ile Thr Thr Leu Pro Pro Ile Tyr Cys Ser Ser Asp Glu Thr Asp Asp Tyr Gly Val Val Ser Ile Asp Ala Gly Val Arg Leu Gly
lacI

ggactcggtaatggcgcgcattgcgcccagcgccatctgatcgttggcaaccagcatcgcagtgggaacgatgccctcattcagcatttg 3150
cctgagccattaccgcgcgtaacgcgggtcgcggtagactagcaaccgttggtcgtagcgtcacccttgctacgggagtaagtcgtaaac
260 255 250 245 240 235
Ser Glu Thr Ile Ala Arg Met Ala Gly Leu Ala Met Gln Asp Asn Ala Val Leu Met Ala Thr Pro Val Ile Gly Glu Asn Leu Met Gln
lacI

catggtttgttgaaaaccggacatggcactccagtcgccttcccgttccgctatcggctgaatttgattgcgagtgagatatttatgcca 3240
gtaccaaacaacttttggcctgtaccgtgaggtcagcggaagggcaaggcgatagccgacttaaactaacgctcactctataaatacggt
230 225 220 215 210 205
Met Thr Gln Gln Phe Gly Ser Met Ala Ser Trp Asp Gly Glu Arg Glu Ala Ile Pro Gln Ile Gln Asn Arg Thr Leu Tyr Lys His Trp
lacI

gccagccagacgcagacgcgccgagacagaacttaatgggcccgctaacagcgcgatttgctggtgacccaatgcgaccagatgctccac 3330
cggtcggtctgcgtctgcgcggctctgtcttgaattacccgggcgattgtcgcgctaaacgaccactgggttacgctggtctacgaggtg
200 195 190 185 180 175
Gly Ala Leu Arg Leu Arg Ala Ser Val Ser Ser Leu Pro Gly Ala Leu Leu Ala Ile Gln Gln His Gly Leu Ala Val Leu His Glu Val
lacI

gcccagtcgcgtaccgtcttcatgggagaaaataatactgttgatgggtgtctggtcagagacatcaagaaataacgccggaacattagt 3420
cgggtcagcgcatggcagaagtaccctcttttattatgacaactacccacagaccagtctctgtagttctttattgcggccttgtaatca
170 165 160 155 150 145
Gly Leu Arg Thr Gly Asp Glu His Ser Phe Ile Ile Ser Asn Ile Pro Thr Gln Asp Ser Val Asp Leu Phe Leu Ala Pro Val Asn Thr
lacI

gcaggcagcttccacagcaatggcatcctggtcatccagcggatagttaatgatcagcccactgacgcgttgcgcgagaagattgtgcac 3510
cgtccgtcgaaggtgtcgttaccgtaggaccagtaggtcgcctatcaattactagtcgggtgactgcgcaacgcgctcttctaacacgtg
140 135 130 125 120 115
Cys Ala Ala Glu Val Ala Ile Ala Asp Gln Asp Asp Leu Pro Tyr Asn Ile Ile Leu Gly Ser Val Arg Gln Ala Leu Leu Asn His Val
lacI

cgccgctttacaggcttcgacgccgcttcgttctaccatcgacaccaccacgctggcacccagttgatcggcgcgagatttaatcgccgc 3600
gcggcgaaatgtccgaagctgcggcgaagcaagatggtagctgtggtggtgcgaccgtgggtcaactagccgcgctctaaattagcggcg
110 105 100 95 90 85
Ala Ala Lys Cys Ala Glu Val Gly Ser Arg Glu Val Met Ser Val Val Val Ser Ala Gly Leu Gln Asp Ala Arg Ser Lys Ile Ala Ala
lacI

gacaatttgcgacggcgcgtgcagggccagactggaggtggcaacgccaatcagcaacgactgtttgcccgccagttgttgtgccacgcg 3690
ctgttaaacgctgccgcgcacgtcccggtctgacctccaccgttgcggttagtcgttgctgacaaacgggcggtcaacaacacggtgcgc
80 75 70 65 60 55
Val Ile Gln Ser Pro Ala His Leu Ala Leu Ser Ser Thr Ala Val Gly Ile Leu Leu Ser Gln Lys Gly Ala Leu Gln Gln Ala Val Arg
lacI

gttgggaatgtaattcagctccgccatcgccgcttccactttttcccgcgttttcgcagaaacgtggctggcctggttcaccacgcggga 3780
caacccttacattaagtcgaggcggtagcggcgaaggtgaaaaagggcgcaaaagcgtctttgcaccgaccggaccaagtggtgcgccct
50 45 40 35 30 25
Asn Pro Ile Tyr Asn Leu Glu Ala Met Ala Ala Glu Val Lys Glu Arg Thr Lys Ala Ser Val His Ser Ala Gln Asn Val Val Arg Ser
lacI

aacggtctgataagagacaccggcatactctgcgacatcgtataacgttactggtttcacattcaccaccctgaattgactctcttccgg 3870
ttgccagactattctctgtggccgtatgagacgctgtagcatattgcaatgaccaaagtgtaagtggtgggacttaactgagagaaggcc
20 15 10 5 1
Val Thr Gln Tyr Ser Val Gly Ala Tyr Glu Ala Val Asp Tyr Leu Thr Val Pro Lys Met
lacI lacI promoter

gcgctatcatgccataccgcgaaaggttttgcgccattcgatggtgtc 3' ••• 3918
cgcgatagtacggtatggcgctttccaaaacgcggtaagctaccacag 5'
lacI promoter

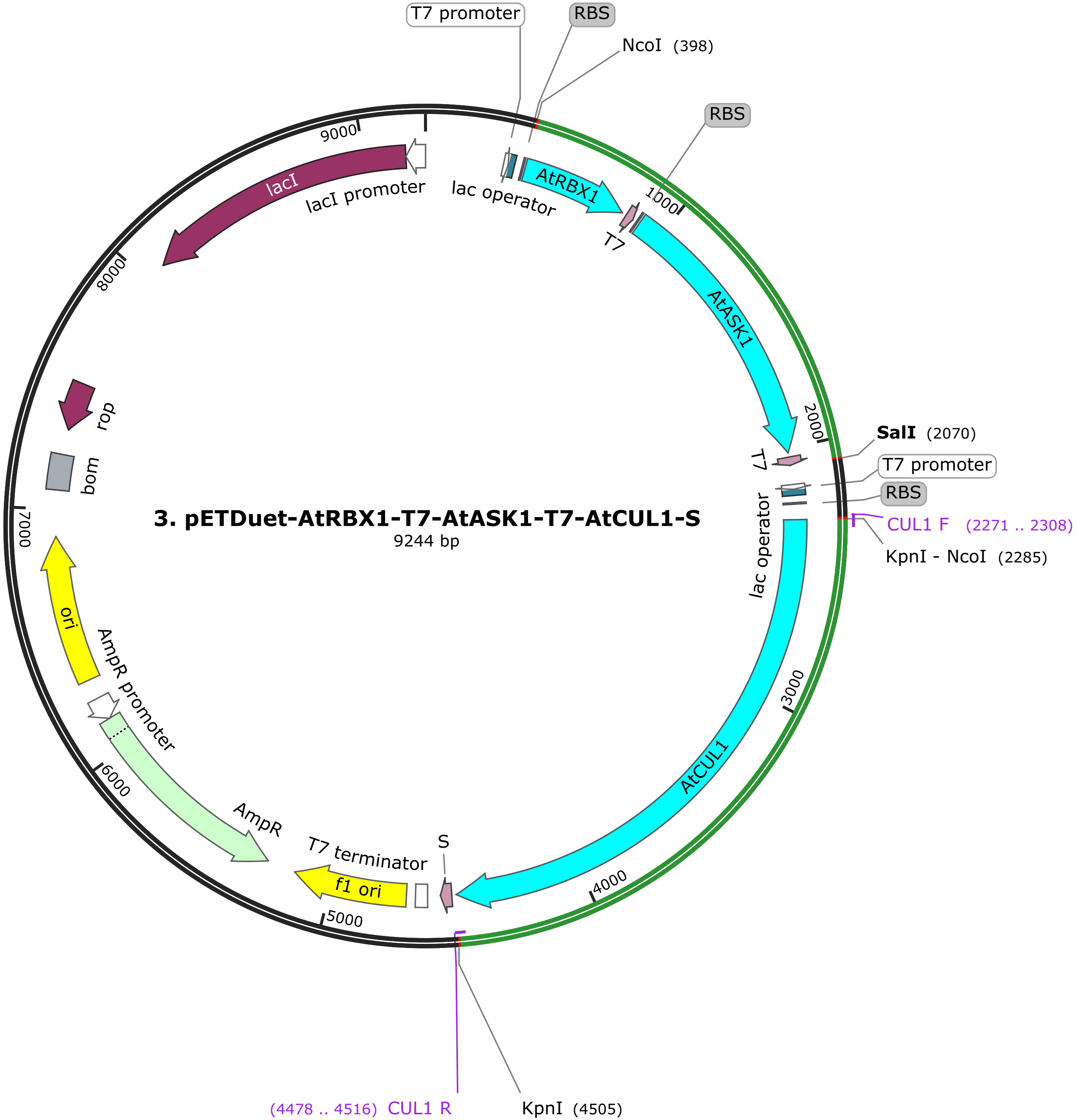

Created with SnapGene®

5′ cgggatctcgacgctctcccttatgcgactcctgcattaggaagcagcccagtagtaggttgaggccgttgagcaccgccgccgcaagga 90
3′ gccctagagctgcgagagggaatacgctgaggacgtaatccttcgtcgggtcatcatccaactccggcaactcgtggcggcggcgttcct

atggtgcatgcaaggagatggcgcccaacagtcccccggccacggggcctgccaccatacccacgccgaaacaagcgctcatgagcccga 180
taccacgtacgttcctctaccgcgggttgtcagggggccggtgccccggacggtggtatgggtgcggctttgttcgcgagtactcgggct

agtggcgagcccgatcttccccatcggtgatgtcggcgatataggcgccagcaaccgcacctgtggcgccggtgatgccggccacgatgc 270
tcaccgctcgggctagaaggggtagccactacagccgctatatccgcggtcgttggcgtggacaccgcggccactacggccggtgctacg

gtccggcgtagaggatcgagatcgatctcgatcccgcgaaattaatacgactcactataggggaattgtgagcggataacaattcccctc 360
caggccgcatctcctagctctagctagagctagggcgctttaattatgctgagtgatatccccttaacactcgcctattgttaaggggag
T7 promoter | lac operator

NcoI
tagaaataattttgtttaactttaagAAGGAGatataccATGGCGACTCTAGACTCCGACGTTACCATGATTCCTGCCGGAGAAGCCTCC 450
atctttattaaaacaaattgaaattcTTCCTCtatatggTACCGCTGAGATCTGAGGCTGCAATGGTACTAAGGACGGCCTCTTCGGAGG
1 Met Ala Thr Leu 5 Asp Ser Asp Val Thr 10 Met Ile Pro Ala Gly 15 Glu Ala Ser
RBS | AtRBX1

AGCAGCGTAGCCGCGTCGTCTTCCAACAAGAAAGCTAAGCGATTCGAAATTAAGAAGTGGAGCGCCGTTGCTCTCTGGGCTTGGGATATC 540
TCGTCGCATCGGCGCAGCAGAAGGTTGTTCTTTCGATTCGCTAAGCTTTAATTCTTCACCTCGCGGCAACGAGAGACCCGAACCCTATAG
Ser Ser 20 Val Ala Ala Ser Ser 25 Ser Asn Lys Lys Ala 30 Lys Arg Phe Glu Ile 35 Lys Lys Trp Ser Ala 40 Val Ala Leu Trp Ala 45 Trp Asp Ile
AtRBX1

GTTGTTGACAACTGTGCGATCTGCAGAAACCACATCATGGATCTTTGTATCGAGTGTCAGGCTAATCAGGCCAGTGCCACAAGTGAAGAG 630
CAACAACTGTTGACACGCTAGACGTCTTTGGTGTAGTACCTAGAAACATAGCTCACAGTCCGATTAGTCCGGTCACGGTGTTCACTTCTC
Val Val 50 Asp Asn Cys Ala Ile 55 Cys Arg Asn His Ile 60 Met Asp Leu Cys Ile 65 Glu Cys Gln Ala Asn 70 Gln Ala Ser Ala Thr 75 Ser Glu Glu
AtRBX1

TGCACTGTAGCTTGGGAGGACGACCAAAATAACTGCAATAAGTATTTCTGTATTCTTGATTGCTCAATGAAAGATGACCACTTGGAGGGG 720
ACGTGACATCGAACCCTCCTGCTGGTTTTATTGACGTTATTCATAAAGACATAAGAACTAACGAGTTACTTTCTACTGGTGAACCTCCCC
Cys Thr 80 Val Ala Trp Glu Asp 85 Asp Gln Asn Asn Cys 90 Asn Lys Tyr Phe Cys 95 Ile Leu Asp Cys Ser 100 Met Lys Asp Asp His 105 Leu Glu Gly
AtRBX1

GTTTGCAATCACGCCTTCCACTTTCACTGCATCAGCAGATGGCTAAAGACTCGTCAAGTTTGTCCATTGGATAACAGTGAGTGGGAGTTT 810
CAAACGTTAGTGCGGAAGGTGAAAGTGACGTAGTCGTCTACCGATTTCTGAGCAGTTCAAACAGGTAACCTATTGTCACTCACCCTCAAA
Val Cys 110 Asn His Ala Phe His 115 Phe His Cys Ile Ser 120 Arg Trp Leu Lys Thr 125 Arg Gln Val Cys Pro 130 Leu Asp Asn Ser Glu 135 Trp Glu Phe
AtRBX1

CAGAAATATGGTCACagccaggatccgaattcgatggctagcatgactggtggacagcaaatgggtCCAtaaCCCCGAAAGAAGGAGATA 900
GTCTTTATACCAGTGtcggtcctaggcttaagctaccgatcgtactgaccacctgtcgtttacccaGGTattGGGGCTTTCTTCCTCTAT
Gln Lys 140 Tyr Gly His Ser Gln Asp Pro Asn Ser 1 Met Ala Ser Met 5 Thr Gly Gly Gln Gln 10 Met Gly Pro *
AtRBX1 (in frame with AtRBX1) T7 | RBS

TACATATGGACAAGTACGAGCTGGTGAAAGACATAGGTGCTGGGAATTTTGGAGTTGCCAGGCTCATGAAGGTCAAAAACTCTAAGGAAC 990
ATGTATACCTGTTCATGCTCGACCACTTTCTGTATCCACGACCCTTAAAACCTCAACGGTCCGAGTACTTCCAGTTTTTGAGATTCCTTG
1 Met Asp Lys Tyr 5 Glu Leu Val Lys Asp 10 Ile Gly Ala Gly Asn 15 Phe Gly Val Ala Arg 20 Leu Met Lys Val Lys 25 Asn Ser Lys Glu
AtASK1

TTGTTGCCATGAAGTACATCGAGCGTGGTCCTAAGATTGATGAGAATGTGGCAAGAGAGATCATTAATCACAGATCACTTCGCCATCCGA 1080
AACAACGGTACTTCATGTAGCTCGCACCAGGATTCTAACTACTCTTACACCGTTCTCTCTAGTAATTAGTGTCTAGTGAAGCGGTAGGCT
Leu 30 Val Ala Met Lys Tyr 35 Ile Glu Arg Gly Pro 40 Lys Ile Asp Glu Asn 45 Val Ala Arg Glu Ile 50 Ile Asn His Arg Ser 55 Leu Arg His Pro
AtASK1

ATATAATCCGGTTCAAGGAGGTGGTGTTGACTCCAACCCATCTTGCCATTGCTATGGAATATGCTGCTGGTGGTGAACTATTCGAGCGTA 1170
TATATTAGGCCAAGTTCCTCCACCACAACTGAGGTTGGGTAGAACGGTAACGATACCTTATACGACGACCACCACTTGATAAGCTCGCAT
60 65 70 75 80 85
Asn Ile Ile Arg Phe Lys Glu Val Val Leu Thr Pro Thr His Leu Ala Ile Ala Met Glu Tyr Ala Ala Gly Gly Glu Leu Phe Glu Arg
AtASK1

TATGCAGTGCTGGAAGATTTAGTGAGGATGAGGAGGAAGGGAACAAGAGAAAACATGCGAGATATTTCTTCCAGCAGCTTATATCAGGTG 1260
ATACGTCACGACCTTCTAAATCACTCCTACTCCTCCTTCCCTTGTTCTCTTTTGTACGCTCTATAAAGAAGGTCGTCGAATATAGTCCAC
90 95 100 105 110 115
Ile Cys Ser Ala Gly Arg Phe Ser Glu Asp Glu Glu Glu Gly Asn Lys Arg Lys His Ala Arg Tyr Phe Phe Gln Gln Leu Ile Ser Gly
AtASK1

TTAGCTATTGCCATGCTATGCAAATATGCCATAGAGATCTGAAGCTCGAGAATACGCTCTTGGATGGAAGTCCTGCTCCACGTCTGAAAA 1350
AATCGATAACGGTACGATACGTTTATACGGTATCTCTAGACTTCGAGCTCTTATGCGAGAACCTACCTTCAGGACGAGGTGCAGACTTTT
120 125 130 135 140 145
Val Ser Tyr Cys His Ala Met Gln Ile Cys His Arg Asp Leu Lys Leu Glu Asn Thr Leu Leu Asp Gly Ser Pro Ala Pro Arg Leu Lys
AtASK1

TCTGTGATTTTGGTTATTCCAAGTCCTCTCTGCTGCACTCTAGGCCCAAATCAACAGTTGGAACTCCAGCATATATTGCACCTGAGGTCC 1440
AGACACTAAAACCAATAAGGTTCAGGAGAGACGACGTGAGATCCGGGTTTAGTTGTCAACCTTGAGGTCGTATATAACGTGGACTCCAGG
150 155 160 165 170 175
Ile Cys Asp Phe Gly Tyr Ser Lys Ser Ser Leu Leu His Ser Arg Pro Lys Ser Thr Val Gly Thr Pro Ala Tyr Ile Ala Pro Glu Val
AtASK1

TTTCTCGAAGAGAATATGATGGCAAGATGGCTGATGTATGGTCTTGTGGTGTGACTCTTTATGTCATGCTGGTTGGAGCATACCCATTTG 1530
AAAGAGCTTCTCTTATACTACCGTTCTACCGACTACATACCAGAACACCACACTGAGAAATACAGTACGACCAACCTCGTATGGGTAAAC
180 185 190 195 200 205
Leu Ser Arg Arg Glu Tyr Asp Gly Lys Met Ala Asp Val Trp Ser Cys Gly Val Thr Leu Tyr Val Met Leu Val Gly Ala Tyr Pro Phe
AtASK1

AAGACCAGGAAGACCCCAAGAACTTCAGGAAAACAATACAAAAAATAATGGCTGTCCAGTACAAGATCCCGGACTACGTCCATATCTCAC 1620
TTCTGGTCCTTCTGGGGTTCTTGAAGTCCTTTTGTTATGTTTTTTATTACCGACAGGTCATGTTCTAGGGCCTGATGCAGGTATAGAGTG
210 215 220 225 230 235
Glu Asp Gln Glu Asp Pro Lys Asn Phe Arg Lys Thr Ile Gln Lys Ile Met Ala Val Gln Tyr Lys Ile Pro Asp Tyr Val His Ile Ser
AtASK1

AGGATTGTAAAAATCTCCTTTCCCGTATATTTGTCGCCAATTCACTCAAGAGGATCACCATTGCAGAAATCAAGAAACATTCATGGTTCC 1710
TCCTAACATTTTTAGAGGAAAGGGCATATAAACAGCGGTTAAGTGAGTTCTCCTAGTGGTAACGTCTTTAGTTCTTTGTAAGTACCAAGG
240 245 250 255 260 265
Gln Asp Cys Lys Asn Leu Leu Ser Arg Ile Phe Val Ala Asn Ser Leu Lys Arg Ile Thr Ile Ala Glu Ile Lys Lys His Ser Trp Phe
AtASK1

TAAAGAATTTGCCAAGGGAACTCACAGAGACAGCTCAAGCTGCATATTTCAAGAAAGAGAACCCAACCTTCTCCCTTCAGACCGTTGAAG 1800
ATTTCTTAAACGGTTCCCTTGAGTGTCTCTGTCGAGTTCGACGTATAAAGTTCTTTCTCTTGGGTTGGAAGAGGGAAGTCTGGCAACTTC
270 275 280 285 290 295
Leu Lys Asn Leu Pro Arg Glu Leu Thr Glu Thr Ala Gln Ala Ala Tyr Phe Lys Lys Glu Asn Pro Thr Phe Ser Leu Gln Thr Val Glu
AtASK1

AGATCATGAAGATAGTGGCTGACGCCAAAACACCGCCTCCTGTTTCCCGATCCATCGGAGGTTTTGGCTGGGGAGGAAATGGGGATGCAG 1890
TCTAGTACTTCTATCACCGACTGCGGTTTTGTGGCGGAGGACAAAGGGCTAGGTAGCCTCCAAAACCGACCCCTCCTTTACCCCTACGTC
300 305 310 315 320 325
Glu Ile Met Lys Ile Val Ala Asp Ala Lys Thr Pro Pro Pro Val Ser Arg Ser Ile Gly Gly Phe Gly Trp Gly Gly Asn Gly Asp Ala
AtASK1

ATGGAAAAGAGGAAGATGCAGAAGACGTGGAGGAGGAAGAGGAGGAGGTGGAAGAAGAGGAAGACGATGAGGATGAATACGATAAGACTG 1980
TACCTTTTCTCCTTCTACGTCTTCTGCACCTCCTCCTTCTCCTCCTCCACCTTCTTCTCCTTCTGCTACTCCTACTTATGCTATTCTGAC
330 335 340 345 350 355
Asp Gly Lys Glu Glu Asp Ala Glu Asp Val Glu Glu Glu Glu Glu Glu Val Glu Glu Glu Glu Asp Asp Glu Asp Glu Tyr Asp Lys Thr
AtASK1

TAAAGGAAGTACACGCAAGTGGAGAAGTGAGAATAAGTagctcggcgcgcctgcagatggctagcatgactggtggacagcaaatgggtg 2070
ATTTCCTTCATGTGCGTTCACCTCTTCACTCTTATTCAtcgagccgcgcggacgtctaccgatcgtactgaccacctgtcgtttacccac

360 365 370 1 5 10
Val Lys Glu Val His Ala Ser Gly Glu Val Arg Ile Ser Ser Ser Ala Arg Leu Gln Met Ala Ser Met Thr Gly Gly Gln Gln Met Gly
AtASK1 (in frame with AtASK1) T7

SalI

tcgacaagcttgcggccgcataatgcttaagtcgaacagaaagtaatcgtattgtacacggccgcataatcgaaattaatacgactcact 2160
agctgttcgaacgccggcgtattacgaattcagcttgtctttcattagcataacatgtgccggcgtattagctttaattatgctgagtga

Val Asp Lys Leu Ala Ala Ala *
(in frame with T7)
T7 promoter

atagggggaattgtgagcggataacaattccccatcttagtatattagttaagtataagAAGGAGatatacatatggcagatctcaattgg 2250
tatcccccttaacactcgcctattgttaaggggtagaatcatataatcaattcatattcTTCCTCtatatgtataccgtctagagttaacc

T7 promoter lac operator RBS

NcoI
KpnI
CUL1 F
cgctgacgtcggtaccATGGAGCGCAAGACTATTGACT

atatcggccggccacgcgatcgctgacgtcggtaccATGGAGCGCAAGACTATTGACTTGGAGCAAGGATGGGACTATATGCAGACTGGA 2340
tatagccggccggtgcgctagcgactgcagccatggTACCTCGCGTTCTGATAACTGAACCTCGTTCCTACCCTGATATACGTCTGACCT

1 5 10 15
Met Glu Arg Lys Thr Ile Asp Leu Glu Gln Gly Trp Asp Tyr Met Gln Thr Gly
AtCUL1

ATTACTAAGCTGAAACGGATTCTGGAAGGATTGAATGAGCCAGCATTTGACTCTGAGCAATATATGATGCTTTATACGACTATCTACAAC 2430
TAATGATTCGACTTTGCCTAAGACCTTCCTAACTTACTCGGTCGTAAACTGAGACTCGTTATATACTACGAAATATGCTGATAGATGTTG

20 25 30 35 40 45
Ile Thr Lys Leu Lys Arg Ile Leu Glu Gly Leu Asn Glu Pro Ala Phe Asp Ser Glu Gln Tyr Met Met Leu Tyr Thr Thr Ile Tyr Asn
AtCUL1

ATGTGCACCCAGAAACCTCCTCATGATTACTCACAGCAGCTTTATGACAAGTATCGGGAAGCATTTGAGGAATATATTAACTCAACAGTT 2520
TACACGTGGGTCTTTGGAGGAGTACTAATGAGTGTCGTCGAAATACTGTTCATAGCCCTTCGTAAACTCCTTATATAATTGAGTTGTCAA

50 55 60 65 70 75
Met Cys Thr Gln Lys Pro Pro His Asp Tyr Ser Gln Gln Leu Tyr Asp Lys Tyr Arg Glu Ala Phe Glu Glu Tyr Ile Asn Ser Thr Val
AtCUL1

TTGCCTGCGTTGAGGGAGAAGCATGATGAGTTTATGCTGCGGGAGCTATTTAAAAGATGGTCAAACCATAAAGTAATGGTCAGATGGCTA 2610
AACGGACGCAACTCCCTCTTCGTACTACTCAAATACGACGCCCTCGATAAATTTTCTACCAGTTTGGTATTTCATTACCAGTCTACCGAT

80 85 90 95 100 105
Leu Pro Ala Leu Arg Glu Lys His Asp Glu Phe Met Leu Arg Glu Leu Phe Lys Arg Trp Ser Asn His Lys Val Met Val Arg Trp Leu
AtCUL1

TCCCGCTTCTTCTACTACCTTGACCGTTACTTCATTGCTCGGAGATCACTTCCACCACTGAATGAAGTTGGCCTGACATGCTTCCGTGAC 2700
AGGGCGAAGAAGATGATGGAACTGGCAATGAAGTAACGAGCCTCTAGTGAAGGTGGTGACTTACTTCAACCGGACTGTACGAAGGCACTG

110 115 120 125 130 135
Ser Arg Phe Phe Tyr Tyr Leu Asp Arg Tyr Phe Ile Ala Arg Arg Ser Leu Pro Pro Leu Asn Glu Val Gly Leu Thr Cys Phe Arg Asp
AtCUL1

CTGGTTTATAATGAGCTACATTCTAAGGTCAAACAAGCTGTAATAGCTCTTGTTGATAAAGAACGGGAGGGCGAGCAGATTGATAGGGCC 2790
GACCAAATATTACTCGATGTAAGATTCCAGTTTGTTCGACATTATCGAGAACAACTATTTCTTGCCCTCCCGCTCGTCTAACTATCCCGG

140 145 150 155 160 165
Leu Val Tyr Asn Glu Leu His Ser Lys Val Lys Gln Ala Val Ile Ala Leu Val Asp Lys Glu Arg Glu Gly Glu Gln Ile Asp Arg Ala
AtCUL1

CTGCTGAAAAACGTATTAGATATCTATGTAGAGATTGGAATGGGGCAGATGGAGAGGTATGAAGAAGATTTTGAAAGCTTCATGCTTCAA 2880
GACGACTTTTTGCATAATCTATAGATACATCTCTAACCTTACCCCGTCTACCTCTCCATACTTCTTCTAAAACTTTCGAAGTACGAAGTT

170 175 180 185 190 195
Leu Leu Lys Asn Val Leu Asp Ile Tyr Val Glu Ile Gly Met Gly Gln Met Glu Arg Tyr Glu Glu Asp Phe Glu Ser Phe Met Leu Gln
AtCUL1

GATACTTCTTCGTATTATTCTCGCAAGGCATCAAGCTGGATTCAGGAAGATTCTTGCCCTGATTACATGTTGAAGTCTGAAGAATGTCTA 2970
CTATGAAGAAGCATAATAAGAGCGTTCCGTAGTTCGACCTAAGTCCTTCTAAGAACGGGACTAATGTACAACTTCAGACTTCTTACAGAT

200 205 210 215 220 225
Asp Thr Ser Ser Tyr Tyr Ser Arg Lys Ala Ser Ser Trp Ile Gln Glu Asp Ser Cys Pro Asp Tyr Met Leu Lys Ser Glu Glu Cys Leu
AtCUL1

AAGAAGGAGAGGGAGAGAGTGGCTCACTACCTACACTCAAGCAGTGAGCCAAAGCTGGTTGAGAAAGTACAACATGAATTGCTGGTTGTG 3060
TTCTTCCTCTCCCTCTCTCACCGAGTGATGGATGTGAGTTCGTCACTCGGTTTCGACCAACTCTTTCATGTTGTACTTAACGACCAACAC

230 235 240 245 250 255
Lys Lys Glu Arg Glu Arg Val Ala His Tyr Leu His Ser Ser Ser Glu Pro Lys Leu Val Glu Lys Val Gln His Glu Leu Leu Val Val
AtCUL1

TTTGCAAGTCAGCTTCTAGAAAAAGAACACTCAGGGTGCCGTGCATTGCTAAGAGATGACAAGGTGGATGATCTCTCCAGGATGTACAGG 3150
AAACGTTCAGTCGAAGATCTTTTTCTTGTGAGTCCCACGGCACGTAACGATTCTCTACTGTTCCACCTACTAGAGAGGTCCTACATGTCC

260 265 270 275 280 285
Phe Ala Ser Gln Leu Leu Glu Lys Glu His Ser Gly Cys Arg Ala Leu Leu Arg Asp Asp Lys Val Asp Asp Leu Ser Arg Met Tyr Arg
AtCUL1

CTTTACCATAAAATTTTGCGAGGCTTGGAACCTGTTGCAAACATCTTTAAGCAGCATGTCACAGCAGAGGGTAACGCTCTTGTCCAACAG 3240
GAAATGGTATTTTAAAACGCTCCGAACCTTGGACAACGTTTGTAGAAATTCGTCGTACAGTGTCGTCTCCCATTGCGAGAACAGGTTGTC

290 295 300 305 310 315
Leu Tyr His Lys Ile Leu Arg Gly Leu Glu Pro Val Ala Asn Ile Phe Lys Gln His Val Thr Ala Glu Gly Asn Ala Leu Val Gln Gln
AtCUL1

GCCGAAGACACGGCTACTAATCAGGTTGCAAATACTGCTAGCGTCCAGGAACAGGTTCTTATCAGAAAAGTGATTGAACTTCATGATAAA 3330
CGGCTTCTGTGCCGATGATTAGTCCAACGTTTATGACGATCGCAGGTCCTTGTCCAAGAATAGTCTTTTCACTAACTTGAAGTACTATTT

320 325 330 335 340 345
Ala Glu Asp Thr Ala Thr Asn Gln Val Ala Asn Thr Ala Ser Val Gln Glu Gln Val Leu Ile Arg Lys Val Ile Glu Leu His Asp Lys
AtCUL1

TACATGGTATATGTCACCGAGTGTTTCCAGAACCACACCCTCTTCCATAAGGCTTTGAAAGAGGCATTTGAGATTTTTTGTAACAAAACG 3420
ATGTACCATATACAGTGGCTCACAAAGGTCTTGGTGTGGGAGAAGGTATTCCGAAACTTTCTCCGTAAACTCTAAAAAACATTGTTTTGC

350 355 360 365 370 375
Tyr Met Val Tyr Val Thr Glu Cys Phe Gln Asn His Thr Leu Phe His Lys Ala Leu Lys Glu Ala Phe Glu Ile Phe Cys Asn Lys Thr
AtCUL1

GTTGCTGGAAGTTCAAGTGCAGAACTACTTGCAACATTTTGCGACAATATTCTCAAAAAGGGGGGAAGTGAAAAGCTGAGTGATGAAGCT 3510
CAACGACCTTCAAGTTCACGTCTTGATGAACGTTGTAAAACGCTGTTATAAGAGTTTTTCCCCCCTTCACTTTTCGACTCACTACTTCGA

380 385 390 395 400 405
Val Ala Gly Ser Ser Ser Ala Glu Leu Leu Ala Thr Phe Cys Asp Asn Ile Leu Lys Lys Gly Gly Ser Glu Lys Leu Ser Asp Glu Ala
AtCUL1

ATCGAAGATACGCTTGAGAAGGTTGTCAAATTGCTTGCATACATAAGTGACAAGGATCTTTTCGCTGAGTTCTACAGGAAGAAGCTGGCC 3600
TAGCTTCTATGCGAACTCTTCCAACAGTTTAACGAACGTATGTATTCACTGTTCCTAGAAAAGCGACTCAAGATGTCCTTCTTCGACCGG

410 415 420 425 430 435
Ile Glu Asp Thr Leu Glu Lys Val Val Lys Leu Leu Ala Tyr Ile Ser Asp Lys Asp Leu Phe Ala Glu Phe Tyr Arg Lys Lys Leu Ala
AtCUL1

CGTAGGCTCTTATTTGATCGCAGTGCTAATGATGATCATGAGAGAAGTATCCTGACAAAGCTCAAGCAACAATGTGGTGGACAGTTTACT 3690
GCATCCGAGAATAAACTAGCGTCACGATTACTACTAGTACTCTCTTCATAGGACTGTTTCGAGTTCGTTGTTACACCACCTGTCAAATGA

440 445 450 455 460 465
Arg Arg Leu Leu Phe Asp Arg Ser Ala Asn Asp Asp His Glu Arg Ser Ile Leu Thr Lys Leu Lys Gln Gln Cys Gly Gly Gln Phe Thr
AtCUL1

TCTAAGATGGAGGGCATGGTGACGGATTTGACACTGGCAAGAGAAAACCAAAACAGTTTCGAGGATTATCTAGGCAGTAACCCTGCTGCA 3780
AGATTCTACCTCCCGTACCACTGCCTAAACTGTGACCGTTCTCTTTTGGTTTTGTCAAAGCTCCTAATAGATCCGTCATTGGGACGACGT

470 475 480 485 490 495
Ser Lys Met Glu Gly Met Val Thr Asp Leu Thr Leu Ala Arg Glu Asn Gln Asn Ser Phe Glu Asp Tyr Leu Gly Ser Asn Pro Ala Ala
AtCUL1

AACCCAGGGATTGACTTGACCGTCACTGTTCTTACCACTGGTTTCTGGCCAAGTTACAAATCATTTGACATAAATCTACCCAGTGAAATG
TTGGGTCCCTAACTGAACTGGCAGTGACAAGAATGGTGACCAAAGACCGGTTCAATGTTTAGTAAACTGTATTTAGATGGGTCACTTTAC 3870

Asn Pro Gly Ile Asp Leu Thr Val Thr Val Leu Thr Thr Gly Phe Trp Pro Ser Tyr Lys Ser Phe Asp Ile Asn Leu Pro Ser Glu Met
AtCUL1

ATCAAGTGTGTTGAAGTCTTCAAAGGGTTTTATGAAACGAAAACGAAACACAGGAAGCTTACGTGGATCTATTCACTGGGAACTTGTCAC
TAGTTCACACAACTTCAGAAGTTTCCCAAAATACTTTGCTTTTGCTTTGTGTCCTTCGAATGCACCTAGATAAGTGACCCTTGAACAGTG 3960

Ile Lys Cys Val Glu Val Phe Lys Gly Phe Tyr Glu Thr Lys Thr Lys His Arg Lys Leu Thr Trp Ile Tyr Ser Leu Gly Thr Cys His
AtCUL1

ATAAACGGGAAGTTTGATCAAAAGGCCATCGAGTTAATAGTGTCTACTTACCAGGCTGCTGTGCTTCTACTCTTTAACACAACTGACAAG
TATTTGCCCTTCAAACTAGTTTTCCGGTAGCTCAATTATCACAGATGAATGGTCCGACGACACGAAGATGAGAAATTGTGTTGACTGTTC 4050

Ile Asn Gly Lys Phe Asp Gln Lys Ala Ile Glu Leu Ile Val Ser Thr Tyr Gln Ala Ala Val Leu Leu Leu Phe Asn Thr Thr Asp Lys
AtCUL1

TTAAGTTACACTGAGATCTTGGCTCAACTGAACCTAAGCCATGAAGATCTAGTTAGGTTGCTTCATTCCTTGTCATGTGCTAAGTACAAG
AATTCAATGTGACTCTAGAACCGAGTTGACTTGGATTCGGTACTTCTAGATCAATCCAACGAAGTAAGGAACAGTACACGATTCATGTTC 4140

Leu Ser Tyr Thr Glu Ile Leu Ala Gln Leu Asn Leu Ser His Glu Asp Leu Val Arg Leu Leu His Ser Leu Ser Cys Ala Lys Tyr Lys
AtCUL1

ATACTCCTTAAGGAGCCAAACACCAAGACTGTCTCCCAGAATGATGCCTTTGAGTTCAACTCCAAATTCACCGATAGAATGCGCAGAATC
TATGAGGAATTCCTCGGTTTGTGGTTCTGACAGAGGGTCTTACTACGGAAACTCAAGTTGAGGTTTAAGTGGCTATCTTACGCGTCTTAG 4230

Ile Leu Leu Lys Glu Pro Asn Thr Lys Thr Val Ser Gln Asn Asp Ala Phe Glu Phe Asn Ser Lys Phe Thr Asp Arg Met Arg Arg Ile
AtCUL1

AAGATCCCTCTTCCCCCAGTTGATGAAAGGAAGAAAGTCGTTGAAGATGTCGATAAAGACAGAAGATATGCAATTGATGCTGCCATTGTC
TTCTAGGGAGAAGGGGGTCAACTACTTTCCTTCTTTCAGCAACTTCTACAGCTATTTCTGTCTTCTATACGTTAACTACGACGGTAACAG 4320

Lys Ile Pro Leu Pro Pro Val Asp Glu Arg Lys Lys Val Val Glu Asp Val Asp Lys Asp Arg Arg Tyr Ala Ile Asp Ala Ala Ile Val
AtCUL1

AGGATCATGAAGAGCAGGAAAGTATTGGGACATCAACAACTTGTTTCTGAGTGTGTTGAGCAACTTAGCCGAATGTTCAAGCCTGATATC
TCCTAGTACTTCTCGTCCTTTCATAACCCTGTAGTTGTTGAACAAAGACTCACACAACTCGTTGAATCGGCTTACAAGTTCGGACTATAG 4410

Arg Ile Met Lys Ser Arg Lys Val Leu Gly His Gln Gln Leu Val Ser Glu Cys Val Glu Gln Leu Ser Arg Met Phe Lys Pro Asp Ile
AtCUL1

AAAGCGATCAAGAAGCGTATGGAGGATTTAATAACCAGAGATTATTTGGAGAGGGACAAGGAGAATCCTAACATGTTTAGGTACTTGGCT
TTTCGCTAGTTCTTCGCATACCTCCTAAATTATTGGTCTCTAATAAACCTCTCCCTGTTCCTCTTAGGATTGTACAAATCCATGAACCGA 4500

Lys Ala Ile Lys Lys Arg Met Glu Asp Leu Ile Thr Arg Asp Tyr Leu Glu Arg Asp Lys Glu Asn Pro Asn Met Phe Arg Tyr Leu Ala
AtCUL1

GATTGTACAAATCCATGAACCGA
CUL1 R

KpnI
ggtacccctcgagtctggtaaagaaaccgctgctgcgaaatttgaacgccagcacatggactcgtctactagcgcagcttaattaacctag
ccatgggagctcagaccatttctttggcgacgacgctttaaacttgcggtcgtgtacctgagcagatgatcgcgtcgaattaattggatc 4590

Gly Thr Leu Glu Ser Gly Lys Glu Thr Ala Ala Ala Lys Phe Glu Arg Gln His Met Asp Ser Ser Thr Ser Ala Ala *
(in frame with AtCUL1) S (in frame with S)

ccatgggagctcagac
CUL1 R

gctgctgccaccgctgagcaataactagcataaccccttggggcctctaaacgggtcttgaggggttttttgctgaaaggaggaactata
cgacgacggtggcgactcgttattgatcgtattggggaaccccggagatttgcccagaactccccaaaaaacgactttcctccttgatat 4680

T7 terminator

tccggattggcgaatgggacgcgccctgtagcggcgcattaagcgcggcgggtgtggtggttacgcgcagcgtgaccgctacacttgcca 4770
aggcctaaccgcttaccctgcgcgggacatcgccgcgtaattcgcgccgcccacaccaccaatgcgcgtcgcactggcgatgtgaacggt
f1 ori

gcgccctagcgcccgctcctttcgctttcttcccttcctttctcgccacgttcgccggctttccccgtcaagctctaaatcgggggctcc 4860
cgcgggatcgcgggcgaggaaagcgaaagaagggaaggaaagagcggtgcaagcggccgaaaggggcagttcgagatttagcccccgagg
f1 ori

ctttagggttccgatttagtgctttacggcacctcgaccccaaaaaacttgattagggtgatggttcacgtagtgggccatcgccctgat 4950
gaaatcccaaggctaaatcacgaaatgccgtggagctggggttttttgaactaatcccactaccaagtgcatcacccggtagcgggacta
f1 ori

agacggtttttcgccctttgacgttggagtccacgttctttaatagtggactcttgttccaaactggaacaacactcaaccctatctcgg 5040
tctgccaaaaagcgggaaactgcaacctcaggtgcaagaaattatcacctgagaacaaggtttgaccttgttgtgagttgggatagagcc
f1 ori

tctattcttttgatttataagggattttgccgatttcggcctattggttaaaaaatgagctgatttaacaaaaatttaacgcgaatttta 5130
agataagaaaactaaatattccctaaaacggctaaagccggataaccaattttttactcgactaaattgtttttaaattgcgcttaaaat
f1 ori

acaaaatattaacgtttacaatttctggcggcacgatggcatgagattatcaaaaaggatcttcacctagatccttttaaattaaaaatg 5220
tgttttataattgcaaatgttaaagaccgccgtgctaccgtactctaatagtttttcctagaagtggatctaggaaaatttaatttttac
f1 ori

aagttttaaatcaatctaaagtatatatgagtaaacttggtctgacagttaccaatgcttaatcagtgaggcacctatctcagcgatctg 5310
ttcaaaatttagttagatttcatatatactcatttgaaccagactgtcaatggttacgaattagtcactccgtggatagagtcgctagac
285 280 275
* Trp His Lys Ile Leu Ser Ala Gly Ile Glu Ala Ile Gln
AmpR

tctatttcgttcatccatagttgcctgactccccgtcgtgtagataactacgatacgggagggcttaccatctggccccagtgctgcaat 5400
agataaagcaagtaggtatcaacggactgaggggcagcacatctattgatgctatgccctcccgaatggtagaccggggtcacgacgtta
270 265 260 255 250 245
Arg Asn Arg Glu Asp Met Thr Ala Gln Ser Gly Thr Thr Tyr Ile Val Val Ile Arg Ser Pro Lys Gly Asp Pro Gly Leu Ala Ala Ile
AmpR

gataccgcgagacccacgctcaccggctccagatttatcagcaataaaccagccagccggaagggccgagcgcagaagtggtcctgcaac 5490
ctatggcgctctgggtgcgagtggccgaggtctaaatagtcgttatttggtcggtcggccttcccggctcgcgtcttcaccaggacgttg
240 235 230 225 220 215
Ile Gly Arg Ser Gly Arg Glu Gly Ala Gly Ser Lys Asp Ala Ile Phe Trp Gly Ala Pro Leu Ala Ser Arg Leu Leu Pro Gly Ala Val
AmpR

tttatccgcctccatccagtctattaattgttgccgggaagctagagtaagtagttcgccagttaatagtttgcgcaacgttgttgccat 5580
aaataggcggaggtaggtcagataattaacaacggcccttcgatctcattcatcaagcggtcaattatcaaacgcgttgcaacaacggta
210 205 200 195 190 185
Lys Asp Ala Glu Met Trp Asp Ile Leu Gln Gln Arg Ser Ala Leu Thr Leu Leu Glu Gly Thr Leu Leu Lys Arg Leu Thr Thr Ala Met
AmpR

tgctacaggcatcgtggtgtcacgctcgtcgtttggtatggcttcattcagctccggttcccaacgatcaaggcgagttacatgatcccc 5670
acgatgtccgtagcaccacagtgcgagcagcaaaccataccgaagtaagtcgaggccaagggttgctagttccgctcaatgtactagggg
180 175 170 165 160 155
Ala Val Pro Met Thr Thr Asp Arg Glu Asp Asn Pro Ile Ala Glu Asn Leu Glu Pro Glu Trp Arg Asp Leu Arg Thr Val His Asp Gly
AmpR

catgttgtgcaaaaaagcggttagctccttcggtcctccgatcgttgtcagaagtaagttggccgcagtgttatcactcatggttatggc 5760
gtacaacacgttttttcgccaatcgaggaagccaggaggctagcaacagtcttcattcaaccggcgtcacaatagtgagtaccaataccg
150 145 140 135 130 125
Met Asn His Leu Phe Ala Thr Leu Glu Lys Pro Gly Gly Ile Thr Thr Leu Leu Leu Asn Ala Ala Thr Asn Asp Ser Met Thr Ile Ala
AmpR

agcactgcataattctcttactgtcatgccatccgtaagatgcttttctgtgactggtgagtactcaaccaagtcattctgagaatagtg 5850
tcgtgacgtattaagagaatgacagtacggtaggcattctacgaaaagacactgaccactcatgagttggttcagtaagactcttatcac
120 115 110 105 100 95
Ala Ser Cys Leu Glu Arg Val Thr Met Gly Asp Thr Leu His Lys Glu Thr Val Pro Ser Tyr Glu Val Leu Asp Asn Gln Ser Tyr His
AmpR

tatgcggcgaccgagttgctcttgcccggcgtcaatacgggataataccgcgccacatagcagaactttaaaagtgctcatcattggaaa 5940
atacgccgctggctcaacgagaacgggccgcagttatgccctattatggcgcggtgtatcgtcttgaaattttcacgagtagtaaccttt
90 85 80 75 70 65
Ile Arg Arg Gly Leu Gln Glu Gln Gly Ala Asp Ile Arg Ser Leu Val Ala Gly Cys Leu Leu Val Lys Phe Thr Ser Met Met Pro Phe
AmpR

acgttcttcggggcgaaaactctcaaggatcttaccgctgttgagatccagttcgatgtaacccactcgtgcacccaactgatcttcagc 6030
tgcaagaagccccgcttttgagagttcctagaatggcgacaactctaggtcaagctacattgggtgagcacgtgggttgactagaagtcg
60 55 50 45 40 35
Arg Glu Glu Pro Arg Phe Ser Glu Leu Ile Lys Gly Ser Asn Leu Asp Leu Glu Ile Tyr Gly Val Arg Ala Gly Leu Gln Asp Glu Ala
AmpR

atcttttactttcaccagcgtttctgggtgagcaaaaacaggaaggcaaaatgccgcaaaaaagggaataagggcgacacggaaatgttg 6120
tagaaaatgaaagtggtcgcaaagacccactcgtttttgtccttccgttttacggcgttttttcccttattcccgctgtgcctttacaac
30 25 20 15 10 5
Asp Lys Val Lys Val Leu Thr Glu Pro His Ala Phe Val Pro Leu Cys Phe Ala Ala Phe Phe Pro Ile Leu Ala Val Arg Phe His Gln
signal sequence
AmpR

aatactcatactcttcctttttcaatcatgattgaagcatttatcagggttattgtctcatgagcggatacatatttgaatgtatttaga 6210
ttatgagtatgagaaggaaaaagttagtactaacttcgtaaatagtcccaataacagagtactcgcctatgtataaacttacataaatct
1
Ile Ser Met
signal sequ...
AmpR
AmpR promoter

aaaataaacaaataggtcatgaccaaaatcccttaacgtgagttttcgttccactgagcgtcagaccccgtagaaaagatcaaaggatct 6300
ttttatttgtttatccagtactggttttagggaattgcactcaaaagcaaggtgactcgcagtctggggcatcttttctagtttcctaga
AmpR promoter

tcttgagatcctttttttctgcgcgtaatctgctgcttgcaaacaaaaaaaccaccgctaccagcggtggtttgtttgccggatcaagag 6390
agaactctaggaaaaaaagacgcgcattagacgacgaacgtttgttttttttggtggcgatggtcgccaccaaacaaacggcctagttctc
ori

ctaccaactctttttccgaaggtaactggcttcagcagagcgcagataccaaatactgtccttctagtgtagccgtagttaggccaccac 6480
gatggttgagaaaaaggcttccattgaccgaagtcgtctcgcgtctatggtttatgacaggaagatcacatcggcatcaatccggtggtg
ori

ttcaagaactctgtagcaccgcctacatacctcgctctgctaatcctgttaccagtggctgctgccagtggcgataagtcgtgtcttacc 6570
aagttcttgagacatcgtggcggatgtatggagcgagacgattaggacaatggtcaccgacgacggtcaccgctattcagcacagaatgg
ori

gggttggactcaagacgatagttaccggataaggcgcagcggtcgggctgaacggggggttcgtgcacacagcccagcttggagcgaacg 6660
cccaacctgagttctgctatcaatggcctattccgcgtcgccagcccgacttgccccccaagcacgtgtgtcgggtcgaacctcgcttgc
ori

```
acctacaccgaactgagatacctacagcgtgagctatgagaaagcgccacgcttcccgaagggagaaaggcggacaggtatccggtaagc
tggatgtggcttgactctatggatgtcgcactcgatactctttcgcggtgcgaagggcttccctctttccgcctgtccataggccattcg   6750
ori

ggcagggtcggaacaggagagcgcacgagggagcttccagggggaaacgcctggtatctttatagtcctgtcgggtttcgccacctctga
ccgtcccagccttgtcctctcgcgtgctccctcgaaggtccccctttgcggaccatagaaatatcaggacagcccaaagcggtggagact   6840
ori

cttgagcgtcgatttttgtgatgctcgtcaggggggcggagcctatggaaaaacgccagcaacgcggcctttttacggttcctggcctttt
gaactcgcagctaaaaacactacgagcagtccccccgcctcggatacctttttgcggtcgttgcgccggaaaaatgccaaggaccggaaaa   6930
ori

tgctggccttttgctcacatgttctttcctgcgttatcccctgattctgtggataaccgtattaccgcctttgagtgagctgataccgct
acgaccggaaaacgagtgtacaagaaaggacgcaataggggactaagacacctattggcataatggcggaaactcactcgactatggcga   7020

cgccgcagccgaacgaccgagcgcagcgagtcagtgagcgaggaagcggaagagcgcctgatgcggtattttctccttacgcatctgtgc
gcggcgtcggcttgctggctcgcgtcgctcagtcactcgctccttcgccttctcgcggactacgccataaaagaggaatgcgtagacacg   7110
bom

ggtatttcacaccgcatatatggtgcactctcagtacaatctgctctgatgccgcatagttaagccagtatacactccgctatcgctacg
ccataaagtgtggcgtatataccacgtgagagtcatgttagacgagactacggcgtatcaattcggtcatatgtgaggcgatagcgatgc   7200
bom

tgactgggtcatggctgcgccccgacacccgccaacacccgctgacgcgccctgacgggcttgtctgctcccggcatccgcttacagaca
actgacccagtaccgacgcggggctgtgggcggttgtgggcgactgcgcgggactgcccgaacagacgagggccgtaggcgaatgtctgt   7290
bom

agctgtgaccgtctccgggagctgcatgtgtcagaggttttcaccgtcatcaccgaaacgcgcgaggcagctgcggtaaagctcatcagc
tcgacactggcagaggccctcgacgtacacagtctccaaaagtggcagtagtggctttgcgcgctccgtcgacgccatttcgagtagtcg   7380
              60          55          50          45
* Leu Asn Glu Gly Asp Asp Gly Phe Arg Ala Leu Cys Ser Arg Tyr Leu Glu Asp Ala
< rop

gtggtcgtgaagcgattcacagatgtctgcctgttcatccgcgtccagctcgttgagtttctccagaagcgttaatgtctggcttctgat
caccagcacttcgctaagtgtctacagacggacaagtaggcgcaggtcgagcaactcaaagaggtcttcgcaattacagaccgaagacta   7470
            40          35          30          25          20          15
His Asp His Leu Ser Glu Cys Ile Asp Ala Gln Glu Asp Ala Asp Leu Glu Asn Leu Lys Glu Leu Leu Thr Leu Thr Gln Ser Arg Ile
< rop

aaagcgggccatgttaagggcggttttttcctgtttggtcactgatgcctccgtgtaagggggatttctgttcatgggggtaatgatacc
tttcgcccggtacaattcccgccaaaaaaggacaaaccagtgactacggaggcacattccccctaaagacaagtacccccattactatgg   7560
            10          5       1
Phe Arg Ala Met Asn Leu Ala Thr Lys Glu Gln Lys Thr Met
< rop

gatgaaacgagagaggatgctcacgatacgggttactgatgatgaacatgcccggttactggaacgttgtgagggtaaacaactggcggt
ctactttgctctctcctacgagtgctatgcccaatgactactacttgtacgggccaatgaccttgcaacactcccatttgttgaccgcca   7650

atggatgcggcgggaccagagaaaaatcactcagggtcaatgccagcgcttcgttaatacagatgtaggtgttccacagggtagccagca
tacctacgccgccctggtctctttttagtgagtcccagttacggtcgcgaagcaattatgtctacatccacaaggtgtcccatcggtcgt   7740

gcatcctgcgatgcagatccggaacataatggtgcagggcgctgacttccgcgtttccagactttacgaaacacggaaaccgaagaccat
cgtaggacgctacgtctaggccttgtattaccacgtcccgcgactgaaggcgcaaaggtctgaaatgctttgtgcctttggcttctggta   7830

tcatgttgttgctcaggtcgcagacgttttgcagcagcagtcgcttcacgttcgctcgcgtatcggtgattcattctgctaaccagtaag
agtacaacaacgagtccagcgtctgcaaaacgtcgtcgtcagcgaagtgcaagcgagcgcatagccactaagtaagacgattggtcattc   7920
```

gcaaccccgccagcctagccgggtcctcaacgacaggagcacgatcatgctagtcatgccccgcgcccaccggaaggagctgactgggtt 8010
cgttggggcggtcggatcggcccaggagttgctgtcctcgtgctagtacgatcagtacggggcgcgggtggccttcctcgactgacccaa

gaaggctctcaagggcatcggtcgagatcccggtgcctaatgagtgagctaacttacattaattgcgttgcgctcactgcccgctttcca 8100
cttccgagagttcccgtagccagctctagggccacggattactcactcgattgaatgtaattaacgcaacgcgagtgacgggcgaaaggt
360
* Gln Gly Ser Glu Leu
lacI

gtcgggaaacctgtcgtgccagctgcattaatgaatcggccaacgcgcggggagaggcggtttgcgtattgggcgccagggtggtttttc 8190
cagccctttggacagcacggtcgacgtaattacttagccggttgcgcgcccctctccgccaaacgcataacccgcggtcccaccaaaaag
355 350 345 340 335 330
Arg Ser Val Gln Arg Ala Leu Gln Met Leu Ser Asp Ala Leu Ala Arg Pro Ser Ala Thr Gln Thr Asn Pro Ala Leu Thr Thr Lys Arg
lacI

ttttcaccagtgagacgggcaacagctgattgcccttcaccgcctggccctgagagagttgcagcaagcggtccacgctggtttgcccca 8280
aaaagtggtcactctgcccgttgtcgactaacgggaagtggcggaccgggactctctcaacgtcgttcgccaggtgcgaccaaacggggt
325 320 315 310 305 300
Lys Val Leu Ser Val Pro Leu Leu Gln Asn Gly Lys Val Ala Gln Gly Gln Ser Leu Gln Leu Leu Arg Asp Val Ser Thr Gln Gly Leu
lacI

gcaggcgaaaatcctgtttgatggtggttaacggcgggatataacatgagctgtcttcggtatcgtcgtatcccactaccgagatgtccg 8370
cgtccgcttttaggacaaactaccaccaattgccgccctatattgtactcgacagaagccatagcagcatagggtgatggctctacaggc
295 290 285 280 275 270
Leu Arg Phe Asp Gln Lys Ile Thr Thr Leu Pro Pro Ile Tyr Cys Ser Ser Asp Glu Thr Asp Asp Tyr Gly Val Val Ser Ile Asp Ala
lacI

caccaacgcgcagcccggactcggtaatggcgcgcattgcgcccagcgccatctgatcgttggcaaccagcatcgcagtgggaacgatgc 8460
gtggttgcgcgtcgggcctgagccattaccgcgcgtaacgcgggtcgcggtagactagcaaccgttggtcgtagcgtcacccttgctacg
265 260 255 250 245 240
Gly Val Arg Leu Gly Ser Glu Thr Ile Ala Arg Met Ala Gly Leu Ala Met Gln Asp Asn Ala Val Leu Met Ala Thr Pro Val Ile Gly
lacI

cctcattcagcatttgcatggtttgttgaaaaccggacatggcactccagtcgccttcccgttccgctatcggctgaatttgattgcgag 8550
ggagtaagtcgtaaacgtaccaaacaacttttggcctgtaccgtgaggtcagcggaagggcaaggcgatagccgacttaaactaacgctc
235 230 225 220 215 210
Glu Asn Leu Met Gln Met Thr Gln Gln Phe Gly Ser Met Ala Ser Trp Asp Gly Glu Arg Glu Ala Ile Pro Gln Ile Gln Asn Arg Thr
lacI

tgagatatttatgccagccagccagacgcagacgcgccgagacagaacttaatgggcccgctaacagcgcgatttgctggtgacccaatg 8640
actctataaatacggtcggtcggtctgcgtctgcgcggctctgtcttgaattacccgggcgattgtcgcgctaaacgaccactgggttac
205 200 195 190 185 180
Leu Tyr Lys His Trp Gly Ala Leu Arg Leu Arg Ala Ser Val Ser Ser Leu Pro Gly Ala Leu Leu Ala Ile Gln Gln His Gly Leu Ala
lacI

cgaccagatgctccacgcccagtcgcgtaccgtcttcatgggagaaaataatactgttgatgggtgtctggtcagagacatcaagaaata 8730
gctggtctacgaggtgcgggtcagcgcatggcagaagtaccctcttttattatgacaactacccacagaccagtctctgtagttctttat
175 170 165 160 155 150
Val Leu His Glu Val Gly Leu Arg Thr Gly Asp Glu His Ser Phe Ile Ile Ser Asn Ile Pro Thr Gln Asp Ser Val Asp Leu Phe Leu
lacI

acgccggaacattagtgcaggcagcttccacagcaatggcatcctggtcatccagcggatagttaatgatcagcccactgacgcgttgcg 8820
tgcggccttgtaatcacgtccgtcgaaggtgtcgttaccgtaggaccagtaggtcgcctatcaattactagtcgggtgactgcgcaacgc
145 140 135 130 125 120
Ala Pro Val Asn Thr Cys Ala Ala Glu Val Ala Ile Ala Asp Gln Asp Asp Leu Pro Tyr Asn Ile Ile Leu Gly Ser Val Arg Gln Ala
lacI

cgagaagattgtgcaccgccgctttacaggcttcgacgccgcttcgttctaccatcgacaccaccacgctggcacccagttgatcggcgc 8910
gctcttctaacacgtggcggcgaaatgtccgaagctgcggcgaagcaagatggtagctgtggtggtgcgaccgtgggtcaactagccgcg
115 110 105 100 95 90
Leu Leu Asn His Val Ala Ala Lys Cys Ala Glu Val Gly Ser Arg Glu Val Met Ser Val Val Val Ser Ala Gly Leu Gln Asp Ala Arg
lacI

```
gagatttaatcgccgcgacaatttgcgacggcgcgtgcagggccagactggaggtggcaacgccaatcagcaacgactgtttgcccgcca   9000
ctctaaattagcggcgctgttaaacgctgccgcgcacgtcccggtctgacctccaccgttgcggttagtcgttgctgacaaacgggcggt
85 Ser Lys Ile Ala Ala 80 Val Ile Gln Ser Pro 75 Ala His Leu Ala Leu 70 Ser Ser Thr Ala Val 65 Gly Ile Leu Leu Ser 60 Gln Lys Gly Ala Leu
< lacI

gttgttgtgccacgcggttgggaatgtaattcagctccgccatcgccgcttccacttttcccgcgttttcgcagaaacgtggctggcct   9090
caacaacacggtgcgccaacccttacattaagtcgaggcggtagcggcgaaggtgaaaagggcgcaaaagcgtctttgcaccgaccgga
55 Gln Gln Ala Val Arg 50 Asn Pro Ile Tyr Asn 45 Leu Glu Ala Met Ala 40 Ala Glu Val Lys Glu 35 Arg Thr Lys Ala Ser 30 Val His Ser Ala Gln
< lacI

ggttcaccacgcgggaaacggtctgataagagacaccggcatactctgcgacatcgtataacgttactggtttcacattcaccaccctga   9180
ccaagtggtgcgccctttgccagactattctctgtggccgtatgagacgctgtagcatattgcaatgaccaaagtgtaagtggtgggact
25 Asn Val Val Arg Ser 20 Val Thr Gln Tyr Ser 15 Val Gly Ala Tyr Glu 10 Ala Val Asp Tyr Leu 5 Thr Val Pro Lys 1 Met
< lacI   < lacI promoter

attgactctcttccgggcgctatcatgccataccgcgaaaggttttgcgccattcgatggtgtc   3'  ...  9244
taactgagagaaggcccgcgatagtacggtatggcgctttccaaaacgcggtaagctaccacag   5'
< lacI promoter
```

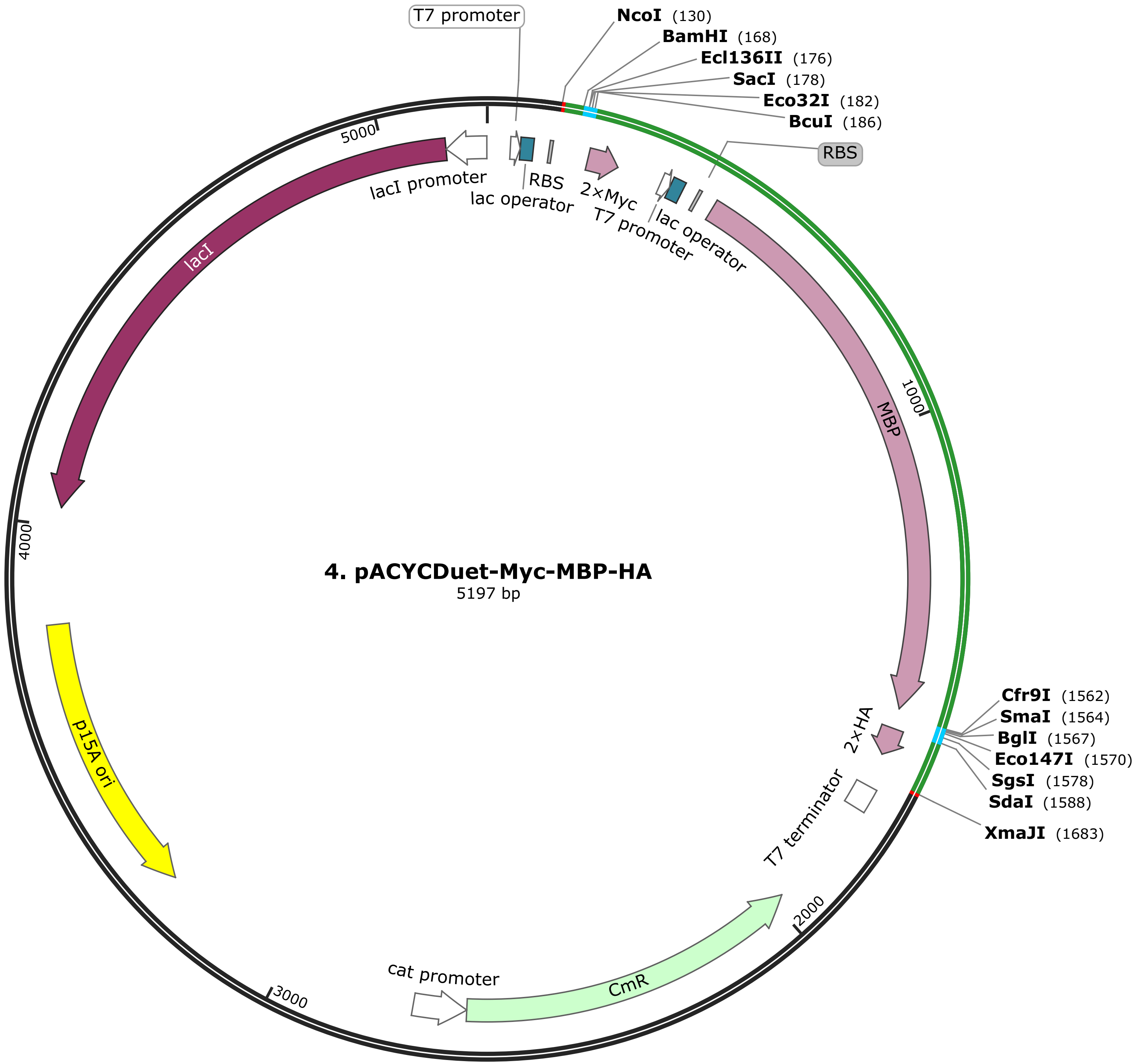

Created with SnapGene®
4. pACYCDuet-Myc-MBP-HA
5197 bp
T7 promoter
NcoI (130)
BamHI (168)
Ecl136II (176)
SacI (178)
Eco32I (182)
BcuI (186)
RBS
lacI promoter
lac operator
RBS
2×Myc
T7 promoter
lac operator
lacI
MBP
Cfr9I (1562)
SmaI (1564)
BglI (1567)
Eco147I (1570)
SgsI (1578)
SdaI (1588)
XmaJI (1683)
2×HA
T7 terminator
p15A ori
cat promoter
CmR
5000
4000
3000
2000
1000

5′ gggatctcgacgctctcccttatgcgactcctgcattaggaaattaatacgactcactataggggaattgtgagcggataacaattcccc 90
3′ ccctagagctgcgagagggaatacgctgaggacgtaatcctttaattatgctgagtgatatccccttaacactcgcctattgttaagggg

T7 promoter | lac operator

NcoI | **BamHI** | **Ecl136II** | **SacI**

tgtagaaataattttgtttaactttaatAAGGAGatataccatgggcagcagcgcatcaccatcatcaccaagccaggGatccGAGCTCG 180
acatctttattaaaacaaattgaaattaTTCCTCtatatggtacccgtcgtcgcgtagtggtagtagtggttcggtccCtaggCTCGAGC

RBS

Eco32I | **BcuI**

ATATCACTAGTCCTGAGCAGAAGCTCATCTCCGAGGAGGACCTCGAGCAGAAGCTCATCTCCGAGGAGGACCTCCTGActgcaggtcgac 270
TATAGTGATCAGGACTCGTCTTCGAGTAGAGGCTCCTCCTGGAGCTCGTCTTCGAGTAGAGGCTCCTCCTGGAGGACTgacgtccagctg

1 Glu Gln Lys Leu 5 Ile Ser Glu Glu Asp 10 Leu Glu Gln Lys Leu 15 Ile Ser Glu Glu Asp 20 Leu Leu Thr Ala Gly Arg
2×Myc

aagcttgcggccgcataatgcttaagtcgaacagaaagtaatcgtattgtacacggccgcataatcgaaattaatacgactcactatagg 360
ttcgaacgccggcgtattacgaattcagcttgtctttcattagcataacatgtgccggcgtattagctttaattatgctgagtgatatcc

Gln Ala Cys Gly Arg Ile Met Leu Lys Ser Asn Arg Lys
(in frame with 2×Myc)
T7 promoter

ggaattgtgagcggataacaattccccatcttagtatattagttaagtataagAAGGAGatatacatatggcagatctcaattgggaatt 450
ccttaacactcgcctattgttaaggggtagaatcatataatcaattcatattcTTCCTCtatatgtataccgtctagagttaacccttaa

lac operator | RBS

catgaaaatcgaagaaggtaaactggtaatctggattaacggcgataaaggctataacggtctcgctgaagtcggtaagaaattcgagaa 540
gtactttagcttcttccatttgaccattagacctaattgccgctatttccgatattgccagagcgacttcagccattctttaagctctt

1 Met Lys Ile Glu 5 Glu Gly Lys Leu Val 10 Ile Trp Ile Asn Gly 15 Asp Lys Gly Tyr Asn 20 Gly Leu Ala Glu Val 25 Gly Lys Lys Phe Glu 30 Lys
MBP

agataccggaattaaagtcaccgttgagcatccggataaactggaagagaaattcccacaggttgcggcaactggcgatggccctgacat 630
tctatggccttaatttcagtggcaactcgtaggcctatttgaccttctctttaagggtgtccaacgccgttgaccgctaccgggactgta

Asp Thr Gly Ile 35 Lys Val Thr Val Glu 40 His Pro Asp Lys Leu 45 Glu Glu Lys Phe Pro 50 Gln Val Ala Ala Thr 55 Gly Asp Gly Pro Asp 60 Ile
MBP

tatcttctgggcacacgaccgctttggtggctacgctcaatctggcctgttggctgaaatcaccccggacaaagcgttccaggacaagct 720
atagaagacccgtgtgctggcgaaaccaccgatgcgagttagaccggacaaccgactttagtggggcctgtttcgcaaggtcctgttcga

Ile Phe Trp Ala 65 His Asp Arg Phe Gly 70 Gly Tyr Ala Gln Ser 75 Gly Leu Leu Ala Glu 80 Ile Thr Pro Asp Lys 85 Ala Phe Gln Asp Lys 90 Leu
MBP

gtatccgtttacctgggatgccgtacgttacaacggcaagctgattgcttacccgatcgctgttgaagcgttatcgctgatttataacaa 810
cataggcaaatggaccctacggcatgcaatgttgccgttcgactaacgaatgggctagcgacaacttcgcaatagcgactaaatattgtt

Tyr Pro Phe Thr 95 Trp Asp Ala Val Arg 100 Tyr Asn Gly Lys Leu 105 Ile Ala Tyr Pro Ile 110 Ala Val Glu Ala Leu 115 Ser Leu Ile Tyr Asn 120 Lys
MBP

agatctgctgccgaacccgccaaaaacctgggaagagatcccggcgctggataaagaactgaaagcgaaaggtaagagcgcgctgatgtt 900
tctagacgacggcttgggcggtttttggacccttctctagggccgcgacctatttcttgactttcgctttccattctcgcgcgactacaa

Asp Leu Leu Pro 125 Asn Pro Pro Lys Thr 130 Trp Glu Glu Ile Pro 135 Ala Leu Asp Lys Glu 140 Leu Lys Ala Lys Gly 145 Lys Ser Ala Leu Met 150 Phe
MBP

caacctgcaagaaccgtacttcacctggccgctgattgctgctgacggggttatgcgttcaagtatgaaaacggcaagtacgacattaa 990
gttggacgttcttggcatgaagtggaccggcgactaacgacgactgccccaatacgcaagttcatacttttgccgttcatgctgtaatt

Asn Leu Gln Glu 155 Pro Tyr Phe Thr Trp 160 Pro Leu Ile Ala Ala 165 Asp Gly Gly Tyr Ala 170 Phe Lys Tyr Glu Asn 175 Gly Lys Tyr Asp Ile 180 Lys
MBP

agacgtgggcgtggataacgctggcgcgaaagcgggtctgaccttcctggttgacctgattaaaaacaaacacatgaatgcagacaccga 1080
tctgcacccgcacctattgcgaccgcgctttcgcccagactggaaggaccaactggactaatttttgtttgtgtacttacgtctgtggct
185 190 195 200 205 210
Asp Val Gly Val Asp Asn Ala Gly Ala Lys Ala Gly Leu Thr Phe Leu Val Asp Leu Ile Lys Asn Lys His Met Asn Ala Asp Thr Asp
MBP

ttactccatcgcagaagctgcctttaataaaggcgaaacagcgatgaccatcaacggcccgtgggcatggtccaacatcgacaccagcaa 1170
aatgaggtagcgtcttcgacggaaattatttccgctttgtcgctactggtagttgccgggcacccgtaccaggttgtagctgtggtcgtt
215 220 225 230 235 240
Tyr Ser Ile Ala Glu Ala Ala Phe Asn Lys Gly Glu Thr Ala Met Thr Ile Asn Gly Pro Trp Ala Trp Ser Asn Ile Asp Thr Ser Lys
MBP

agtgaattatggtgtaacggtactgccgaccttcaagggtcaaccatccaaaccgttcgttggcgtgctgagcgcaggtattaacgccgc 1260
tcacttaataccacattgccatgacggctggaagttcccagttggtaggtttggcaagcaaccgcacgactcgcgtccataattgcggcg
245 250 255 260 265 270
Val Asn Tyr Gly Val Thr Val Leu Pro Thr Phe Lys Gly Gln Pro Ser Lys Pro Phe Val Gly Val Leu Ser Ala Gly Ile Asn Ala Ala
MBP

cagtccgaacaaagagctggcaaaagagttcctcgaaaactatctgctgactgatgaaggtctggaagcggttaataaagacaaaccgct 1350
gtcaggcttgtttctcgaccgttttctcaaggagcttttgatagacgactgactacttccagaccttcgccaattatttctgtttggcga
275 280 285 290 295 300
Ser Pro Asn Lys Glu Leu Ala Lys Glu Phe Leu Glu Asn Tyr Leu Leu Thr Asp Glu Gly Leu Glu Ala Val Asn Lys Asp Lys Pro Leu
MBP

gggtgccgtagcgctgaagtcttacgaggaagagttggcgaaagatccacgtattgccgccactatggaaaacgcccagaaaggtgaaat 1440
cccacggcatcgcgacttcagaatgctccttctcaaccgctttctaggtgcataacggcggtgataccttttgcgggtctttccacttta
305 310 315 320 325 330
Gly Ala Val Ala Leu Lys Ser Tyr Glu Glu Glu Leu Ala Lys Asp Pro Arg Ile Ala Ala Thr Met Glu Asn Ala Gln Lys Gly Glu Ile
MBP

catgccgaacatcccgcagatgtccgctttctggtatgccgtgcgtactgcggtgatcaacgccgccagcggtcgtcagactgtcgatga 1530
gtacggcttgtagggcgtctacaggcgaaagaccatacggcacgcatgacgccactagttgcggcggtcgccagcagtctgacagctact
335 340 345 350 355 360
Met Pro Asn Ile Pro Gln Met Ser Ala Phe Trp Tyr Ala Val Arg Thr Ala Val Ile Asn Ala Ala Ser Gly Arg Gln Thr Val Asp Glu
MBP

BglI
SmaI Eco147I
Cfr9I SgsI SdaI
agccctgaaagacgcgcagactgaattcgagCCCGGGAGGCCTctcggcgcgcctgcaggtTACCCATACGACGTACCAGATTACGCTTA 1620
tcgggactttctgcgcgtctgacttaagctcGGGCCCTCCGGAgagccgcgcggacgtccaATGGGTATGCTGCATGGTCTAATGCGAAT
365 1 5 10
Ala Leu Lys Asp Ala Gln Thr Glu Phe Glu Pro Gly Arg Pro Leu Gly Ala Pro Ala Gly Tyr Pro Tyr Asp Val Pro Asp Tyr Ala Tyr
MBP (in frame with MBP) 2×HA

XmaJI
CCCATACGACGTACCAGATTACGCTcgacaagcttgcggccgcataatgcttaagtaattaacctaggctgctgccaccgctgagcaata 1710
GGGTATGCTGCATGGTCTAATGCGAgctgttcgaacgccggcgtattacgaattcattaattggatccgacgacggtggcgactcgttat
15
Pro Tyr Asp Val Pro Asp Tyr Ala Arg Gln Ala Cys Gly Arg Ile Met Leu Lys
2×HA (in frame with 2×HA)

actagcataaccccttggggcctctaaacgggtcttgaggggttttttgctgaaacctcaggcatttgagaagcacacggtcacactgct 1800
tgatcgtattggggaaccccggagatttgcccagaactccccaaaaaacgactttggagtccgtaaactcttcgtgtgccagtgtgacga
T7 terminator

tccggtagtcaataaaccggtaaaccagcaatagacataagcggctatttaacgaccctgccctgaaccgacgaccgggtcgaatttgct 1890
aggccatcagttatttggccatttggtcgttatctgtattcgccgataaattgctgggacgggacttggctgctggcccagcttaaacga

ttcgaatttctgccattcatccgcttattatcacttattcaggcgtagcaccaggcgtttaagggcaccaataactgccttaaaaaaatt 1980
aagcttaaagacggtaagtaggcgaataatagtgaataagtccgcatcgtggtccgcaaattcccgtggttattgacggaatttttttaa

220
*
CmR

acgccccgccctgccactcatcgcagtactgttgtaattcattaagcattctgccgacatggaagccatcacagacggcatgatgaacct 2070
tgcggggcgggacggtgagtagcgtcatgacaacattaagtaattcgtaagacggctgtaccttcggtagtgtctgccgtactacttgga

215 210 205 200 195 190
Ala Gly Gly Gln Trp Glu Asp Cys Tyr Gln Gln Leu Glu Asn Leu Met Arg Gly Val His Phe Gly Asp Cys Val Ala His His Val Gln
CmR

gaatcgccagcggcatcagcaccttgtcgccttgcgtataatatttgcccatagtgaaaacgggggcgaagaagttgtccatattggcca 2160
cttagcggtcgccgtagtcgtggaacagcggaacgcatattataaacgggtatcacttttgcccccgcttcttcaacaggtataaccggt

185 180 175 170 165 160
Ile Ala Leu Pro Met Leu Val Lys Asp Gly Gln Thr Tyr Tyr Lys Gly Met Thr Phe Val Pro Ala Phe Phe Asn Asp Met Asn Ala Val
CmR

cgtttaaatcaaaactggtgaaactcacccagggattggctgagacgaaaaacatattctcaataaaccctttagggaaataggccaggt 2250
gcaaatttagttttgaccactttgagtgggtccctaaccgactctgctttttgtataagagttatttgggaaatccctttatccggtcca

155 150 145 140 135 130
Asn Leu Asp Phe Ser Thr Phe Ser Val Trp Pro Asn Ala Ser Val Phe Phe Met Asn Glu Ile Phe Gly Lys Pro Phe Tyr Ala Leu Asn
CmR

tttcaccgtaacacgccacatcttgcgaatatatgtgtagaaactgccggaaatcgtcgtggtattcactccagagcgatgaaaacgttt 2340
aaagtggcattgtgcggtgtagaacgcttatatacacatctttgacggcctttagcagcaccataagtgaggtctcgctacttttgcaaa

125 120 115 110 105 100
Glu Gly Tyr Cys Ala Val Asp Gln Ser Tyr Ile His Leu Phe Gln Arg Phe Asp Asp His Tyr Glu Ser Trp Leu Ser Ser Phe Thr Glu
CmR

cagtttgctcatggaaaacggtgtaacaagggtgaacactatcccatatcaccagctcaccgtctttcattgccatacggaactccggat 2430
gtcaaacgagtaccttttgccacattgttcccacttgtgatagggtatagtggtcgagtggcagaaagtaacggtatgccttgaggccta

95 90 85 80 75 70
Thr Gln Glu His Phe Val Thr Tyr Cys Pro His Val Ser Asp Trp Ile Val Leu Glu Gly Asp Lys Met Ala Met Arg Phe Glu Pro His
CmR

gagcattcatcaggcgggcaagaatgtgaataaaggccggataaaacttgtgcttatttttctttacggtctttaaaaaggccgtaatat 2520
ctcgtaagtagtccgcccgttcttacacttatttccggcctattttgaacacgaataaaaagaaatgccagaaatttttccggcattata

65 60 55 50 45 40
Ala Asn Met Leu Arg Ala Leu Ile His Ile Phe Ala Pro Tyr Phe Lys His Lys Asn Lys Lys Val Thr Lys Leu Phe Ala Thr Ile Asp
CmR

ccagctgaacggtctggttataggtacattgagcaactgactgaaatgcctcaaaatgttctttacgatgccattgggatatatcaacgg 2610
ggtcgacttgccagaccaatatccatgtaactcgttgactgactttacggagttttacaagaaatgctacggtaaccctatatagttgcc

35 30 25 20 15 10
Leu Gln Val Thr Gln Asn Tyr Thr Cys Gln Ala Val Ser Gln Phe Ala Glu Phe His Glu Lys Arg His Trp Gln Ser Ile Asp Val Thr
CmR

tggtatatccagtgatttttttctccattttagcttccttagctcctgaaaatctcgataactcaaaaaatacgcccggtagtgatctta 2700
accatataggtcactaaaaaaagaggtaaaatcgaaggaatcgaggacttttagagctattgagttttttatgcgggccatcactagaat

5 1
Thr Tyr Gly Thr Ile Lys Lys Glu Met
CmR
cat promoter

tttcattatggtgaaagttggaacctcttacgtgccgatcaacgtctcatttttcgccaaaagttggcccagggcttcccggtatcaacag 2790
aaagtaataccactttcaaccttggagaatgcacggctagttgcagagtaaaaagcggttttcaaccgggtcccgaagggccatagttgtc

cat promoter

ggacaccaggatttatttattctgcgaagtgatcttccgtcacaggtatttattcggcgcaaagtgcgtcgggtgatgctgccaacttac 2880
cctgtggtcctaaataaataagacgcttcactagaaggcagtgtccataaataagccgcgtttcacgcagcccactacgacggttgaatg

tgatttagtgtatgatggtgtttttgaggtgctccagtggcttctgtttctatcagctgtccctcctgttcagctactgacggggtggtg 2970
actaaatcacatactaccacaaaaactccacgaggtcaccgaagacaaagatagtcgacagggaggacaagtcgatgactgccccaccac

cgtaacggcaaaagcaccgccggacatcagcgctagcggagtgtatactggcttactatgttggcactgatgagggtgtcagtgaagtgc 3060
gcattgccgttttcgtggcggcctgtagtcgcgatcgcctcacatatgaccgaatgatacaaccgtgactactcccacagtcacttcacg

ttcatgtggcaggagaaaaaaggctgcaccggtgcgtcagcagaatatgtgatacaggatatattccgcttcctcgctcactgactcgct 3150
aagtacaccgtcctctttttttccgacgtggccacgcagtcgtcttatacactatgtcctatataaggcgaaggagcgagtgactgagcga

acgctcggtcgttcgactgcggcgagcggaaatggcttacgaacggggcggagatttcctggaagatgccaggaagatacttaacaggga 3240
tgcgagccagcaagctgacgccgctcgcctttaccgaatgcttgccccgcctctaaaggaccttctacggtccttctatgaattgtccct

agtgagagggccgcggcaaagccgtttttccataggctccgcccccctgacaagcatcacgaaatctgacgctcaaatcagtggtggcga 3330
tcactctcccggcgccgtttcggcaaaaaggtatccgaggcggggggactgttcgtagtgctttagactgcgagtttagtcaccaccgct
p15A ori

aacccgacaggactataaagataccaggcgtttccccctggcggctccctcgtgcgctctcctgttcctgcctttcggtttaccggtgtca 3420
ttgggctgtcctgatatttctatggtccgcaaagggggaccgccgagggagcacgcgagaggacaaggacggaaagccaaatggccacagt
p15A ori

ttccgctgttatggccgcgtttgtctcattccacgcctgacactcagttccgggtaggcagttcgctccaagctggactgtatgcacgaa 3510
aaggcgacaataccggcgcaaacagagtaaggtgcggactgtgagtcaaggcccatccgtcaagcgaggttcgacctgacatacgtgctt
p15A ori

ccccccgttcagtccgaccgctgcgccttatccggtaactatcgtcttgagtccaacccggaaagacatgcaaaagcaccactggcagca 3600
ggggggcaagtcaggctggcgacgcggaataggccattgatagcagaactcaggttgggcctttctgtacgttttcgtggtgaccgtcgt
p15A ori

gccactggtaattgatttagaggagttagtcttgaagtcatgcgccggttaaggctaaactgaaaggacaagttttggtgactgcgctcc 3690
cggtgaccattaactaaatctcctcaatcagaacttcagtacgcggccaattccgatttgactttcctgttcaaaaccactgacgcgagg
p15A ori

tccaagccagttacctcggttcaaagagttggtagctcagagaaccttcgaaaaaccgccctgcaaggcggttttttcgttttcagagca 3780
aggttcggtcaatggagccaagtttctcaaccatcgagtctcttggaagctttttggcgggacgttccgccaaaaaagcaaaagtctcgt
p15A ori

agagattacgcgcagaccaaaacgatctcaagaagatcatcttattaatcagataaaatatttctagatttcagtgcaatttatctcttc 3870
tctctaatgcgcgtctggttttgctagagttcttctagtagaataattagtctattttataaagatctaaagtcacgttaaatagagaag
p15A ori

aaatgtagcacctgaagtcagccccatacgatataagttgtaattctcatgttagtcatgccccgcgcccaccggaaggagctgactggg 3960
tttacatcgtggacttcagtcggggtatgctatattcaacattaagagtacaatcagtacggggcgcgggtggccttcctcgactgaccc

ttgaaggctctcaagggcatcggtcgagatcccggtgcctaatgagtgagctaacttacattaattgcgttgcgctcactgcccgctttc 4050
aacttccgagagttcccgtagccagctctagggccacggattactcactcgattgaatgtaattaacgcaacgcgagtgacgggcgaaag
360
* Gln Gly Ser Glu
lacI

cagtcgggaaacctgtcgtgccagctgcattaatgaatcggccaacgcgcggggagaggcggtttgcgtattgggcgccagggtggtttt 4140
gtcagccctttggacagcacggtcgacgtaattacttagccggttgcgcgcccctctccgccaaacgcataacccgcggtcccaccaaaa
355 350 345 340 335 330
Leu Arg Ser Val Gln Arg Ala Leu Gln Met Leu Ser Asp Ala Leu Ala Arg Pro Ser Ala Thr Gln Thr Asn Pro Ala Leu Thr Thr Lys
lacI

tcttttcaccagtgagacgggcaacagctgattgcccttcaccgcctggccctgagagagttgcagcaagcggtccacgctggtttgccc 4230
agaaaagtggtcactctgcccgttgtcgactaacgggaagtggcggaccgggactctctcaacgtcgttcgccaggtgcgaccaaacggg
325 320 315 310 305 300
Arg Lys Val Leu Ser Val Pro Leu Leu Gln Asn Gly Lys Val Ala Gln Gly Gln Ser Leu Gln Leu Leu Arg Asp Val Ser Thr Gln Gly
lacI

cagcaggcgaaaatcctgtttgatggtggttaacggcgggatataacatgagctgtcttcggtatcgtcgtatcccactaccgagatgtc 4320
gtcgtccgcttttaggacaaactaccaccaattgccgccctatattgtactcgacagaagccatagcagcatagggtgatggctctacag
295 290 285 280 275 270
Leu Leu Arg Phe Asp Gln Lys Ile Thr Thr Leu Pro Pro Ile Tyr Cys Ser Ser Asp Glu Thr Asp Asp Tyr Gly Val Val Ser Ile Asp
lacI

cgcaccaacgcgcagcccggactcggtaatggcgcgcattgcgcccagcgccatctgatcgttggcaaccagcatcgcagtgggaacgat 4410
gcgtggttgcgcgtcgggcctgagccattaccgcgcgtaacgcgggtcgcggtagactagcaaccgttggtcgtagcgtcacccttgcta
265 260 255 250 245 240
Ala Gly Val Arg Leu Gly Ser Glu Thr Ile Ala Arg Met Ala Gly Leu Ala Met Gln Asp Asn Ala Val Leu Met Ala Thr Pro Val Ile
lacI

gccctcattcagcatttgcatggtttgttgaaaaccggacatggcactccagtcgccttcccgttccgctatcggctgaatttgattgcg 4500
cgggagtaagtcgtaaacgtaccaaacaacttttggcctgtaccgtgaggtcagcggaagggcaaggcgatagccgacttaaactaacgc
235 230 225 220 215 210
Gly Glu Asn Leu Met Gln Met Thr Gln Gln Phe Gly Ser Met Ala Ser Trp Asp Gly Glu Arg Glu Ala Ile Pro Gln Ile Gln Asn Arg
lacI

agtgagatatttatgccagccagccagacgcagacgcgccgagacagaacttaatgggcccgctaacagcgcgatttgctggtgacccaa 4590
tcactctataaatacggtcggtcggtctgcgtctgcgcggctctgtcttgaattacccgggcgattgtcgcgctaaacgaccactgggtt
205 200 195 190 185 180
Thr Leu Tyr Lys His Trp Gly Ala Leu Arg Leu Arg Ala Ser Val Ser Ser Leu Pro Gly Ala Leu Leu Ala Ile Gln Gln His Gly Leu
lacI

tgcgaccagatgctccacgcccagtcgcgtaccgtcttcatgggagaaaataatactgttgatgggtgtctggtcagagacatcaagaaa 4680
acgctggtctacgaggtgcgggtcagcgcatggcagaagtaccctcttttattatgacaactacccacagaccagtctctgtagttcttt
175 170 165 160 155 150
Ala Val Leu His Glu Val Gly Leu Arg Thr Gly Asp Glu His Ser Phe Ile Ile Ser Asn Ile Pro Thr Gln Asp Ser Val Asp Leu Phe
lacI

taacgccggaacattagtgcaggcagcttccacagcaatggcatcctggtcatccagcggatagttaatgatcagcccactgacgcgttg 4770
attgcggccttgtaatcacgtccgtcgaaggtgtcgttaccgtaggaccagtaggtcgcctatcaattactagtcgggtgactgcgcaac
145 140 135 130 125 120
Leu Ala Pro Val Asn Thr Cys Ala Ala Glu Val Ala Ile Ala Asp Gln Asp Asp Leu Pro Tyr Asn Ile Ile Leu Gly Ser Val Arg Gln
lacI

cgcgagaagattgtgcaccgccgctttacaggcttcgacgccgcttcgttctaccatcgacaccaccacgctggcacccagttgatcggc 4860
gcgctcttctaacacgtggcggcgaaatgtccgaagctgcggcgaagcaagatggtagctgtggtggtgcgaccgtgggtcaactagccg
115 110 105 100 95 90
Ala Leu Leu Asn His Val Ala Ala Lys Cys Ala Glu Val Gly Ser Arg Glu Val Met Ser Val Val Val Ser Ala Gly Leu Gln Asp Ala
lacI

gcgagatttaatcgccgcgacaatttgcgacggcgcgtgcagggccagactggaggtggcaacgccaatcagcaacgactgtttgcccgc 4950
cgctctaaattagcggcgctgttaaacgctgccgcgcacgtcccggtctgacctccaccgttgcggttagtcgttgctgacaaacgggcg
85 80 75 70 65 60
Arg Ser Lys Ile Ala Ala Val Ile Gln Ser Pro Ala His Leu Ala Leu Ser Ser Thr Ala Val Gly Ile Leu Leu Ser Gln Lys Gly Ala
lacI

cagttgttgtgccacgcggttgggaatgtaattcagctccgccatcgccgcttccactttttcccgcgttttcgcagaaacgtggctggc 5040
gtcaacaacacggtgcgccaacccttacattaagtcgaggcggtagcggcgaaggtgaaaaagggcgcaaaagcgtctttgcaccgaccg
55 50 45 40 35 30
Leu Gln Gln Ala Val Arg Asn Pro Ile Tyr Asn Leu Glu Ala Met Ala Ala Glu Val Lys Glu Arg Thr Lys Ala Ser Val His Ser Ala
lacI

ctggttcaccacgcgggaaacggtctgataagagacaccggcatactctgcgacatcgtataacgttactggtttcacattcaccaccct 5130
gaccaagtggtgcgccctttgccagactattctctgtggccgtatgagacgctgtagcatattgcaatgaccaaagtgtaagtggtggga
25 20 15 10 5 1
Gln Asn Val Val Arg Ser Val Thr Gln Tyr Ser Val Gly Ala Tyr Glu Ala Val Asp Tyr Leu Thr Val Pro Lys Met
lacI
lacI promoter

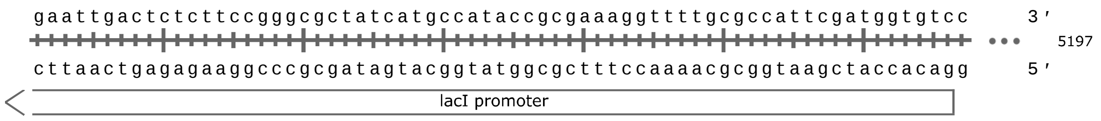

gaattgactctcttccgggcgctatcatgccataccgcgaaaggttttgcgccattcgatggtgtcc 3′
5197
cttaactgagagaaggcccgcgatagtacggtatggcgctttccaaaacgcggtaagctaccacagg 5′
lacI promoter

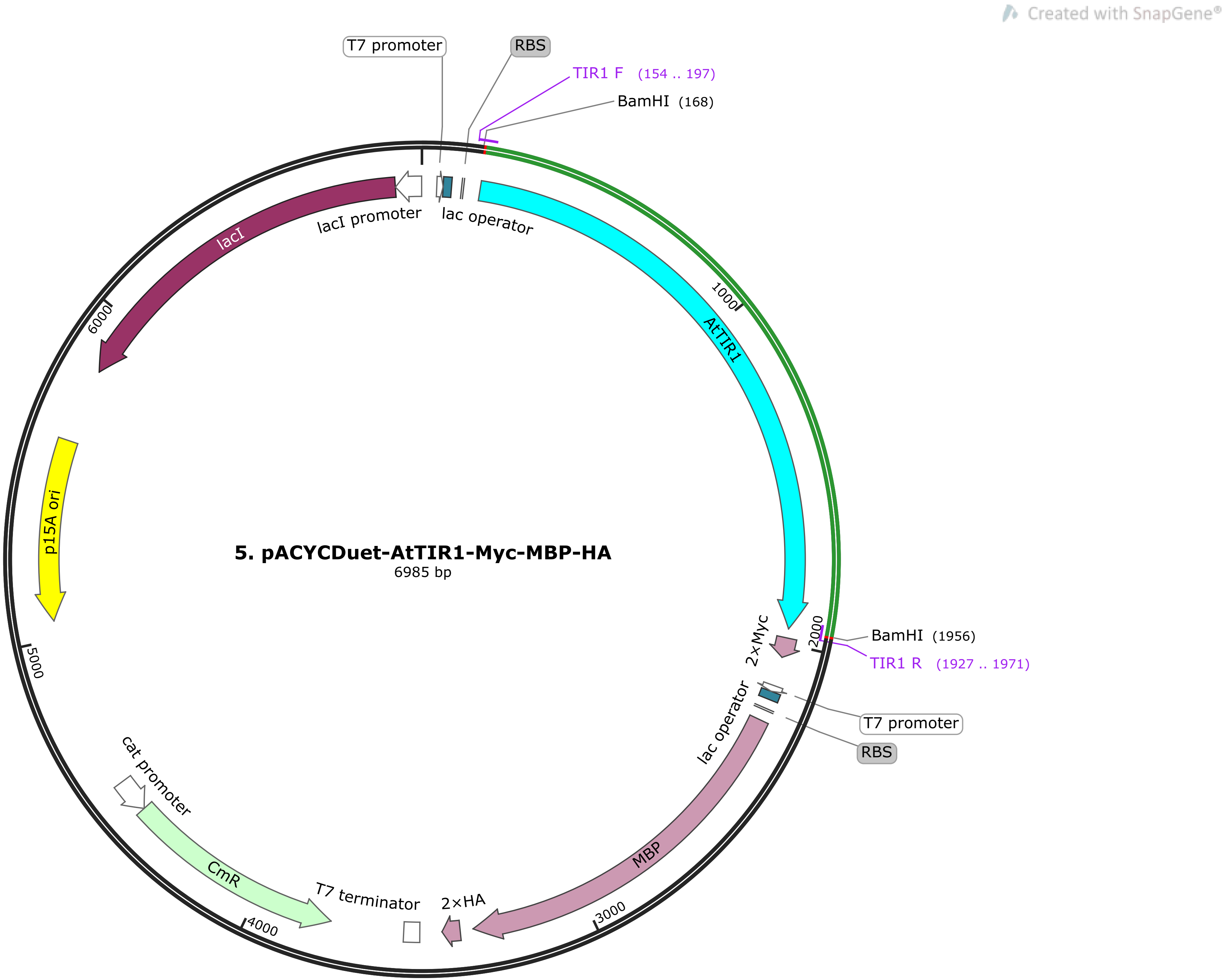
Created with SnapGene®
T7 promoter
RBS
TIR1 F (154 .. 197)
BamHI (168)
lacI promoter
lac operator
lacI
AtTIR1
1000
6000
p15A ori
5. pACYCDuet-AtTIR1-Myc-MBP-HA
6985 bp
2×Myc
2000
BamHI (1956)
TIR1 R (1927 .. 1971)
5000
lac operator
T7 promoter
RBS
cat promoter
MBP
CmR
T7 terminator
2×HA
3000
4000

Sequence: 5. pACYCDuet-AtTIR1-Myc-MBP-HA.dna (Circular / 6985 bp)
Enzymes: < Unsaved Enzyme Set > (1 of 678 total)
Unique Cutters **Bold**
Features: 18 total
Primers: 5 total

5′ gggatctcgacgctctcccttatgcgactcctgcattaggaaattaatacgactcactataggggaattgtgagcggataacaattcccc 90
3′ ccctagagctgcgagagggaatacgctgaggacgtaatcctttaattatgctgagtgatatccccttaacactcgcctattgttaagggg

T7 promoter
lac operator

BamHI
TIR1 F
catcaccaagccaggGatccATGCAGA

tgtagaaataattttgtttaactttaataaggagatataccatgggcagcagcGcatcaccatcatcaccaagccagggatccATGCAGA 180
acatctttattaaaacaaattgaaattattcctctatatggtacccgtcgtcgCgtagtggtagtagtggttcggtccctaggTACGTCT

1
Met Gln
RBS
AtTIR1

TIR1 F
AGCGAATAGCCTTGTCG

AGCGAATAGCCTTGTCGTTTCCAGAAGAGGTACTAGAGCATGTGTTCTCGTTTATTCAGCTGGATAAGGATAGGAACTCAGTCTCTCTGG 270
TCGCTTATCGGAACAGCAAAGGTCTTCTCCATGATCTCGTACACAAGAGCAAATAAGTCGACCTATTCCTATCCTTGAGTCAGAGAGACC

5 10 15 20 25 30
Lys Arg Ile Ala Leu Ser Phe Pro Glu Glu Val Leu Glu His Val Phe Ser Phe Ile Gln Leu Asp Lys Asp Arg Asn Ser Val Ser Leu
AtTIR1

TGTGCAAGTCATGGTACGAGATCGAGCGGTGGTGCAGGAGGAAAGTCTTCATCGGGAACTGCTACGCCGTGAGTCCAGCGACGGTGATTA 360
ACACGTTCAGTACCATGCTCTAGCTCGCCACCACGTCCTCCTTTCAGAAGTAGCCCTTGACGATGCGGCACTCAGGTCGCTGCCACTAAT

35 40 45 50 55 60
Val Cys Lys Ser Trp Tyr Glu Ile Glu Arg Trp Cys Arg Arg Lys Val Phe Ile Gly Asn Cys Tyr Ala Val Ser Pro Ala Thr Val Ile
AtTIR1

GGAGGTTCCCGAAAGTGAGATCCGTGGAGCTTAAAGGAAAACCTCACTTTGCTGACTTTAATTTGGTACCTGACGGATGGGGAGGTTACG 450
CCTCCAAGGGCTTTCACTCTAGGCACCTCGAATTTCCTTTTGGAGTGAAACGACTGAAATTAAACCATGGACTGCCTACCCCTCCAATGC

65 70 75 80 85 90
Arg Arg Phe Pro Lys Val Arg Ser Val Glu Leu Lys Gly Lys Pro His Phe Ala Asp Phe Asn Leu Val Pro Asp Gly Trp Gly Gly Tyr
AtTIR1

TGTATCCATGGATTGAGGCCATGTCTTCGTCTTACACGTGGCTTGAAGAGATAAGGCTGAAGAGGATGGTGGTCACCGACGATTGCTTGG 540
ACATAGGTACCTAACTCCGGTACAGAAGCAGAATGTGCACCGAACTTCTCTATTCCGACTTCTCCTACCACCAGTGGCTGCTAACGAACC

95 100 105 110 115 120
Val Tyr Pro Trp Ile Glu Ala Met Ser Ser Ser Tyr Thr Trp Leu Glu Glu Ile Arg Leu Lys Arg Met Val Val Thr Asp Asp Cys Leu
AtTIR1

AGCTCATAGCCAAGTCTTTTAAGAATTTTAAGGTTCTTGTGCTTTCTTCCTGCGAAGGCTTCTCCACCGATGGTCTCGCTGCTATCGCTG 630
TCGAGTATCGGTTCAGAAAATTCTTAAAATTCCAAGAACACGAAAGAAGGACGCTTCCGAAGAGGTGGCTACCAGAGCGACGATAGCGAC

125 130 135 140 145 150
Glu Leu Ile Ala Lys Ser Phe Lys Asn Phe Lys Val Leu Val Leu Ser Ser Cys Glu Gly Phe Ser Thr Asp Gly Leu Ala Ala Ile Ala
AtTIR1

CCACTTGCAGGAATCTGAAAGAGCTTGACTTACGAGAGAGTGATGTTGACGACGTTAGTGGCCACTGGCTTAGCCATTTCCCAGATACAT 720
GGTGAACGTCCTTAGACTTTCTCGAACTGAATGCTCTCTCACTACAACTGCTGCAATCACCGGTGACCGAATCGGTAAAGGGTCTATGTA

155 160 165 170 175 180
Ala Thr Cys Arg Asn Leu Lys Glu Leu Asp Leu Arg Glu Ser Asp Val Asp Asp Val Ser Gly His Trp Leu Ser His Phe Pro Asp Thr
AtTIR1

ACACTTCTTTGGTATCACTCAATATATCTTGCTTAGCATCTGAGGTCAGTTTCTCTGCTCTGGAAAGGCTGGTGACTAGGTGTCCCAATC 810
TGTGAAGAAACCATAGTGAGTTATATAGAACGAATCGTAGACTCCAGTCAAAGAGACGAGACCTTTCCGACCACTGATCCACAGGGTTAG

185 190 195 200 205 210
Tyr Thr Ser Leu Val Ser Leu Asn Ile Ser Cys Leu Ala Ser Glu Val Ser Phe Ser Ala Leu Glu Arg Leu Val Thr Arg Cys Pro Asn
AtTIR1

TCAAGTCTCTCAAGCTTAACCGAGCTGTTCCACTTGAAAAATTGGCTACTTTACTTCAAAGAGCACCTCAATTGGAGGAATTGGGCACTG 900
AGTTCAGAGAGTTCGAATTGGCTCGACAAGGTGAACTTTTTAACCGATGAAATGAAGTTTCTCGTGGAGTTAACCTCCTTAACCCGTGAC

215 220 225 230 235 240
Leu Lys Ser Leu Lys Leu Asn Arg Ala Val Pro Leu Glu Lys Leu Ala Thr Leu Leu Gln Arg Ala Pro Gln Leu Glu Glu Leu Gly Thr
AtTIR1

GTGGGTACACTGCAGAAGTGCGACCAGATGTTTACTCTGGTTTATCTGTAGCGCTCTCTGGGTGCAAGGAATTGAGGTGCTTATCTGGAT 990
CACCCATGTGACGTCTTCACGCTGGTCTACAAATGAGACCAAATAGACATCGCGAGAGACCCACGTTCCTTAACTCCACGAATAGACCTA
245 250 255 260 265 270
Gly Gly Tyr Thr Ala Glu Val Arg Pro Asp Val Tyr Ser Gly Leu Ser Val Ala Leu Ser Gly Cys Lys Glu Leu Arg Cys Leu Ser Gly
AtTIR1

TTTGGGATGCTGTTCCTGCCTATCTTCCAGCAGTTTATTCGGTTTGCAGTCGGCTTACAACTTTGAATCTGAGTTATGCAACAGTCCAGA 1080
AAACCCTACGACAAGGACGGATAGAAGGTCGTCAAATAAGCCAAACGTCAGCCGAATGTTGAAACTTAGACTCAATACGTTGTCAGGTCT
275 280 285 290 295 300
Phe Trp Asp Ala Val Pro Ala Tyr Leu Pro Ala Val Tyr Ser Val Cys Ser Arg Leu Thr Thr Leu Asn Leu Ser Tyr Ala Thr Val Gln
AtTIR1

GCTATGATCTTGTCAAGCTTCTTTGTCAATGCCCTAAACTGCAGCGCCTCTGGGTGCTTGACTACATCGAGGATGCTGGTCTTGAGGTGC 1170
CGATACTAGAACAGTTCGAAGAAACAGTTACGGGATTTGACGTCGCGGAGACCCACGAACTGATGTAGCTCCTACGACCAGAACTCCACG
305 310 315 320 325 330
Ser Tyr Asp Leu Val Lys Leu Leu Cys Gln Cys Pro Lys Leu Gln Arg Leu Trp Val Leu Asp Tyr Ile Glu Asp Ala Gly Leu Glu Val
AtTIR1

TTGCTTCAACCTGCAAGGACCTACGCGAGCTGAGAGTGTTTCCGTCCGAGCCTTTTGTCATGGAACCAAATGTGGCATTGACGGAACAGG 1260
AACGAAGTTGGACGTTCCTGGATGCGCTCGACTCTCACAAAGGCAGGCTCGGAAAACAGTACCTTGGTTTACACCGTAACTGCCTTGTCC
335 340 345 350 355 360
Leu Ala Ser Thr Cys Lys Asp Leu Arg Glu Leu Arg Val Phe Pro Ser Glu Pro Phe Val Met Glu Pro Asn Val Ala Leu Thr Glu Gln
AtTIR1

GGCTTGTCTCCGTCTCCATGGGCTGTCCAAAACTCGAGTCGGTTCTCTACTTCTGCCGTCAAATGACCAATGCTGCATTGATAACCATTG 1350
CCGAACAGAGGCAGAGGTACCCGACAGGTTTTGAGCTCAGCCAAGAGATGAAGACGGCAGTTTACTGGTTACGACGTAACTATTGGTAAC
365 370 375 380 385 390
Gly Leu Val Ser Val Ser Met Gly Cys Pro Lys Leu Glu Ser Val Leu Tyr Phe Cys Arg Gln Met Thr Asn Ala Ala Leu Ile Thr Ile
AtTIR1

CTAGGAACCGTCCCAACATGACTCGCTTCCGTTTGTGCATCATTGAGCCAAAAGCCCCAGACTATCTGACTCTAGAGCCACTGGATATTG 1440
GATCCTTGGCAGGGTTGTACTGAGCGAAGGCAAACACGTAGTAACTCGGTTTTCGGGGTCTGATAGACTGAGATCTCGGTGACCTATAAC
395 400 405 410 415 420
Ala Arg Asn Arg Pro Asn Met Thr Arg Phe Arg Leu Cys Ile Ile Glu Pro Lys Ala Pro Asp Tyr Leu Thr Leu Glu Pro Leu Asp Ile
AtTIR1

GATTTGGAGCCATAGTAGAGCACTGCAAGGATCTCCGTCGCCTCTCTCTATCTGGCCTCTTGACCGACAAGGTTTTTGAATACATTGGGA 1530
CTAAACCTCGGTATCATCTCGTGACGTTCCTAGAGGCAGCGGAGAGAGATAGACCGGAGAACTGGCTGTTCCAAAAACTTATGTAACCCT
425 430 435 440 445 450
Gly Phe Gly Ala Ile Val Glu His Cys Lys Asp Leu Arg Arg Leu Ser Leu Ser Gly Leu Leu Thr Asp Lys Val Phe Glu Tyr Ile Gly
AtTIR1

CATATGCCAAGAAGATGGAAATGCTCTCAGTGGCATTTGCAGGAGACAGTGACTTAGGCATGCATCATGTTTTGTCCGGGTGCGATAGCT 1620
GTATACGGTTCTTCTACCTTTACGAGAGTCACCGTAAACGTCCTCTGTCACTGAATCCGTACGTAGTACAAAACAGGCCCACGCTATCGA
455 460 465 470 475 480
Thr Tyr Ala Lys Lys Met Glu Met Leu Ser Val Ala Phe Ala Gly Asp Ser Asp Leu Gly Met His His Val Leu Ser Gly Cys Asp Ser
AtTIR1

TGAGGAAACTAGAGATAAGGGACTGCCCGTTTGGAGACAAGGCGCTTTTGGCCAATGCTTCAAAGCTGGAGACAATGCGATCCCTTTGGA 1710
ACTCCTTTGATCTCTATTCCCTGACGGGCAAACCTCTGTTCCGCGAAAACCGGTTACGAAGTTTCGACCTCTGTTACGCTAGGGAAACCT
485 490 495 500 505 510
Leu Arg Lys Leu Glu Ile Arg Asp Cys Pro Phe Gly Asp Lys Ala Leu Leu Ala Asn Ala Ser Lys Leu Glu Thr Met Arg Ser Leu Trp
AtTIR1

TGTCTTCTTGTTCCGTGAGTTTTGGAGCCTGCAAGTTACTAGGACAGAAGATGCCAAAGCTGAATGTGGAAGTCATCGATGAACGGGGTG 1800
ACAGAAGAACAAGGCACTCAAAACCTCGGACGTTCAATGATCCTGTCTTCTACGGTTTCGACTTACACCTTCAGTAGCTACTTGCCCCAC
515 520 525 530 535 540
Met Ser Ser Cys Ser Val Ser Phe Gly Ala Cys Lys Leu Leu Gly Gln Lys Met Pro Lys Leu Asn Val Glu Val Ile Asp Glu Arg Gly
AtTIR1

```
CACCGGACTCGAGACCAGAGAGCTGCCCTGTTGAGAGAGTCTTCATATACCGAACAGTGGCTGGTCCTCGATTTGACATGCCTGGCTTCG   1890
GTGGCCTGAGCTCTGGTCTCTCGACGGGACAACTCTCTCAGAAGTATATGGCTTGTCACCGACCAGGAGCTAAACTGTACGGACCGAAGC
      545            550            555            560            565            570
Ala Pro Asp Ser Arg Pro Glu Ser Cys Pro Val Glu Arg Val Phe Ile Tyr Arg Thr Val Ala Gly Pro Arg Phe Asp Met Pro Gly Phe
AtTIR1

                                                                        BamHI
TCTGGAACATGGACCAAGACTCAACAATGAGGTTTTCCAGGCAAATCATTACTACTAACGGATTAGGATCCGAGCTCGATATCACTAGTC   1980
AGACCTTGTACCTGGTTCTGAGTTGTTACTCCAAAAGGTCCGTTTAGTAATGATGATTGCCTAATCCTAGGCTCGAGCTATAGTGATCAG
      575            580            585            590
Val Trp Asn Met Asp Gln Asp Ser Thr Met Arg Phe Ser Arg Gln Ile Ile Thr Thr Asn Gly Leu Gly Ser Glu Leu Asp Ile Thr Ser
AtTIR1                                                                     (in frame with AtTIR1)
GGTCCGTTTAGTAATGATGATTGCCTAATCCTAGGCTCGAGCTAT
TIR1 R

CTGAGCAGAAGCTCATCTCCGAGGAGGACCTCGAGCAGAAGCTCATCTCCGAGGAGGACCTCCTGActgcaggtcgacaagcttgcggcc   2070
GACTCGTCTTCGAGTAGAGGCTCCTCCTGGAGCTCGTCTTCGAGTAGAGGCTCCTCCTGGAGGACTgacgtccagctgttcgaacgccgg
     1           5            10             15             20
Pro Glu Gln Lys Leu Ile Ser Glu Glu Asp Leu Glu Gln Lys Leu Ile Ser Glu Glu Asp Leu Leu Thr Ala Gly Arg Gln Ala Cys Gly
2×Myc                                                                      (in frame with 2×Myc)

gcataatgcttaagtcgaacagaaagtaatcgtattgtacacggccgcataatcgaaattaatacgactcactataggggaattgtgagc   2160
cgtattacgaattcagcttgtctttcattagcataacatgtgccggcgtattagctttaattatgctgagtgatatccccttaacactcg
Arg Ile Met Leu Lys Ser Asn Arg Lys *
(in frame with 2×Myc)                                                 T7 promoter   lac operator

ggataacaattccccatcttagtatattagttaagtataagaaggagatatacatatggcagatctcaattgggaattcatgaaaatcga   2250
cctattgttaaggggtagaatcatataatcaattcatattcttcctctatatgtataccgtctagagttaacccttaagtactttttagct
                                                                                 1
                                                                                 Met Lys Ile Glu
lac operator                              RBS                                    MBP

agaaggtaaactggtaatctggattaacggcgataaaggctataacggtctcgctgaagtcggtaagaaattcgagaaagataccggaat   2340
tcttccatttgaccattagacctaattgccgctatttccgatattgccagagcgacttcagccattctttaagctctttctatggcctta
 5            10             15             20             25             30
Glu Gly Lys Leu Val Ile Trp Ile Asn Gly Asp Lys Gly Tyr Asn Gly Leu Ala Glu Val Gly Lys Lys Phe Glu Lys Asp Thr Gly Ile
MBP

taaagtcaccgttgagcatccggataaactggaagagaaattcccacaggttgcggcaactggcgatggccctgacattatcttctgggc   2430
atttcagtggcaactcgtaggcctatttgaccttctctttaagggtgtccaacgccgttgaccgctaccgggactgtaatagaagacccg
 35            40             45             50             55             60
Lys Val Thr Val Glu His Pro Asp Lys Leu Glu Glu Lys Phe Pro Gln Val Ala Ala Thr Gly Asp Gly Pro Asp Ile Ile Phe Trp Ala
MBP

acacgaccgctttggtggctacgctcaatctggcctgttggctgaaatcaccccggacaaagcgttccaggacaagctgtatccgtttac   2520
tgtgctggcgaaaccaccgatgcgagttagaccggacaaccgactttagtggggcctgtttcgcaaggtcctgttcgacataggcaaatg
 65            70             75             80             85             90
His Asp Arg Phe Gly Gly Tyr Ala Gln Ser Gly Leu Leu Ala Glu Ile Thr Pro Asp Lys Ala Phe Gln Asp Lys Leu Tyr Pro Phe Thr
MBP

ctgggatgccgtacgttacaacggcaagctgattgcttacccgatcgctgttgaagcgttatcgctgatttataacaaagatctgctgcc   2610
gaccctacggcatgcaatgttgccgttcgactaacgaatgggctagcgacaacttcgcaatagcgactaaatattgtttctagacgacgg
 95            100            105            110            115            120
Trp Asp Ala Val Arg Tyr Asn Gly Lys Leu Ile Ala Tyr Pro Ile Ala Val Glu Ala Leu Ser Leu Ile Tyr Asn Lys Asp Leu Leu Pro
MBP

gaacccgccaaaaacctgggaagagatcccggcgctggataaagaactgaaagcgaaaggtaagagcgcgctgatgttcaacctgcaaga   2700
cttgggcggtttttggacccttctctagggccgcgacctatttcttgactttcgctttccattctcgcgcgactacaagttggacgttct
 125            130            135            140            145            150
Asn Pro Pro Lys Thr Trp Glu Glu Ile Pro Ala Leu Asp Lys Glu Leu Lys Ala Lys Gly Lys Ser Ala Leu Met Phe Asn Leu Gln Glu
MBP
```

accgtacttcacctggccgctgattgctgctgacgggggttatgcgttcaagtatgaaaacggcaagtacgacattaaagacgtgggcgt 2790
tggcatgaagtggaccggcgactaacgacgactgcccccaatacgcaagttcatacttttgccgttcatgctgtaatttctgcacccgca

155 160 165 170 175 180
Pro Tyr Phe Thr Trp Pro Leu Ile Ala Ala Asp Gly Gly Tyr Ala Phe Lys Tyr Glu Asn Gly Lys Tyr Asp Ile Lys Asp Val Gly Val
MBP

ggataacgctggcgcgaaagcgggtctgaccttcctggttgacctgattaaaaacaaacacatgaatgcagacaccgattactccatcgc 2880
cctattgcgaccgcgctttcgcccagactggaaggaccaactggactaatttttgtttgtgtacttacgtctgtggctaatgaggtagcg

185 190 195 200 205 210
Asp Asn Ala Gly Ala Lys Ala Gly Leu Thr Phe Leu Val Asp Leu Ile Lys Asn Lys His Met Asn Ala Asp Thr Asp Tyr Ser Ile Ala
MBP

agaagctgcctttaataaaggcgaaacagcgatgaccatcaacggcccgtgggcatggtccaacatcgacaccagcaaagtgaattatgg 2970
tcttcgacggaaattatttccgctttgtcgctactggtagttgccgggcacccgtaccaggttgtagctgtggtcgtttcacttaatacc

215 220 225 230 235 240
Glu Ala Ala Phe Asn Lys Gly Glu Thr Ala Met Thr Ile Asn Gly Pro Trp Ala Trp Ser Asn Ile Asp Thr Ser Lys Val Asn Tyr Gly
MBP

tgtaacggtactgccgaccttcaagggtcaaccatccaaaccgttcgttggcgtgctgagcgcaggtattaacgccgccagtccgaacaa 3060
acattgccatgacggctggaagttcccagttggtaggtttggcaagcaaccgcacgactcgcgtccataattgcggcggtcaggcttgtt

245 250 255 260 265 270
Val Thr Val Leu Pro Thr Phe Lys Gly Gln Pro Ser Lys Pro Phe Val Gly Val Leu Ser Ala Gly Ile Asn Ala Ala Ser Pro Asn Lys
MBP

agagctggcaaaagagttcctcgaaaactatctgctgactgatgaaggtctggaagcggttaataaagacaaaccgctgggtgccgtagc 3150
tctcgaccgttttctcaaggagcttttgatagacgactgactacttccagaccttcgccaattatttctgtttggcgacccacggcatcg

275 280 285 290 295 300
Glu Leu Ala Lys Glu Phe Leu Glu Asn Tyr Leu Leu Thr Asp Glu Gly Leu Glu Ala Val Asn Lys Asp Lys Pro Leu Gly Ala Val Ala
MBP

gctgaagtcttacgaggaagagttggcgaaagatccacgtattgccgccactatggaaaacgcccagaaaggtgaaatcatgccgaacat 3240
cgacttcagaatgctccttctcaaccgctttctaggtgcataacggcggtgataccttttgcgggtctttccactttagtacggcttgta

305 310 315 320 325 330
Leu Lys Ser Tyr Glu Glu Glu Leu Ala Lys Asp Pro Arg Ile Ala Ala Thr Met Glu Asn Ala Gln Lys Gly Glu Ile Met Pro Asn Ile
MBP

cccgcagatgtccgctttctggtatgccgtgcgtactgcggtgatcaacgccgccagcggtcgtcagactgtcgatgaagccctgaaaga 3330
gggcgtctacaggcgaaagaccatacggcacgcatgacgccactagttgcggcggtcgccagcagtctgacagctacttcgggactttct

335 340 345 350 355 360
Pro Gln Met Ser Ala Phe Trp Tyr Ala Val Arg Thr Ala Val Ile Asn Ala Ala Ser Gly Arg Gln Thr Val Asp Glu Ala Leu Lys Asp
MBP

cgcgcagactgaattcgagCCCGGGAGGCCTctcggcgcgcctgcaggtTACCCATACGACGTACCAGATTACGCTTACCCATACGACGT 3420
gcgcgtctgacttaagctcGGGCCCTCCGGAgagccgcgcggacgtccaATGGGTATGCTGCATGGTCTAATGCGAATGGGTATGCTGCA

365 1 5 10
Ala Gln Thr Glu Phe Glu Pro Gly Arg Pro Leu Gly Ala Pro Ala Gly Tyr Pro Tyr Asp Val Pro Asp Tyr Ala Tyr Pro Tyr Asp Val
MBP (in frame with MBP) 2×HA

ACCAGATTACGCTcgacaagcttgcggccgcataatgcttaagtaattaacctaggctgctgccaccgctgagcaataactagcataacc 3510
TGGTCTAATGCGAgctgttcgaacgccggcgtattacgaattcattaattggatccgacgacggtggcgactcgttattgatcgtattgg

15
Pro Asp Tyr Ala Arg Gln Ala Cys Gly Arg Ile Met Leu Lys *
2×HA (in frame with 2×HA) T7 terminator

ccttggggcctctaaacgggtcttgaggggttttttgctgaaacctcaggcatttgagaagcacacggtcacactgcttccggtagtcaa 3600
ggaaccccggagatttgcccagaactccccaaaaaacgactttggagtccgtaaactcttcgtgtgccagtgtgacgaaggccatcagtt

T7 terminator

taaaccggtaaaccagcaatagacataagcggctatttaacgaccctgccctgaaccgacgaccgggtcgaatttgctttcgaatttctg 3690
atttggccatttggtcgttatctgtattcgccgataaattgctgggacgggacttggctgctggcccagcttaaacgaaagcttaaagac

ccattcatccgcttattatcacttattcaggcgtagcaccaggcgtttaagggcaccaataactgccttaaaaaaattacgccccgccct 3780
ggtaagtaggcgaataatagtgaataagtccgcatcgtggtccgcaaattcccgtggttattgacggaatttttttaatgcggggcggga

220
* Ala Gly Gly Gln
CmR

gccactcatcgcagtactgttgtaattcattaagcattctgccgacatggaagccatcacagacggcatgatgaacctgaatcgccagcg 3870
cggtgagtagcgtcatgacaacattaagtaattcgtaagacggctgtaccttcggtagtgtctgccgtactacttggacttagcggtcgc

215 210 205 200 195 190
Trp Glu Asp Cys Tyr Gln Gln Leu Glu Asn Leu Met Arg Gly Val His Phe Gly Asp Cys Val Ala His His Val Gln Ile Ala Leu Pro
CmR

gcatcagcaccttgtcgccttgcgtataatatttgcccatagtgaaaacgggggcgaagaagttgtccatattggccacgtttaaatcaa 3960
cgtagtcgtggaacagcggaacgcatattataaacgggtatcacttttgcccccgcttcttcaacaggtataaccggtgcaaatttagtt

185 180 175 170 165 160
Met Leu Val Lys Asp Gly Gln Thr Tyr Tyr Lys Gly Met Thr Phe Val Pro Ala Phe Phe Asn Asp Met Asn Ala Val Asn Leu Asp Phe
CmR

aactggtgaaactcacccagggattggctgagacgaaaaacatattctcaataaaccctttagggaaataggccaggttttcaccgtaac 4050
ttgaccactttgagtgggtccctaaccgactctgctttttgtataagagttatttgggaaatccctttatccggtccaaaagtggcattg

155 150 145 140 135 130
Ser Thr Phe Ser Val Trp Pro Asn Ala Ser Val Phe Phe Met Asn Glu Ile Phe Gly Lys Pro Phe Tyr Ala Leu Asn Glu Gly Tyr Cys
CmR

acgccacatcttgcgaatatatgtgtagaaactgccggaaatcgtcgtggtattcactccagagcgatgaaaacgtttcagtttgctcat 4140
tgcggtgtagaacgcttatatacacatctttgacggcctttagcagcaccataagtgaggtctcgctacttttgcaaagtcaaacgagta

125 120 115 110 105 100
Ala Val Asp Gln Ser Tyr Ile His Leu Phe Gln Arg Phe Asp Asp His Tyr Glu Ser Trp Leu Ser Ser Phe Thr Glu Thr Gln Glu His
CmR

ggaaaacggtgtaacaagggtgaacactatcccatatcaccagctcaccgtctttcattgccatacggaactccggatgagcattcatca 4230
ccttttgccacattgttcccacttgtgatagggtatagtggtcgagtggcagaaagtaacggtatgccttgaggcctactcgtaagtagt

95 90 85 80 75 70
Phe Val Thr Tyr Cys Pro His Val Ser Asp Trp Ile Val Leu Glu Gly Asp Lys Met Ala Met Arg Phe Glu Pro His Ala Asn Met Leu
CmR

ggcgggcaagaatgtgaataaaggccggataaaacttgtgcttatttttctttacggtctttaaaaaggccgtaatatccagctgaacgg 4320
ccgcccgttcttacacttatttccggcctattttgaacacgaataaaaagaaatgccagaaatttttccggcattataggtcgacttgcc

65 60 55 50 45 40
Arg Ala Leu Ile His Ile Phe Ala Pro Tyr Phe Lys His Lys Asn Lys Lys Val Thr Lys Leu Phe Ala Thr Ile Asp Leu Gln Val Thr
CmR

tctggttataggtacattgagcaactgactgaaatgcctcaaaatgttctttacgatgccattgggatatatcaacggtggtatatccag 4410
agaccaatatccatgtaactcgttgactgactttacggagttttacaagaaatgctacggtaaccctatatagttgccaccatataggtc

35 30 25 20 15 10
Gln Asn Tyr Thr Cys Gln Ala Val Ser Gln Phe Ala Glu Phe His Glu Lys Arg His Trp Gln Ser Ile Asp Val Thr Thr Tyr Gly Thr
CmR

tgatttttttctccattttagcttccttagctcctgaaaatctcgataactcaaaaaatacgcccggtagtgatcttatttcattatggt 4500
actaaaaaaagaggtaaaatcgaaggaatcgaggacttttagagctattgagttttttatgcgggccatcactagaataaagtaatacca

5 1
Ile Lys Lys Glu Met
CmR
cat promoter

gaaagttggaacctcttacgtgccgatcaacgtctcattttcgccaaaagttggcccagggcttcccggtatcaacagggacaccaggat 4590
ctttcaaccttggagaatgcacggctagttgcagagtaaaagcggttttcaaccgggtcccgaagggccatagttgtccctgtggtccta

cat promoter

ttatttattctgcgaagtgatcttccgtcacaggtatttattcggcgcaaagtgcgtcgggtgatgctgccaacttactgatttagtgta 4680
aataaataagacgcttcactagaaggcagtgtccataaataagccgcgtttcacgcagcccactacgacggttgaatgactaaatcacat

```
tgatggtgtttttgaggtgctccagtggcttctgtttctatcagctgtccctcctgttcagctactgacggggtggtgcgtaacggcaaa
actaccacaaaaactccacgaggtcaccgaagacaaagatagtcgacagggaggacaagtcgatgactgccccaccacgcattgccgttt   4770

agcaccgccggacatcagcgctagcggagtgtatactggcttactatgttggcactgatgagggtgtcagtgaagtgcttcatgtggcag
tcgtggcggcctgtagtcgcgatcgcctcacatatgaccgaatgatacaaccgtgactactcccacagtcacttcacgaagtacaccgtc   4860

gagaaaaaaggctgcaccggtgcgtcagcagaatatgtgatacaggatatattccgcttcctcgctcactgactcgctacgctcggtcgt
ctcttttttccgacgtggccacgcagtcgtcttatacactatgtcctatataaggcgaaggagcgagtgactgagcgatgcgagccagca   4950

tcgactgcggcgagcggaaatggcttacgaacggggcggagatttcctggaagatgccaggaagatacttaacagggaagtgagagggcc
agctgacgccgctcgcctttaccgaatgcttgccccgcctctaaaggaccttctacggtccttctatgaattgtcccttcactctcccgg   5040

gcggcaaagccgtttttccataggctccgcccccctgacaagcatcacgaaatctgacgctcaaatcagtggtggcgaaacccgacagga
cgccgtttcggcaaaaaggtatccgaggcggggggactgttcgtagtgctttagactgcgagtttagtcaccaccgctttgggctgtcct   5130
p15A ori

ctataaagataccaggcgtttccccctggcggctccctcgtgcgctctcctgttcctgcctttcggtttaccggtgtcattccgctgttat
gatatttctatggtccgcaaagggggaccgccgagggagcacgcgagaggacaaggacggaaagccaaatggccacagtaaggcgacaata   5220
p15A ori

ggccgcgtttgtctcattccacgcctgacactcagttccgggtaggcagttcgctccaagctggactgtatgcacgaaccccccgttcag
ccggcgcaaacagagtaaggtgcggactgtgagtcaaggcccatccgtcaagcgaggttcgacctgacatacgtgcttggggggcaagtc   5310
p15A ori

tccgaccgctgcgccttatccggtaactatcgtcttgagtccaacccggaaagacatgcaaaagcaccactggcagcagccactggtaat
aggctggcgacgcggaataggccattgatagcagaactcaggttgggcctttctgtacgttttcgtggtgaccgtcgtcggtgaccatta   5400
p15A ori

tgatttagaggagttagtcttgaagtcatgcgccggttaaggctaaactgaaaggacaagttttggtgactgcgctcctccaagccagtt
actaaatctcctcaatcagaacttcagtacgcggccaattccgatttgactttcctgttcaaaaccactgacgcgaggaggttcggtcaa   5490
p15A ori

acctcggttcaaagagttggtagctcagagaaccttcgaaaaaccgccctgcaaggcggttttttcgttttcagagcaagagattacgcg
tggagccaagtttctcaaccatcgagtctcttggaagctttttggcgggacgttccgccaaaaaagcaaaagtctcgttctctaatgcgc   5580
p15A ori

cagaccaaaacgatctcaagaagatcatcttattaatcagataaaatatttctagatttcagtgcaatttatctcttcaaatgtagcacc
gtctggttttgctagagttcttctagtagaataattagtctattttataaagatctaaagtcacgttaaatagagaagtttacatcgtgg   5670
p15A ori

tgaagtcagccccatacgatataagttgtaattctcatgttagtcatgccccgcgcccaccggaaggagctgactgggttgaaggctctc
acttcagtcggggtatgctatattcaacattaagagtacaatcagtacggggcgcgggtggccttcctcgactgacccaacttccgagag   5760

aagggcatcggtcgagatcccggtgcctaatgagtgagctaacttacattaattgcgttgcgctcactgcccgctttccagtcgggaaac
ttcccgtagccagctctagggccacggattactcactcgattgaatgtaattaacgcaacgcgagtgacgggcgaaaggtcagccctttg   5850
     360                 355
*  Gln Gly Ser Glu Leu Arg Ser Val
lacI

ctgtcgtgccagctgcattaatgaatcggccaacgcgcggggagaggcggtttgcgtattgggcgccagggtggtttttcttttcaccag
gacagcacggtcgacgtaattacttagccggttgcgcgcccctctccgccaaacgcataacccgcggtcccaccaaaaagaaaagtggtc   5940
        350                 345                 340                 335                 330                 325
Gln Arg Ala Leu Gln Met Leu Ser Asp Ala Leu Ala Arg Pro Ser Ala Thr Gln Thr Asn Pro Ala Leu Thr Thr Lys Arg Lys Val Leu
lacI
```

tgagacgggcaacagctgattgcccttcaccgcctggccctgagagagttgcagcaagcggtccacgctggtttgccccagcaggcgaaa 6030
actctgcccgttgtcgactaacgggaagtggcggaccgggactctctcaacgtcgttcgccaggtgcgaccaaacggggtcgtccgcttt
320 315 310 305 300 295
Ser Val Pro Leu Leu Gln Asn Gly Lys Val Ala Gln Gly Gln Ser Leu Gln Leu Leu Arg Asp Val Ser Thr Gln Gly Leu Leu Arg Phe
lacI

atcctgtttgatggtggttaacggcgggatataacatgagctgtcttcggtatcgtcgtatcccactaccgagatgtccgcaccaacgcg 6120
taggacaaactaccaccaattgccgccctatattgtactcgacagaagccatagcagcatagggtgatggctctacaggcgtggttgcgc
290 285 280 275 270 265
Asp Gln Lys Ile Thr Thr Leu Pro Pro Ile Tyr Cys Ser Ser Asp Glu Thr Asp Asp Tyr Gly Val Val Ser Ile Asp Ala Gly Val Arg
lacI

cagcccggactcggtaatggcgcgcattgcgcccagcgccatctgatcgttggcaaccagcatcgcagtgggaacgatgccctcattcag 6210
gtcgggcctgagccattaccgcgcgtaacgcgggtcgcggtagactagcaaccgttggtcgtagcgtcacccttgctacgggagtaagtc
260 255 250 245 240 235
Leu Gly Ser Glu Thr Ile Ala Arg Met Ala Gly Leu Ala Met Gln Asp Asn Ala Val Leu Met Ala Thr Pro Val Ile Gly Glu Asn Leu
lacI

catttgcatggtttgttgaaaaccggacatggcactccagtcgccttcccgttccgctatcggctgaatttgattgcgagtgagatattt 6300
gtaaacgtaccaaacaacttttggcctgtaccgtgaggtcagcggaagggcaaggcgatagccgacttaaactaacgctcactctataaa
230 225 220 215 210 205
Met Gln Met Thr Gln Gln Phe Gly Ser Met Ala Ser Trp Asp Gly Glu Arg Glu Ala Ile Pro Gln Ile Gln Asn Arg Thr Leu Tyr Lys
lacI

atgccagccagccagacgcagacgcgccgagacagaacttaatgggcccgctaacagcgcgatttgctggtgacccaatgcgaccagatg 6390
tacggtcggtcggtctgcgtctgcgcggctctgtcttgaattacccgggcgattgtcgcgctaaacgaccactgggttacgctggtctac
200 195 190 185 180 175
His Trp Gly Ala Leu Arg Leu Arg Ala Ser Val Ser Ser Leu Pro Gly Ala Leu Leu Ala Ile Gln Gln His Gly Leu Ala Val Leu His
lacI

ctccacgcccagtcgcgtaccgtcttcatgggagaaaataatactgttgatgggtgtctggtcagagacatcaagaaataacgccggaac 6480
gaggtgcgggtcagcgcatggcagaagtaccctcttttattatgacaactacccacagaccagtctctgtagttctttattgcggccttg
170 165 160 155 150 145
Glu Val Gly Leu Arg Thr Gly Asp Glu His Ser Phe Ile Ile Ser Asn Ile Pro Thr Gln Asp Ser Val Asp Leu Phe Leu Ala Pro Val
lacI

attagtgcaggcagcttccacagcaatggcatcctggtcatccagcggatagttaatgatcagcccactgacgcgttgcgcgagaagatt 6570
taatcacgtccgtcgaaggtgtcgttaccgtaggaccagtaggtcgcctatcaattactagtcgggtgactgcgcaacgcgctcttctaa
140 135 130 125 120 115
Asn Thr Cys Ala Ala Glu Val Ala Ile Ala Asp Gln Asp Asp Leu Pro Tyr Asn Ile Ile Leu Gly Ser Val Arg Gln Ala Leu Leu Asn
lacI

gtgcaccgccgctttacaggcttcgacgccgcttcgttctaccatcgacaccaccacgctggcacccagttgatcggcgcgagatttaat 6660
cacgtggcggcgaaatgtccgaagctgcggcgaagcaagatggtagctgtggtggtgcgaccgtgggtcaactagccgcgctctaaatta
110 105 100 95 90 85
His Val Ala Ala Lys Cys Ala Glu Val Gly Ser Arg Glu Val Met Ser Val Val Val Ser Ala Gly Leu Gln Asp Ala Arg Ser Lys Ile
lacI

cgccgcgacaatttgcgacggcgcgtgcagggccagactggaggtggcaacgccaatcagcaacgactgtttgcccgccagttgttgtgc 6750
gcggcgctgttaaacgctgccgcgcacgtcccggtctgacctccaccgttgcggttagtcgttgctgacaaacgggcggtcaacaacacg
80 75 70 65 60 55
Ala Ala Val Ile Gln Ser Pro Ala His Leu Ala Leu Ser Ser Thr Ala Val Gly Ile Leu Leu Ser Gln Lys Gly Ala Leu Gln Gln Ala
lacI

cacgcggttgggaatgtaattcagctccgccatcgccgcttccactttttcccgcgttttcgcagaaacgtggctggcctggttcaccac 6840
gtgcgccaacccttacattaagtcgaggcggtagcggcgaaggtgaaaaagggcgcaaaagcgtctttgcaccgaccggaccaagtggtg
50 45 40 35 30 25
Val Arg Asn Pro Ile Tyr Asn Leu Glu Ala Met Ala Ala Glu Val Lys Glu Arg Thr Lys Ala Ser Val His Ser Ala Gln Asn Val Val
lacI

gcgggaaacggtctgataagagacaccggcatactctgcgacatcgtataacgttactggtttcacattcaccaccctgaattgactctc 6930
cgccctttgccagactattctctgtggccgtatgagacgctgtagcatattgcaatgaccaaagtgtaagtggtgggacttaactgagag

20 15 10 5 1
Arg Ser Val Thr Gln Tyr Ser Val Gly Ala Tyr Glu Ala Val Asp Tyr Leu Thr Val Pro Lys Met

lacI
lacI promoter

ttccgggcgctatcatgccataccgcgaaaggttttgcgccattcgatggtgtcc ••• 3′ 6985
aaggcccgcgatagtacggtatggcgctttccaaaacgcggtaagctaccacagg 5′

lacI promoter

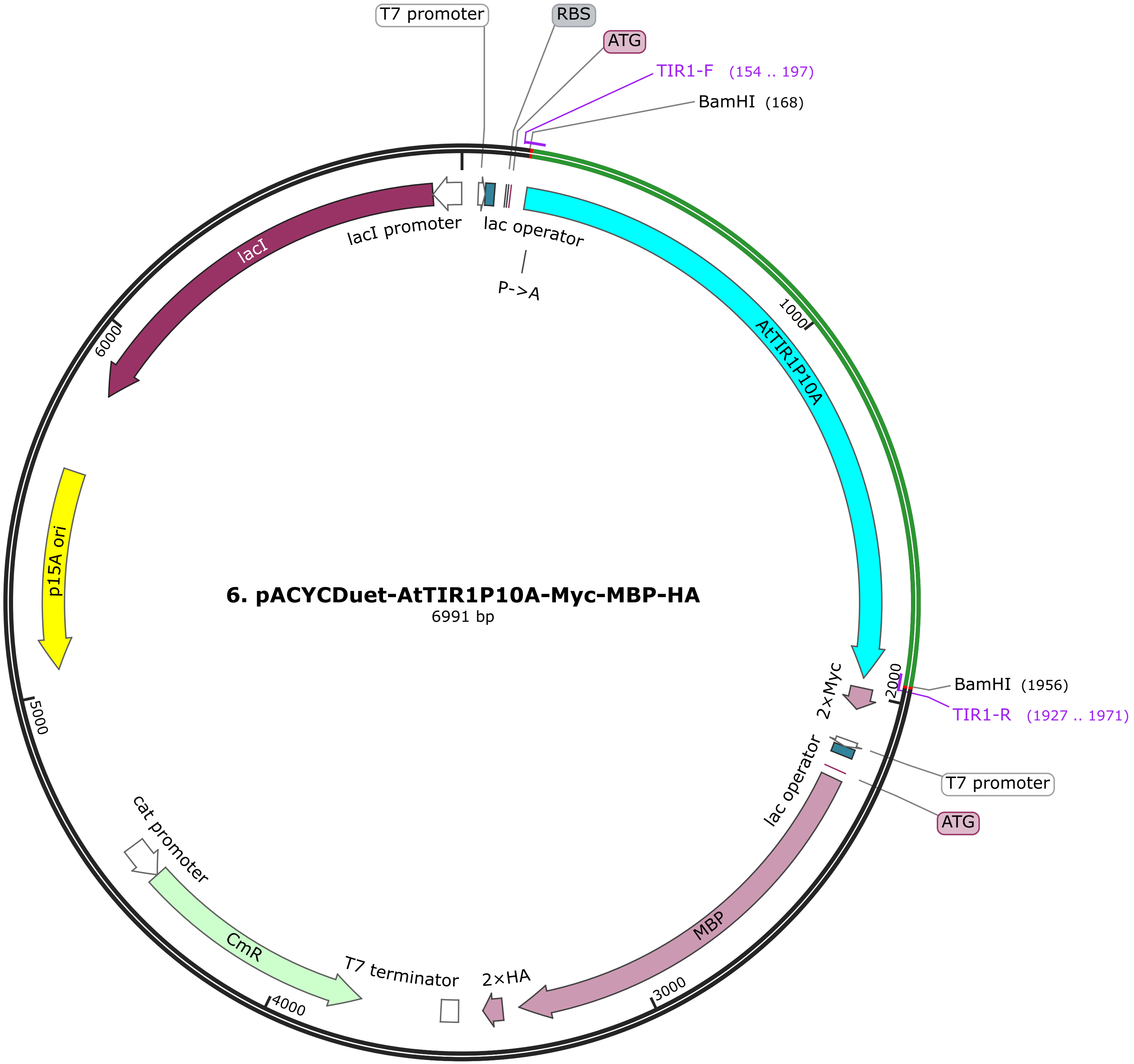

Created with SnapGene®
T7 promoter
RBS
ATG
TIR1-F (154 .. 197)
BamHI (168)
lacI promoter
lac operator
P->A
lacI
6000
1000
AtTIR1P10A
p15A ori
6. pACYCDuet-AtTIR1P10A-Myc-MBP-HA
6991 bp
2×Myc
2000
BamHI (1956)
TIR1-R (1927 .. 1971)
5000
lac operator
T7 promoter
ATG
cat promoter
MBP
CmR
T7 terminator
2×HA
3000
4000

5' gggatctcgacgctctcccttatgcgactcctgcattaggaattaatacgactcactataggggaattgtgagcggataacaattcccc 90
3' ccctagagctgcgagagggaatacgctgaggacgtaatcctttaattatgctgagtgatatccccttaacactcgcctattgttaagggg

T7 promoter
lac operator

BamHI
TIR1-F
catcaccaagccaggGatccATGCAGA

tgtagaaataattttgtttaactttaataaggagatataccatgggcagcagcGcatcaccatcatcaccaagccaggGatccATGCAGA 180
acatctttattaaaacaaattgaaattattcctctatatggtacccgtcgtcgCgtagtggtagtagtggttcggtccCtaggTACGTCT

1 Met Gly Ser Ser Ala Ser Pro Ser Ser Pro Ser Gln Gly Ser 1 Met Gln
RBS
ATG (in frame with ATG)
AtTIR1P10A

TIR1-F
AGCGAATAGCCTTGTCG

AGCGAATAGCCTTGTCGTTTGCAGAAGAGGTACTAGAGCATGTGTTCTCGTTTATTCAGCTGGATAAGGATAGGAACTCAGTCTCTCTGG 270
TCGCTTATCGGAACAGCAAACGTCTTCTCCATGATCTCGTACACAAGAGCAAATAAGTCGACCTATTCCTATCCTTGAGTCAGAGAGACC

Lys Arg 5 Ile Ala Leu Ser Phe 10 Ala Glu Glu Val Leu 15 Glu His Val Phe Ser 20 Phe Ile Gln Leu Asp 25 Lys Asp Arg Asn Ser 30 Val Ser Leu
AtTIR1P10A
P->A

TGTGCAAGTCATGGTACGAGATCGAGCGGTGGTGCAGGAGGAAAGTCTTCATCGGGAACTGCTACGCCGTGAGTCCAGCGACGGTGATTA 360
ACACGTTCAGTACCATGCTCTAGCTCGCCACCACGTCCTCCTTTCAGAAGTAGCCCTTGACGATGCGGCACTCAGGTCGCTGCCACTAAT

Val Cys 35 Lys Ser Trp Tyr Glu 40 Ile Glu Arg Trp Cys 45 Arg Arg Lys Val Phe 50 Ile Gly Asn Cys Tyr 55 Ala Val Ser Pro Ala 60 Thr Val Ile
AtTIR1P10A

GGAGGTTCCCGAAAGTGAGATCCGTGGAGCTTAAAGGAAAACCTCACTTTGCTGACTTTAATTTGGTACCTGACGGATGGGGAGGTTACG 450
CCTCCAAGGGCTTTCACTCTAGGCACCTCGAATTTCCTTTTGGAGTGAAACGACTGAAATTAAACCATGGACTGCCTACCCCTCCAATGC

Arg Arg 65 Phe Pro Lys Val Arg 70 Ser Val Glu Leu Lys 75 Gly Lys Pro His Phe 80 Ala Asp Phe Asn Leu 85 Val Pro Asp Gly Trp 90 Gly Gly Tyr
AtTIR1P10A

TGTATCCATGGATTGAGGCCATGTCTTCGTCTTACACGTGGCTTGAAGAGATAAGGCTGAAGAGGATGGTGGTCACCGACGATTGCTTGG 540
ACATAGGTACCTAACTCCGGTACAGAAGCAGAATGTGCACCGAACTTCTCTATTCCGACTTCTCCTACCACCAGTGGCTGCTAACGAACC

Val Tyr 95 Pro Trp Ile Glu Ala 100 Met Ser Ser Ser Tyr 105 Thr Trp Leu Glu Glu 110 Ile Arg Leu Lys Arg 115 Met Val Val Thr Asp 120 Asp Cys Leu
AtTIR1P10A

AGCTCATAGCCAAGTCTTTTAAGAATTTTAAGGTTCTTGTGCTTTCTTCCTGCGAAGGCTTCTCCACCGATGGTCTCGCTGCTATCGCTG 630
TCGAGTATCGGTTCAGAAAATTCTTAAAATTCCAAGAACACGAAAGAAGGACGCTTCCGAAGAGGTGGCTACCAGAGCGACGATAGCGAC

Glu Leu 125 Ile Ala Lys Ser Phe 130 Lys Asn Phe Lys Val 135 Leu Val Leu Ser Ser 140 Cys Glu Gly Phe Ser 145 Thr Asp Gly Leu Ala 150 Ala Ile Ala
AtTIR1P10A

CCACTTGCAGGAATCTGAAAGAGCTTGACTTACGAGAGAGTGATGTTGACGACGTTAGTGGCCACTGGCTTAGCCATTTCCCAGATACAT 720
GGTGAACGTCCTTAGACTTTCTCGAACTGAATGCTCTCTCACTACAACTGCTGCAATCACCGGTGACCGAATCGGTAAAGGGTCTATGTA

Ala Thr 155 Cys Arg Asn Leu Lys 160 Glu Leu Asp Leu Arg 165 Glu Ser Asp Val Asp 170 Asp Val Ser Gly His 175 Trp Leu Ser His Phe 180 Pro Asp Thr
AtTIR1P10A

ACACTTCTTTGGTATCACTCAATATATCTTGCTTAGCATCTGAGGTCAGTTTCTCTGCTCTGGAAAGGCTGGTGACTAGGTGTCCCAATC 810
TGTGAAGAAACCATAGTGAGTTATATAGAACGAATCGTAGACTCCAGTCAAAGAGACGAGACCTTTCCGACCACTGATCCACAGGGTTAG

Tyr Thr 185 Ser Leu Val Ser Leu 190 Asn Ile Ser Cys Leu 195 Ala Ser Glu Val Ser 200 Phe Ser Ala Leu Glu 205 Arg Leu Val Thr Arg 210 Cys Pro Asn
AtTIR1P10A

TCAAGTCTCTCAAGCTTAACCGAGCTGTTCCACTTGAAAAATTGGCTACTTTACTTCAAAGAGCACCTCAATTGGAGGAATTGGGCACTG 900
AGTTCAGAGAGTTCGAATTGGCTCGACAAGGTGAACTTTTTAACCGATGAAATGAAGTTTCTCGTGGAGTTAACCTCCTTAACCCGTGAC
215 220 225 230 235 240
Leu Lys Ser Leu Lys Leu Asn Arg Ala Val Pro Leu Glu Lys Leu Ala Thr Leu Leu Gln Arg Ala Pro Gln Leu Glu Glu Leu Gly Thr
AtTIR1P10A

GTGGGTACACTGCAGAAGTGCGACCAGATGTTTACTCTGGTTTATCTGTAGCGCTCTCTGGGTGCAAGGAATTGAGGTGCTTATCTGGAT 990
CACCCATGTGACGTCTTCACGCTGGTCTACAAATGAGACCAAATAGACATCGCGAGAGACCCACGTTCCTTAACTCCACGAATAGACCTA
245 250 255 260 265 270
Gly Gly Tyr Thr Ala Glu Val Arg Pro Asp Val Tyr Ser Gly Leu Ser Val Ala Leu Ser Gly Cys Lys Glu Leu Arg Cys Leu Ser Gly
AtTIR1P10A

TTTGGGATGCTGTTCCTGCCTATCTTCCAGCAGTTTATTCGGTTTGCAGTCGGCTTACAACTTTGAATCTGAGTTATGCAACAGTCCAGA 1080
AAACCCTACGACAAGGACGGATAGAAGGTCGTCAAATAAGCCAAACGTCAGCCGAATGTTGAAACTTAGACTCAATACGTTGTCAGGTCT
275 280 285 290 295 300
Phe Trp Asp Ala Val Pro Ala Tyr Leu Pro Ala Val Tyr Ser Val Cys Ser Arg Leu Thr Thr Leu Asn Leu Ser Tyr Ala Thr Val Gln
AtTIR1P10A

GCTATGATCTTGTCAAGCTTCTTTGTCAATGCCCTAAACTGCAGCGCCTCTGGGTGCTTGACTACATCGAGGATGCTGGTCTTGAGGTGC 1170
CGATACTAGAACAGTTCGAAGAAACAGTTACGGGATTTGACGTCGCGGAGACCCACGAACTGATGTAGCTCCTACGACCAGAACTCCACG
305 310 315 320 325 330
Ser Tyr Asp Leu Val Lys Leu Leu Cys Gln Cys Pro Lys Leu Gln Arg Leu Trp Val Leu Asp Tyr Ile Glu Asp Ala Gly Leu Glu Val
AtTIR1P10A

TTGCTTCAACCTGCAAGGACCTACGCGAGCTGAGAGTGTTTCCGTCCGAGCCTTTTGTCATGGAACCAAATGTGGCATTGACGGAACAGG 1260
AACGAAGTTGGACGTTCCTGGATGCGCTCGACTCTCACAAAGGCAGGCTCGGAAAACAGTACCTTGGTTTACACCGTAACTGCCTTGTCC
335 340 345 350 355 360
Leu Ala Ser Thr Cys Lys Asp Leu Arg Glu Leu Arg Val Phe Pro Ser Glu Pro Phe Val Met Glu Pro Asn Val Ala Leu Thr Glu Gln
AtTIR1P10A

GGCTTGTCTCCGTCTCCATGGGCTGTCCAAAACTCGAGTCGGTTCTCTACTTCTGCCGTCAAATGACCAATGCTGCATTGATAACCATTG 1350
CCGAACAGAGGCAGAGGTACCCGACAGGTTTTGAGCTCAGCCAAGAGATGAAGACGGCAGTTTACTGGTTACGACGTAACTATTGGTAAC
365 370 375 380 385 390
Gly Leu Val Ser Val Ser Met Gly Cys Pro Lys Leu Glu Ser Val Leu Tyr Phe Cys Arg Gln Met Thr Asn Ala Ala Leu Ile Thr Ile
AtTIR1P10A

CTAGGAACCGTCCCAACATGACTCGCTTCCGTTTGTGCATCATTGAGCCAAAAGCCCCAGACTATCTGACTCTAGAGCCACTGGATATTG 1440
GATCCTTGGCAGGGTTGTACTGAGCGAAGGCAAACACGTAGTAACTCGGTTTTCGGGGTCTGATAGACTGAGATCTCGGTGACCTATAAC
395 400 405 410 415 420
Ala Arg Asn Arg Pro Asn Met Thr Arg Phe Arg Leu Cys Ile Ile Glu Pro Lys Ala Pro Asp Tyr Leu Thr Leu Glu Pro Leu Asp Ile
AtTIR1P10A

GATTTGGAGCCATAGTAGAGCACTGCAAGGATCTCCGTCGCCTCTCTCTATCTGGCCTCTTGACCGACAAGGTTTTTGAATACATTGGGA 1530
CTAAACCTCGGTATCATCTCGTGACGTTCCTAGAGGCAGCGGAGAGAGATAGACCGGAGAACTGGCTGTTCCAAAAACTTATGTAACCCT
425 430 435 440 445 450
Gly Phe Gly Ala Ile Val Glu His Cys Lys Asp Leu Arg Arg Leu Ser Leu Ser Gly Leu Leu Thr Asp Lys Val Phe Glu Tyr Ile Gly
AtTIR1P10A

CATATGCCAAGAAGATGGAAATGCTCTCAGTGGCATTTGCAGGAGACAGTGACTTAGGCATGCATCATGTTTTGTCCGGGTGCGATAGCT 1620
GTATACGGTTCTTCTACCTTTACGAGAGTCACCGTAAACGTCCTCTGTCACTGAATCCGTACGTAGTACAAAACAGGCCCACGCTATCGA
455 460 465 470 475 480
Thr Tyr Ala Lys Lys Met Glu Met Leu Ser Val Ala Phe Ala Gly Asp Ser Asp Leu Gly Met His His Val Leu Ser Gly Cys Asp Ser
AtTIR1P10A

TGAGGAAACTAGAGATAAGGGACTGCCCGTTTGGAGACAAGGCGCTTTTGGCCAATGCTTCAAAGCTGGAGACAATGCGATCCCTTTGGA 1710
ACTCCTTTGATCTCTATTCCCTGACGGGCAAACCTCTGTTCCGCGAAAACCGGTTACGAAGTTTCGACCTCTGTTACGCTAGGGAAACCT
485 490 495 500 505 510
Leu Arg Lys Leu Glu Ile Arg Asp Cys Pro Phe Gly Asp Lys Ala Leu Leu Ala Asn Ala Ser Lys Leu Glu Thr Met Arg Ser Leu Trp
AtTIR1P10A

TGTCTTCTTGTTCCGTGAGTTTTGGAGCCTGCAAGTTACTAGGACAGAAGATGCCAAAGCTGAATGTGGAAGTCATCGATGAACGGGGTG 1800
ACAGAAGAACAAGGCACTCAAAACCTCGGACGTTCAATGATCCTGTCTTCTACGGTTTCGACTTACACCTTCAGTAGCTACTTGCCCCAC

515 520 525 530 535 540
Met Ser Ser Cys Ser Val Ser Phe Gly Ala Cys Lys Leu Leu Gly Gln Lys Met Pro Lys Leu Asn Val Glu Val Ile Asp Glu Arg Gly
AtTIR1P10A

CACCGGACTCGAGACCAGAGAGCTGCCCTGTTGAGAGAGTCTTCATATACCGAACAGTGGCTGGTCCTCGATTTGACATGCCTGGCTTCG 1890
GTGGCCTGAGCTCTGGTCTCTCGACGGGACAACTCTCTCAGAAGTATATGGCTTGTCACCGACCAGGAGCTAAACTGTACGGACCGAAGC

545 550 555 560 565 570
Ala Pro Asp Ser Arg Pro Glu Ser Cys Pro Val Glu Arg Val Phe Ile Tyr Arg Thr Val Ala Gly Pro Arg Phe Asp Met Pro Gly Phe
AtTIR1P10A

BamHI
TCTGGAACATGGACCAAGACTCAACAATGAGGTTTTCCAGGCAAATCATTACTACTAACGGATTAGGATCCGAGCTCGATATCACTAGTC 1980
AGACCTTGTACCTGGTTCTGAGTTGTTACTCCAAAAGGTCCGTTTAGTAATGATGATTGCCTAATCCTAGGCTCGAGCTATAGTGATCAG

575 580 585 590
Val Trp Asn Met Asp Gln Asp Ser Thr Met Arg Phe Ser Arg Gln Ile Ile Thr Thr Asn Gly Leu Gly Ser Glu Leu Asp Ile Thr Ser
AtTIR1P10A (in frame with AtTIR1P10A)

GGTCCGTTTAGTAATGATGATTGCCTAATCCTAGGCTCGAGCTAT
TIR1-R

CTGAGCAGAAGCTCATCTCCGAGGAGGACCTCGAGCAGAAGCTCATCTCCGAGGAGGACCTCCTGActgcaggtcgacaagcttgcggcc 2070
GACTCGTCTTCGAGTAGAGGCTCCTCCTGGAGCTCGTCTTCGAGTAGAGGCTCCTCCTGGAGGACTgacgtccagctgttcgaacgccgg

1 5 10 15 20
Pro Glu Gln Lys Leu Ile Ser Glu Glu Asp Leu Glu Gln Lys Leu Ile Ser Glu Glu Asp Leu Leu Thr Ala Gly Arg Gln Ala Cys Gly
2×Myc (in frame with 2×Myc)

gcataatgcttaagtcgaacagaaagtaatcgtattgtacacggccgcataatcgaaattaatacgactcactataggggaattgtgagc 2160
cgtattacgaattcagcttgtctttcattagcataacatgtgccggcgtattagctttaattatgctgagtgatatccccttaacactcg

Arg Ile Met Leu Lys Ser Asn Arg Lys
(in frame with 2×Myc)
T7 promoter lac operator

ggataacaattccccatcttagtatattagttaagtataagaaggagatatacatatggcagatctcaattgggaattcatgaaaatcga 2250
cctattgttaaggggtagaatcatataatcaattcatattcttcctctatatgtataccgtctagagttaacccttaagtacttttagct

1 1
Met Ala Asp Leu Asn Trp Glu Phe Met Lys Ile Glu
lac operator (in frame with ATG) MBP
ATG

agaaggtaaactggtaatctggattaacggcgataaaggctataacggtctcgctgaagtcggtaagaaattcgagaaagataccggaat 2340
tcttccatttgaccattagacctaattgccgctatttccgatattgccagagcgacttcagccattctttaagctctttctatggcctta

5 10 15 20 25 30
Glu Gly Lys Leu Val Ile Trp Ile Asn Gly Asp Lys Gly Tyr Asn Gly Leu Ala Glu Val Gly Lys Lys Phe Glu Lys Asp Thr Gly Ile
MBP

taaagtcaccgttgagcatccggataaactggaagagaaattcccacaggttgcggcaactggcgatggccctgacattatcttctgggc 2430
atttcagtggcaactcgtaggcctatttgaccttctctttaagggtgtccaacgccgttgaccgctaccgggactgtaatagaagacccg

35 40 45 50 55 60
Lys Val Thr Val Glu His Pro Asp Lys Leu Glu Glu Lys Phe Pro Gln Val Ala Ala Thr Gly Asp Gly Pro Asp Ile Ile Phe Trp Ala
MBP

acacgaccgctttggtggctacgctcaatctggcctgttggctgaaatcaccccggacaaagcgttccaggacaagctgtatccgtttac 2520
tgtgctggcgaaaccaccgatgcgagttagaccggacaaccgactttagtggggcctgtttcgcaaggtcctgttcgacataggcaaatg

65 70 75 80 85 90
His Asp Arg Phe Gly Gly Tyr Ala Gln Ser Gly Leu Leu Ala Glu Ile Thr Pro Asp Lys Ala Phe Gln Asp Lys Leu Tyr Pro Phe Thr
MBP

ctgggatgccgtacgttacaacggcaagctgattgcttacccgatcgctgttgaagcgttatcgctgatttataacaaagatctgctgcc 2610
gaccctacggcatgcaatgttgccgttcgactaacgaatgggctagcgacaacttcgcaatagcgactaaatattgtttctagacgacgg

95 100 105 110 115 120
Trp Asp Ala Val Arg Tyr Asn Gly Lys Leu Ile Ala Tyr Pro Ile Ala Val Glu Ala Leu Ser Leu Ile Tyr Asn Lys Asp Leu Leu Pro
MBP

```
gaacccgccaaaaacctgggaagagatcccggcgctggataaagaactgaaagcgaaaggtaagagcgcgctgatgttcaacctgcaaga   2700
cttgggcggtttttggacccttctctagggccgcgacctatttcttgactttcgctttccattctcgcgcgactacaagttggacgttct
125 Asn Pro Pro Lys Thr 130 Trp Glu Glu Ile Pro 135 Ala Leu Asp Lys Glu 140 Leu Lys Ala Lys Gly 145 Lys Ser Ala Leu Met 150 Phe Asn Leu Gln Glu
MBP

accgtacttcacctggccgctgattgctgctgacggggggttatgcgttcaagtatgaaaacggcaagtacgacattaaagacgtgggcgt   2790
tggcatgaagtggaccggcgactaacgacgactgccccccaatacgcaagttcatacttttgccgttcatgctgtaatttctgcacccgca
155 Pro Tyr Phe Thr Trp 160 Pro Leu Ile Ala Ala 165 Asp Gly Gly Tyr Ala 170 Phe Lys Tyr Glu Asn 175 Gly Lys Tyr Asp Ile 180 Lys Asp Val Gly Val
MBP

ggataacgctggcgcgaaagcgggtctgaccttcctggttgacctgattaaaaacaaacacatgaatgcagacaccgattactccatcgc   2880
cctattgcgaccgcgctttcgcccagactggaaggaccaactggactaatttttgtttgtgtacttacgtctgtggctaatgaggtagcg
185 Asp Asn Ala Gly Ala 190 Lys Ala Gly Leu Thr 195 Phe Leu Val Asp Leu 200 Ile Lys Asn Lys His 205 Met Asn Ala Asp Thr 210 Asp Tyr Ser Ile Ala
MBP

agaagctgcctttaataaaggcgaaacagcgatgaccatcaacggcccgtgggcatggtccaacatcgacaccagcaaagtgaattatgg   2970
tcttcgacggaaattatttccgctttgtcgctactggtagttgccgggcacccgtaccaggttgtagctgtggtcgtttcacttaatacc
215 Glu Ala Ala Phe Asn 220 Lys Gly Glu Thr Ala 225 Met Thr Ile Asn Gly 230 Pro Trp Ala Trp Ser 235 Asn Ile Asp Thr Ser 240 Lys Val Asn Tyr Gly
MBP

tgtaacggtactgccgaccttcaagggtcaaccatccaaaccgttcgttggcgtgctgagcgcaggtattaacgccgccagtccgaacaa   3060
acattgccatgacggctggaagttcccagttggtaggtttggcaagcaaccgcacgactcgcgtccataattgcggcggtcaggcttgtt
245 Val Thr Val Leu Pro 250 Thr Phe Lys Gly Gln 255 Pro Ser Lys Pro Phe 260 Val Gly Val Leu Ser 265 Ala Gly Ile Asn Ala 270 Ala Ser Pro Asn Lys
MBP

agagctggcaaaagagttcctcgaaaactatctgctgactgatgaaggtctggaagcggttaataaagacaaaccgctgggtgccgtagc   3150
tctcgaccgttttctcaaggagcttttgatagacgactgactacttccagaccttcgccaattatttctgtttggcgacccacggcatcg
275 Glu Leu Ala Lys Glu 280 Phe Leu Glu Asn Tyr 285 Leu Leu Thr Asp Glu 290 Gly Leu Glu Ala Val 295 Asn Lys Asp Lys Pro 300 Leu Gly Ala Val Ala
MBP

gctgaagtcttacgaggaagagttggcgaaagatccacgtattgccgccactatggaaaacgcccagaaaggtgaaatcatgccgaacat   3240
cgacttcagaatgctccttctcaaccgctttctaggtgcataacggcggtgataccttttgcgggtctttccactttagtacggcttgta
305 Leu Lys Ser Tyr Glu 310 Glu Glu Leu Ala Lys 315 Asp Pro Arg Ile Ala 320 Ala Thr Met Glu Asn 325 Ala Gln Lys Gly Glu 330 Ile Met Pro Asn Ile
MBP

cccgcagatgtccgctttctggtatgccgtgcgtactgcggtgatcaacgccgccagcggtcgtcagactgtcgatgaagccctgaaaga   3330
gggcgtctacaggcgaaagaccatacggcacgcatgacgccactagttgcggcggtcgccagcagtctgacagctacttcgggactttct
335 Pro Gln Met Ser Ala 340 Phe Trp Tyr Ala Val 345 Arg Thr Ala Val Ile 350 Asn Ala Ala Ser Gly 355 Arg Gln Thr Val Asp 360 Glu Ala Leu Lys Asp
MBP

cgcgcagactgaattcgagCCCGGGAGGCCTAGGCCTctcggcgcgcctgcaggtTACCCATACGACGTACCAGATTACGCTTACCCATA   3420
gcgcgtctgacttaagctcGGGCCCTCCGGATCCGGAgagccgcgcggacgtccaATGGGTATGCTGCATGGTCTAATGCGAATGGGTAT
365 Ala Gln Thr  Glu Phe Glu Pro Gly Arg Pro Arg Pro Leu Gly Ala Pro Ala Gly  1 Tyr Pro Tyr Asp 5 Val Pro Asp Tyr Ala 10 Tyr Pro Tyr
MBP  (in frame with MBP)  2×HA

CGACGTACCAGATTACGCTcgacaagcttgcggccgcataatgcttaagtaattaacctaggctgctgccaccgctgagcaataactagc   3510
GCTGCATGGTCTAATGCGAgctgttcgaacgccggcgtattacgaattcattaattggatccgacgacggtggcgactcgttattgatcg
Asp Val 15 Pro Asp Tyr Ala  Arg Gln Ala Cys Gly Arg Ile Met Leu Lys *
2×HA  (in frame with 2×HA)
T7 terminator

ataaccccttggggcctctaaacgggtcttgaggggttttttgctgaaacctcaggcatttgagaagcacacggtcacactgcttccggt   3600
tattggggaaccccggagatttgcccagaactccccaaaaaacgactttggagtccgtaaactcttcgtgtgccagtgtgacgaaggcca
T7 terminator
```

```
agtcaataaaccggtaaaccagcaatagacataagcggctatttaacgaccctgccctgaaccgacgaccgggtcgaatttgctttcgaa   3690
tcagttatttggccatttggtcgttatctgtattcgccgataaattgctgggacgggacttggctgctggcccagcttaaacgaaagctt

tttctgccattcatccgcttattatcacttattcaggcgtagcaccaggcgtttaagggcaccaataactgccttaaaaaaattacgccc   3780
aaagacggtaagtaggcgaataatagtgaataagtccgcatcgtggtccgcaaattcccgtggttattgacggaatttttttaatgcggg
220 * Ala Gly
CmR

cgccctgccactcatcgcagtactgttgtaattcattaagcattctgccgacatggaagccatcacagacggcatgatgaacctgaatcg   3870
gcgggacggtgagtagcgtcatgacaacattaagtaattcgtaagacggctgtaccttcggtagtgtctgccgtactacttggacttagc
215 210 205 200 195 190
Gly Gln Trp Glu Asp Cys Tyr Gln Gln Leu Glu Asn Leu Met Arg Gly Val His Phe Gly Asp Cys Val Ala His His Val Gln Ile Ala
CmR

ccagcggcatcagcaccttgtcgccttgcgtataatatttgcccatagtgaaaacgggggcgaagaagttgtccatattggccacgttta   3960
ggtcgccgtagtcgtggaacagcggaacgcatattataaacgggtatcacttttgcccccgcttcttcaacaggtataaccggtgcaaat
185 180 175 170 165 160
Leu Pro Met Leu Val Lys Asp Gly Gln Thr Tyr Tyr Lys Gly Met Thr Phe Val Pro Ala Phe Phe Asn Asp Met Asn Ala Val Asn Leu
CmR

aatcaaaactggtgaaactcacccagggattggctgagacgaaaaacatattctcaataaaccctttagggaaataggccaggttttcac   4050
ttagttttgaccactttgagtgggtccctaaccgactctgctttttgtataagagttatttgggaaatccctttatccggtccaaaagtg
155 150 145 140 135 130
Asp Phe Ser Thr Phe Ser Val Trp Pro Asn Ala Ser Val Phe Phe Met Asn Glu Ile Phe Gly Lys Pro Phe Tyr Ala Leu Asn Glu Gly
CmR

cgtaacacgccacatcttgcgaatatatgtgtagaaactgccggaaatcgtcgtggtattcactccagagcgatgaaaacgtttcagttt   4140
gcattgtgcggtgtagaacgcttatatacacatctttgacggcctttagcagcaccataagtgaggtctcgctacttttgcaaagtcaaa
125 120 115 110 105 100
Tyr Cys Ala Val Asp Gln Ser Tyr Ile His Leu Phe Gln Arg Phe Asp Asp His Tyr Glu Ser Trp Leu Ser Ser Phe Thr Glu Thr Gln
CmR

gctcatggaaaacggtgtaacaagggtgaacactatcccatatcaccagctcaccgtctttcattgccatacggaactccggatgagcat   4230
cgagtaccttttgccacattgttcccacttgtgatagggtatagtggtcgagtggcagaaagtaacggtatgccttgaggcctactcgta
95 90 85 80 75 70
Glu His Phe Val Thr Tyr Cys Pro His Val Ser Asp Trp Ile Val Leu Glu Gly Asp Lys Met Ala Met Arg Phe Glu Pro His Ala Asn
CmR

tcatcaggcgggcaagaatgtgaataaaggccggataaaacttgtgcttatttttctttacggtctttaaaaaggccgtaatatccagct   4320
agtagtccgcccgttcttacacttatttccggcctattttgaacacgaataaaaagaaatgccagaaatttttccggcattataggtcga
65 60 55 50 45 40
Met Leu Arg Ala Leu Ile His Ile Phe Ala Pro Tyr Phe Lys His Lys Asn Lys Lys Val Thr Lys Leu Phe Ala Thr Ile Asp Leu Gln
CmR

gaacggtctggttataggtacattgagcaactgactgaaatgcctcaaaatgttctttacgatgccattgggatatatcaacggtggtat   4410
cttgccagaccaatatccatgtaactcgttgactgactttacggagttttacaagaaatgctacggtaaccctatatagttgccaccata
35 30 25 20 15 10
Val Thr Gln Asn Tyr Thr Cys Gln Ala Val Ser Gln Phe Ala Glu Phe His Glu Lys Arg His Trp Gln Ser Ile Asp Val Thr Thr Tyr
CmR

atccagtgatttttttctccatttagcttccttagctcctgaaaatctcgataactcaaaaaatacgcccggtagtgatcttatttcat   4500
taggtcactaaaaaaagaggtaaatcgaaggaatcgaggacttttagagctattgagttttttatgcgggccatcactagaataaagta
5 1
Gly Thr Ile Lys Lys Glu Met
CmR    cat promoter

tatggtgaaagttggaacctcttacgtgccgatcaacgtctcatttcgccaaaagttggcccagggcttcccggtatcaacagggacac   4590
ataccactttcaaccttggagaatgcacggctagttgcagagtaaaagcggttttcaaccgggtcccgaagggccatagttgtccctgtg
cat promoter
```

caggatttatttattctgcgaagtgatcttccgtcacaggtatttattcggcgcaaagtgcgtcgggtgatgctgccaacttactgattt
4680
gtcctaaataaataagacgcttcactagaaggcagtgtccataaataagccgcgtttcacgcagcccactacgacggttgaatgactaaa

agtgtatgatggtgtttttgaggtgctccagtggcttctgtttctatcagctgtccctcctgttcagctactgacggggtggtgcgtaac
4770
tcacatactaccacaaaaactccacgaggtcaccgaagacaaagatagtcgacagggaggacaagtcgatgactgccccaccacgcattg

ggcaaaagcaccgccggacatcagcgctagcggagtgtatactggcttactatgttggcactgatgagggtgtcagtgaagtgcttcatg
4860
ccgttttcgtggcggcctgtagtcgcgatcgcctcacatatgaccgaatgatacaaccgtgactactcccacagtcacttcacgaagtac

tggcaggagaaaaaaggctgcaccggtgcgtcagcagaatatgtgatacaggatatattccgcttcctcgctcactgactcgctacgctc
4950
accgtcctcttttttccgacgtggccacgcagtcgtcttatacactatgtcctatataaggcgaaggagcgagtgactgagcgatgcgag

ggtcgttcgactgcggcgagcggaaatggcttacgaacggggcggagatttcctggaagatgccaggaagatacttaacagggaagtgag
5040
ccagcaagctgacgccgctcgcctttaccgaatgcttgccccgcctctaaaggaccttctacggtccttctatgaattgtcccttcactc

agggccgcggcaaagccgtttttccataggctccgcccccctgacaagcatcacgaaatctgacgctcaaatcagtggtggcgaaacccg
5130
tcccggcgccgtttcggcaaaaaggtatccgaggcgggggggactgttcgtagtgctttagactgcgagtttagtcaccaccgctttgggc
p15A ori

acaggactataaagataccaggcgtttccccctggcggctccctcgtgcgctctcctgttcctgcctttcggtttaccggtgtcattccgc
5220
tgtcctgatatttctatggtccgcaaagggggaccgccgagggagcacgcgagaggacaaggacggaaagccaaatggccacagtaaggcg
p15A ori

tgttatggccgcgtttgtctcattccacgcctgacactcagttccgggtaggcagttcgctccaagctggactgtatgcacgaacccccc
5310
acaataccggcgcaaacagagtaaggtgcggactgtgagtcaaggcccatccgtcaagcgaggttcgacctgacatacgtgcttgggggg
p15A ori

gttcagtccgaccgctgcgccttatccggtaactatcgtcttgagtccaacccggaaagacatgcaaaagcaccactggcagcagccact
5400
caagtcaggctggcgacgcggaataggccattgatagcagaactcaggttgggcctttctgtacgttttcgtggtgaccgtcgtcggtga
p15A ori

ggtaattgatttagaggagttagtcttgaagtcatgcgccggttaaggctaaactgaaaggacaagttttggtgactgcgctcctccaag
5490
ccattaactaaatctcctcaatcagaacttcagtacgcggccaattccgatttgactttcctgttcaaaaccactgacgcgaggaggttc
p15A ori

ccagttacctcggttcaaagagttggtagctcagagaaccttcgaaaaaccgccctgcaaggcggttttttcgttttcagagcaagagat
5580
ggtcaatggagccaagtttctcaaccatcgagtctcttggaagctttttggcgggacgttccgccaaaaaagcaaaagtctcgttctcta
p15A ori

tacgcgcagaccaaaacgatctcaagaagatcatcttattaatcagataaaatatttctagatttcagtgcaatttatctcttcaaatgt
5670
atgcgcgtctggttttgctagagttcttctagtagaataattagtctattttataaagatctaaagtcacgttaaatagagaagtttaca
p15A ori

agcacctgaagtcagccccatacgatataagttgtaattctcatgttagtcatgccccgcgcccaccggaaggagctgactgggttgaag
5760
tcgtggacttcagtcggggtatgctatattcaacattaagagtacaatcagtacggggcgcgggtggccttcctcgactgacccaacttc

gctctcaagggcatcggtcgagatcccggtgcctaatgagtgagctaacttacattaattgcgttgcgctcactgcccgctttccagtcg
5850
cgagagttcccgtagccagctctagggccacggattactcactcgattgaatgtaattaacgcaacgcgagtgacgggcgaaaggtcagc
360 355
* Gln Gly Ser Glu Leu Arg
lacI

ggaaacctgtcgtgccagctgcattaatgaatcggccaacgcgcggggagaggcggtttgcgtattgggcgccagggtggtttttctttt 5940
cctttggacagcacggtcgacgtaattacttagccggttgcgcgcccctctccgccaaacgcataacccgcggtcccaccaaaaagaaaa
350 345 340 335 330 325
Ser Val Gln Arg Ala Leu Gln Met Leu Ser Asp Ala Leu Ala Arg Pro Ser Ala Thr Gln Thr Asn Pro Ala Leu Thr Thr Lys Arg Lys
lacI

caccagtgagacgggcaacagctgattgcccttcaccgcctggccctgagagagttgcagcaagcggtccacgctggtttgccccagcag 6030
gtggtcactctgcccgttgtcgactaacgggaagtggcggaccgggactctctcaacgtcgttcgccaggtgcgaccaaacggggtcgtc
320 315 310 305 300 295
Val Leu Ser Val Pro Leu Leu Gln Asn Gly Lys Val Ala Gln Gly Gln Ser Leu Gln Leu Leu Arg Asp Val Ser Thr Gln Gly Leu Leu
lacI

gcgaaaatcctgtttgatggtggttaacggcgggatataacatgagctgtcttcggtatcgtcgtatcccactaccgagatgtccgcacc 6120
cgcttttaggacaaactaccaccaattgccgccctatattgtactcgacagaagccatagcagcatagggtgatggctctacaggcgtgg
290 285 280 275 270 265
Arg Phe Asp Gln Lys Ile Thr Thr Leu Pro Pro Ile Tyr Cys Ser Ser Asp Glu Thr Asp Asp Tyr Gly Val Val Ser Ile Asp Ala Gly
lacI

aacgcgcagcccggactcggtaatggcgcgcattgcgcccagcgccatctgatcgttggcaaccagcatcgcagtgggaacgatgccctc 6210
ttgcgcgtcgggcctgagccattaccgcgcgtaacgcgggtcgcggtagactagcaaccgttggtcgtagcgtcacccttgctacgggag
260 255 250 245 240 235
Val Arg Leu Gly Ser Glu Thr Ile Ala Arg Met Ala Gly Leu Ala Met Gln Asp Asn Ala Val Leu Met Ala Thr Pro Val Ile Gly Glu
lacI

attcagcatttgcatggtttgttgaaaaccggacatggcactccagtcgccttcccgttccgctatcggctgaatttgattgcgagtgag 6300
taagtcgtaaacgtaccaaacaacttttggcctgtaccgtgaggtcagcggaagggcaaggcgatagccgacttaaactaacgctcactc
230 225 220 215 210 205
Asn Leu Met Gln Met Thr Gln Gln Phe Gly Ser Met Ala Ser Trp Asp Gly Glu Arg Glu Ala Ile Pro Gln Ile Gln Asn Arg Thr Leu
lacI

atatttatgccagccagccagacgcagacgcgccgagacagaacttaatgggcccgctaacagcgcgatttgctggtgacccaatgcgac 6390
tataaatacggtcggtcggtctgcgtctgcgcggctctgtcttgaattacccgggcgattgtcgcgctaaacgaccactgggttacgctg
200 195 190 185 180 175
Tyr Lys His Trp Gly Ala Leu Arg Leu Arg Ala Ser Val Ser Ser Leu Pro Gly Ala Leu Leu Ala Ile Gln Gln His Gly Leu Ala Val
lacI

cagatgctccacgcccagtcgcgtaccgtcttcatgggagaaaataatactgttgatgggtgtctggtcagagacatcaagaaataacgc 6480
gtctacgaggtgcgggtcagcgcatggcagaagtaccctcttttattatgacaactacccacagaccagtctctgtagttctttattgcg
170 165 160 155 150 145
Leu His Glu Val Gly Leu Arg Thr Gly Asp Glu His Ser Phe Ile Ile Ser Asn Ile Pro Thr Gln Asp Ser Val Asp Leu Phe Leu Ala
lacI

cggaacattagtgcaggcagcttccacagcaatggcatcctggtcatccagcggatagttaatgatcagcccactgacgcgttgcgcgag 6570
gccttgtaatcacgtccgtcgaaggtgtcgttaccgtaggaccagtaggtcgcctatcaattactagtcgggtgactgcgcaacgcgctc
140 135 130 125 120 115
Pro Val Asn Thr Cys Ala Ala Glu Val Ala Ile Ala Asp Gln Asp Asp Leu Pro Tyr Asn Ile Ile Leu Gly Ser Val Arg Gln Ala Leu
lacI

aagattgtgcaccgccgctttacaggcttcgacgccgcttcgttctaccatcgacaccaccacgctggcacccagttgatcggcgcgaga 6660
ttctaacacgtggcggcgaaatgtccgaagctgcggcgaagcaagatggtagctgtggtggtgcgaccgtgggtcaactagccgcgctct
110 105 100 95 90 85
Leu Asn His Val Ala Ala Lys Cys Ala Glu Val Gly Ser Arg Glu Val Met Ser Val Val Val Ser Ala Gly Leu Gln Asp Ala Arg Ser
lacI

tttaatcgccgcgacaatttgcgacggcgcgtgcagggccagactggaggtggcaacgccaatcagcaacgactgtttgcccgccagttg 6750
aaattagcggcgctgttaaacgctgccgcgcacgtcccggtctgacctccaccgttgcggttagtcgttgctgacaaacgggcggtcaac
80 75 70 65 60 55
Lys Ile Ala Ala Val Ile Gln Ser Pro Ala His Leu Ala Leu Ser Ser Thr Ala Val Gly Ile Leu Leu Ser Gln Lys Gly Ala Leu Gln
lacI

```
ttgtgccacgcggttgggaatgtaattcagctccgccatcgccgcttccactttttcccgcgttttcgcagaaacgtggctggcctggtt   6840
aacacggtgcgccaacccttacattaagtcgaggcggtagcggcgaaggtgaaaaagggcgcaaaagcgtctttgcaccgaccggaccaa
                 50                 45                 40                 35                 30                 25
Gln Ala Val Arg Asn Pro Ile Tyr Asn Leu Glu Ala Met Ala Ala Glu Val Lys Glu Arg Thr Lys Ala Ser Val His Ser Ala Gln Asn
lacI

caccacgcgggaaacggtctgataagagacaccggcatactctgcgacatcgtataacgttactggtttcacattcaccaccctgaattg   6930
gtggtgcgccctttgccagactattctctgtggccgtatgagacgctgtagcatattgcaatgaccaaagtgtaagtggtgggacttaac
                 20                 15                 10                 5                 1
Val Val Arg Ser Val Thr Gln Tyr Ser Val Gly Ala Tyr Glu Ala Val Asp Tyr Leu Thr Val Pro Lys Met
lacI                                                                    lacI promoter

actctcttccgggcgctatcatgccataccgcgaaaggttttgcgccattcgatggtgtcc   ••• 3′   6991
tgagagaaggcccgcgatagtacggtatggcgctttccaaaacgcggtaagctaccacagg       5′
lacI promoter
```

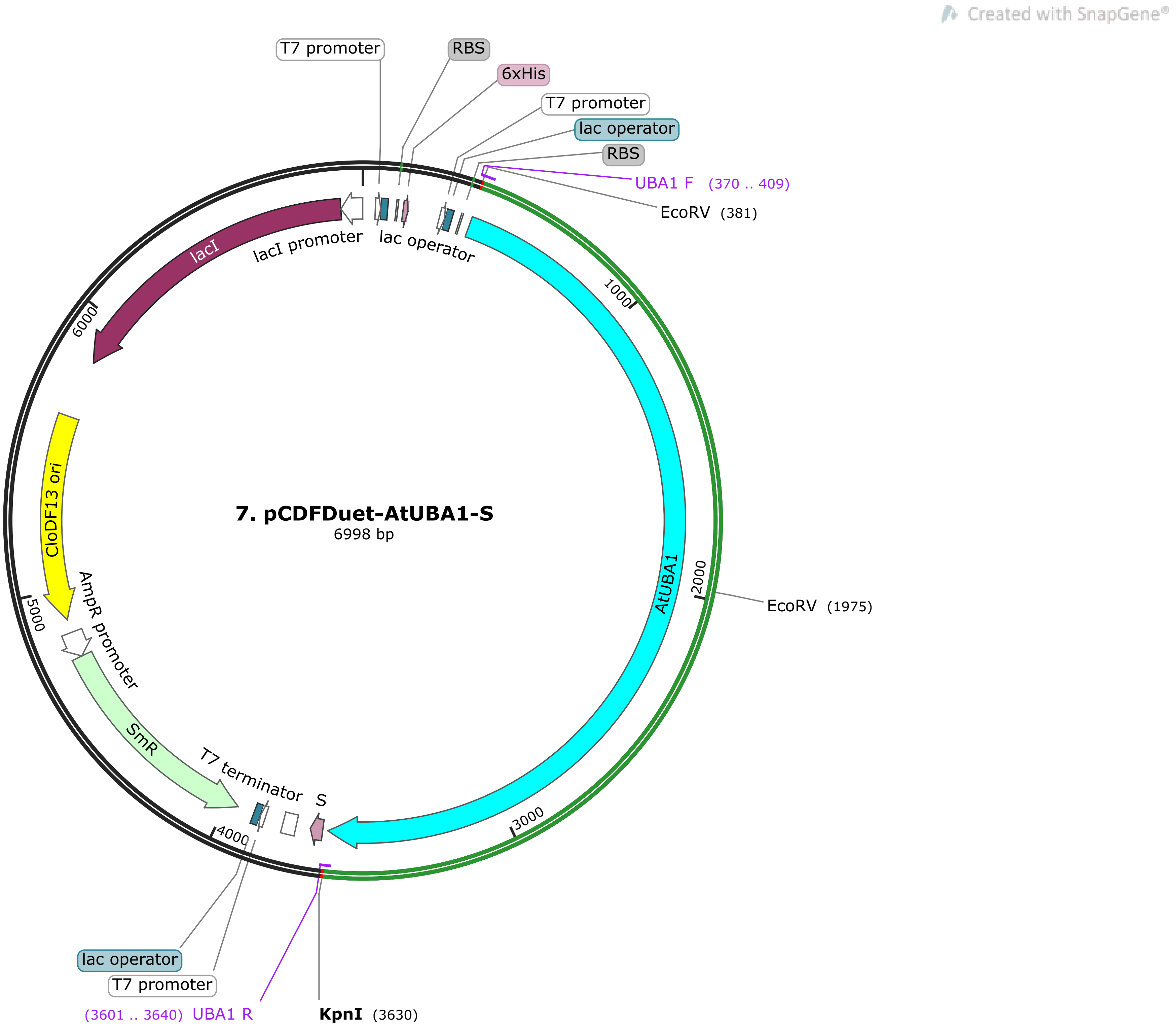

Created with SnapGene®
T7 promoter
RBS
6xHis
T7 promoter
lac operator
RBS
UBA1 F (370 .. 409)
EcoRV (381)
lacI promoter
lac operator
lacI
1000
6000
7. pCDFDuet-AtUBA1-S
6998 bp
CloDF13 ori
AtUBA1
2000
EcoRV (1975)
5000
AmpR promoter
SmR
T7 terminator
S
3000
4000
lac operator
T7 promoter
(3601 .. 3640) UBA1 R
KpnI (3630)

Sequence: 7. pCDFDuet-AtUBA1-S.dna (Circular / 6998 bp)
Enzymes: < Unsaved Enzyme Set > (2 of 678 total)
Features: 19 total
Primers: 5 total

Unique Cutters **Bold**

5' gggatctcgacgctctcccttatgcgactcctgcattaggaaattaatacgactcactataggggaattgtgagcggataacaattcccc 90
3' ccctagagctgcgagagggaatacgctgaggacgtaatcctttaattatgctgagtgatatccccttaacactcgcctattgttaagggg

T7 promoter | lac operator

tgtagaaataattttgtttaactttaatAAGGAGatataccatgggcagcagccatcaccatcatcaccacagccaggatccgaattcg 180
acatctttattaaaacaaattgaaattaTTCCTCtatatggtacccgtcgtcggtagtggtagtagtggtgtcggtcctaggcttaagc

1 His His His His 5 His His Ser Gln Gly Ser Glu Phe

RBS | 6xHis | (in frame with 6xHis)

agctcggcgcgcctgcaggtcgacaagcttgcggccgcataatgcttaagtcgaacagaaagtaatcgtattgtacacggccgcataatc 270
tcgagccgcgcggacgtccagctgttcgaacgccggcgtattacgaattcagcttgtctttcattagcataacatgtgccggcgtattag

Glu Leu Gly Ala Pro Ala Gly Arg Gln Ala Cys Gly Arg Ile Met Leu Lys Ser Asn Arg Lys *
(in frame with 6xHis)

gaaattaatacgactcactataggggaattgtgagcggataacaattccccatcttagtatattagttaagtataagAAGGAGatataca 360
ctttaattatgctgagtgatatccccttaacactcgcctattgttaaggggtagaatcatataatcaattcatattcTTCCTCtatatgt

T7 promoter | lac operator | RBS

EcoRV

UBA1 F
tctcaattggatatcgATGCTTCACAAGCGAGCTAGTGAA

tatggcagatctcaattggatatcGATGCTTCACAAGCGAGCTAGTGAAGCTAACGATAAGAACGATAATACCATCATCGGTTCCGATTT 450
ataccgtctagagttaacctatagcTACGAAGTGTTCGCTCGATCACTTCGATTGCTATTCTTGCTATTATGGTAGTAGCCAAGGCTAAA

1 Met Leu His Lys 5 Arg Ala Ser Glu Ala 10 Asn Asp Lys Asn Asp 15 Asn Thr Ile Ile Gly 20 Ser Asp Leu
AtUBA1

AGCATCATCTAAGAAACGCCGTATCGATTTCACTGAATCTTCGTCTGATAAGTCTTCTTCGATTTTAGCTTCTGGTAGTAGCAGGGGTTT 540
TCGTAGTAGATTCTTTGCGGCATAGCTAAAGTGACTTAGAAGCAGACTATTCAGAAGAAGCTAAAATCGAAGACCATCATCGTCCCCAAA

Ala Ser 25 Ser Lys Lys Arg Arg 30 Ile Asp Phe Thr Glu 35 Ser Ser Ser Asp Lys 40 Ser Ser Ser Ile Leu 45 Ala Ser Gly Ser Ser 50 Arg Gly Phe
AtUBA1

TCACGGCGATAGCGTCGTTCAGCAAATCGACATGGCTTTTGGTAATTCGAACCGTCAGGAGATTGATGAAGATCTGCACAGTCGCCAGCT 630
AGTGCCGCTATCGCAGCAAGTCGTTTAGCTGTACCGAAAACCATTAAGCTTGGCAGTCCTCTAACTACTTCTAGACGTGTCAGCGGTCGA

His Gly 55 Asp Ser Val Val Gln 60 Gln Ile Asp Met Ala 65 Phe Gly Asn Ser Asn 70 Arg Gln Glu Ile Asp 75 Glu Asp Leu His Ser 80 Arg Gln Leu
AtUBA1

CGCCGTCTATGGTCGTGAGACTATGAGGCGTCTCTTTGCTTCCAATGTTCTCATCTCGGGGATGCACGGTCTTGGTGCCGAGATTGCCAA 720
GCGGCAGATACCAGCACTCTGATACTCCGCAGAGAAACGAAGGTTACAAGAGTAGAGCCCCTACGTGCCAGAACCACGGCTCTAACGGTT

Ala Val 85 Tyr Gly Arg Glu Thr 90 Met Arg Arg Leu Phe 95 Ala Ser Asn Val Leu 100 Ile Ser Gly Met His 105 Gly Leu Gly Ala Glu 110 Ile Ala Lys
AtUBA1

GAATCTTATACTTGCTGGTGTGAAGTCTGTGACCTTGCATGATGAAAGAGTTGTAGAGCTATGGGACTTATCAAGCAACTTTGTTTTCTC 810
CTTAGAATATGAACGACCACACTTCAGACACTGGAACGTACTACTTTCTCAACATCTCGATACCCTGAATAGTTCGTTGAAACAAAAGAG

Asn Leu 115 Ile Leu Ala Gly Val 120 Lys Ser Val Thr Leu 125 His Asp Glu Arg Val 130 Val Glu Leu Trp Asp 135 Leu Ser Ser Asn Phe 140 Val Phe Ser
AtUBA1

GGAGGATGATGTTGGCAAGAATAGAGCAGATGCTTCTGTTCAGAAGTTACAGGATCTTAACAATGCTGTGGTTGTCTCTAGCTTGACCAA 900
CCTCCTACTACAACCGTTCTTATCTCGTCTACGAAGACAAGTCTTCAATGTCCTAGAATTGTTACGACACCAACAGAGATCGAACTGGTT

Glu Asp 145 Asp Val Gly Lys Asn 150 Arg Ala Asp Ala Ser 155 Val Gln Lys Leu Gln 160 Asp Leu Asn Asn Ala 165 Val Val Val Ser Ser 170 Leu Thr Lys
AtUBA1

AAGCTTAAACAAAGAGGATCTTTCTGGCTTCCAGGTTGTTGTATTCTCTGACATAAGCATGGAAAGAGCAATCGAATTTGATGACTATTG 990
TTCGAATTTGTTTCTCCTAGAAAGACCGAAGGTCCAACAACATAAGAGACTGTATTCGTACCTTTCTCGTTAGCTTAAACTACTGATAAC
175 180 185 190 195 200
Ser Leu Asn Lys Glu Asp Leu Ser Gly Phe Gln Val Val Val Phe Ser Asp Ile Ser Met Glu Arg Ala Ile Glu Phe Asp Asp Tyr Cys
AtUBA1

TCACAGCCATCAGCCTCCTATCGCTTTTGTCAAGGCTGATGTCAGGGGTCTTTTCGGCTCTGTCTTTTGTGATTTTGGGCCTGAATTTGC 1080
AGTGTCGGTAGTCGGAGGATAGCGAAAACAGTTCCGACTACAGTCCCCAGAAAAGCCGAGACAGAAAACACTAAAACCCGGACTTAAACG
205 210 215 220 225 230
His Ser His Gln Pro Pro Ile Ala Phe Val Lys Ala Asp Val Arg Gly Leu Phe Gly Ser Val Phe Cys Asp Phe Gly Pro Glu Phe Ala
AtUBA1

GGTTCTTGATGTTGATGGAGAGGAACCCCATACAGGCATCATTGCGTCTATCTCTAATGAGAACCAGGCATTTATTTCCTGTGTCGACGA 1170
CCAAGAACTACAACTACCTCTCCTTGGGGTATGTCCGTAGTAACGCAGATAGAGATTACTCTTGGTCCGTAAATAAAGGACACAGCTGCT
235 240 245 250 255 260
Val Leu Asp Val Asp Gly Glu Glu Pro His Thr Gly Ile Ile Ala Ser Ile Ser Asn Glu Asn Gln Ala Phe Ile Ser Cys Val Asp Asp
AtUBA1

TGAGAGGCTTGAGTTTGAAGATGGTGACCTAGTTGTTTTCTCAGAAGTTGAGGGTATGACAGAATTGAATGATGGGAAACCAAGGAAGAT 1260
ACTCTCCGAACTCAAACTTCTACCACTGGATCAACAAAAGAGTCTTCAACTCCCATACTGTCTTAACTTACTACCCTTTGGTTCCTTCTA
265 270 275 280 285 290
Glu Arg Leu Glu Phe Glu Asp Gly Asp Leu Val Val Phe Ser Glu Val Glu Gly Met Thr Glu Leu Asn Asp Gly Lys Pro Arg Lys Ile
AtUBA1

AAAAAGTACACGGCCATATTCATTCACCCTTGATGAGGACACTACTAACTATGGAACATATGTGAAGGGTGGTATTGTCACTCAAGTGAA 1350
TTTTTCATGTGCCGGTATAAGTAAGTGGGAACTACTCCTGTGATGATTGATACCTTGTATACACTTCCCACCATAACAGTGAGTTCACTT
295 300 305 310 315 320
Lys Ser Thr Arg Pro Tyr Ser Phe Thr Leu Asp Glu Asp Thr Thr Asn Tyr Gly Thr Tyr Val Lys Gly Gly Ile Val Thr Gln Val Lys
AtUBA1

ACAGCCAAAGTTACTGAATTTCAAGCCATTGAGGGAAGCGCTTAAGGATCCAGGGGATTTTCTATTTAGTGATTTCTCTAAGTTTGATCG 1440
TGTCGGTTTCAATGACTTAAAGTTCGGTAACTCCCTTCGCGAATTCCTAGGTCCCCTAAAAGATAAATCACTAAAGAGATTCAAACTAGC
325 330 335 340 345 350
Gln Pro Lys Leu Leu Asn Phe Lys Pro Leu Arg Glu Ala Leu Lys Asp Pro Gly Asp Phe Leu Phe Ser Asp Phe Ser Lys Phe Asp Arg
AtUBA1

GCCTCCACTCCTTCACTTAGCATTCCAGGCACTTGATCATTTTAAAGCTGAAGCTGGCAGATTTCCTGTTGCTGGCTCAGAAGAGGACGC 1530
CGGAGGTGAGGAAGTGAATCGTAAGGTCCGTGAACTAGTAAAATTTCGACTTCGACCGTCTAAAGGACAACGACCGAGTCTTCTCCTGCG
355 360 365 370 375 380
Pro Pro Leu Leu His Leu Ala Phe Gln Ala Leu Asp His Phe Lys Ala Glu Ala Gly Arg Phe Pro Val Ala Gly Ser Glu Glu Asp Ala
AtUBA1

TCAGAAGCTTATATCGATTGCCACAGCCATCAATACCGGGCAGGGTGATTTAAAGGTGGAGAATGTTGATCAGAAGCTTCTAAGACACTT 1620
AGTCTTCGAATATAGCTAACGGTGTCGGTAGTTATGGCCCGTCCCACTAAATTTCCACCTCTTACAACTAGTCTTCGAAGATTCTGTGAA
385 390 395 400 405 410
Gln Lys Leu Ile Ser Ile Ala Thr Ala Ile Asn Thr Gly Gln Gly Asp Leu Lys Val Glu Asn Val Asp Gln Lys Leu Leu Arg His Phe
AtUBA1

TTCCTTTGGAGCCAAGGCTGTCCTGAATCCTATGGCTGCAATGTTTGGCGGTATTGTTGGACAGGAGGTTGTTAAAGCTTGCTCTGGAAA 1710
AAGGAAACCTCGGTTCCGACAGGACTTAGGATACCGACGTTACAAACCGCCATAACAACCTGTCCTCCAACAATTTCGAACGAGACCTTT
415 420 425 430 435 440
Ser Phe Gly Ala Lys Ala Val Leu Asn Pro Met Ala Ala Met Phe Gly Gly Ile Val Gly Gln Glu Val Val Lys Ala Cys Ser Gly Lys
AtUBA1

ATTTCATCCTCTCTTCCAGTTTTTCTACTTTGATTCAGTGGAGTCACTCCCCTCTGAACCTGTGGACTCGAGTGACTTTGCACCAAGAAA 1800
TAAAGTAGGAGAGAAGGTCAAAAAGATGAAACTAAGTCACCTCAGTGAGGGGAGACTTGGACACCTGAGCTCACTGAAACGTGGTTCTTT
445 450 455 460 465 470
Phe His Pro Leu Phe Gln Phe Phe Tyr Phe Asp Ser Val Glu Ser Leu Pro Ser Glu Pro Val Asp Ser Ser Asp Phe Ala Pro Arg Asn
AtUBA1

```
CAGCCGGTATGATGCCCAAATATCTGTATTTGGGGCCAAGTTCCAGAAGAAACTTGAAGATGCTAAAGTTTTCACAGTAGGGTCTGGTGC 1890
GTCGGCCATACTACGGGTTTATAGACATAAACCCCGGTTCAAGGTCTTCTTTGAACTTCTACGATTTCAAAAGTGTCATCCCAGACCACG
475 480 485 490 495 500
Ser Arg Tyr Asp Ala Gln Ile Ser Val Phe Gly Ala Lys Phe Gln Lys Lys Leu Glu Asp Ala Lys Val Phe Thr Val Gly Ser Gly Ala
AtUBA1

EcoRV
TCTTGGCTGTGAGTTCTTGAAAAATTTGGCTCTGATGGGGGTTTCTTGTGGAAGCCAAGGGAAGCTGACAGTGACAGATGATGATATCAT 1980
AGAACCGACACTCAAGAACTTTTTAAACCGAGACTACCCCCAAAGAACACCTTCGGTTCCCTTCGACTGTCACTGTCTACTACTATAGTA
505 510 515 520 525 530
Leu Gly Cys Glu Phe Leu Lys Asn Leu Ala Leu Met Gly Val Ser Cys Gly Ser Gln Gly Lys Leu Thr Val Thr Asp Asp Asp Ile Ile
AtUBA1

TGAGAAGAGTAACCTCAGTCGTCAATTTCTGTTCCGTGACTGGAACATTGGACAGGCTAAATCCACAGTTGCTGCTTCGGCTGCTGCTGT 2070
ACTCTTCTCATTGGAGTCAGCAGTTAAAGACAAGGCACTGACCTTGTAACCTGTCCGATTTAGGTGTCAACGACGAAGCCGACGACGACA
535 540 545 550 555 560
Glu Lys Ser Asn Leu Ser Arg Gln Phe Leu Phe Arg Asp Trp Asn Ile Gly Gln Ala Lys Ser Thr Val Ala Ala Ser Ala Ala Ala Val
AtUBA1

TATAAATCCTCGCTTCAACATTGAGGCCCTGCAAAACCGTGTTGGCGCGGAGACTGAGAATGTATTTGACGATGCCTTCTGGGAGAACTT 2160
ATATTTAGGAGCGAAGTTGTAACTCCGGGACGTTTTGGCACAACCGCGCCTCTGACTCTTACATAAACTGCTACGGAAGACCCTCTTGAA
565 570 575 580 585 590
Ile Asn Pro Arg Phe Asn Ile Glu Ala Leu Gln Asn Arg Val Gly Ala Glu Thr Glu Asn Val Phe Asp Asp Ala Phe Trp Glu Asn Leu
AtUBA1

AACTGTTGTCGTCAATGCACTGGATAATGTTAATGCGAGGCTCTATGTTGATTCGAGGTGCTTGTATTTCCAGAAGCCTCTCCTTGAGTC 2250
TTGACAACAGCAGTTACGTGACCTATTACAATTACGCTCCGAGATACAACTAAGCTCCACGAACATAAAGGTCTTCGGAGAGGAACTCAG
595 600 605 610 615 620
Thr Val Val Val Asn Ala Leu Asp Asn Val Asn Ala Arg Leu Tyr Val Asp Ser Arg Cys Leu Tyr Phe Gln Lys Pro Leu Leu Glu Ser
AtUBA1

TGGAACTCTTGGTACAAAGTGCAACACACAGTCGGTTATCCCACATCTAACTGAAAATTACGGTGCCTCAAGGGACCCACCAGAGAAACA 2340
ACCTTGAGAACCATGTTTCACGTTGTGTGTCAGCCAATAGGGTGTAGATTGACTTTTAATGCCACGGAGTTCCCTGGGTGGTCTCTTTGT
625 630 635 640 645 650
Gly Thr Leu Gly Thr Lys Cys Asn Thr Gln Ser Val Ile Pro His Leu Thr Glu Asn Tyr Gly Ala Ser Arg Asp Pro Pro Glu Lys Gln
AtUBA1

GGCCCCCATGTGTACAGTGCATTCCTTCCCGCATAACATTGATCACTGTTTAACTTGGGCTCGCTCTGAGTTTGAGGGTCTGCTGGAGAA 2430
CCGGGGGTACACATGTCACGTAAGGAAGGGCGTATTGTAACTAGTGACAAATTGAACCCGAGCGAGACTCAAACTCCCAGACGACCTCTT
655 660 665 670 675 680
Ala Pro Met Cys Thr Val His Ser Phe Pro His Asn Ile Asp His Cys Leu Thr Trp Ala Arg Ser Glu Phe Glu Gly Leu Leu Glu Lys
AtUBA1

GACTCCCGCTGAAGTGAATGCATATCTTTCTAGCCCAGTTGAGTACACTAACTCAATGATGAGTGCTGGTGATGCTCAGGCGAGGGACAC 2520
CTGAGGGCGACTTCACTTACGTATAGAAAGATCGGGTCAACTCATGTGATTGAGTTACTACTCACGACCACTACGAGTCCGCTCCCTGTG
685 690 695 700 705 710
Thr Pro Ala Glu Val Asn Ala Tyr Leu Ser Ser Pro Val Glu Tyr Thr Asn Ser Met Met Ser Ala Gly Asp Ala Gln Ala Arg Asp Thr
AtUBA1

ATTGGAGAGGATTGTTGAGTGCCTTGAAAAGGAGAAGTGTGAGACCTTCCAAGACTGCTTAACCTGGGCACGACTGAGGTTTGAGGATTA 2610
TAACCTCTCCTAACAACTCACGGAACTTTTCCTCTTCACACTCTGGAAGGTTCTGACGAATTGGACCCGTGCTGACTCCAAACTCCTAAT
715 720 725 730 735 740
Leu Glu Arg Ile Val Glu Cys Leu Glu Lys Glu Lys Cys Glu Thr Phe Gln Asp Cys Leu Thr Trp Ala Arg Leu Arg Phe Glu Asp Tyr
AtUBA1

CTTTGTGAACCGTGTCAAGCAATTGATATACACATTTCCTGAAGATGCTGCGACTAGTACCGGAGCTCCATTCTGGTCTGCTCCAAAAAG 2700
GAAACACTTGGCACAGTTCGTTAACTATATGTGTAAAGGACTTCTACGACGCTGATCATGGCCTCGAGGTAAGACCAGACGAGGTTTTTC
745 750 755 760 765 770
Phe Val Asn Arg Val Lys Gln Leu Ile Tyr Thr Phe Pro Glu Asp Ala Ala Thr Ser Thr Gly Ala Pro Phe Trp Ser Ala Pro Lys Arg
AtUBA1
```

```
ATTCCCTCGTCCACTCCAGTACTCGTCGTCCGACCCAAGTCTCCTTAACTTTATCACAGCGACTGCTATTTTACGAGCAGAGACATTTGG   2790
TAAGGGAGCAGGTGAGGTCATGAGCAGCAGGCTGGGTTCAGAGGAATTGAAATAGTGTCGCTGACGATAAAATGCTCGTCTCTGTAAACC
        775         780         785         790         795         800
Phe Pro Arg Pro Leu Gln Tyr Ser Ser Ser Asp Pro Ser Leu Leu Asn Phe Ile Thr Ala Thr Ala Ile Leu Arg Ala Glu Thr Phe Gly
AtUBA1

GATCCCGATACCTGAGTGGACTAAGAACCCAAAGGAAGCAGCAGAAGCTGTTGACAGAGTGATAGTCCCAGACTTTGAGCCAAGGCAAGA   2880
CTAGGGCTATGGACTCACCTGATTCTTGGGTTTCCTTCGTCGTCTTCGACAACTGTCTCACTATCAGGGTCTGAAACTCGGTTCCGTTCT
        805         810         815         820         825         830
Ile Pro Ile Pro Glu Trp Thr Lys Asn Pro Lys Glu Ala Ala Glu Ala Val Asp Arg Val Ile Val Pro Asp Phe Glu Pro Arg Gln Asp
AtUBA1

TGCAAAGATTGTGACTGATGAGAAAGCTACCACTTTAACCACTGCTTCGGTGGACGACGCTGCAGTCATCGACGATCTCATTGCTAAGAT   2970
ACGTTTCTAACACTGACTACTCTTTCGATGGTGAAATTGGTGACGAAGCCACCTGCTGCGACGTCAGTAGCTGCTAGAGTAACGATTCTA
        835         840         845         850         855         860
Ala Lys Ile Val Thr Asp Glu Lys Ala Thr Thr Leu Thr Thr Ala Ser Val Asp Asp Ala Ala Val Ile Asp Asp Leu Ile Ala Lys Ile
AtUBA1

CGACCAGTGTAGGCATAACTTGTCGCCAGACTTCAGGATGAAACCTATTCAGTTCGAAAAGGATGATGATACAAACTATCACATGGATGT   3060
GCTGGTCACATCCGTATTGAACAGCGGTCTGAAGTCCTACTTTGGATAAGTCAAGCTTTTCCTACTACTATGTTTGATAGTGTACCTACA
        865         870         875         880         885         890
Asp Gln Cys Arg His Asn Leu Ser Pro Asp Phe Arg Met Lys Pro Ile Gln Phe Glu Lys Asp Asp Asp Thr Asn Tyr His Met Asp Val
AtUBA1

GATAGCAGGACTAGCCAACATGAGGGCGAGAAATTACAGCATCCCTGAAGTGGACAAGCTGAAAGCAAAGTTCATAGCAGGGAGAATCAT   3150
CTATCGTCCTGATCGGTTGTACTCCCGCTCTTTAATGTCGTAGGGACTTCACCTGTTCGACTTTCGTTTCAAGTATCGTCCCTCTTAGTA
        895         900         905         910         915         920
Ile Ala Gly Leu Ala Asn Met Arg Ala Arg Asn Tyr Ser Ile Pro Glu Val Asp Lys Leu Lys Ala Lys Phe Ile Ala Gly Arg Ile Ile
AtUBA1

CCCAGCCATTGCGACTTCAACAGCCATGGCTACTGGGCTGGTCTGCCTCGAGCTGTACAAGGTCCTTGATGGAGGACATAAAGTGGAAGC   3240
GGGTCGGTAACGCTGAAGTTGTCGGTACCGATGACCCGACCAGACGGAGCTCGACATGTTCCAGGAACTACCTCCTGTATTTCACCTTCG
        925         930         935         940         945         950
Pro Ala Ile Ala Thr Ser Thr Ala Met Ala Thr Gly Leu Val Cys Leu Glu Leu Tyr Lys Val Leu Asp Gly Gly His Lys Val Glu Ala
AtUBA1

TTACAGAAACACATTTGCCAACCTGGCACTTCCACTATTCTCCATGGCTGAACCACTTCCACCAAAGGTGGTGAAGCACCGTGACATGGC   3330
AATGTCTTTGTGTAAACGGTTGGACCGTGAAGGTGATAAGAGGTACCGACTTGGTGAAGGTGGTTTCCACCACTTCGTGGCACTGTACCG
        955         960         965         970         975         980
Tyr Arg Asn Thr Phe Ala Asn Leu Ala Leu Pro Leu Phe Ser Met Ala Glu Pro Leu Pro Pro Lys Val Val Lys His Arg Asp Met Ala
AtUBA1

TTGGACAGTTTGGGACAGATGGGTTCTCAAGGGAAACCCAACTCTTCGTGAGGTGTTGCAGTGGCTGGAGGACAAAGGGCTTAGCGCATA   3420
AACCTGTCAAACCCTGTCTACCCAAGAGTTCCCTTTGGGTTGAGAAGCACTCCACAACGTCACCGACCTCCTGTTTCCCGAATCGCGTAT
        985         990         995         1000        1005        1010
Trp Thr Val Trp Asp Arg Trp Val Leu Lys Gly Asn Pro Thr Leu Arg Glu Val Leu Gln Trp Leu Glu Asp Lys Gly Leu Ser Ala Tyr
AtUBA1

CAGCATCTCTTGTGGAAGCTGTCTTCTGTTCAACAGTATGTTCACAAGGCACAAGGAGAGGATGGACAAGAAGGTTGTGGATCTCGCTCG   3510
GTCGTAGAGAACACCTTCGACAGAAGACAAGTTGTCATACAAGTGTTCCGTGTTCCTCTCCTACCTGTTCTTCCAACACCTAGAGCGAGC
        1015        1020        1025        1030        1035        1040
Ser Ile Ser Cys Gly Ser Cys Leu Leu Phe Asn Ser Met Phe Thr Arg His Lys Glu Arg Met Asp Lys Lys Val Val Asp Leu Ala Arg
AtUBA1

GGATGTTGCGAAAGTCGAGTTACCGCCTTATCGCAACCATCTTGATGTAGTGGTGGCTTGTGAGGATGAAGATGACAATGACGTCGATAT   3600
CCTACAACGCTTTCAGCTCAATGGCGGAATAGCGTTGGTAGAACTACATCACCACCGAACACTCCTACTTCTACTGTTACTGCAGCTATA
        1045        1050        1055        1060        1065        1070
Asp Val Ala Lys Val Glu Leu Pro Pro Tyr Arg Asn His Leu Asp Val Val Val Ala Cys Glu Asp Glu Asp Asp Asn Asp Val Asp Ile
AtUBA1
```

KpnI

TCCTCTCGTCTCTATCTACTTCAGGggtaccctcgagtctggtaaagaaaccgctgctgcgaaatttgaacgccagcacatggactcgtc 3690
AGGAGAGCAGAGATAGATGAAGTCCccatgggagctcagaccatttctttggcgacgacgctttaaacttgcggtcgtgtacctgagcag

1075 1080 1 5 10 15
Pro Leu Val Ser Ile Tyr Phe Arg Gly Thr Leu Glu Ser Gly Lys Glu Thr Ala Ala Ala Lys Phe Glu Arg Gln His Met Asp Ser Ser
AtUBA1 (in frame with AtUBA1) S

AGGAGAGCAGAGATAGATGAAGTCCccatgggagctcaga
UBA1 R

tactagcgcagcttaattaacctaggctgctgccaccgctgagcaataactagcataaccccttggggcctctaaacgggtcttgagggg 3780
atgatcgcgtcgaattaattggatccgacgacggtggcgactcgttattgatcgtattggggaaccccggagatttgcccagaactcccc

Thr Ser Ala Ala *
(in frame with S)
T7 terminator

tttttgctgaaacctcaggcatttgagaagcacacggtcacactgcttccggtagtcaataaaccggtaaaccagcaatagacataagc 3870
aaaaaacgactttggagtccgtaaactcttcgtgtgccagtgtgacgaaggccatcagttatttggccatttggtcgttatctgtattcg

T7 terminator
T7 promoter

ggctatttaacgaccctgccctgaaccgacgaccgggtcatcgtggccggatcttgcggcccctcggcttgaacgaattgttagacatta 3960
ccgataaattgctgggacgggacttggctgctggcccagtagcaccggcctagaacgccggggagccgaacttgcttaacaatctgtaat

lac operator
T7 promoter
*
SmR

tttgccgactaccttggtgatctcgcctttcacgtagtggacaaattcttccaactgatctgcgcgcgaggccaagcgatcttcttcttg 4050
aaacggctgatggaaccactagagcggaaagtgcatcacctgtttaagaaggttgactagacgcgcgctccggttcgctagaagaagaac

260 255 250 245 240 235
Lys Gly Val Val Lys Thr Ile Glu Gly Lys Val Tyr His Val Phe Glu Glu Leu Gln Asp Ala Arg Ser Ala Leu Arg Asp Glu Glu Gln
SmR

tccaagataagcctgtctagcttcaagtatgacgggctgatactgggccggcaggcgctccattgcccagtcggcagcgacatccttcgg 4140
aggttctattcggacagatcgaagttcatactgcccgactatgacccggccgtccgcgaggtaacgggtcagccgtcgctgtaggaagcc

230 225 220 215 210 205
Gly Leu Tyr Ala Gln Arg Ala Glu Leu Ile Val Pro Gln Tyr Gln Ala Pro Leu Arg Glu Met Ala Trp Asp Ala Ala Val Asp Lys Pro
SmR

cgcgattttgccggttactgcgctgtaccaaatgcgggacaacgtaagcactacatttcgctcatcgccagcccagtcgggcggcgagtt 4230
gcgctaaaacggccaatgacgcgacatggtttacgccctgttgcattcgtgatgtaaagcgagtagcggtcgggtcagcccgccgctcaa

200 195 190 185 180 175
Ala Ile Lys Gly Thr Val Ala Ser Tyr Trp Ile Arg Ser Leu Thr Leu Val Val Asn Arg Glu Asp Gly Ala Trp Asp Pro Pro Ser Asn
SmR

ccatagcgttaaggtttcatttagcgcctcaaatagatcctgttcaggaaccggatcaaagagttcctccgccgctggacctaccaaggc 4320
ggtatcgcaattccaaagtaaatcgcggagtttatctaggacaagtccttggcctagtttctcaaggaggcggcgacctggatggttccg

170 165 160 155 150 145
Trp Leu Thr Leu Thr Glu Asn Leu Ala Glu Phe Leu Asp Gln Glu Pro Val Pro Asp Phe Leu Glu Glu Ala Ala Pro Gly Val Leu Ala
SmR

aacgctatgttctcttgcttttgtcagcaagatagccagatcaatgtcgatcgtggctggctcgaagatacctgcaagaatgtcattgcg 4410
ttgcgatacaagagaacgaaaacagtcgttctatcggtctagttacagctagcaccgaccgagcttctatggacgttcttacagtaacgc

140 135 130 125 120 115
Val Ser His Glu Arg Ala Lys Thr Leu Leu Ile Ala Leu Asp Ile Asp Ile Thr Ala Pro Glu Phe Ile Gly Ala Leu Ile Asp Asn Arg
SmR

ctgccattctccaaattgcagttcgcgcttagctggataacgccacggaatgatgtcgtcgtgcacaacaatggtgacttctacagcgcg 4500
gacggtaagaggtttaacgtcaagcgcgaatcgacctattgcggtgccttactacagcagcacgtgttgttaccactgaagatgtcgcgc

110 105 100 95 90 85
Gln Trp Glu Gly Phe Gln Leu Glu Arg Lys Ala Pro Tyr Arg Trp Pro Ile Ile Asp Asp His Val Val Ile Thr Val Glu Val Ala Arg
SmR

gagaatctcgctctctccaggggaagccgaagtttccaaaaggtcgttgatcaaagctcgccgcgttgtttcatcaagccttacggtcac 4590
ctcttagagcgagagaggtccccttcggcttcaaaggttttccagcaactagtttcgagcggcgcaacaaagtagttcggaatgccagtg
80 75 70 65 60 55
Leu Ile Glu Ser Glu Gly Pro Ser Ala Ser Thr Glu Leu Leu Asp Asn Ile Leu Ala Arg Arg Thr Thr Glu Asp Leu Arg Val Thr Val
SmR

cgtaaccagcaaatcaatatcactgtgtggcttcaggccgccatccactgcggagccgtacaaatgtacggccagcaacgtcggttcgag 4680
gcattggtcgtttagttatagtgacacaccgaagtccggcggtaggtgacgcctcggcatgtttacatgccggtcgttgcagccaagctc
50 45 40 35 30 25
Thr Val Leu Leu Asp Ile Asp Ser His Pro Lys Leu Gly Gly Asp Val Ala Ser Gly Tyr Leu His Val Ala Leu Leu Thr Pro Glu Leu
SmR

atggcgctcgatgacgccaactacctctgatagttgagtcgatacttcggcgatcaccgcttccctcatactcttcctttttcaatatta 4770
taccgcgagctactgcggttgatggagactatcaactcagctatgaagccgctagtggcgaagggagtatgagaaggaaaaagttataat
20 15 10 5 1
His Arg Glu Ile Val Gly Val Val Glu Ser Leu Gln Thr Ser Val Glu Ala Ile Val Ala Glu Arg Met
SmR
AmpR promoter

ttgaagcatttatcagggttattgtctcatgagcggatacatatttgaatgtatttagaaaaataaacaaatagctagctcactcggtcg 4860
aacttcgtaaatagtcccaataacagagtactcgcctatgtataaacttacataaatctttttatttgtttatcgatcgagtgagccagc
AmpR promoter

ctacgctccgggcgtgagactgcggcgggcgctgcggacacatacaaagttacccacagattccgtggataagcaggggactaacatgtg 4950
gatgcgaggcccgcactctgacgccgcccgcgacgcctgtgtatgtttcaatgggtgtctaaggcacctattcgtcccctgattgtacac
CloDF13 ori

aggcaaaacagcagggccgcgccggtggcgtttttccataggctccgccctcctgccagagttcacataaacagacgcttttccggtgca 5040
tccgttttgtcgtcccggcgcggccaccgcaaaaaggtatccgaggcgggaggacggtctcaagtgtatttgtctgcgaaaaggccacgt
CloDF13 ori

tctgtgggagccgtgaggctcaaccatgaatctgacagtacgggcgaaacccgacaggacttaaagatccccaccgtttccggcgggtcg 5130
agacaccctcggcactccgagttggtacttagactgtcatgcccgctttgggctgtcctgaatttctaggggtggcaaaggccgcccagc
CloDF13 ori

ctccctcttgcgctctcctgttccgaccctgccgtttaccggatacctgttccgcctttctcccttacgggaagtgtggcgctttctcat 5220
gagggagaacgcgagaggacaaggctgggacggcaaatggcctatggacaaggcggaaagagggaatgcccttcacaccgcgaaagagta
CloDF13 ori

agctcacacactggtatctcggctcggtgtaggtcgttcgctccaagctgggctgtaagcaagaactccccgttcagcccgactgctgcg 5310
tcgagtgtgtgaccatagagccgagccacatccagcaagcgaggttcgacccgacattcgttcttgaggggcaagtcgggctgacgacgc
CloDF13 ori

ccttatccggtaactgttcacttgagtccaacccggaaaagcacggtaaaacgccactggcagcagccattggtaactgggagttcgcag 5400
ggaataggccattgacaagtgaactcaggttgggccttttcgtgccattttgcggtgaccgtcgtcggtaaccattgaccctcaagcgtc
CloDF13 ori

aggatttgtttagctaaacacgcggttgctcttgaagtgtgcgccaaagtccggctacactggaaggacagatttggttgctgtgctctg 5490
tcctaaacaaatcgatttgtgcgccaacgagaacttcacacgcggtttcaggccgatgtgaccttcctgtctaaaccaacgacacgagac
CloDF13 ori

cgaaagccagttaccacggttaagcagttccccaactgacttaaccttcgatcaaaccacctccccaggtggtttttttcgtttacagggc 5580
gctttcggtcaatggtgccaattcgtcaaggggttgactgaattggaagctagtttggtggaggggtccaccaaaaaaagcaaatgtcccg
CloDF13 ori

aaaagattacgcgcagaaaaaaaggatctcaagaagatcctttgatcttttctactgaaccgctctagatttcagtgcaatttatctctt 5670
ttttctaatgcgcgtctttttttcctagagttcttctaggaaactagaaaagatgacttggcgagatctaaagtcacgttaaatagagaa
CloDF13 ori

caaatgtagcacctgaagtcagccccatacgatataagttgtaattctcatgttagtcatgccccgcgcccaccggaaggagctgactgg 5760
gtttacatcgtggacttcagtcggggtatgctatattcaacattaagagtacaatcagtacggggcgcgggtggccttcctcgactgacc

gttgaaggctctcaagggcatcggtcgagatcccggtgcctaatgagtgagctaacttacattaattgcgttgcgctcactgcccgcttt 5850
caacttccgagagttcccgtagccagctctagggccacggattactcactcgattgaatgtaattaacgcaacgcgagtgacgggcgaaa
360
* Gln Gly Ser Glu
lacI

ccagtcgggaaacctgtcgtgccagctgcattaatgaatcggccaacgcgcggggagaggcggtttgcgtattgggcgccagggtggttt 5940
ggtcagccctttggacagcacggtcgacgtaattacttagccggttgcgcgcccctctccgccaaacgcataacccgcggtcccaccaaa
355 350 345 340 335 330
Leu Arg Ser Val Gln Arg Ala Leu Gln Met Leu Ser Asp Ala Leu Ala Arg Pro Ser Ala Thr Gln Thr Asn Pro Ala Leu Thr Thr Lys
lacI

ttcttttcaccagtgagacgggcaacagctgattgcccttcaccgcctggccctgagagagttgcagcaagcggtccacgctggtttgcc 6030
aagaaaagtggtcactctgcccgttgtcgactaacgggaagtggcggaccgggactctctcaacgtcgttcgccaggtgcgaccaaacgg
325 320 315 310 305 300
Arg Lys Val Leu Ser Val Pro Leu Leu Gln Asn Gly Lys Val Ala Gln Gly Gln Ser Leu Gln Leu Leu Arg Asp Val Ser Thr Gln Gly
lacI

ccagcaggcgaaaatcctgtttgatggtggttaacggcgggatataacatgagctgtcttcggtatcgtcgtatcccactaccgagatgt 6120
ggtcgtccgcttttaggacaaactaccaccaattgccgccctatattgtactcgacagaagccatagcagcatagggtgatggctctaca
295 290 285 280 275 270
Leu Leu Arg Phe Asp Gln Lys Ile Thr Thr Leu Pro Pro Ile Tyr Cys Ser Ser Asp Glu Thr Asp Asp Tyr Gly Val Val Ser Ile Asp
lacI

ccgcaccaacgcgcagcccggactcggtaatggcgcgcattgcgcccagcgccatctgatcgttggcaaccagcatcgcagtgggaacga 6210
ggcgtggttgcgcgtcgggcctgagccattaccgcgcgtaacgcgggtcgcggtagactagcaaccgttggtcgtagcgtcacccttgct
265 260 255 250 245 240
Ala Gly Val Arg Leu Gly Ser Glu Thr Ile Ala Arg Met Ala Gly Leu Ala Met Gln Asp Asn Ala Val Leu Met Ala Thr Pro Val Ile
lacI

tgccctcattcagcatttgcatggtttgttgaaaaccggacatggcactccagtcgccttcccgttccgctatcggctgaatttgattgc 6300
acgggagtaagtcgtaaacgtaccaaacaacttttggcctgtaccgtgaggtcagcggaagggcaaggcgatagccgacttaaactaacg
235 230 225 220 215 210
Gly Glu Asn Leu Met Gln Met Thr Gln Gln Phe Gly Ser Met Ala Ser Trp Asp Gly Glu Arg Glu Ala Ile Pro Gln Ile Gln Asn Arg
lacI

gagtgagatatttatgccagccagccagacgcagacgcgccgagacagaacttaatgggcccgctaacagcgcgatttgctggtgaccca 6390
ctcactctataaatacggtcggtcggtctgcgtctgcgcggctctgtcttgaattacccgggcgattgtcgcgctaaacgaccactgggt
205 200 195 190 185 180
Thr Leu Tyr Lys His Trp Gly Ala Leu Arg Leu Arg Ala Ser Val Ser Ser Leu Pro Gly Ala Leu Leu Ala Ile Gln Gln His Gly Leu
lacI

atgcgaccagatgctccacgcccagtcgcgtaccgtcttcatgggagaaaataatactgttgatgggtgtctggtcagagacatcaagaa 6480
tacgctggtctacgaggtgcgggtcagcgcatggcagaagtaccctcttttattatgacaactacccacagaccagtctctgtagttctt
175 170 165 160 155 150
Ala Val Leu His Glu Val Gly Leu Arg Thr Gly Asp Glu His Ser Phe Ile Ile Ser Asn Ile Pro Thr Gln Asp Ser Val Asp Leu Phe
lacI

ataacgccggaacattagtgcaggcagcttccacagcaatggcatcctggtcatccagcggatagttaatgatcagcccactgacgcgtt 6570
tattgcggccttgtaatcacgtccgtcgaaggtgtcgttaccgtaggaccagtaggtcgcctatcaattactagtcgggtgactgcgcaa
145 140 135 130 125 120
Leu Ala Pro Val Asn Thr Cys Ala Ala Glu Val Ala Ile Ala Asp Gln Asp Asp Leu Pro Tyr Asn Ile Ile Leu Gly Ser Val Arg Gln
lacI

gcgcgagaagattgtgcaccgccgctttacaggcttcgacgccgcttcgttctaccatcgacaccaccacgctggcacccagttgatcgg 6660
cgcgctcttctaacacgtggcggcgaaatgtccgaagctgcggcgaagcaagatggtagctgtggtggtgcgaccgtgggtcaactagcc

115 110 105 100 95 90
Ala Leu Leu Asn His Val Ala Ala Lys Cys Ala Glu Val Gly Ser Arg Glu Val Met Ser Val Val Val Ser Ala Gly Leu Gln Asp Ala
lacI

cgcgagatttaatcgccgcgacaatttgcgacggcgcgtgcagggccagactggaggtggcaacgccaatcagcaacgactgtttgcccg 6750
gcgctctaaattagcggcgctgttaaacgctgccgcgcacgtcccggtctgacctccaccgttgcggttagtcgttgctgacaaacgggc

85 80 75 70 65 60
Arg Ser Lys Ile Ala Ala Val Ile Gln Ser Pro Ala His Leu Ala Leu Ser Ser Thr Ala Val Gly Ile Leu Leu Ser Gln Lys Gly Ala
lacI

ccagttgttgtgccacgcggttgggaatgtaattcagctccgccatcgccgcttccacttttttcccgcgttttcgcagaaacgtggctgg 6840
ggtcaacaacacggtgcgccaacccttacattaagtcgaggcggtagcggcgaaggtgaaaaagggcgcaaaagcgtctttgcaccgacc

55 50 45 40 35 30
Leu Gln Gln Ala Val Arg Asn Pro Ile Tyr Asn Leu Glu Ala Met Ala Ala Glu Val Lys Glu Arg Thr Lys Ala Ser Val His Ser Ala
lacI

cctggttcaccacgcgggaaacggtctgataagagacaccggcatactctgcgacatcgtataacgttactggtttcacattcaccaccc 6930
ggaccaagtggtgcgccctttgccagactattctctgtggccgtatgagacgctgtagcatattgcaatgaccaaagtgtaagtggtggg

25 20 15 10 5 1
Gln Asn Val Val Arg Ser Val Thr Gln Tyr Ser Val Gly Ala Tyr Glu Ala Val Asp Tyr Leu Thr Val Pro Lys Met
lacI
lacI promoter

tgaattgactctcttccgggcgctatcatgccataccgcgaaaggttttgcgccattcgatggtgtcc 3′
••• 6998
acttaactgagagaaggcccgcgatagtacggtatggcgctttccaaaacgcggtaagctaccacagg 5′

lacI promoter

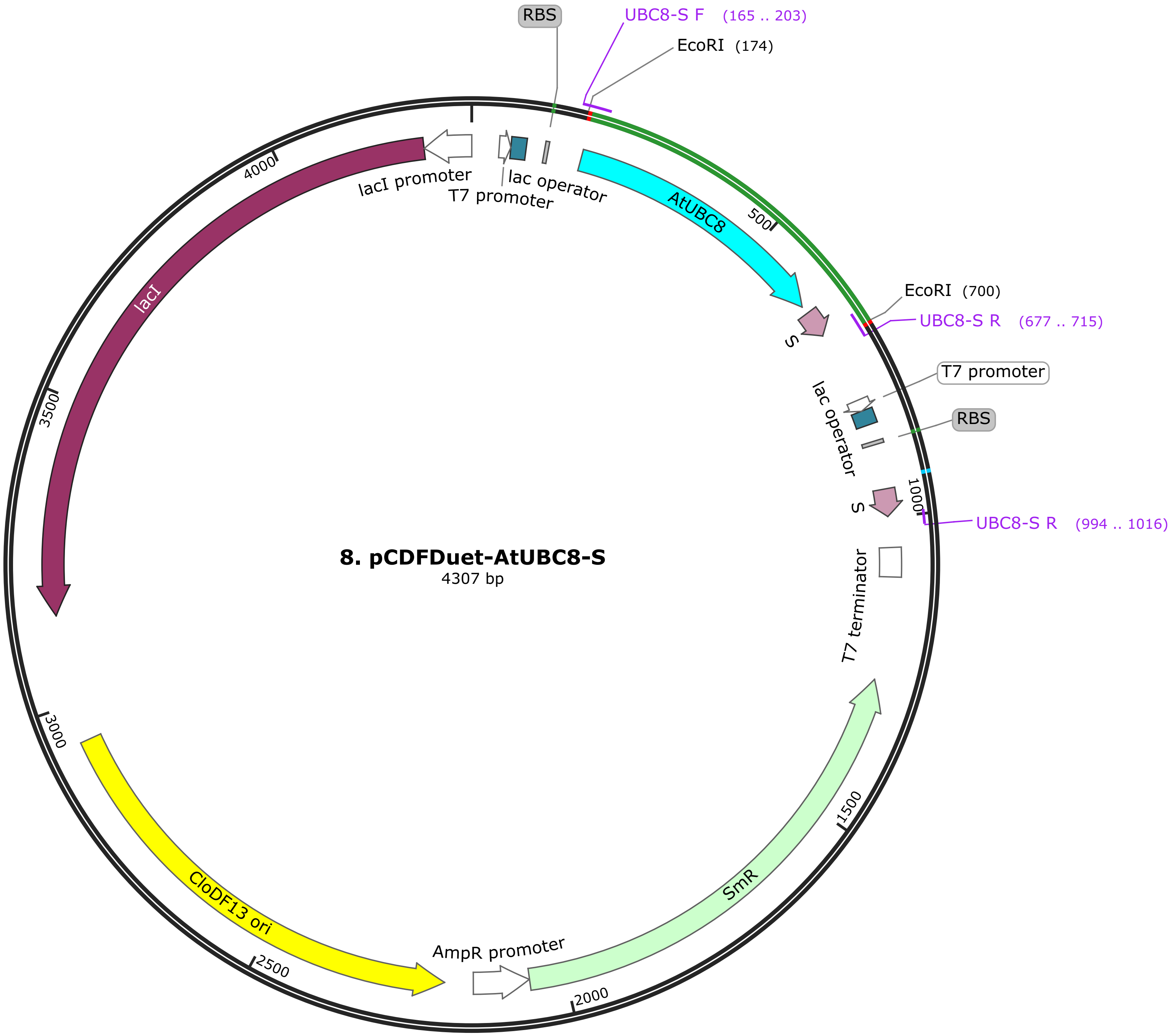

Created with SnapGene®
RBS
UBC8-S F (165 .. 203)
EcoRI (174)
EcoRI (700)
UBC8-S R (677 .. 715)
T7 promoter
RBS
UBC8-S R (994 .. 1016)
lacI promoter
T7 promoter
lac operator
AtUBC8
S
lac operator
S
T7 terminator
lacI
8. pCDFDuet-AtUBC8-S
4307 bp
CloDF13 ori
AmpR promoter
SmR
500
1000
1500
2000
2500
3000
3500
4000

5′ cgggatctcgacgctctcccttatgcgactcctgcattaggaaattaatacgactcactataggggaattgtgagcggataacaattccc 90
3′ gccctagagctgcgagagggaatacgctgaggacgtaatcctttaattatgctgagtgatatccccttaacactcgcctattgttaaggg
T7 promoter · lac operator

EcoRI
UBC8-S F
ccaggatccgaattcC
ctgtagaaataattttgtttaactttaatAAGGAGatataccatgggcagcagccatcaccatcatcaccacagccaggatccgaattcC 180
gacatctttattaaaacaaattgaaattaTTCCTCtatatggtacccgtcgtcggtagtggtagtagtggtgtcggtcctaggcttaagG
RBS

UBC8-S F
ATGGCTTCGAAACGGATCTTGAA
ATGGCTTCGAAACGGATCTTGAAGGAGCTGAAGGATCTCCAGAAAGACCCTCCAACCTCCTGCAGTGCAGGTCCAGTTGCTGAAGACATG 270
TACCGAAGCTTTGCCTAGAACTTCCTCGACTTCCTAGAGGTCTTTCTGGGAGGTTGGAGGACGTCACGTCCAGGTCAACGACTTCTGTAC
1 · 5 · 10 · 15 · 20 · 25 · 30
Met Ala Ser Lys Arg Ile Leu Lys Glu Leu Lys Asp Leu Gln Lys Asp Pro Pro Thr Ser Cys Ser Ala Gly Pro Val Ala Glu Asp Met
AtUBC8

TTTCATTGGCAAGCTACAATTATGGGTCCTGCAGAGAGTCCGTATTCAGGCGGTGTGTTTCTCGTTACCATTCACTTCCCTCCGGACTAT 360
AAAGTAACCGTTCGATGTTAATACCCAGGACGTCTCTCAGGCATAAGTCCGCCACACAAAGAGCAATGGTAAGTGAAGGGAGGCCTGATA
35 · 40 · 45 · 50 · 55 · 60
Phe His Trp Gln Ala Thr Ile Met Gly Pro Ala Glu Ser Pro Tyr Ser Gly Gly Val Phe Leu Val Thr Ile His Phe Pro Pro Asp Tyr
AtUBC8

CCATTCAAACCACCAAAGGTTGCATTTAGGACGAAGGTGTTTCACCCTAATATCAACAGCAACGGAAGCATTTGCCTTGACATTTTGAAA 450
GGTAAGTTTGGTGGTTTCCAACGTAAATCCTGCTTCCACAAAGTGGGATTATAGTTGTCGTTGCCTTCGTAAACGGAACTGTAAAACTTT
65 · 70 · 75 · 80 · 85 · 90
Pro Phe Lys Pro Pro Lys Val Ala Phe Arg Thr Lys Val Phe His Pro Asn Ile Asn Ser Asn Gly Ser Ile Cys Leu Asp Ile Leu Lys
AtUBC8

GAACAATGGAGCCCTGCCCTCACCATTTCCAAGGTGTTGCTCTCGATATGTTCGCTGTTAACAGATCCAAATCCAGATGACCCTTTGGTA 540
CTTGTTACCTCGGGACGGGAGTGGTAAAGGTTCCACAACGAGAGCTATACAAGCGACAATTGTCTAGGTTTAGGTCTACTGGGAAACCAT
95 · 100 · 105 · 110 · 115 · 120
Glu Gln Trp Ser Pro Ala Leu Thr Ile Ser Lys Val Leu Leu Ser Ile Cys Ser Leu Leu Thr Asp Pro Asn Pro Asp Asp Pro Leu Val
AtUBC8

CCAGAGATTGCACACATGTACAAAACCGACAGAGCCAAATACGAGGCTACTGCAAGAAACTGGACTCAGAAGTATGCCATGGGcctcgag 630
GGTCTCTAACGTGTGTACATGTTTTGGCTGTCTCGGTTTATGCTCCGATGACGTTCTTTGACCTGAGTCTTCATACGGTACCCggagctc
125 · 130 · 135 · 140 · 145
Pro Glu Ile Ala His Met Tyr Lys Thr Asp Arg Ala Lys Tyr Glu Ala Thr Ala Arg Asn Trp Thr Gln Lys Tyr Ala Met Gly Leu Glu
AtUBC8

EcoRI
tctggtaaagaaaccgctgctgcgaaatttgaacgccagcacatggactcgtctactagcgcagcttaagaattcgagctcggcgcgcct 720
agaccatttctttggcgacgacgctttaaacttgcggtcgtgtacctgagcagatgatcgcgtcgaattcttaagctcgagccgcgcgga
1 · 5 · 10 · 15
Ser Gly Lys Glu Thr Ala Ala Ala Lys Phe Glu Arg Gln His Met Asp Ser Ser Thr Ser Ala Ala
S
(in frame with S)
tgagcagatgatcgcgtcgaattcttaagctcgagccgc
UBC8-S R

gcaggtcgacaagcttgcggccgcataatgcttaagtcgaacagaaagtaatcgtattgtacacggccgcataatcgaaattaatacgac 810
cgtccagctgttcgaacgccggcgtattacgaattcagcttgtctttcattagcataacatgtgccggcgtattagctttaattatgctg
T7 promoter

tcactataggggaattgtgagcggataacaattccccatcttagtatattagttaagtataagAAGGAGatatacatatggcagatctca 900
agtgatatccccttaacactcgcctattgttaaggggtagaatcatataatcaattcatattcTTCCTCtatatgtataccgtctagagt
T7 promoter · lac operator · RBS

attggatatcggccggccacgcgatcgctgacgtcggtacccctcgagtctggtaaagaaaccgctgctgcgaaatttgaacgccagcaca 990
taacctatagccggccggtgcgctagcgactgcagccatgggagctcagaccatttctttggcgacgacgctttaaacttgcggtcgtgt

1 5 10
Lys Glu Thr Ala Ala Ala Lys Phe Glu Arg Gln His
S

tggactcgtctactagcgcagcttaattaacctaggctgctgccaccgctgagcaataactagcataaccccttggggcctctaaacggg 1080
acctgagcagatgatcgcgtcgaattaattggatccgacgacggtggcgactcgttattgatcgtattggggaaccccggagatttgccc

15
Met Asp Ser Ser Thr Ser Ala Ala *
S (in frame with S)
T7 terminator

tgagcagatgatcgcgtcgaatt cttaagctcgagccgc
UBC8-S R

tcttgaggggttttttgctgaaacctcaggcatttgagaagcacacggtcacactgcttccggtagtcaataaaccggtaaaccagcaat 1170
agaactccccaaaaaacgactttggagtccgtaaactcttcgtgtgccagtgtgacgaaggccatcagttatttggccatttggtcgtta

T7 terminator

agacataagcggctatttaacgaccctgccctgaaccgacgaccgggtcatcgtggccggatcttgcggcccctcggcttgaacgaattg 1260
tctgtattcgccgataaattgctgggacgggacttggctgctggcccagtagcaccggcctagaacgccggggagccgaacttgcttaac

ttagacattatttgccgactaccttggtgatctcgcctttcacgtagtggacaaattcttccaactgatctgcgcgcgaggccaagcgat 1350
aatctgtaataaacggctgatggaaccactagagcggaaagtgcatcacctgtttaagaaggttgactagacgcgcgctccggttcgcta

260 255 250 245 240
* Lys Gly Val Val Lys Thr Ile Glu Gly Lys Val Tyr His Val Phe Glu Glu Leu Gln Asp Ala Arg Ser Ala Leu Arg Asp
SmR

cttcttcttgtccaagataagcctgtctagcttcaagtatgacgggctgatactgggccggcaggcgctccattgcccagtcggcagcga 1440
gaagaagaacaggttctattcggacagatcgaagttcatactgcccgactatgacccggccgtccgcgaggtaacgggtcagccgtcgct

235 230 225 220 215 210
Glu Glu Gln Gly Leu Tyr Ala Gln Arg Ala Glu Leu Ile Val Pro Gln Tyr Gln Ala Pro Leu Arg Glu Met Ala Trp Asp Ala Ala Val
SmR

catccttcggcgcgattttgccggttactgcgctgtaccaaatgcgggacaacgtaagcactacatttcgctcatcgccagcccagtcgg 1530
gtaggaagccgcgctaaaacggccaatgacgcgacatggtttacgccctgttgcattcgtgatgtaaagcgagtagcggtcgggtcagcc

205 200 195 190 185 180
Asp Lys Pro Ala Ile Lys Gly Thr Val Ala Ser Tyr Trp Ile Arg Ser Leu Thr Leu Val Val Asn Arg Glu Asp Gly Ala Trp Asp Pro
SmR

gcggcgagttccatagcgttaaggtttcatttagcgcctcaaatagatcctgttcaggaaccggatcaaagagttcctccgccgctggac 1620
cgccgctcaaggtatcgcaattccaaagtaaatcgcggagtttatctaggacaagtccttggcctagtttctcaaggaggcggcgacctg

175 170 165 160 155 150
Pro Ser Asn Trp Leu Thr Leu Thr Glu Asn Leu Ala Glu Phe Leu Asp Gln Glu Pro Val Pro Asp Phe Leu Glu Glu Ala Ala Pro Gly
SmR

ctaccaaggcaacgctatgttctcttgcttttgtcagcaagatagccagatcaatgtcgatcgtggctggctcgaagatacctgcaagaa 1710
gatggttccgttgcgatacaagagaacgaaaacagtcgttctatcggtctagttacagctagcaccgaccgagcttctatggacgttctt

145 140 135 130 125 120
Val Leu Ala Val Ser His Glu Arg Ala Lys Thr Leu Leu Ile Ala Leu Asp Ile Asp Ile Thr Ala Pro Glu Phe Ile Gly Ala Leu Ile
SmR

tgtcattgcgctgccattctccaaattgcagttcgcgcttagctggataacgccacggaatgatgtcgtcgtgcacaacaatggtgactt 1800
acagtaacgcgacggtaagaggtttaacgtcaagcgcgaatcgacctattgcggtgccttactacagcagcacgtgttgttaccactgaa

115 110 105 100 95 90
Asp Asn Arg Gln Trp Glu Gly Phe Gln Leu Glu Arg Lys Ala Pro Tyr Arg Trp Pro Ile Ile Asp Asp His Val Val Ile Thr Val Glu
SmR

ctacagcgcggagaatctcgctctctccaggggaagccgaagtttccaaaaggtcgttgatcaaagctcgccgcgttgtttcatcaagcc 1890
gatgtcgcgcctcttagagcgagagaggtccccttcggcttcaaaggttttccagcaactagtttcgagcggcgcaacaaagtagttcgg

85 80 75 70 65 60
Val Ala Arg Leu Ile Glu Ser Glu Gly Pro Ser Ala Ser Thr Glu Leu Leu Asp Asn Ile Leu Ala Arg Arg Thr Thr Glu Asp Leu Arg
SmR

```
ttacggtcaccgtaaccagcaaatcaatatcactgtgtggcttcaggccgccatccactgcggagccgtacaaatgtacggccagcaacg   1980
aatgccagtggcattggtcgtttagttatagtgacacaccgaagtccggcggtaggtgacgcctcggcatgtttacatgccggtcgttgc
     55              50              45              40              35              30
Val Thr Val Thr Val Leu Leu Asp Ile Asp Ser His Pro Lys Leu Gly Gly Asp Val Ala Ser Gly Tyr Leu His Val Ala Leu Leu Thr
< SmR

tcggttcgagatggcgctcgatgacgccaactacctctgatagttgagtcgatacttcggcgatcaccgcttccctcatactcttccttt   2070
agccaagctctaccgcgagctactgcggttgatggagactatcaactcagctatgaagccgctagtggcgaagggagtatgagaaggaaa
     25              20              15              10              5           1
Pro Glu Leu His Arg Glu Ile Val Gly Val Val Glu Ser Leu Gln Thr Ser Val Glu Ala Ile Val Ala Glu Arg Met
< SmR
< AmpR promoter

ttcaatattattgaagcatttatcagggttattgtctcatgagcggatacatatttgaatgtatttagaaaaataaacaaatagctagct   2160
aagttataataacttcgtaaatagtcccaataacagagtactcgcctatgtataaacttacataaatctttttatttgtttatcgatcga
< AmpR promoter

cactcggtcgctacgctccgggcgtgagactgcggcgggcgctgcggacacatacaaagttacccacagattccgtggataagcagggga   2250
gtgagccagcgatgcgaggcccgcactctgacgccgcccgcgacgcctgtgtatgtttcaatgggtgtctaaggcacctattcgtcccct
< CloDF13 ori

ctaacatgtgaggcaaaacagcagggccgcgccggtggcgtttttccataggctccgccctcctgccagagttcacataaacagacgctt   2340
gattgtacactccgttttgtcgtcccggcgcggccaccgcaaaaaggtatccgaggcgggaggacggtctcaagtgtatttgtctgcgaa
< CloDF13 ori

ttccggtgcatctgtgggagccgtgaggctcaaccatgaatctgacagtacgggcgaaacccgacaggacttaaagatccccaccgtttc   2430
aaggccacgtagacaccctcggcactccgagttggtacttagactgtcatgcccgctttgggctgtcctgaatttctaggggtggcaaag
< CloDF13 ori

cggcgggtcgctccctcttgcgctctcctgttccgaccctgccgtttaccggatacctgttccgcctttctcccttacgggaagtgtggc   2520
gccgcccagcgagggagaacgcgagaggacaaggctgggacggcaaatggcctatggacaaggcggaaagagggaatgcccttcacaccg
< CloDF13 ori

gctttctcatagctcacacactggtatctcggctcggtgtaggtcgttcgctccaagctgggctgtaagcaagaactccccgttcagccc   2610
cgaaagagtatcgagtgtgtgaccatagagccgagccacatccagcaagcgaggttcgacccgacattcgttcttgaggggcaagtcggg
< CloDF13 ori

gactgctgcgccttatccggtaactgttcacttgagtccaacccggaaaagcacggtaaaacgccactggcagcagccattggtaactgg   2700
ctgacgacgcggaataggccattgacaagtgaactcaggttgggccttttcgtgccattttgcggtgaccgtcgtcggtaaccattgacc
< CloDF13 ori

gagttcgcagaggatttgtttagctaaacacgcggttgctcttgaagtgtgcgccaaagtccggctacactggaaggacagatttggttg   2790
ctcaagcgtctcctaaacaaatcgatttgtgcgccaacgagaacttcacacgcggtttcaggccgatgtgaccttcctgtctaaaccaac
< CloDF13 ori

ctgtgctctgcgaaagccagttaccacggttaagcagttccccaactgacttaaccttcgatcaaaccacctccccaggtggtttttcg   2880
gacacgagacgctttcggtcaatggtgccaattcgtcaaggggttgactgaattggaagctagtttggtggaggggtccaccaaaaaagc
< CloDF13 ori

tttacagggcaaaagattacgcgcagaaaaaaaggatctcaagaagatcctttgatcttttctactgaaccgctctagatttcagtgcaa   2970
aaatgtcccgttttctaatgcgcgtcttttttttcctagagttcttctaggaaactagaaaagatgacttggcgagatctaaagtcacgtt
< CloDF13 ori

tttatctcttcaaatgtagcacctgaagtcagccccatacgatataagttgtaattctcatgttagtcatgccccgcgcccaccggaagg   3060
aaatagagaagtttacatcgtggacttcagtcggggtatgctatattcaacattaagagtacaatcagtacggggcgcgggtggccttcc
```

agctgactgggttgaaggctctcaagggcatcggtcgagatcccggtgcctaatgagtgagctaacttacattaattgcgttgcgctcac 3150

tcgactgacccaacttccgagagttcccgtagccagctctagggccacggattactcactcgattgaatgtaattaacgcaacgcgagtg

lacI

tgcccgctttccagtcgggaaacctgtcgtgccagctgcattaatgaatcggccaacgcgcggggagaggcggtttgcgtattgggcgcc 3240

acgggcgaaaggtcagccctttggacagcacggtcgacgtaattacttagccggttgcgcgcccctctccgccaaacgcataacccgcgg

360 Gln Gly Ser Glu Leu 355 Arg Ser Val Gln Arg 350 Ala Leu Gln Met Leu 345 Ser Asp Ala Leu Ala 340 Arg Pro Ser Ala Thr 335 Gln Thr Asn Pro Ala

lacI

agggtggtttttcttttcaccagtgagacgggcaacagctgattgcccttcaccgcctggccctgagagagttgcagcaagcggtccacg 3330

tcccaccaaaaagaaaagtggtcactctgcccgttgtcgactaacgggaagtggcggaccgggactctctcaacgtcgttcgccaggtgc

330 Leu Thr Thr Lys Arg 325 Lys Val Leu Ser Val 320 Pro Leu Leu Gln Asn 315 Gly Lys Val Ala Gln 310 Gly Gln Ser Leu Gln 305 Leu Leu Arg Asp Val

lacI

ctggtttgccccagcaggcgaaaatcctgtttgatggtggttaacggcgggatataacatgagctgtcttcggtatcgtcgtatcccact 3420

gaccaaacggggtcgtccgcttttaggacaaactaccaccaattgccgccctatattgtactcgacagaagccatagcagcatagggtga

300 Ser Thr Gln Gly Leu 295 Leu Arg Phe Asp Gln 290 Lys Ile Thr Thr Leu 285 Pro Pro Ile Tyr Cys 280 Ser Ser Asp Glu Thr 275 Asp Asp Tyr Gly Val

lacI

accgagatgtccgcaccaacgcgcagcccggactcggtaatggcgcgcattgcgcccagcgccatctgatcgttggcaaccagcatcgca 3510

tggctctacaggcgtggttgcgcgtcgggcctgagccattaccgcgcgtaacgcgggtcgcggtagactagcaaccgttggtcgtagcgt

270 Val Ser Ile Asp Ala 265 Gly Val Arg Leu Gly 260 Ser Glu Thr Ile Ala 255 Arg Met Ala Gly Leu 250 Ala Met Gln Asp Asn 245 Ala Val Leu Met Ala

lacI

gtgggaacgatgccctcattcagcatttgcatggtttgttgaaaaccggacatggcactccagtcgccttcccgttccgctatcggctga 3600

cacccttgctacgggagtaagtcgtaaacgtaccaaacaactttggcctgtaccgtgaggtcagcggaagggcaaggcgatagccgact

240 Thr Pro Val Ile Gly 235 Glu Asn Leu Met Gln 230 Met Thr Gln Gln Phe 225 Gly Ser Met Ala Ser 220 Trp Asp Gly Glu Arg 215 Glu Ala Ile Pro Gln

lacI

atttgattgcgagtgagatatttatgccagccagccagacgcagacgcgccgagacagaacttaatgggcccgctaacagcgcgatttgc 3690

taaactaacgctcactctataaatacggtcggtcggtctgcgtctgcgcggctctgtcttgaattacccgggcgattgtcgcgctaaacg

210 Ile Gln Asn Arg Thr 205 Leu Tyr Lys His Trp 200 Gly Ala Leu Arg Leu 195 Arg Ala Ser Val Ser 190 Ser Leu Pro Gly Ala 185 Leu Leu Ala Ile Gln

lacI

tggtgacccaatgcgaccagatgctccacgcccagtcgcgtaccgtcttcatgggagaaaataatactgttgatgggtgtctggtcagag 3780

accactgggttacgctggtctacgaggtgcgggtcagcgcatggcagaagtaccctcttttattatgacaactacccacagaccagtctc

180 Gln His Gly Leu Ala 175 Val Leu His Glu Val 170 Gly Leu Arg Thr Gly 165 Asp Glu His Ser Phe 160 Ile Ile Ser Asn Ile 155 Pro Thr Gln Asp Ser

lacI

acatcaagaaataacgccggaacattagtgcaggcagcttccacagcaatggcatcctggtcatccagcggatagttaatgatcagccca 3870

tgtagttctttattgcggccttgtaatcacgtccgtcgaaggtgtcgttaccgtaggaccagtaggtcgcctatcaattactagtcgggt

150 Val Asp Leu Phe Leu 145 Ala Pro Val Asn Thr 140 Cys Ala Ala Glu Val 135 Ala Ile Ala Asp Gln 130 Asp Asp Leu Pro Tyr 125 Asn Ile Ile Leu Gly

lacI

ctgacgcgttgcgcgagaagattgtgcaccgccgctttacaggcttcgacgccgcttcgttctaccatcgacaccaccacgctggcaccc 3960

gactgcgcaacgcgctcttctaacacgtggcggcgaaatgtccgaagctgcggcgaagcaagatggtagctgtggtggtgcgaccgtggg

120 Ser Val Arg Gln Ala 115 Leu Leu Asn His Val 110 Ala Ala Lys Cys Ala 105 Glu Val Gly Ser Arg 100 Glu Val Met Ser Val 95 Val Val Ser Ala Gly

lacI

```
agttgatcggcgcgagatttaatcgccgcgacaatttgcgacggcgcgtgcagggccagactggaggtggcaacgccaatcagcaacgac    4050
tcaactagccgcgctctaaattagcggcgctgttaaacgctgccgcgcacgtcccggtctgacctccaccgttgcggttagtcgttgctg
90                  85                  80                  75                  70                  65
Leu Gln Asp Ala Arg Ser Lys Ile Ala Ala Val Ile Gln Ser Pro Ala His Leu Ala Leu Ser Ser Thr Ala Val Gly Ile Leu Leu Ser
< lacI

tgtttgcccgccagttgttgtgccacgcggttgggaatgtaattcagctccgccatcgccgcttccactttttcccgcgttttcgcagaa    4140
acaaacgggcggtcaacaacacggtgcgccaacccttacattaagtcgaggcggtagcggcgaaggtgaaaaagggcgcaaaagcgtctt
60                  55                  50                  45                  40                  35
Gln Lys Gly Ala Leu Gln Gln Ala Val Arg Asn Pro Ile Tyr Asn Leu Glu Ala Met Ala Ala Glu Val Lys Glu Arg Thr Lys Ala Ser
< lacI

acgtggctggcctggttcaccacgcgggaaacggtctgataagagacaccggcatactctgcgacatcgtataacgttactggtttcaca    4230
tgcaccgaccggaccaagtggtgcgccctttgccagactattctctgtggccgtatgagacgctgtagcatattgcaatgaccaaagtgt
30                  25                  20                  15                  10                  5                   1
Val His Ser Ala Gln Asn Val Val Arg Ser Val Thr Gln Tyr Ser Val Gly Ala Tyr Glu Ala Val Asp Tyr Leu Thr Val Pro Lys Met
< lacI                                                                                                  < lacI promoter

ttcaccaccctgaattgactctcttccgggcgctatcatgccataccgcgaaaggttttgcgccattcgatggtgtc    3′
                                                                              ••• 4307
aagtggtgggacttaactgagagaaggcccgcgatagtacggtatggcgctttccaaaacgcggtaagctaccacag    5′
< lacI promoter
```

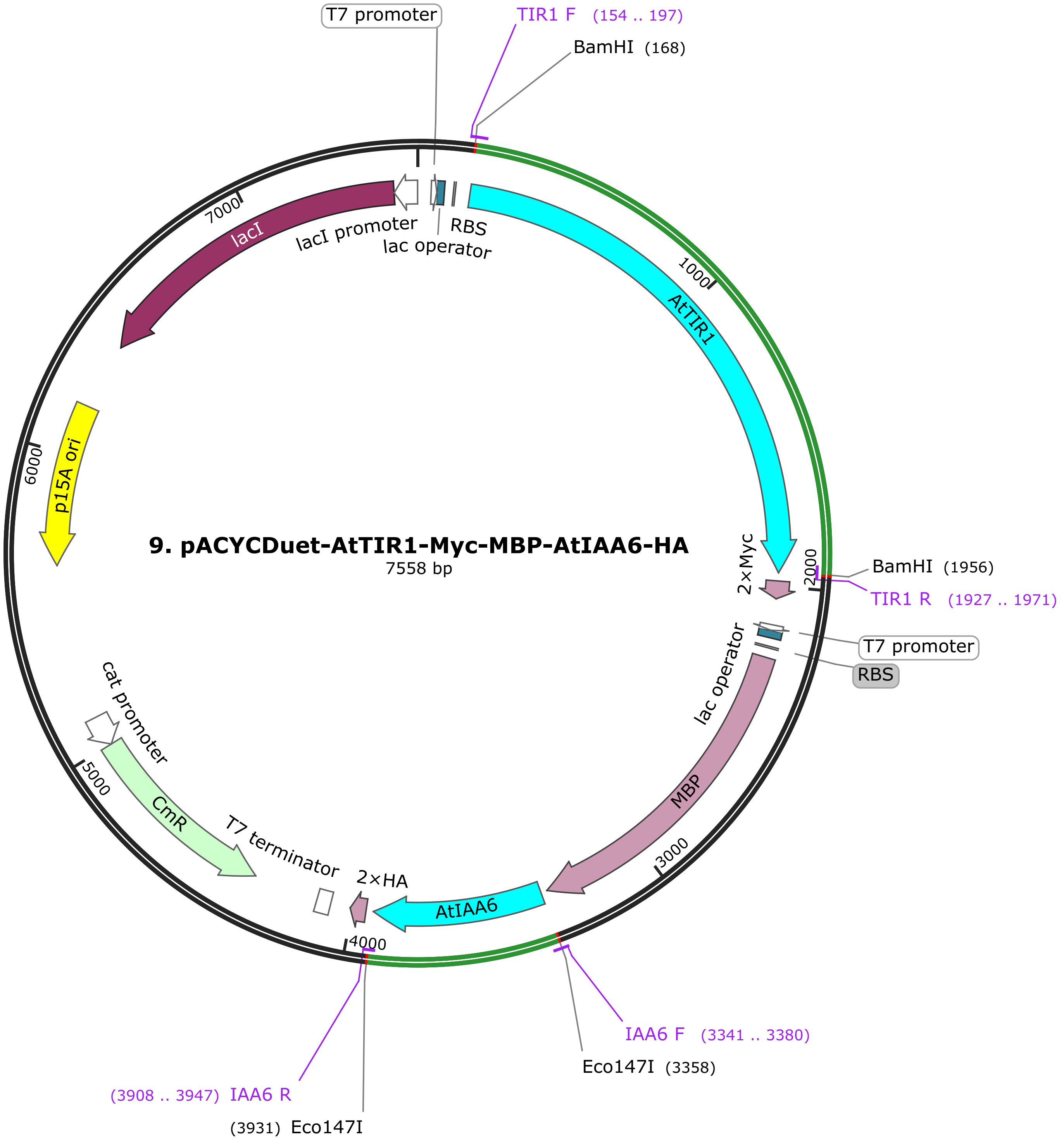

9. pACYCDuet-AtTIR1-Myc-MBP-AtIAA6-HA
7558 bp
T7 promoter
TIR1 F (154 .. 197)
BamHI (168)
lacI promoter
lac operator
RBS
lacI
7000
1000
AtTIR1
6000
p15A ori
2×Myc
2000
BamHI (1956)
TIR1 R (1927 .. 1971)
T7 promoter
RBS
lac operator
cat promoter
5000
CmR
MBP
3000
T7 terminator
2×HA
AtIAA6
4000
IAA6 F (3341 .. 3380)
Eco147I (3358)
(3908 .. 3947) IAA6 R
(3931) Eco147I
Created with SnapGene®

5' gggatctcgacgctctcccttatgcgactcctgcattaggaaattaatacgactcactataggggaattgtgagcggataacaattcccc 90
3' ccctagagctgcgagagggaatacgctgaggacgtaatcctttaattatgctgagtgatatccccttaacactcgcctattgttaagggg

T7 promoter | lac operator

BamHI

TIR1 F
catcaccaagccaggGatccATGCAGA

tgtagaaataattttgtttaactttaataaggagatataccatgggcagcagcGcatcaccatcatcaccaagccaggGatccATGCAGA 180
acatctttattaaaacaaattgaaattattcctctatatggtacccgtcgtcgCgtagtggtagtagtggttcggtccCtaggTACGTCT

1
Met Gln
RBS | AtTIR1

TIR1 F
AGCGAATAGCCTTGTCG

AGCGAATAGCCTTGTCGTTTCCAGAAGAGGTACTAGAGCATGTGTTCTCGTTTATTCAGCTGGATAAGGATAGGAACTCAGTCTCTCTGG 270
TCGCTTATCGGAACAGCAAAGGTCTTCTCCATGATCTCGTACACAAGAGCAAATAAGTCGACCTATTCCTATCCTTGAGTCAGAGAGACC

5 10 15 20 25 30
Lys Arg Ile Ala Leu Ser Phe Pro Glu Glu Val Leu Glu His Val Phe Ser Phe Ile Gln Leu Asp Lys Asp Arg Asn Ser Val Ser Leu
AtTIR1

TGTGCAAGTCATGGTACGAGATCGAGCGGTGGTGCAGGAGGAAAGTCTTCATCGGGAACTGCTACGCCGTGAGTCCAGCGACGGTGATTA 360
ACACGTTCAGTACCATGCTCTAGCTCGCCACCACGTCCTCCTTTCAGAAGTAGCCCTTGACGATGCGGCACTCAGGTCGCTGCCACTAAT

35 40 45 50 55 60
Val Cys Lys Ser Trp Tyr Glu Ile Glu Arg Trp Cys Arg Arg Lys Val Phe Ile Gly Asn Cys Tyr Ala Val Ser Pro Ala Thr Val Ile
AtTIR1

GGAGGTTCCCGAAAGTGAGATCCGTGGAGCTTAAAGGAAAACCTCACTTTGCTGACTTTAATTTGGTACCTGACGGATGGGGAGGTTACG 450
CCTCCAAGGGCTTTCACTCTAGGCACCTCGAATTTCCTTTTGGAGTGAAACGACTGAAATTAAACCATGGACTGCCTACCCCTCCAATGC

65 70 75 80 85 90
Arg Arg Phe Pro Lys Val Arg Ser Val Glu Leu Lys Gly Lys Pro His Phe Ala Asp Phe Asn Leu Val Pro Asp Gly Trp Gly Gly Tyr
AtTIR1

TGTATCCATGGATTGAGGCCATGTCTTCGTCTTACACGTGGCTTGAAGAGATAAGGCTGAAGAGGATGGTGGTCACCGACGATTGCTTGG 540
ACATAGGTACCTAACTCCGGTACAGAAGCAGAATGTGCACCGAACTTCTCTATTCCGACTTCTCCTACCACCAGTGGCTGCTAACGAACC

95 100 105 110 115 120
Val Tyr Pro Trp Ile Glu Ala Met Ser Ser Ser Tyr Thr Trp Leu Glu Glu Ile Arg Leu Lys Arg Met Val Val Thr Asp Asp Cys Leu
AtTIR1

AGCTCATAGCCAAGTCTTTTAAGAATTTTAAGGTTCTTGTGCTTTCTTCCTGCGAAGGCTTCTCCACCGATGGTCTCGCTGCTATCGCTG 630
TCGAGTATCGGTTCAGAAAATTCTTAAAATTCCAAGAACACGAAAGAAGGACGCTTCCGAAGAGGTGGCTACCAGAGCGACGATAGCGAC

125 130 135 140 145 150
Glu Leu Ile Ala Lys Ser Phe Lys Asn Phe Lys Val Leu Val Leu Ser Ser Cys Glu Gly Phe Ser Thr Asp Gly Leu Ala Ala Ile Ala
AtTIR1

CCACTTGCAGGAATCTGAAAGAGCTTGACTTACGAGAGAGTGATGTTGACGACGTTAGTGGCCACTGGCTTAGCCATTTCCCAGATACAT 720
GGTGAACGTCCTTAGACTTTCTCGAACTGAATGCTCTCTCACTACAACTGCTGCAATCACCGGTGACCGAATCGGTAAAGGGTCTATGTA

155 160 165 170 175 180
Ala Thr Cys Arg Asn Leu Lys Glu Leu Asp Leu Arg Glu Ser Asp Val Asp Asp Val Ser Gly His Trp Leu Ser His Phe Pro Asp Thr
AtTIR1

ACACTTCTTTGGTATCACTCAATATATCTTGCTTAGCATCTGAGGTCAGTTTCTCTGCTCTGGAAAGGCTGGTGACTAGGTGTCCCAATC 810
TGTGAAGAAACCATAGTGAGTTATATAGAACGAATCGTAGACTCCAGTCAAAGAGACGAGACCTTTCCGACCACTGATCCACAGGGTTAG

185 190 195 200 205 210
Tyr Thr Ser Leu Val Ser Leu Asn Ile Ser Cys Leu Ala Ser Glu Val Ser Phe Ser Ala Leu Glu Arg Leu Val Thr Arg Cys Pro Asn
AtTIR1

TCAAGTCTCTCAAGCTTAACCGAGCTGTTCCACTTGAAAAATTGGCTACTTTACTTCAAAGAGCACCTCAATTGGAGGAATTGGGCACTG 900
AGTTCAGAGAGTTCGAATTGGCTCGACAAGGTGAACTTTTTAACCGATGAAATGAAGTTTCTCGTGGAGTTAACCTCCTTAACCCGTGAC

215 220 225 230 235 240
Leu Lys Ser Leu Lys Leu Asn Arg Ala Val Pro Leu Glu Lys Leu Ala Thr Leu Leu Gln Arg Ala Pro Gln Leu Glu Glu Leu Gly Thr
AtTIR1

GTGGGTACACTGCAGAAGTGCGACCAGATGTTTACTCTGGTTTATCTGTAGCGCTCTCTGGGTGCAAGGAATTGAGGTGCTTATCTGGAT 990
CACCCATGTGACGTCTTCACGCTGGTCTACAAATGAGACCAAATAGACATCGCGAGAGACCCACGTTCCTTAACTCCACGAATAGACCTA
245 250 255 260 265 270
Gly Gly Tyr Thr Ala Glu Val Arg Pro Asp Val Tyr Ser Gly Leu Ser Val Ala Leu Ser Gly Cys Lys Glu Leu Arg Cys Leu Ser Gly
AtTIR1

TTTGGGATGCTGTTCCTGCCTATCTTCCAGCAGTTTATTCGGTTTGCAGTCGGCTTACAACTTTGAATCTGAGTTATGCAACAGTCCAGA 1080
AAACCCTACGACAAGGACGGATAGAAGGTCGTCAAATAAGCCAAACGTCAGCCGAATGTTGAAACTTAGACTCAATACGTTGTCAGGTCT
275 280 285 290 295 300
Phe Trp Asp Ala Val Pro Ala Tyr Leu Pro Ala Val Tyr Ser Val Cys Ser Arg Leu Thr Thr Leu Asn Leu Ser Tyr Ala Thr Val Gln
AtTIR1

GCTATGATCTTGTCAAGCTTCTTTGTCAATGCCCTAAACTGCAGCGCCTCTGGGTGCTTGACTACATCGAGGATGCTGGTCTTGAGGTGC 1170
CGATACTAGAACAGTTCGAAGAAACAGTTACGGGATTTGACGTCGCGGAGACCCACGAACTGATGTAGCTCCTACGACCAGAACTCCACG
305 310 315 320 325 330
Ser Tyr Asp Leu Val Lys Leu Leu Cys Gln Cys Pro Lys Leu Gln Arg Leu Trp Val Leu Asp Tyr Ile Glu Asp Ala Gly Leu Glu Val
AtTIR1

TTGCTTCAACCTGCAAGGACCTACGCGAGCTGAGAGTGTTTCCGTCCGAGCCTTTTGTCATGGAACCAAATGTGGCATTGACGGAACAGG 1260
AACGAAGTTGGACGTTCCTGGATGCGCTCGACTCTCACAAAGGCAGGCTCGGAAAACAGTACCTTGGTTTACACCGTAACTGCCTTGTCC
335 340 345 350 355 360
Leu Ala Ser Thr Cys Lys Asp Leu Arg Glu Leu Arg Val Phe Pro Ser Glu Pro Phe Val Met Glu Pro Asn Val Ala Leu Thr Glu Gln
AtTIR1

GGCTTGTCTCCGTCTCCATGGGCTGTCCAAAACTCGAGTCGGTTCTCTACTTCTGCCGTCAAATGACCAATGCTGCATTGATAACCATTG 1350
CCGAACAGAGGCAGAGGTACCCGACAGGTTTTGAGCTCAGCCAAGAGATGAAGACGGCAGTTTACTGGTTACGACGTAACTATTGGTAAC
365 370 375 380 385 390
Gly Leu Val Ser Val Ser Met Gly Cys Pro Lys Leu Glu Ser Val Leu Tyr Phe Cys Arg Gln Met Thr Asn Ala Ala Leu Ile Thr Ile
AtTIR1

CTAGGAACCGTCCCAACATGACTCGCTTCCGTTTGTGCATCATTGAGCCAAAAGCCCCAGACTATCTGACTCTAGAGCCACTGGATATTG 1440
GATCCTTGGCAGGGTTGTACTGAGCGAAGGCAAACACGTAGTAACTCGGTTTTCGGGGTCTGATAGACTGAGATCTCGGTGACCTATAAC
395 400 405 410 415 420
Ala Arg Asn Arg Pro Asn Met Thr Arg Phe Arg Leu Cys Ile Ile Glu Pro Lys Ala Pro Asp Tyr Leu Thr Leu Glu Pro Leu Asp Ile
AtTIR1

GATTTGGAGCCATAGTAGAGCACTGCAAGGATCTCCGTCGCCTCTCTCTATCTGGCCTCTTGACCGACAAGGTTTTTGAATACATTGGGA 1530
CTAAACCTCGGTATCATCTCGTGACGTTCCTAGAGGCAGCGGAGAGAGATAGACCGGAGAACTGGCTGTTCCAAAAACTTATGTAACCCT
425 430 435 440 445 450
Gly Phe Gly Ala Ile Val Glu His Cys Lys Asp Leu Arg Arg Leu Ser Leu Ser Gly Leu Leu Thr Asp Lys Val Phe Glu Tyr Ile Gly
AtTIR1

CATATGCCAAGAAGATGGAAATGCTCTCAGTGGCATTTGCAGGAGACAGTGACTTAGGCATGCATCATGTTTTGTCCGGGTGCGATAGCT 1620
GTATACGGTTCTTCTACCTTTACGAGAGTCACCGTAAACGTCCTCTGTCACTGAATCCGTACGTAGTACAAAACAGGCCCACGCTATCGA
455 460 465 470 475 480
Thr Tyr Ala Lys Lys Met Glu Met Leu Ser Val Ala Phe Ala Gly Asp Ser Asp Leu Gly Met His His Val Leu Ser Gly Cys Asp Ser
AtTIR1

TGAGGAAACTAGAGATAAGGGACTGCCCGTTTGGAGACAAGGCGCTTTTGGCCAATGCTTCAAAGCTGGAGACAATGCGATCCCTTTGGA 1710
ACTCCTTTGATCTCTATTCCCTGACGGGCAAACCTCTGTTCCGCGAAAACCGGTTACGAAGTTTCGACCTCTGTTACGCTAGGGAAACCT
485 490 495 500 505 510
Leu Arg Lys Leu Glu Ile Arg Asp Cys Pro Phe Gly Asp Lys Ala Leu Leu Ala Asn Ala Ser Lys Leu Glu Thr Met Arg Ser Leu Trp
AtTIR1

TGTCTTCTTGTTCCGTGAGTTTTGGAGCCTGCAAGTTACTAGGACAGAAGATGCCAAAGCTGAATGTGGAAGTCATCGATGAACGGGGTG 1800
ACAGAAGAACAAGGCACTCAAAACCTCGGACGTTCAATGATCCTGTCTTCTACGGTTTCGACTTACACCTTCAGTAGCTACTTGCCCCAC
515 520 525 530 535 540
Met Ser Ser Cys Ser Val Ser Phe Gly Ala Cys Lys Leu Leu Gly Gln Lys Met Pro Lys Leu Asn Val Glu Val Ile Asp Glu Arg Gly
AtTIR1

CACCGGACTCGAGACCAGAGAGCTGCCCTGTTGAGAGAGTCTTCATATACCGAACAGTGGCTGGTCCTCGATTTGACATGCCTGGCTTCG 1890
GTGGCCTGAGCTCTGGTCTCTCGACGGGACAACTCTCTCAGAAGTATATGGCTTGTCACCGACCAGGAGCTAAACTGTACGGACCGAAGC
545 550 555 560 565 570
Ala Pro Asp Ser Arg Pro Glu Ser Cys Pro Val Glu Arg Val Phe Ile Tyr Arg Thr Val Ala Gly Pro Arg Phe Asp Met Pro Gly Phe
AtTIR1

BamHI
TCTGGAACATGGACCAAGACTCAACAATGAGGTTTTCCAGGCAAATCATTACTACTAACGGATTAGGATCCGAGCTCGATATCACTAGTC 1980
AGACCTTGTACCTGGTTCTGAGTTGTTACTCCAAAAGGTCCGTTTAGTAATGATGATTGCCTAATCCTAGGCTCGAGCTATAGTGATCAG
575 580 585 590
Val Trp Asn Met Asp Gln Asp Ser Thr Met Arg Phe Ser Arg Gln Ile Ile Thr Thr Asn Gly Leu Gly Ser Glu Leu Asp Ile Thr Ser
AtTIR1 (in frame with AtTIR1)
GGTCCGTTTAGTAATGATGATTGCCTAATCCTAGGCTCGAGCTAT
TIR1 R

CTGAGCAGAAGCTCATCTCCGAGGAGGACCTCGAGCAGAAGCTCATCTCCGAGGAGGACCTCCTGActgcaggtcgacaagcttgcggcc 2070
GACTCGTCTTCGAGTAGAGGCTCCTCCTGGAGCTCGTCTTCGAGTAGAGGCTCCTCCTGGAGGACTgacgtccagctgttcgaacgccgg
1 5 10 15 20
Pro Glu Gln Lys Leu Ile Ser Glu Glu Asp Leu Glu Gln Lys Leu Ile Ser Glu Glu Asp Leu Leu Thr Ala Gly Arg Gln Ala Cys Gly
2×Myc (in frame with 2×Myc)

gcataatgcttaagtcgaacagaaagtaatcgtattgtacacggccgcataatcgaaattaatacgactcactataggggaattgtgagc 2160
cgtattacgaattcagcttgtctttcattagcataacatgtgccggcgtattagctttaattatgctgagtgatatccccttaacactcg
Arg Ile Met Leu Lys Ser Asn Arg Lys *
(in frame with 2×Myc)
T7 promoter lac operator

ggataacaattccccatcttagtatattagttaagtataagaaggagatatacatatggcagatctcaattgggaattcatgaaaatcga 2250
cctattgttaaggggtagaatcatataatcaattcatattcttcctctatatgtataccgtctagagttaacccttaagtactttagct
1
Met Lys Ile Glu
lac operator RBS MBP

agaaggtaaactggtaatctggattaacggcgataaaggctataacggtctcgctgaagtcggtaagaaattcgagaaagataccggaat 2340
tcttccatttgaccattagacctaattgccgctatttccgatattgccagagcgacttcagccattctttaagctctttctatggcctta
5 10 15 20 25 30
Glu Gly Lys Leu Val Ile Trp Ile Asn Gly Asp Lys Gly Tyr Asn Gly Leu Ala Glu Val Gly Lys Lys Phe Glu Lys Asp Thr Gly Ile
MBP

taaagtcaccgttgagcatccggataaactggaagagaaattcccacaggttgcggcaactggcgatggccctgacattatcttctgggc 2430
atttcagtggcaactcgtaggcctatttgaccttctctttaagggtgtccaacgccgttgaccgctaccgggactgtaatagaagacccg
35 40 45 50 55 60
Lys Val Thr Val Glu His Pro Asp Lys Leu Glu Glu Lys Phe Pro Gln Val Ala Ala Thr Gly Asp Gly Pro Asp Ile Ile Phe Trp Ala
MBP

acacgaccgctttggtggctacgctcaatctggcctgttggctgaaatcaccccggacaaagcgttccaggacaagctgtatccgtttac 2520
tgtgctggcgaaaccaccgatgcgagttagaccggacaaccgactttagtggggcctgtttcgcaaggtcctgttcgacataggcaaatg
65 70 75 80 85 90
His Asp Arg Phe Gly Gly Tyr Ala Gln Ser Gly Leu Leu Ala Glu Ile Thr Pro Asp Lys Ala Phe Gln Asp Lys Leu Tyr Pro Phe Thr
MBP

ctgggatgccgtacgttacaacggcaagctgattgcttacccgatcgctgttgaagcgttatcgctgatttataacaaagatctgctgcc 2610
gaccctacggcatgcaatgttgccgttcgactaacgaatgggctagcgacaacttcgcaatagcgactaaatattgtttctagacgacgg
95 100 105 110 115 120
Trp Asp Ala Val Arg Tyr Asn Gly Lys Leu Ile Ala Tyr Pro Ile Ala Val Glu Ala Leu Ser Leu Ile Tyr Asn Lys Asp Leu Leu Pro
MBP

gaacccgccaaaaacctgggaagagatcccggcgctggataaagaactgaaagcgaaaggtaagagcgcgctgatgttcaacctgcaaga 2700
cttgggcggtttttggacccttctctagggccgcgacctatttcttgactttcgctttccattctcgcgcgactacaagttggacgttct
125 130 135 140 145 150
Asn Pro Pro Lys Thr Trp Glu Glu Ile Pro Ala Leu Asp Lys Glu Leu Lys Ala Lys Gly Lys Ser Ala Leu Met Phe Asn Leu Gln Glu
MBP

accgtacttcacctggccgctgattgctgctgacggggttatgcgttcaagtatgaaaacggcaagtacgacattaaagacgtgggcgt 2790
tggcatgaagtggaccggcgactaacgacgactgccccaatacgcaagttcatacttttgccgttcatgctgtaatttctgcacccgca
155 160 165 170 175 180
Pro Tyr Phe Thr Trp Pro Leu Ile Ala Ala Asp Gly Gly Tyr Ala Phe Lys Tyr Glu Asn Gly Lys Tyr Asp Ile Lys Asp Val Gly Val
MBP

ggataacgctggcgcgaaagcgggtctgaccttcctggttgacctgattaaaaacaaacacatgaatgcagacaccgattactccatcgc 2880
cctattgcgaccgcgctttcgcccagactggaaggaccaactggactaatttttgtttgtgtacttacgtctgtggctaatgaggtagcg
185 190 195 200 205 210
Asp Asn Ala Gly Ala Lys Ala Gly Leu Thr Phe Leu Val Asp Leu Ile Lys Asn Lys His Met Asn Ala Asp Thr Asp Tyr Ser Ile Ala
MBP

agaagctgcctttaataaaggcgaaacagcgatgaccatcaacggcccgtgggcatggtccaacatcgacaccagcaaagtgaattatgg 2970
tcttcgacggaaattatttccgctttgtcgctactggtagttgccgggcacccgtaccaggttgtagctgtggtcgtttcacttaatacc
215 220 225 230 235 240
Glu Ala Ala Phe Asn Lys Gly Glu Thr Ala Met Thr Ile Asn Gly Pro Trp Ala Trp Ser Asn Ile Asp Thr Ser Lys Val Asn Tyr Gly
MBP

tgtaacggtactgccgaccttcaagggtcaaccatccaaaccgttcgttggcgtgctgagcgcaggtattaacgccgccagtccgaacaa 3060
acattgccatgacggctggaagttcccagttggtaggtttggcaagcaaccgcacgactcgcgtccataattgcggcggtcaggcttgtt
245 250 255 260 265 270
Val Thr Val Leu Pro Thr Phe Lys Gly Gln Pro Ser Lys Pro Phe Val Gly Val Leu Ser Ala Gly Ile Asn Ala Ala Ser Pro Asn Lys
MBP

agagctggcaaaagagttcctcgaaaactatctgctgactgatgaaggtctggaagcggttaataaagacaaaccgctgggtgccgtagc 3150
tctcgaccgttttctcaaggagcttttgatagacgactgactacttccagaccttcgccaattatttctgtttggcgacccacggcatcg
275 280 285 290 295 300
Glu Leu Ala Lys Glu Phe Leu Glu Asn Tyr Leu Leu Thr Asp Glu Gly Leu Glu Ala Val Asn Lys Asp Lys Pro Leu Gly Ala Val Ala
MBP

gctgaagtcttacgaggaagagttggcgaaagatccacgtattgccgccactatggaaaacgcccagaaaggtgaaatcatgccgaacat 3240
cgacttcagaatgctccttctcaaccgctttctaggtgcataacggcggtgataccttttgcgggtctttccactttagtacggcttgta
305 310 315 320 325 330
Leu Lys Ser Tyr Glu Glu Glu Leu Ala Lys Asp Pro Arg Ile Ala Ala Thr Met Glu Asn Ala Gln Lys Gly Glu Ile Met Pro Asn Ile
MBP

cccgcagatgtccgctttctggtatgccgtgcgtactgcggtgatcaacgccgccagcggtcgtcagactgtcgatgaagccctgaaaga 3330
gggcgtctacaggcgaaagaccatacggcacgcatgacgccactagttgcggcggtcgccagcagtctgacagctacttcgggactttct
335 340 345 350 355 360
Pro Gln Met Ser Ala Phe Trp Tyr Ala Val Arg Thr Ala Val Ile Asn Ala Ala Ser Gly Arg Gln Thr Val Asp Glu Ala Leu Lys Asp
MBP

Eco147I
IAA6 F
gaattcgagCCCGGGAGGCCTATGGCAAAGGAAGGTCTAG
cgcgcagactgaattcgagCCCGGGAGGCCTATGGCAAAGGAAGGTCTAGCACTCGAGATCACAGAGCTTCGATTGGGTCTTCCAGGAGA 3420
gcgcgtctgacttaagctcGGGCCCTCCGGATACCGTTTCCTTCCAGATCGTGAGCTCTAGTGTCTCGAAGCTAACCCAGAAGGTCCTCT
365 1 5 10 15 20
Ala Gln Thr Glu Phe Glu Pro Gly Arg Pro Met Ala Lys Glu Gly Leu Ala Leu Glu Ile Thr Glu Leu Arg Leu Gly Leu Pro Gly Asp
MBP (in frame with MBP) AtIAA6

TAATTATAGCGAAATATCAGTATGCGGATCGAGTAAGAAGAAGAAGAGGGTGCTCTCGGATATGATGACCTCATCAGCGTTAGATACTGA 3510
ATTAATATCGCTTTATAGTCATACGCCTAGCTCATTCTTCTTCTTCTCCCACGAGAGCCTATACTACTGGAGTAGTCGCAATCTATGACT
25 30 35 40 45 50
Asn Tyr Ser Glu Ile Ser Val Cys Gly Ser Ser Lys Lys Lys Lys Arg Val Leu Ser Asp Met Met Thr Ser Ser Ala Leu Asp Thr Glu
AtIAA6

GAATGAAAACAGCGTCGTTTCATCAGTTGAAGATGAATCACTGCCGGTTGTGAAGAGTCAAGCGGTGGGATGGCCACCTGTGTGTTCTTA 3600
CTTACTTTTGTCGCAGCAAAGTAGTCAACTTCTACTTAGTGACGGCCAACACTTCTCAGTTCGCCACCCTACCGGTGGACACACAAGAAT
55 60 65 70 75 80
Asn Glu Asn Ser Val Val Ser Ser Val Glu Asp Glu Ser Leu Pro Val Val Lys Ser Gln Ala Val Gly Trp Pro Pro Val Cys Ser Tyr
AtIAA6

CAGGAGAAAGAAGAACAATGAGGAAGCATCGAAAGCTATAGGCTACGTGAAAGTGAGCATGGATGGTGTGCCATACATGAGGAAGATTGA 3690

GTCCTCTTTCTTCTTGTTACTCCTTCGTAGCTTTCGATATCCGATGCACTTTCACTCGTACCTACCACACGGTATGTACTCCTTCTAACT

85 90 95 100 105 110

Arg Arg Lys Lys Asn Asn Glu Glu Ala Ser Lys Ala Ile Gly Tyr Val Lys Val Ser Met Asp Gly Val Pro Tyr Met Arg Lys Ile Asp

AtIAA6

CCTTGGTTCGAGCAACAGTTATATTAATCTAGTCACGGTTCTTGAGAATCTCTTCGGCTGTCTTGGCATAGGAGTGGCGAAGGAGGGTAA 3780

GGAACCAAGCTCGTTGTCAATATAATTAGATCAGTGCCAAGAACTCTTAGAGAAGCCGACAGAACCGTATCCTCACCGCTTCCTCCCATT

115 120 125 130 135 140

Leu Gly Ser Ser Asn Ser Tyr Ile Asn Leu Val Thr Val Leu Glu Asn Leu Phe Gly Cys Leu Gly Ile Gly Val Ala Lys Glu Gly Lys

AtIAA6

GAAGTGTGAATACATTATTATATACGAAGACAAGGATAGAGACTGGATGCTCGTCGGAGATGTACCTTGGCAGATGTTTAAAGAATCATG 3870

CTTCACACTTATGTAATAATATATGCTTCTGTTCCTATCTCTGACCTACGAGCAGCCTCTACATGGAACCGTCTACAAATTTCTTAGTAC

145 150 155 160 165 170

Lys Cys Glu Tyr Ile Ile Ile Tyr Glu Asp Lys Asp Arg Asp Trp Met Leu Val Gly Asp Val Pro Trp Gln Met Phe Lys Glu Ser Cys

AtIAA6

Eco147I

CAAGAGGCTGAGGATCGTGAAGAGATCAGATGCAACTGGTTTTGGTCTCCAGCAAGATAGGCCTctcggcgcgcctgcaggtTACCCATA 3960

GTTCTCCGACTCCTAGCACTTCTCTAGTCTACGTTGACCAAAACCAGAGGTCGTTCTATCCGGAgagccgcgcggacgtccaATGGGTAT

175 180 185 1

Lys Arg Leu Arg Ile Val Lys Arg Ser Asp Ala Thr Gly Phe Gly Leu Gln Gln Asp Arg Pro Leu Gly Ala Pro Ala Gly Tyr Pro Tyr

AtIAA6 (in frame with AtIAA6) 2×HA

CCAAAACCAGAGGTCGTTCTATCCGGAgagccgcgcggac

IAA6 R

CGACGTACCAGATTACGCTTACCCATACGACGTACCAGATTACGCTcgacaagcttgcggccgcataatgcttaagtaattaacctaggc 4050

GCTGCATGGTCTAATGCGAATGGGTATGCTGCATGGTCTAATGCGAgctgttcgaacgccggcgtattacgaattcattaattggatccg

5 10 15

Asp Val Pro Asp Tyr Ala Tyr Pro Tyr Asp Val Pro Asp Tyr Ala Arg Gln Ala Cys Gly Arg Ile Met Leu Lys *

2×HA (in frame with 2×HA)

tgctgccaccgctgagcaataactagcataaccccttggggcctctaaacgggtcttgaggggttttttgctgaaacctcaggcatttga 4140

acgacggtggcgactcgttattgatcgtattggggaaccccggagatttgcccagaactccccaaaaaacgactttggagtccgtaaact

T7 terminator

gaagcacacggtcacactgcttccggtagtcaataaaccggtaaaccagcaatagacataagcggctatttaacgaccctgccctgaacc 4230

cttcgtgtgccagtgtgacgaaggccatcagttatttggccatttggtcgttatctgtattcgccgataaattgctgggacgggacttgg

gacgaccgggtcgaatttgctttcgaatttctgccattcatccgcttattatcacttattcaggcgtagcaccaggcgtttaagggcacc 4320

ctgctggcccagcttaaacgaaagcttaaagacggtaagtaggcgaataatagtgaataagtccgcatcgtggtccgcaaattcccgtgg

aataactgccttaaaaaaattacgccccgccctgccactcatcgcagtactgttgtaattcattaagcattctgccgacatggaagccat 4410

ttattgacggaatttttttaatgcggggcgggacggtgagtagcgtcatgacaacattaagtaattcgtaagacggctgtaccttcggta

220 215 210 205 200

* Ala Gly Gly Gln Trp Glu Asp Cys Tyr Gln Gln Leu Glu Asn Leu Met Arg Gly Val His Phe Gly Asp

CmR

cacagacggcatgatgaacctgaatcgccagcggcatcagcaccttgtcgccttgcgtataatatttgcccatagtgaaaacgggggcga 4500

gtgtctgccgtactacttggacttagcggtcgccgtagtcgtggaacagcggaacgcatattataaacgggtatcacttttgcccccgct

195 190 185 180 175 170

Cys Val Ala His His Val Gln Ile Ala Leu Pro Met Leu Val Lys Asp Gly Gln Thr Tyr Tyr Lys Gly Met Thr Phe Val Pro Ala Phe

CmR

agaagttgtccatattggccacgtttaaatcaaaactggtgaaactcacccagggattggctgagacgaaaaacatattctcaataaacc 4590

tcttcaacaggtataaccggtgcaaatttagttttgaccactttgagtgggtccctaaccgactctgctttttgtataagagttatttgg

165 160 155 150 145 140

Phe Asn Asp Met Asn Ala Val Asn Leu Asp Phe Ser Thr Phe Ser Val Trp Pro Asn Ala Ser Val Phe Phe Met Asn Glu Ile Phe Gly

CmR

ctttagggaaataggccaggttttcaccgtaacacgccacatcttgcgaatatatgtgtagaaactgccggaaatcgtcgtggtattcac 4680
gaaatccctttatccggtccaaaagtggcattgtgcggtgtagaacgcttatatacacatctttgacggcctttagcagcaccataagtg
135 130 125 120 115 110
Lys Pro Phe Tyr Ala Leu Asn Glu Gly Tyr Cys Ala Val Asp Gln Ser Tyr Ile His Leu Phe Gln Arg Phe Asp Asp His Tyr Glu Ser
CmR

tccagagcgatgaaaacgtttcagtttgctcatggaaaacggtgtaacaagggtgaacactatcccatatcaccagctcaccgtctttca 4770
aggtctcgctacttttgcaaagtcaaacgagtaccttttgccacattgttcccacttgtgatagggtatagtggtcgagtggcagaaagt
105 100 95 90 85 80
Trp Leu Ser Ser Phe Thr Glu Thr Gln Glu His Phe Val Thr Tyr Cys Pro His Val Ser Asp Trp Ile Val Leu Glu Gly Asp Lys Met
CmR

ttgccatacggaactccggatgagcattcatcaggcgggcaagaatgtgaataaaggccggataaaacttgtgcttatttttctttacgg 4860
aacggtatgccttgaggcctactcgtaagtagtccgcccgttcttacacttatttccggcctattttgaacacgaataaaaagaaatgcc
75 70 65 60 55 50
Ala Met Arg Phe Glu Pro His Ala Asn Met Leu Arg Ala Leu Ile His Ile Phe Ala Pro Tyr Phe Lys His Lys Asn Lys Lys Val Thr
CmR

tctttaaaaaggccgtaatatccagctgaacggtctggttataggtacattgagcaactgactgaaatgcctcaaaatgttctttacgat 4950
agaaatttttccggcattataggtcgacttgccagaccaatatccatgtaactcgttgactgactttacggagttttacaagaaatgcta
45 40 35 30 25 20
Lys Leu Phe Ala Thr Ile Asp Leu Gln Val Thr Gln Asn Tyr Thr Cys Gln Ala Val Ser Gln Phe Ala Glu Phe His Glu Lys Arg His
CmR

gccattgggatatatcaacggtggtatatccagtgatttttttctccattttagcttccttagctcctgaaaatctcgataactcaaaaa 5040
cggtaaccctatatagttgccaccatataggtcactaaaaaaagaggtaaaatcgaaggaatcgaggacttttagagctattgagttttt
15 10 5 1
Trp Gln Ser Ile Asp Val Thr Thr Tyr Gly Thr Ile Lys Lys Glu Met
CmR
cat promoter

atacgcccggtagtgatcttatttcattatggtgaaagttggaacctcttacgtgccgatcaacgtctcattttcgccaaaagttggccc 5130
tatgcgggccatcactagaataaagtaataccactttcaaccttggagaatgcacggctagttgcagagtaaaagcggttttcaaccggg
cat promoter

agggcttcccggtatcaacagggacaccaggatttatttattctgcgaagtgatcttccgtcacaggtatttattcggcgcaaagtgcgt 5220
tcccgaagggccatagttgtccctgtggtcctaaataaataagacgcttcactagaaggcagtgtccataaataagccgcgtttcacgca

cgggtgatgctgccaacttactgatttagtgtatgatggtgtttttgaggtgctccagtggcttctgtttctatcagctgtccctcctgt 5310
gcccactacgacggttgaatgactaaatcacatactaccacaaaaactccacgaggtcaccgaagacaaagatagtcgacagggaggaca

tcagctactgacggggtggtgcgtaacggcaaaagcaccgccggacatcagcgctagcggagtgtatactggcttactatgttggcactg 5400
agtcgatgactgccccaccacgcattgccgttttcgtggcggcctgtagtcgcgatcgcctcacatatgaccgaatgatacaaccgtgac

atgagggtgtcagtgaagtgcttcatgtggcaggagaaaaaaggctgcaccggtgcgtcagcagaatatgtgatacaggatatattccgc 5490
tactcccacagtcacttcacgaagtacaccgtcctcttttttccgacgtggccacgcagtcgtcttatacactatgtcctatataaggcg

ttcctcgctcactgactcgctacgctcggtcgttcgactgcggcgagcggaaatggcttacgaacggggcggagatttcctggaagatgc 5580
aaggagcgagtgactgagcgatgcgagccagcaagctgacgccgctcgcctttaccgaatgcttgccccgcctctaaaggaccttctacg

caggaagatacttaacagggaagtgagagggccgcggcaaagccgtttttccataggctccgcccccctgacaagcatcacgaaatctga 5670
gtccttctatgaattgtcccttcactctcccggcgccgtttcggcaaaaaggtatccgaggcggggggactgttcgtagtgctttagact
p15A ori

cgctcaaatcagtggtggcgaaacccgacaggactataaagataccaggcgtttccccctggcggctccctcgtgcgctctcctgttcctg 5760
gcgagtttagtcaccaccgctttgggctgtcctgatatttctatggtccgcaaaggggaccgccgagggagcacgcgagaggacaaggac
p15A ori

cctttcggtttaccggtgtcattccgctgttatggccgcgtttgtctcattccacgcctgacactcagttccgggtaggcagttcgctcc 5850
ggaaagccaaatggccacagtaaggcgacaataccggcgcaaacagagtaaggtgcggactgtgagtcaaggcccatccgtcaagcgagg
p15A ori

aagctggactgtatgcacgaaccccccgttcagtccgaccgctgcgccttatccggtaactatcgtcttgagtccaacccggaaagacat 5940
ttcgacctgacatacgtgcttggggggcaagtcaggctggcgacgcggaataggccattgatagcagaactcaggttgggcctttctgta
p15A ori

gcaaaagcaccactggcagcagccactggtaattgatttagaggagttagtcttgaagtcatgcgccggttaaggctaaactgaaaggac 6030
cgttttcgtggtgaccgtcgtcggtgaccattaactaaatctcctcaatcagaacttcagtacgcggccaattccgatttgactttcctg
p15A ori

aagttttggtgactgcgctcctccaagccagttacctcggttcaaagagttggtagctcagagaaccttcgaaaaaccgccctgcaaggc 6120
ttcaaaaccactgacgcgaggaggttcggtcaatggagccaagtttctcaaccatcgagtctcttggaagctttttggcgggacgttccg
p15A ori

ggttttttcgttttcagagcaagagattacgcgcagaccaaaacgatctcaagaagatcatcttattaatcagataaaatatttctagat 6210
ccaaaaaagcaaaagtctcgttctctaatgcgcgtctggttttgctagagttcttctagtagaataattagtctattttataaagatcta
p15A ori

ttcagtgcaatttatctcttcaaatgtagcacctgaagtcagccccatacgatataagttgtaattctcatgttagtcatgccccgcgcc 6300
aagtcacgttaaatagagaagtttacatcgtggacttcagtcggggtatgctatattcaacattaagagtacaatcagtacggggcgcgg

caccggaaggagctgactgggttgaaggctctcaagggcatcggtcgagatcccggtgcctaatgagtgagctaacttacattaattgcg 6390
gtggccttcctcgactgacccaacttccgagagttcccgtagccagctctagggccacggattactcactcgattgaatgtaattaacgc

ttgcgctcactgcccgctttccagtcgggaaacctgtcgtgccagctgcattaatgaatcggccaacgcgcggggagaggcggtttgcgt 6480
aacgcgagtgacgggcgaaaggtcagccctttggacagcacggtcgacgtaattacttagccggttgcgcgcccctctccgccaaacgca
360 355 350 345 340 335
* Gln Gly Ser Glu Leu Arg Ser Val Gln Arg Ala Leu Gln Met Leu Ser Asp Ala Leu Ala Arg Pro Ser Ala Thr Gln Thr
lacI

attgggcgccagggtggtttttcttttcaccagtgagacgggcaacagctgattgcccttcaccgcctggccctgagagagttgcagcaa 6570
taacccgcggtcccaccaaaaagaaaagtggtcactctgcccgttgtcgactaacgggaagtggcggaccgggactctctcaacgtcgtt
330 325 320 315 310 305
Asn Pro Ala Leu Thr Thr Lys Arg Lys Val Leu Ser Val Pro Leu Leu Gln Asn Gly Lys Val Ala Gln Gly Gln Ser Leu Gln Leu Leu
lacI

gcggtccacgctggtttgccccagcaggcgaaaatcctgtttgatggtggttaacggcgggatataacatgagctgtcttcggtatcgtc 6660
cgccaggtgcgaccaaacggggtcgtccgcttttaggacaaactaccaccaattgccgccctatattgtactcgacagaagccatagcag
300 295 290 285 280 275
Arg Asp Val Ser Thr Gln Gly Leu Leu Arg Phe Asp Gln Lys Ile Thr Thr Leu Pro Pro Ile Tyr Cys Ser Ser Asp Glu Thr Asp Asp
lacI

gtatcccactaccgagatgtccgcaccaacgcgcagcccggactcggtaatggcgcgcattgcgcccagcgccatctgatcgttggcaac 6750
catagggtgatggctctacaggcgtggttgcgcgtcgggcctgagccattaccgcgcgtaacgcgggtcgcggtagactagcaaccgttg
270 265 260 255 250 245
Tyr Gly Val Val Ser Ile Asp Ala Gly Val Arg Leu Gly Ser Glu Thr Ile Ala Arg Met Ala Gly Leu Ala Met Gln Asp Asn Ala Val
lacI

cagcatcgcagtgggaacgatgccctcattcagcatttgcatggtttgttgaaaaccggacatggcactccagtcgccttcccgttccgc 6840
gtcgtagcgtcacccttgctacgggagtaagtcgtaaacgtaccaaacaacttttggcctgtaccgtgaggtcagcggaagggcaaggcg
240 235 230 225 220 215
Leu Met Ala Thr Pro Val Ile Gly Glu Asn Leu Met Gln Met Thr Gln Gln Phe Gly Ser Met Ala Ser Trp Asp Gly Glu Arg Glu Ala
lacI

```
tatcggctgaatttgattgcgagtgagatatttatgccagccagccagacgcagacgcgccgagacagaacttaatgggcccgctaacag   6930
atagccgacttaaactaacgctcactctataaatacggtcggtcggtctgcgtctgcgcggctctgtcttgaattacccgggcgattgtc
          210                 205                 200                 195                 190                 185
Ile Pro Gln Ile Gln Asn Arg Thr Leu Tyr Lys His Trp Gly Ala Leu Arg Leu Arg Ala Ser Val Ser Ser Leu Pro Gly Ala Leu Leu
< lacI

cgcgatttgctggtgacccaatgcgaccagatgctccacgcccagtcgcgtaccgtcttcatgggagaaaataatactgttgatgggtgt   7020
gcgctaaacgaccactgggttacgctggtctacgaggtgcgggtcagcgcatggcagaagtaccctcttttattatgacaactacccaca
          180                 175                 170                 165                 160                 155
Ala Ile Gln Gln His Gly Leu Ala Val Leu His Glu Val Gly Leu Arg Thr Gly Asp Glu His Ser Phe Ile Ile Ser Asn Ile Pro Thr
< lacI

ctggtcagagacatcaagaaataacgccggaacattagtgcaggcagcttccacagcaatggcatcctggtcatccagcggatagttaat   7110
gaccagtctctgtagttctttattgcggccttgtaatcacgtccgtcgaaggtgtcgttaccgtaggaccagtaggtcgcctatcaatta
          150                 145                 140                 135                 130                 125
Gln Asp Ser Val Asp Leu Phe Leu Ala Pro Val Asn Thr Cys Ala Ala Glu Val Ala Ile Ala Asp Gln Asp Asp Leu Pro Tyr Asn Ile
< lacI

gatcagcccactgacgcgttgcgcgagaagattgtgcaccgccgctttacaggcttcgacgccgcttcgttctaccatcgacaccaccac   7200
ctagtcgggtgactgcgcaacgcgctcttctaacacgtggcggcgaaatgtccgaagctgcggcgaagcaagatggtagctgtggtggtg
          120                 115                 110                 105                 100                 95
Ile Leu Gly Ser Val Arg Gln Ala Leu Leu Asn His Val Ala Ala Lys Cys Ala Glu Val Gly Ser Arg Glu Val Met Ser Val Val Val
< lacI

gctggcacccagttgatcggcgcgagatttaatcgccgcgacaatttgcgacggcgcgtgcagggccagactggaggtggcaacgccaat   7290
cgaccgtgggtcaactagccgcgctctaaattagcggcgctgttaaacgctgccgcgcacgtcccggtctgacctccaccgttgcggtta
          90                  85                  80                  75                  70                  65
Ser Ala Gly Leu Gln Asp Ala Arg Ser Lys Ile Ala Ala Val Ile Gln Ser Pro Ala His Leu Ala Leu Ser Ser Thr Ala Val Gly Ile
< lacI

cagcaacgactgtttgcccgccagttgttgtgccacgcggttgggaatgtaattcagctccgccatcgccgcttccacttttcccgcgt   7380
gtcgttgctgacaaacgggcggtcaacaacacggtgcgccaacccttacattaagtcgaggcggtagcggcgaaggtgaaaagggcgca
          60                  55                  50                  45                  40                  35
Leu Leu Ser Gln Lys Gly Ala Leu Gln Gln Ala Val Arg Asn Pro Ile Tyr Asn Leu Glu Ala Met Ala Ala Glu Val Lys Glu Arg Thr
< lacI

tttcgcagaaacgtggctggcctggttcaccacgcgggaaacggtctgataagagacaccggcatactctgcgacatcgtataacgttac   7470
aaagcgtctttgcaccgaccggaccaagtggtgcgccctttgccagactattctctgtggccgtatgagacgctgtagcatattgcaatg
          30                  25                  20                  15                  10                  5
Lys Ala Ser Val His Ser Ala Gln Asn Val Val Arg Ser Val Thr Gln Tyr Ser Val Gly Ala Tyr Glu Ala Val Asp Tyr Leu Thr Val
< lacI

tggtttcacattcaccaccctgaattgactctcttccgggcgctatcatgccataccgcgaaaggttttgcgccattcgatggtgtcc   3'  ••• 7558
accaaagtgtaagtggtgggacttaactgagagaaggcccgcgatagtacggtatggcgctttccaaaacgcggtaagctaccacagg   5'
          1
Pro Lys Met
< lacI   < lacI promoter
```

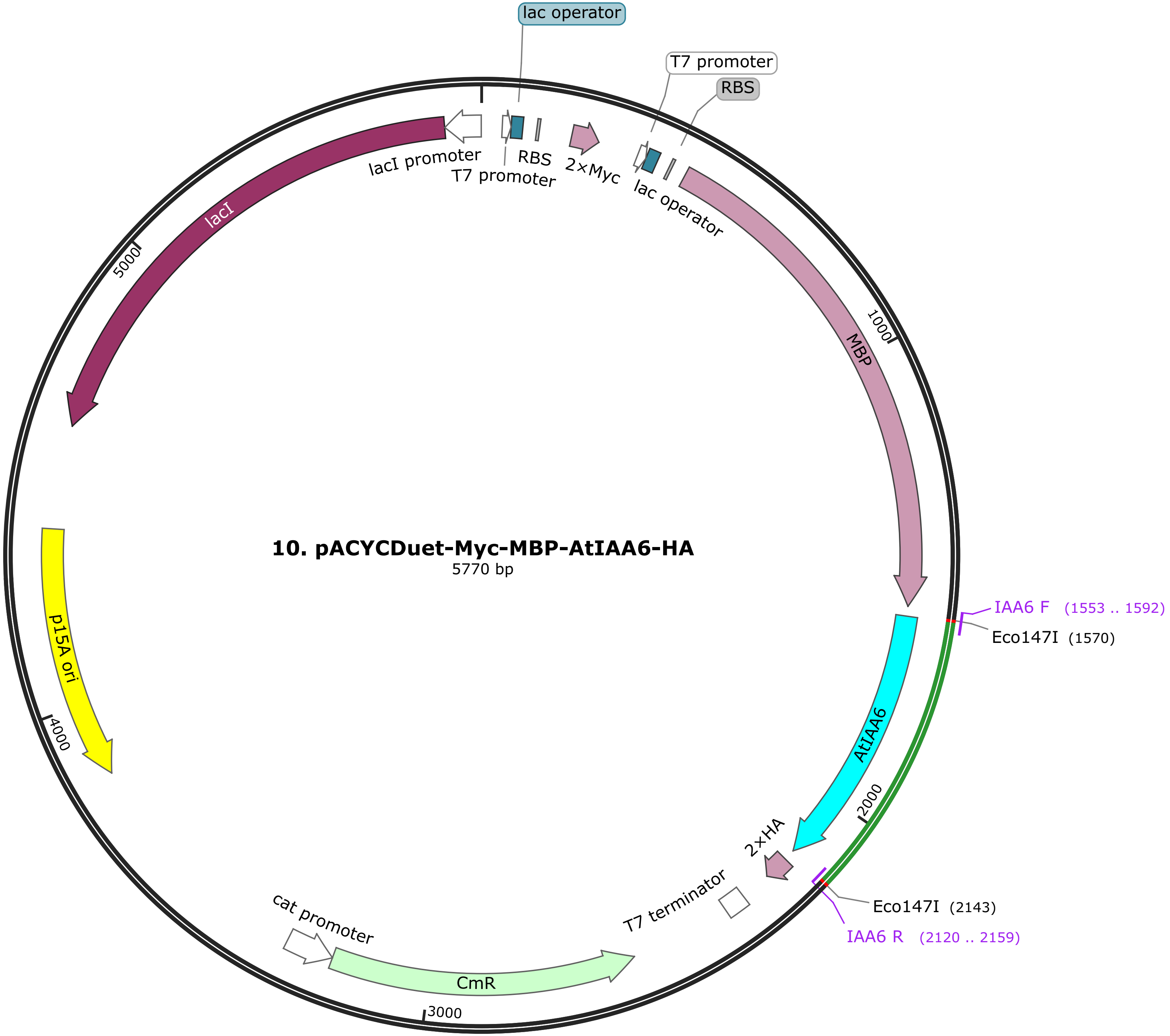
Created with SnapGene®
10. pACYCDuet-Myc-MBP-AtIAA6-HA
5770 bp
lac operator
T7 promoter
RBS
lacI promoter
T7 promoter
RBS
2×Myc
lac operator
lacI
5000
1000
MBP
IAA6 F (1553 .. 1592)
Eco147I (1570)
p15A ori
4000
AtIAA6
2000
2×HA
Eco147I (2143)
IAA6 R (2120 .. 2159)
T7 terminator
cat promoter
CmR
3000

Sequence: 10. pACYCDuet-Myc-MBP-AtIAA6-HA.dna (Circular / 5770 bp)
Enzymes: < Unsaved Enzyme Set > (1 of 678 total)
Features: 18 total
Primers: 5 total

Unique Cutters **Bold**

5′ gggatctcgacgctctcccttatgcgactcctgcattaggaaattaatacgactcactataggggaattgtgagcggataacaattcccc 90
3′ ccctagagctgcgagagggaatacgctgaggacgtaatcctttaattatgctgagtgatatccccttaacactcgcctattgttaagggg

T7 promoter | lac operator

tgtagaaataattttgtttaactttaatAAGGAGatataccatgggcagcagcgcatcaccatcatcaccaagccaggGatccGAGCTCG 180
acatctttattaaaacaaattgaaattaTTCCTCtatatggtacccgtcgtcgcgtagtggtagtagtggttcggtccCtaggCTCGAGC

RBS

ATATCACTAGTCCTGAGCAGAAGCTCATCTCCGAGGAGGACCTCGAGCAGAAGCTCATCTCCGAGGAGGACCTCCTGActgcaggtcgac 270
TATAGTGATCAGGACTCGTCTTCGAGTAGAGGCTCCTCCTGGAGCTCGTCTTCGAGTAGAGGCTCCTCCTGGAGGACTgacgtccagctg

1 5 10 15 20
Glu Gln Lys Leu Ile Ser Glu Glu Asp Leu Glu Gln Lys Leu Ile Ser Glu Glu Asp Leu Leu Thr Ala Gly Arg
2×Myc

aagcttgcggccgcataatgcttaagtcgaacagaaagtaatcgtattgtacacggccgcataatcgaaattaatacgactcactatagg 360
ttcgaacgccggcgtattacgaattcagcttgtctttcattagcataacatgtgccggcgtattagctttaattatgctgagtgatatcc

Gln Ala Cys Gly Arg Ile Met Leu Lys Ser Asn Arg Lys *
(in frame with 2×Myc)
T7 promoter

ggaattgtgagcggataacaattccccatcttagtatattagttaagtataagaaggagatatacatatggcagatctcaattgggaatt 450
ccttaacactcgcctattgttaaggggtagaatcatataatcaattcatattcttcctctatatgtataccgtctagagttaacccttaa

lac operator | RBS

catgaaaatcgaagaaggtaaactggtaatctggattaacggcgataaaggctataacggtctcgctgaagtcggtaagaaattcgagaa 540
gtacttttagcttcttccatttgaccattagacctaattgccgctatttccgatattgccagagcgacttcagccattctttaagctctt

1 5 10 15 20 25 30
Met Lys Ile Glu Glu Gly Lys Leu Val Ile Trp Ile Asn Gly Asp Lys Gly Tyr Asn Gly Leu Ala Glu Val Gly Lys Lys Phe Glu Lys
MBP

agataccggaattaaagtcaccgttgagcatccggataaactggaagagaaattcccacaggttgcggcaactggcgatggccctgacat 630
tctatggccttaatttcagtggcaactcgtaggcctatttgaccttctctttaagggtgtccaacgccgttgaccgctaccgggactgta

35 40 45 50 55 60
Asp Thr Gly Ile Lys Val Thr Val Glu His Pro Asp Lys Leu Glu Glu Lys Phe Pro Gln Val Ala Ala Thr Gly Asp Gly Pro Asp Ile
MBP

tatcttctgggcacacgaccgctttggtggctacgctcaatctggcctgttggctgaaatcaccccggacaaagcgttccaggacaagct 720
atagaagacccgtgtgctggcgaaaccaccgatgcgagttagaccggacaaccgactttagtggggcctgtttcgcaaggtcctgttcga

65 70 75 80 85 90
Ile Phe Trp Ala His Asp Arg Phe Gly Gly Tyr Ala Gln Ser Gly Leu Leu Ala Glu Ile Thr Pro Asp Lys Ala Phe Gln Asp Lys Leu
MBP

gtatccgtttacctgggatgccgtacgttacaacggcaagctgattgcttacccgatcgctgttgaagcgttatcgctgatttataacaa 810
cataggcaaatggaccctacggcatgcaatgttgccgttcgactaacgaatgggctagcgacaacttcgcaatagcgactaaatattgtt

95 100 105 110 115 120
Tyr Pro Phe Thr Trp Asp Ala Val Arg Tyr Asn Gly Lys Leu Ile Ala Tyr Pro Ile Ala Val Glu Ala Leu Ser Leu Ile Tyr Asn Lys
MBP

agatctgctgccgaacccgccaaaaacctgggaagagatcccggcgctggataaagaactgaaagcgaaaggtaagagcgcgctgatgtt 900
tctagacgacggcttgggcggtttttggacccttctctagggccgcgacctatttcttgactttcgctttccattctcgcgcgactacaa

125 130 135 140 145 150
Asp Leu Leu Pro Asn Pro Pro Lys Thr Trp Glu Glu Ile Pro Ala Leu Asp Lys Glu Leu Lys Ala Lys Gly Lys Ser Ala Leu Met Phe
MBP

caacctgcaagaaccgtacttcacctggccgctgattgctgctgacggggttatgcgttcaagtatgaaaacggcaagtacgacattaa 990
gttggacgttcttggcatgaagtggaccggcgactaacgacgactgccccaatacgcaagttcatacttttgccgttcatgctgtaatt

155 160 165 170 175 180
Asn Leu Gln Glu Pro Tyr Phe Thr Trp Pro Leu Ile Ala Ala Asp Gly Gly Tyr Ala Phe Lys Tyr Glu Asn Gly Lys Tyr Asp Ile Lys
MBP

```
agacgtgggcgtggataacgctggcgcgaaagcgggtctgaccttcctggttgacctgattaaaaacaaacacatgaatgcagacaccga 1080
tctgcacccgcacctattgcgaccgcgctttcgcccagactggaaggaccaactggactaatttttgtttgtgtacttacgtctgtggct
185 190 195 200 205 210
Asp Val Gly Val Asp Asn Ala Gly Ala Lys Ala Gly Leu Thr Phe Leu Val Asp Leu Ile Lys Asn Lys His Met Asn Ala Asp Thr Asp
MBP

ttactccatcgcagaagctgcctttaataaaggcgaaacagcgatgaccatcaacggcccgtgggcatggtccaacatcgacaccagcaa 1170
aatgaggtagcgtcttcgacggaaattatttccgctttgtcgctactggtagttgccgggcacccgtaccaggttgtagctgtggtcgtt
215 220 225 230 235 240
Tyr Ser Ile Ala Glu Ala Ala Phe Asn Lys Gly Glu Thr Ala Met Thr Ile Asn Gly Pro Trp Ala Trp Ser Asn Ile Asp Thr Ser Lys
MBP

agtgaattatggtgtaacggtactgccgaccttcaagggtcaaccatccaaaccgttcgttggcgtgctgagcgcaggtattaacgccgc 1260
tcacttaataccacattgccatgacggctggaagttcccagttggtaggtttggcaagcaaccgcacgactcgcgtccataattgcggcg
245 250 255 260 265 270
Val Asn Tyr Gly Val Thr Val Leu Pro Thr Phe Lys Gly Gln Pro Ser Lys Pro Phe Val Gly Val Leu Ser Ala Gly Ile Asn Ala Ala
MBP

cagtccgaacaaagagctggcaaaagagttcctcgaaaactatctgctgactgatgaaggtctggaagcggttaataaagacaaaccgct 1350
gtcaggcttgtttctcgaccgttttctcaaggagcttttgatagacgactgactacttccagaccttcgccaattatttctgtttggcga
275 280 285 290 295 300
Ser Pro Asn Lys Glu Leu Ala Lys Glu Phe Leu Glu Asn Tyr Leu Leu Thr Asp Glu Gly Leu Glu Ala Val Asn Lys Asp Lys Pro Leu
MBP

gggtgccgtagcgctgaagtcttacgaggaagagttggcgaaagatccacgtattgccgccactatggaaaacgcccagaaaggtgaaat 1440
cccacggcatcgcgacttcagaatgctccttctcaaccgctttctaggtgcataacggcggtgataccttttgcgggtctttccacttta
305 310 315 320 325 330
Gly Ala Val Ala Leu Lys Ser Tyr Glu Glu Glu Leu Ala Lys Asp Pro Arg Ile Ala Ala Thr Met Glu Asn Ala Gln Lys Gly Glu Ile
MBP

catgccgaacatcccgcagatgtccgctttctggtatgccgtgcgtactgcggtgatcaacgccgccagcggtcgtcagactgtcgatga 1530
gtacggcttgtagggcgtctacaggcgaaagaccatacggcacgcatgacgccactagttgcggcggtcgccagcagtctgacagctact
335 340 345 350 355 360
Met Pro Asn Ile Pro Gln Met Ser Ala Phe Trp Tyr Ala Val Arg Thr Ala Val Ile Asn Ala Ala Ser Gly Arg Gln Thr Val Asp Glu
MBP

Eco147I
IAA6 F
gaattcgagCCCGGGAGGCCTATGGCAAAGGAAGGTCTAG
agccctgaaagacgcgcagactgaattcgagCCCGGGAGGCCTATGGCAAAGGAAGGTCTAGCACTCGAGATCACAGAGCTTCGATTGGG 1620
tcgggactttctgcgcgtctgacttaagctcGGGCCCTCCGGATACCGTTTCCTTCCAGATCGTGAGCTCTAGTGTCTCGAAGCTAACCC
365 1 5 10 15
Ala Leu Lys Asp Ala Gln Thr Glu Phe Glu Pro Gly Arg Pro Met Ala Lys Glu Gly Leu Ala Leu Glu Ile Thr Glu Leu Arg Leu Gly
MBP (in frame with MBP) AtIAA6

TCTTCCAGGAGATAATTATAGCGAAATATCAGTATGCGGATCGAGTAAGAAGAAGAAGAGGGTGCTCTCGGATATGATGACCTCATCAGC 1710
AGAAGGTCCTCTATTAATATCGCTTTATAGTCATACGCCTAGCTCATTCTTCTTCTTCTCCCACGAGAGCCTATACTACTGGAGTAGTCG
20 25 30 35 40 45
Leu Pro Gly Asp Asn Tyr Ser Glu Ile Ser Val Cys Gly Ser Ser Lys Lys Lys Lys Arg Val Leu Ser Asp Met Met Thr Ser Ser Ala
AtIAA6

GTTAGATACTGAGAATGAAAACAGCGTCGTTTCATCAGTTGAAGATGAATCACTGCCGGTTGTGAAGAGTCAAGCGGTGGGATGGCCACC 1800
CAATCTATGACTCTTACTTTTGTCGCAGCAAAGTAGTCAACTTCTACTTAGTGACGGCCAACACTTCTCAGTTCGCCACCCTACCGGTGG
50 55 60 65 70 75
Leu Asp Thr Glu Asn Glu Asn Ser Val Val Ser Ser Val Glu Asp Glu Ser Leu Pro Val Val Lys Ser Gln Ala Val Gly Trp Pro Pro
AtIAA6

TGTGTGTTCTTACAGGAGAAAGAAGAACAATGAGGAAGCATCGAAAGCTATAGGCTACGTGAAAGTGAGCATGGATGGTGTGCCATACAT 1890
ACACACAAGAATGTCCTCTTTCTTCTTGTTACTCCTTCGTAGCTTTCGATATCCGATGCACTTTCACTCGTACCTACCACACGGTATGTA
80 85 90 95 100 105
Val Cys Ser Tyr Arg Arg Lys Lys Asn Asn Glu Glu Ala Ser Lys Ala Ile Gly Tyr Val Lys Val Ser Met Asp Gly Val Pro Tyr Met
AtIAA6
```

GAGGAAGATTGACCTTGGTTCGAGCAACAGTTATATTAATCTAGTCACGGTTCTTGAGAATCTCTTCGGCTGTCTTGGCATAGGAGTGGC 1980

CTCCTTCTAACTGGAACCAAGCTCGTTGTCAATATAATTAGATCAGTGCCAAGAACTCTTAGAGAAGCCGACAGAACCGTATCCTCACCG

110 115 120 125 130 135

Arg Lys Ile Asp Leu Gly Ser Ser Asn Ser Tyr Ile Asn Leu Val Thr Val Leu Glu Asn Leu Phe Gly Cys Leu Gly Ile Gly Val Ala

AtIAA6

GAAGGAGGGTAAGAAGTGTGAATACATTATTATATACGAAGACAAGGATAGAGACTGGATGCTCGTCGGAGATGTACCTTGGCAGATGTT 2070

CTTCCTCCCATTCTTCACACTTATGTAATAATATATGCTTCTGTTCCTATCTCTGACCTACGAGCAGCCTCTACATGGAACCGTCTACAA

140 145 150 155 160 165

Lys Glu Gly Lys Lys Cys Glu Tyr Ile Ile Ile Tyr Glu Asp Lys Asp Arg Asp Trp Met Leu Val Gly Asp Val Pro Trp Gln Met Phe

AtIAA6

Eco147I

TAAAGAATCATGCAAGAGGCTGAGGATCGTGAAGAGATCAGATGCAACTGGTTTTGGTCTCCAGCAAGATAGGCCTctcggcgcgcctgc 2160

ATTTCTTAGTACGTTCTCCGACTCCTAGCACTTCTCTAGTCTACGTTGACCAAAACCAGAGGTCGTTCTATCCGGAgagccgcgcggacg

170 175 180 185

Lys Glu Ser Cys Lys Arg Leu Arg Ile Val Lys Arg Ser Asp Ala Thr Gly Phe Gly Leu Gln Gln Asp Arg Pro Leu Gly Ala Pro Ala

AtIAA6 (in frame with AtIAA6)

CCAAAACCAGAGGTCGTTCTATCCGGAgagccgcgcggac

IAA6 R

aggtTACCCATACGACGTACCAGATTACGCTTACCCATACGACGTACCAGATTACGCTcgacaagcttgcggccgcataatgcttaagta 2250

tccaATGGGTATGCTGCATGGTCTAATGCGAATGGGTATGCTGCATGGTCTAATGCGAgctgttcgaacgccggcgtattacgaattcat

1 5 10 15

Gly Tyr Pro Tyr Asp Val Pro Asp Tyr Ala Tyr Pro Tyr Asp Val Pro Asp Tyr Ala Arg Gln Ala Cys Gly Arg Ile Met Leu Lys *

2×HA (in frame with 2×HA)

attaacctaggctgctgccaccgctgagcaataactagcataaccccttggggcctctaaacgggtcttgaggggttttttgctgaaacc 2340

taattggatccgacgacggtggcgactcgttattgatcgtattggggaaccccggagatttgcccagaactccccaaaaaacgactttgg

T7 terminator

tcaggcatttgagaagcacacggtcacactgcttccggtagtcaataaaccggtaaaccagcaatagacataagcggctatttaacgacc 2430

agtccgtaaactcttcgtgtgccagtgtgacgaaggccatcagttatttggccatttggtcgttatctgtattcgccgataaattgctgg

ctgccctgaaccgacgaccgggtcgaatttgctttcgaatttctgccattcatccgcttattatcacttattcaggcgtagcaccaggcg 2520

gacgggacttggctgctggcccagcttaaacgaaagcttaaagacggtaagtaggcgaataatagtgaataagtccgcatcgtggtccgc

tttaagggcaccaataactgccttaaaaaaattacgccccgccctgccactcatcgcagtactgttgtaattcattaagcattctgccga 2610

aaattcccgtggttattgacggaatttttttaatgcggggcgggacggtgagtagcgtcatgacaacattaagtaattcgtaagacggct

220 215 210 205

* Ala Gly Gly Gln Trp Glu Asp Cys Tyr Gln Gln Leu Glu Asn Leu Met Arg Gly Val

CmR

catggaagccatcacagacggcatgatgaacctgaatcgccagcggcatcagcaccttgtcgccttgcgtataatatttgcccatagtga 2700

gtaccttcggtagtgtctgccgtactacttggacttagcggtcgccgtagtcgtggaacagcggaacgcatattataaacgggtatcact

200 195 190 185 180 175

His Phe Gly Asp Cys Val Ala His His Val Gln Ile Ala Leu Pro Met Leu Val Lys Asp Gly Gln Thr Tyr Tyr Lys Gly Met Thr Phe

CmR

aaacgggggcgaagaagttgtccatattggccacgtttaaatcaaaactggtgaaactcacccagggattggctgagacgaaaaacatat 2790

tttgcccccgcttcttcaacaggtataaccggtgcaaatttagttttgaccactttgagtgggtccctaaccgactctgctttttgtata

170 165 160 155 150 145

Val Pro Ala Phe Phe Asn Asp Met Asn Ala Val Asn Leu Asp Phe Ser Thr Phe Ser Val Trp Pro Asn Ala Ser Val Phe Phe Met Asn

CmR

tctcaataaaccctttagggaaataggccaggttttcaccgtaacacgccacatcttgcgaatatatgtgtagaaactgccggaaatcgt 2880

agagttatttgggaaatccctttatccggtccaaaagtggcattgtgcggtgtagaacgcttatatacacatctttgacggcctttagca

140 135 130 125 120 115

Glu Ile Phe Gly Lys Pro Phe Tyr Ala Leu Asn Glu Gly Tyr Cys Ala Val Asp Gln Ser Tyr Ile His Leu Phe Gln Arg Phe Asp Asp

CmR

```
cgtggtattcactccagagcgatgaaaacgtttcagtttgctcatggaaaacggtgtaacaagggtgaacactatcccatatcaccagct   2970
gcaccataagtgaggtctcgctacttttgcaaagtcaaacgagtaccttttgccacattgttcccacttgtgatagggtatagtggtcga
110 105 100 95 90 85
His Tyr Glu Ser Trp Leu Ser Ser Phe Thr Glu Thr Gln Glu His Phe Val Thr Tyr Cys Pro His Val Ser Asp Trp Ile Val Leu Glu
CmR

caccgtctttcattgccatacggaactccggatgagcattcatcaggcgggcaagaatgtgaataaaggccggataaaacttgtgcttat   3060
gtggcagaaagtaacggtatgccttgaggcctactcgtaagtagtccgcccgttcttacacttatttccggcctattttgaacacgaata
80 75 70 65 60 55
Gly Asp Lys Met Ala Met Arg Phe Glu Pro His Ala Asn Met Leu Arg Ala Leu Ile His Ile Phe Ala Pro Tyr Phe Lys His Lys Asn
CmR

tttctttacggtctttaaaaaggccgtaatatccagctgaacggtctggttataggtacattgagcaactgactgaaatgcctcaaaat   3150
aaaagaaatgccagaaatttttccggcattataggtcgacttgccagaccaatatccatgtaactcgttgactgactttacggagtttta
50 45 40 35 30 25
Lys Lys Val Thr Lys Leu Phe Ala Thr Ile Asp Leu Gln Val Thr Gln Asn Tyr Thr Cys Gln Ala Val Ser Gln Phe Ala Glu Phe His
CmR

gttctttacgatgccattgggatatatcaacggtggtatatccagtgatttttttctccattttagcttccttagctcctgaaaatctcg   3240
caagaaatgctacggtaaccctatatagttgccaccatataggtcactaaaaaaagaggtaaaatcgaaggaatcgaggacttttagagc
20 15 10 5 1
Glu Lys Arg His Trp Gln Ser Ile Asp Val Thr Thr Tyr Gly Thr Ile Lys Lys Glu Met
CmR    cat promoter

ataactcaaaaaatacgcccggtagtgatcttatttcattatggtgaaagttggaacctcttacgtgccgatcaacgtctcattttcgcc   3330
tattgagttttttatgcgggccatcactagaataaagtaataccactttcaaccttggagaatgcacggctagttgcagagtaaaagcgg
cat promoter

aaaagttggcccagggcttcccggtatcaacagggacaccaggatttatttattctgcgaagtgatcttccgtcacaggtatttattcgg   3420
ttttcaaccgggtcccgaagggccatagttgtccctgtggtcctaaataaataagacgcttcactagaaggcagtgtccataaataagcc

cgcaaagtgcgtcgggtgatgctgccaacttactgatttagtgtatgatggtgtttttgaggtgctccagtggcttctgtttctatcagc   3510
gcgtttcacgcagcccactacgacggttgaatgactaaatcacatactaccacaaaaactccacgaggtcaccgaagacaaagatagtcg

tgtccctcctgttcagctactgacggggtggtgcgtaacggcaaaagcaccgccggacatcagcgctagcggagtgtatactggcttact   3600
acagggaggacaagtcgatgactgccccaccacgcattgccgttttcgtggcggcctgtagtcgcgatcgcctcacatatgaccgaatga

atgttggcactgatgagggtgtcagtgaagtgcttcatgtggcaggagaaaaaaggctgcaccggtgcgtcagcagaatatgtgatacag   3690
tacaaccgtgactactcccacagtcacttcacgaagtacaccgtcctctttttttccgacgtggccacgcagtcgtcttatacactatgtc

gatatattccgcttcctcgctcactgactcgctacgctcggtcgttcgactgcggcgagcggaaatggcttacgaacggggcggagattt   3780
ctatataaggcgaaggagcgagtgactgagcgatgcgagccagcaagctgacgccgctcgcctttaccgaatgcttgccccgcctctaaa

cctggaagatgccaggaagatacttaacagggaagtgagagggccgcggcaaagccgtttttccataggctccgcccccctgacaagcat   3870
ggaccttctacggtccttctatgaattgtcccttcactctcccggcgccgtttcggcaaaaaggtatccgaggcggggggactgttcgta
p15A ori

cacgaaatctgacgctcaaatcagtggtggcgaaacccgacaggactataaagataccaggcgtttccccctggcggctccctcgtgcgct   3960
gtgctttagactgcgagtttagtcaccaccgctttgggctgtcctgatatttctatggtccgcaaagggggaccgccgagggagcacgcga
p15A ori

ctcctgttcctgcctttcggtttaccggtgtcattccgctgttatggccgcgtttgtctcattccacgcctgacactcagttccgggtag   4050
gaggacaaggacggaaagccaaatggccacagtaaggcgacaataccggcgcaaacagagtaaggtgcggactgtgagtcaaggcccatc
p15A ori
```

```
gcagttcgctccaagctggactgtatgcacgaaccccccgttcagtccgaccgctgcgccttatccggtaactatcgtcttgagtccaac   4140
cgtcaagcgaggttcgacctgacatacgtgcttggggggcaagtcaggctggcgacgcggaataggccattgatagcagaactcaggttg
< p15A ori

ccggaaagacatgcaaaagcaccactggcagcagccactggtaattgatttagaggagttagtcttgaagtcatgcgccggttaaggcta   4230
ggcctttctgtacgttttcgtggtgaccgtcgtcggtgaccattaactaaatctcctcaatcagaacttcagtacgcggccaattccgat
< p15A ori

aactgaaaggacaagttttggtgactgcgctcctccaagccagttacctcggttcaaagagttggtagctcagagaaccttcgaaaaacc   4320
ttgactttcctgttcaaaaccactgacgcgaggaggttcggtcaatggagccaagtttctcaaccatcgagtctcttggaagctttttgg
< p15A ori

gccctgcaaggcggttttttcgttttcagagcaagagattacgcgcagaccaaaacgatctcaagaagatcatcttattaatcagataaa   4410
cgggacgttccgccaaaaaagcaaaagtctcgttctctaatgcgcgtctggttttgctagagttcttctagtagaataattagtctattt
< p15A ori

atatttctagatttcagtgcaatttatctcttcaaatgtagcacctgaagtcagccccatacgatataagttgtaattctcatgttagtc   4500
tataaagatctaaagtcacgttaaatagagaagtttacatcgtggacttcagtcggggtatgctatattcaacattaagagtacaatcag

atgccccgcgcccaccggaaggagctgactgggttgaaggctctcaagggcatcggtcgagatcccggtgcctaatgagtgagctaactt   4590
tacggggcgcgggtggccttcctcgactgacccaacttccgagagttcccgtagccagctctagggccacggattactcactcgattgaa

acattaattgcgttgcgctcactgcccgctttccagtcgggaaacctgtcgtgccagctgcattaatgaatcggccaacgcgcggggaga   4680
tgtaattaacgcaacgcgagtgacgggcgaaaggtcagccctttggacagcacggtcgacgtaattacttagccggttgcgcgcccctct
    360                 355                 350                 345                 340
*  Gln  Gly  Ser  Glu  Leu  Arg  Ser  Val  Gln  Arg  Ala  Leu  Gln  Met  Leu  Ser  Asp  Ala  Leu  Ala  Arg  Pro  Ser
< lacI

ggcggtttgcgtattgggcgccagggtggtttttcttttcaccagtgagacgggcaacagctgattgcccttcaccgcctggccctgaga   4770
ccgccaaacgcataacccgcggtcccaccaaaaagaaaagtggtcactctgcccgttgtcgactaacgggaagtggcggaccgggactct
          335                 330                 325                 320                 315                 310
Ala  Thr  Gln  Thr  Asn  Pro  Ala  Leu  Thr  Thr  Lys  Arg  Lys  Val  Leu  Ser  Val  Pro  Leu  Leu  Gln  Asn  Gly  Lys  Val  Ala  Gln  Gly  Gln  Ser
< lacI

gagttgcagcaagcggtccacgctggtttgccccagcaggcgaaaatcctgtttgatggtggttaacggcgggatataacatgagctgtc   4860
ctcaacgtcgttcgccaggtgcgaccaaacggggtcgtccgcttttaggacaaactaccaccaattgccgccctatattgtactcgacag
          305                 300                 295                 290                 285                 280
Leu  Gln  Leu  Leu  Arg  Asp  Val  Ser  Thr  Gln  Gly  Leu  Leu  Arg  Phe  Asp  Gln  Lys  Ile  Thr  Thr  Leu  Pro  Pro  Ile  Tyr  Cys  Ser  Ser  Asp
< lacI

ttcggtatcgtcgtatcccactaccgagatgtccgcaccaacgcgcagcccggactcggtaatggcgcgcattgcgcccagcgccatctg   4950
aagccatagcagcatagggtgatggctctacaggcgtggttgcgcgtcgggcctgagccattaccgcgcgtaacgcgggtcgcggtagac
          275                 270                 265                 260                 255                 250
Glu  Thr  Asp  Asp  Tyr  Gly  Val  Val  Ser  Ile  Asp  Ala  Gly  Val  Arg  Leu  Gly  Ser  Glu  Thr  Ile  Ala  Arg  Met  Ala  Gly  Leu  Ala  Met  Gln
< lacI

atcgttggcaaccagcatcgcagtgggaacgatgccctcattcagcatttgcatggtttgttgaaaaccggacatggcactccagtcgcc   5040
tagcaaccgttggtcgtagcgtcacccttgctacgggagtaagtcgtaaacgtaccaaacaacttttggcctgtaccgtgaggtcagcgg
          245                 240                 235                 230                 225                 220
Asp  Asn  Ala  Val  Leu  Met  Ala  Thr  Pro  Val  Ile  Gly  Glu  Asn  Leu  Met  Gln  Met  Thr  Gln  Gln  Phe  Gly  Ser  Met  Ala  Ser  Trp  Asp  Gly
< lacI

ttcccgttccgctatcggctgaatttgattgcgagtgagatatttatgccagccagccagacgcagacgcgccgagacagaacttaatgg   5130
aagggcaaggcgatagccgacttaaactaacgctcactctataaatacggtcggtcggtctgcgtctgcgcggctctgtcttgaattacc
          215                 210                 205                 200                 195                 190
Glu  Arg  Glu  Ala  Ile  Pro  Gln  Ile  Gln  Asn  Arg  Thr  Leu  Tyr  Lys  His  Trp  Gly  Ala  Leu  Arg  Leu  Arg  Ala  Ser  Val  Ser  Ser  Leu  Pro
< lacI
```

gcccgctaacagcgcgatttgctggtgacccaatgcgaccagatgctccacgcccagtcgcgtaccgtcttcatgggagaaaataatact 5220
cgggcgattgtcgcgctaaacgaccactgggttacgctggtctacgaggtgcgggtcagcgcatggcagaagtaccctcttttattatga
185 180 175 170 165 160
Gly Ala Leu Leu Ala Ile Gln Gln His Gly Leu Ala Val Leu His Glu Val Gly Leu Arg Thr Gly Asp Glu His Ser Phe Ile Ile Ser
lacI

gttgatgggtgtctggtcagagacatcaagaaataacgccggaacattagtgcaggcagcttccacagcaatggcatcctggtcatccag 5310
caactacccacagaccagtctctgtagttctttattgcggccttgtaatcacgtccgtcgaaggtgtcgttaccgtaggaccagtaggtc
155 150 145 140 135 130
Asn Ile Pro Thr Gln Asp Ser Val Asp Leu Phe Leu Ala Pro Val Asn Thr Cys Ala Ala Glu Val Ala Ile Ala Asp Gln Asp Asp Leu
lacI

cggatagttaatgatcagcccactgacgcgttgcgcgagaagattgtgcaccgccgctttacaggcttcgacgccgcttcgttctaccat 5400
gcctatcaattactagtcgggtgactgcgcaacgcgctcttctaacacgtggcggcgaaatgtccgaagctgcggcgaagcaagatggta
125 120 115 110 105 100
Pro Tyr Asn Ile Ile Leu Gly Ser Val Arg Gln Ala Leu Leu Asn His Val Ala Ala Lys Cys Ala Glu Val Gly Ser Arg Glu Val Met
lacI

cgacaccaccacgctggcacccagttgatcggcgcgagatttaatcgccgcgacaatttgcgacggcgcgtgcagggccagactggaggt 5490
gctgtggtggtgcgaccgtgggtcaactagccgcgctctaaattagcggcgctgttaaacgctgccgcgcacgtcccggtctgacctcca
95 90 85 80 75 70
Ser Val Val Val Ser Ala Gly Leu Gln Asp Ala Arg Ser Lys Ile Ala Ala Val Ile Gln Ser Pro Ala His Leu Ala Leu Ser Ser Thr
lacI

ggcaacgccaatcagcaacgactgtttgcccgccagttgttgtgccacgcggttgggaatgtaattcagctccgccatcgccgcttccac 5580
ccgttgcggttagtcgttgctgacaaacgggcggtcaacaacacggtgcgccaacccttacattaagtcgaggcggtagcggcgaaggtg
65 60 55 50 45 40
Ala Val Gly Ile Leu Leu Ser Gln Lys Gly Ala Leu Gln Gln Ala Val Arg Asn Pro Ile Tyr Asn Leu Glu Ala Met Ala Ala Glu Val
lacI

tttttcccgcgttttcgcagaaacgtggctggcctggttcaccacgcgggaaacggtctgataagagacaccggcatactctgcgacatc 5670
aaaaagggcgcaaaagcgtctttgcaccgaccggaccaagtggtgcgccctttgccagactattctctgtggccgtatgagacgctgtag
35 30 25 20 15 10
Lys Glu Arg Thr Lys Ala Ser Val His Ser Ala Gln Asn Val Val Arg Ser Val Thr Gln Tyr Ser Val Gly Ala Tyr Glu Ala Val Asp
lacI

gtataacgttactggtttcacattcaccaccctgaattgactctcttccgggcgctatcatgccataccgcgaaaggttttgcgccattc 5760
catattgcaatgaccaaagtgtaagtggtgggacttaactgagagaaggcccgcgatagtacggtatggcgctttccaaaacgcggtaag
5 1
Tyr Leu Thr Val Pro Lys Met
lacI
lacI promoter

gatggtgtcc 3′
••• 5770
ctaccacagg 5′
lacI promoter

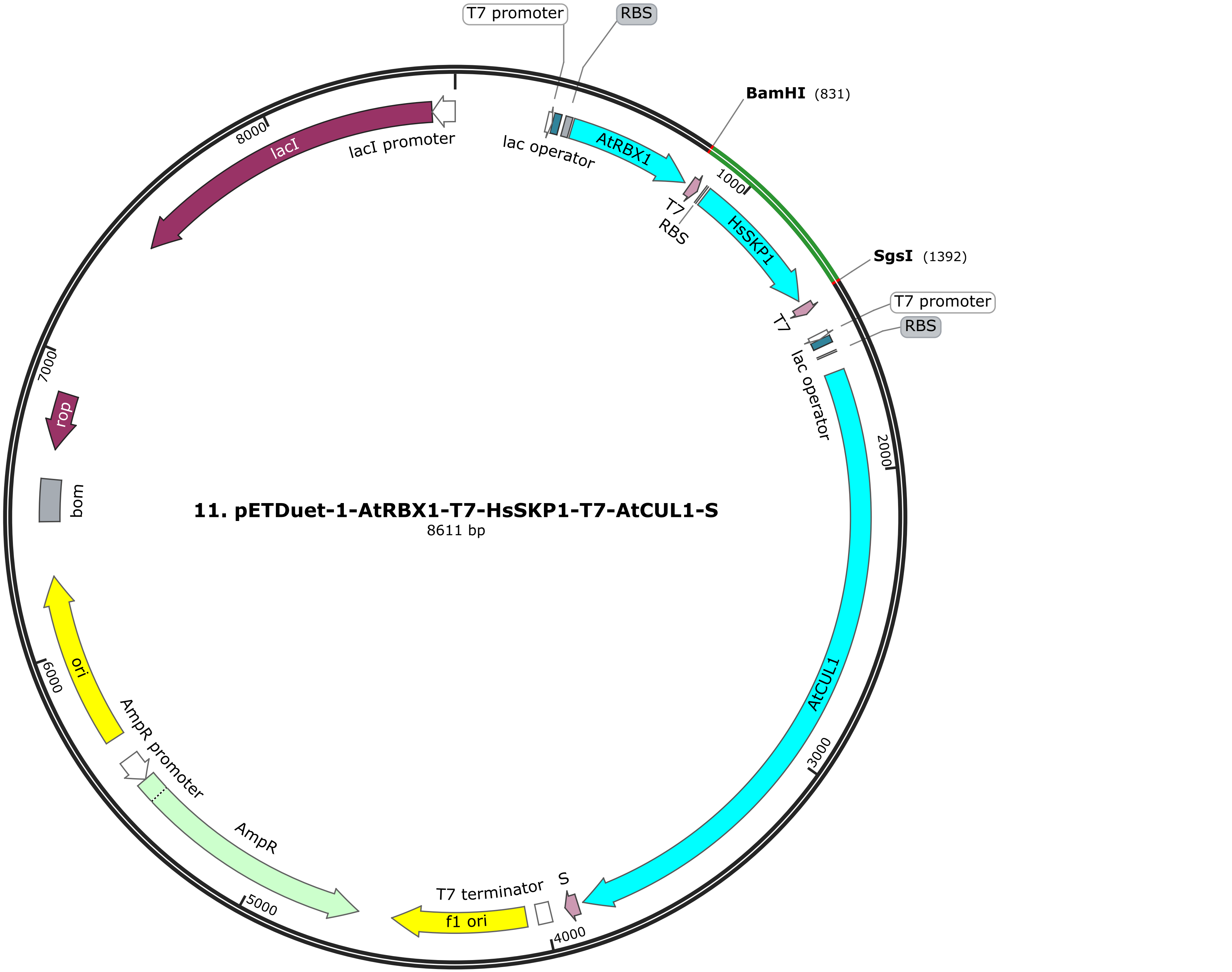
Created with SnapGene®
11. pETDuet-1-AtRBX1-T7-HsSKP1-T7-AtCUL1-S
8611 bp
T7 promoter
RBS
BamHI (831)
SgsI (1392)
T7 promoter
RBS
lac operator
AtRBX1
T7
RBS
HsSKP1
T7
lac operator
AtCUL1
S
T7 terminator
f1 ori
AmpR
AmpR promoter
ori
bom
rop
lacI
lacI promoter
1000
2000
3000
4000
5000
6000
7000
8000

Unique Cutters **Bold**

5′ cgggatctcgacgctctcccttatgcgactcctgcattaggaagcagcccagtagtaggttgaggccgttgagcaccgccgccgcaagga 90
3′ gccctagagctgcgagagggaatacgctgaggacgtaatccttcgtcgggtcatcatccaactccggcaactcgtggcggcggcgttcct

atggtgcatgcaaggagatggcgcccaacagtcccccggccacggggcctgccaccatacccacgccgaaacaagcgctcatgagcccga 180
taccacgtacgttcctctaccgcgggttgtcagggggccggtgccccggacggtggtatgggtgcggctttgttcgcgagtactcgggct

agtggcgagcccgatcttccccatcggtgatgtcggcgatataggcgccagcaaccgcacctgtggcgccggtgatgccggccacgatgc 270
tcaccgctcgggctagaaggggtagccactacagccgctatatccgcggtcgttggcgtggacaccgcggccactacggccggtgctacg

gtccggcgtagaggatcgagatcgatctcgatcccgcgaaattaatacgactcactataggggaattgtgagcggataacaattcccctc 360
caggccgcatctcctagctctagctagagctagggcgctttaattatgctgagtgatatccccttaacactcgcctattgttaaggggag
T7 promoter | lac operator

tagaaataattttgtttaactttaagaaggagatataccATGGCGACTCTAGACTCCGACGTTACCATGATTCCTGCCGGAGAAGCCTCC 450
atctttattaaaacaaattgaaattcttcctctatatggTACCGCTGAGATCTGAGGCTGCAATGGTACTAAGGACGGCCTCTTCGGAGG
1 5 10 15
Met Ala Thr Leu Asp Ser Asp Val Thr Met Ile Pro Ala Gly Glu Ala Ser
RBS | AtRBX1

AGCAGCGTAGCCGCGTCGTCTTCCAACAAGAAAGCTAAGCGATTCGAAATTAAGAAGTGGAGCGCCGTTGCTCTCTGGGCTTGGGATATC 540
TCGTCGCATCGGCGCAGCAGAAGGTTGTTCTTTCGATTCGCTAAGCTTTAATTCTTCACCTCGCGGCAACGAGAGACCCGAACCCTATAG
20 25 30 35 40 45
Ser Ser Val Ala Ala Ser Ser Ser Asn Lys Lys Ala Lys Arg Phe Glu Ile Lys Lys Trp Ser Ala Val Ala Leu Trp Ala Trp Asp Ile
AtRBX1

GTTGTTGACAACTGTGCGATCTGCAGAAACCACATCATGGATCTTTGTATCGAGTGTCAGGCTAATCAGGCCAGTGCCACAAGTGAAGAG 630
CAACAACTGTTGACACGCTAGACGTCTTTGGTGTAGTACCTAGAAACATAGCTCACAGTCCGATTAGTCCGGTCACGGTGTTCACTTCTC
50 55 60 65 70 75
Val Val Asp Asn Cys Ala Ile Cys Arg Asn His Ile Met Asp Leu Cys Ile Glu Cys Gln Ala Asn Gln Ala Ser Ala Thr Ser Glu Glu
AtRBX1

TGCACTGTAGCTTGGGAGGACGACCAAAATAACTGCAATAAGTATTTCTGTATTCTTGATTGCTCAATGAAAGATGACCACTTGGAGGGG 720
ACGTGACATCGAACCCTCCTGCTGGTTTTATTGACGTTATTCATAAAGACATAAGAACTAACGAGTTACTTTCTACTGGTGAACCTCCCC
80 85 90 95 100 105
Cys Thr Val Ala Trp Glu Asp Asp Gln Asn Asn Cys Asn Lys Tyr Phe Cys Ile Leu Asp Cys Ser Met Lys Asp Asp His Leu Glu Gly
AtRBX1

GTTTGCAATCACGCCTTCCACTTTCACTGCATCAGCAGATGGCTAAAGACTCGTCAAGTTTGTCCATTGGATAACAGTGAGTGGGAGTTT 810
CAAACGTTAGTGCGGAAGGTGAAAGTGACGTAGTCGTCTACCGATTTCTGAGCAGTTCAAACAGGTAACCTATTGTCACTCACCCTCAAA
110 115 120 125 130 135
Val Cys Asn His Ala Phe His Phe His Cys Ile Ser Arg Trp Leu Lys Thr Arg Gln Val Cys Pro Leu Asp Asn Ser Glu Trp Glu Phe
AtRBX1

BamHI
CAGAAATATGGTCACAgccaggatccgaattcgatggctagcatgactggtggacagcaaatgggtCCAtaaCCCCGAAAGAAGGAGATA 900
GTCTTTATACCAGTGtcggtcctaggcttaagctaccgatcgtactgaccacctgtcgtttacccaGGTattGGGGCTTTCTTCCTCTAT
140 1 5 10
Gln Lys Tyr Gly His Ser Gln Asp Pro Asn Ser Met Ala Ser Met Thr Gly Gly Gln Gln Met Gly Pro *
AtRBX1 (in frame with AtRBX1) T7 | RBS

TACATatgccttcaattaagttgcagagttctgatggagagatatttgaagttgatgtggaaattgccaaacaatctgtgactattaaga 990
ATGTAtacggaagttaattcaacgtctcaagactacctctctataaacttcaactacacctttaacggtttgttagacactgataattct
1 5 10 15 20 25
Met Pro Ser Ile Lys Leu Gln Ser Ser Asp Gly Glu Ile Phe Glu Val Asp Val Glu Ile Ala Lys Gln Ser Val Thr Ile Lys
HsSKP1

ccatgttggaagatttgggaatggatgatgaaggagatgatgacccagttcctctaccaaatgtgaatgcagcaatattaaaaaaggtca 1080
ggtacaaccttctaaacccttacctactacttcctctactactgggtcaaggagatggtttacacttacgtcgttataattttttccagt
30 35 40 45 50 55
Thr Met Leu Glu Asp Leu Gly Met Asp Asp Glu Gly Asp Asp Asp Pro Val Pro Leu Pro Asn Val Asn Ala Ala Ile Leu Lys Lys Val
HsSKP1

ttcagtggtgcacccaccacaaggatgaccctcctcctcctgaagatgatgagaacaaagaaaagcgaacagatgatatccctgtttggg 1170
aagtcaccacgtgggtggtgttcctactgggaggaggaggacttctactactcttgtttcttttcgcttgtctactatagggacaaaccc
60 65 70 75 80 85
Ile Gln Trp Cys Thr His His Lys Asp Asp Pro Pro Pro Pro Glu Asp Asp Glu Asn Lys Glu Lys Arg Thr Asp Asp Ile Pro Val Trp
HsSKP1

accaagaattcctgaaagttgaccaaggaacacttttttgaactcattctggctgcaaactacttagacatcaaaggtttgcttgatgtta 1260
tggttcttaaggactttcaactggttccttgtgaaaaacttgagtaagaccgacgtttgatgaatctgtagtttccaaacgaactacaat
90 95 100 105 110 115
Asp Gln Glu Phe Leu Lys Val Asp Gln Gly Thr Leu Phe Glu Leu Ile Leu Ala Ala Asn Tyr Leu Asp Ile Lys Gly Leu Leu Asp Val
HsSKP1

catgcaagactgttgccaatatgatcaaggggaaaactcctgaggagattcgcaagaccttcaatatcaaaaatgactttactgaagagg 1350
gtacgttctgacaacggttatactagttccccttttgaggactcctctaagcgttctggaagttatagtttttactgaaatgacttctcc
120 125 130 135 140 145
Thr Cys Lys Thr Val Ala Asn Met Ile Lys Gly Lys Thr Pro Glu Glu Ile Arg Lys Thr Phe Asn Ile Lys Asn Asp Phe Thr Glu Glu
HsSKP1

SgsI

aggaagcccaggtaggtagcacacagtttgtcttagctcggcgcgcctgcagatggctagcatgactggtggacagcaaatgggtgtcg 1440
tccttcgggtccatccatcgtgtgtcaaaacagaatcgagccgcgcggacgtctaccgatcgtactgaccacctgtcgtttacccacagc
150 155 160 1 5 10
Glu Gln Ala Gln Val Gly Ser Thr Gln Phe Cys Leu Ser Ser Ala Arg Leu Gln Met Ala Ser Met Thr Gly Gly Gln Gln Met Gly Val
HsSKP1 (in frame with HsSKP1) T7

acaagcttgcggccgcataatgcttaagtcgaacagaaagtaatcgtattgtacacggccgcataatcgaaattaatacgactcactata 1530
tgttcgaacgccggcgtattacgaattcagcttgtctttcattagcataacatgtgccggcgtattagctttaattatgctgagtgatat
Asp Lys Leu Ala Ala Ala *
(in frame with T7)
T7 promoter

ggggaattgtgagcggataacaattccccatcttagtatattagttaagtataagAAGGAGatatacatatggcagatctcaattggata 1620
ccccttaacactcgcctattgttaaggggtagaatcatataatcaattcatattcTTCCTCtatatgtataccgtctagagttaacctat
lac operator RBS
T7 promoter

tcggccggccacgcgatcgctgacgtcggtaccATGGAGCGCAAGACTATTGACTTGGAGCAAGGATGGGACTATATGCAGACTGGAATT 1710
agccggccggtgcgctagcgactgcagccatggTACCTCGCGTTCTGATAACTGAACCTCGTTCCTACCCTGATATACGTCTGACCTTAA
1 5 10 15
Met Glu Arg Lys Thr Ile Asp Leu Glu Gln Gly Trp Asp Tyr Met Gln Thr Gly Ile
AtCUL1

ACTAAGCTGAAACGGATTCTGGAAGGATTGAATGAGCCAGCATTTGACTCTGAGCAATATATGATGCTTTATACGACTATCTACAACATG 1800
TGATTCGACTTTGCCTAAGACCTTCCTAACTTACTCGGTCGTAAACTGAGACTCGTTATATACTACGAAATATGCTGATAGATGTTGTAC
20 25 30 35 40 45
Thr Lys Leu Lys Arg Ile Leu Glu Gly Leu Asn Glu Pro Ala Phe Asp Ser Glu Gln Tyr Met Met Leu Tyr Thr Thr Ile Tyr Asn Met
AtCUL1

TGCACCCAGAAACCTCCTCATGATTACTCACAGCAGCTTTATGACAAGTATCGGGAAGCATTTGAGGAATATATTAACTCAACAGTTTTG 1890
ACGTGGGTCTTTGGAGGAGTACTAATGAGTGTCGTCGAAATACTGTTCATAGCCCTTCGTAAACTCCTTATATAATTGAGTTGTCAAAAC
50 55 60 65 70 75
Cys Thr Gln Lys Pro Pro His Asp Tyr Ser Gln Gln Leu Tyr Asp Lys Tyr Arg Glu Ala Phe Glu Glu Tyr Ile Asn Ser Thr Val Leu
AtCUL1

CCTGCGTTGAGGGAGAAGCATGATGAGTTTATGCTGCGGGAGCTATTTAAAAGATGGTCAAACCATAAAGTAATGGTCAGATGGCTATCC 1980
GGACGCAACTCCCTCTTCGTACTACTCAAATACGACGCCCTCGATAAATTTTCTACCAGTTTGGTATTTCATTACCAGTCTACCGATAGG
80 85 90 95 100 105
Pro Ala Leu Arg Glu Lys His Asp Glu Phe Met Leu Arg Glu Leu Phe Lys Arg Trp Ser Asn His Lys Val Met Val Arg Trp Leu Ser
AtCUL1

CGCTTCTTCTACTACCTTGACCGTTACTTCATTGCTCGGAGATCACTTCCACCACTGAATGAAGTTGGCCTGACATGCTTCCGTGACCTG 2070
GCGAAGAAGATGATGGAACTGGCAATGAAGTAACGAGCCTCTAGTGAAGGTGGTGACTTACTTCAACCGGACTGTACGAAGGCACTGGAC
110 115 120 125 130 135
Arg Phe Phe Tyr Tyr Leu Asp Arg Tyr Phe Ile Ala Arg Arg Ser Leu Pro Pro Leu Asn Glu Val Gly Leu Thr Cys Phe Arg Asp Leu
AtCUL1

GTTTATAATGAGCTACATTCTAAGGTCAAACAAGCTGTAATAGCTCTTGTTGATAAAGAACGGGAGGGCGAGCAGATTGATAGGGCCCTG 2160
CAAATATTACTCGATGTAAGATTCCAGTTTGTTCGACATTATCGAGAACAACTATTTCTTGCCCTCCCGCTCGTCTAACTATCCCGGGAC
140 145 150 155 160 165
Val Tyr Asn Glu Leu His Ser Lys Val Lys Gln Ala Val Ile Ala Leu Val Asp Lys Glu Arg Glu Gly Glu Gln Ile Asp Arg Ala Leu
AtCUL1

CTGAAAAACGTATTAGATATCTATGTAGAGATTGGAATGGGGCAGATGGAGAGGTATGAAGAAGATTTTGAAAGCTTCATGCTTCAAGAT 2250
GACTTTTTGCATAATCTATAGATACATCTCTAACCTTACCCCGTCTACCTCTCCATACTTCTTCTAAAACTTTCGAAGTACGAAGTTCTA
170 175 180 185 190 195
Leu Lys Asn Val Leu Asp Ile Tyr Val Glu Ile Gly Met Gly Gln Met Glu Arg Tyr Glu Glu Asp Phe Glu Ser Phe Met Leu Gln Asp
AtCUL1

ACTTCTTCGTATTATTCTCGCAAGGCATCAAGCTGGATTCAGGAAGATTCTTGCCCTGATTACATGTTGAAGTCTGAAGAATGTCTAAAG 2340
TGAAGAAGCATAATAAGAGCGTTCCGTAGTTCGACCTAAGTCCTTCTAAGAACGGGACTAATGTACAACTTCAGACTTCTTACAGATTTC
200 205 210 215 220 225
Thr Ser Ser Tyr Tyr Ser Arg Lys Ala Ser Ser Trp Ile Gln Glu Asp Ser Cys Pro Asp Tyr Met Leu Lys Ser Glu Glu Cys Leu Lys
AtCUL1

AAGGAGAGGGAGAGAGTGGCTCACTACCTACACTCAAGCAGTGAGCCAAAGCTGGTTGAGAAAGTACAACATGAATTGCTGGTTGTGTTT 2430
TTCCTCTCCCTCTCTCACCGAGTGATGGATGTGAGTTCGTCACTCGGTTTCGACCAACTCTTTCATGTTGTACTTAACGACCAACACAAA
230 235 240 245 250 255
Lys Glu Arg Glu Arg Val Ala His Tyr Leu His Ser Ser Ser Glu Pro Lys Leu Val Glu Lys Val Gln His Glu Leu Leu Val Val Phe
AtCUL1

GCAAGTCAGCTTCTAGAAAAAGAACACTCAGGGTGCCGTGCATTGCTAAGAGATGACAAGGTGGATGATCTCTCCAGGATGTACAGGCTT 2520
CGTTCAGTCGAAGATCTTTTTCTTGTGAGTCCCACGGCACGTAACGATTCTCTACTGTTCCACCTACTAGAGAGGTCCTACATGTCCGAA
260 265 270 275 280 285
Ala Ser Gln Leu Leu Glu Lys Glu His Ser Gly Cys Arg Ala Leu Leu Arg Asp Asp Lys Val Asp Asp Leu Ser Arg Met Tyr Arg Leu
AtCUL1

TACCATAAAATTTTGCGAGGCTTGGAACCTGTTGCAAACATCTTTAAGCAGCATGTCACAGCAGAGGGTAACGCTCTTGTCCAACAGGCC 2610
ATGGTATTTTAAAACGCTCCGAACCTTGGACAACGTTTGTAGAAATTCGTCGTACAGTGTCGTCTCCCATTGCGAGAACAGGTTGTCCGG
290 295 300 305 310 315
Tyr His Lys Ile Leu Arg Gly Leu Glu Pro Val Ala Asn Ile Phe Lys Gln His Val Thr Ala Glu Gly Asn Ala Leu Val Gln Gln Ala
AtCUL1

GAAGACACGGCTACTAATCAGGTTGCAAATACTGCTAGCGTCCAGGAACAGGTTCTTATCAGAAAAGTGATTGAACTTCATGATAAATAC 2700
CTTCTGTGCCGATGATTAGTCCAACGTTTATGACGATCGCAGGTCCTTGTCCAAGAATAGTCTTTTCACTAACTTGAAGTACTATTTATG
320 325 330 335 340 345
Glu Asp Thr Ala Thr Asn Gln Val Ala Asn Thr Ala Ser Val Gln Glu Gln Val Leu Ile Arg Lys Val Ile Glu Leu His Asp Lys Tyr
AtCUL1

ATGGTATATGTCACCGAGTGTTTCCAGAACCACACCCTCTTCCATAAGGCTTTGAAAGAGGCATTTGAGATTTTTTGTAACAAAACGGTT 2790
TACCATATACAGTGGCTCACAAAGGTCTTGGTGTGGGAGAAGGTATTCCGAAACTTTCTCCGTAAACTCTAAAAAACATTGTTTTGCCAA
350 355 360 365 370 375
Met Val Tyr Val Thr Glu Cys Phe Gln Asn His Thr Leu Phe His Lys Ala Leu Lys Glu Ala Phe Glu Ile Phe Cys Asn Lys Thr Val
AtCUL1

GCTGGAAGTTCAAGTGCAGAACTACTTGCAACATTTTGCGACAATATTCTCAAAAAGGGGGGAAGTGAAAAGCTGAGTGATGAAGCTATC 2880
CGACCTTCAAGTTCACGTCTTGATGAACGTTGTAAAACGCTGTTATAAGAGTTTTTCCCCCCTTCACTTTTCGACTCACTACTTCGATAG
380 385 390 395 400 405
Ala Gly Ser Ser Ser Ala Glu Leu Leu Ala Thr Phe Cys Asp Asn Ile Leu Lys Lys Gly Gly Ser Glu Lys Leu Ser Asp Glu Ala Ile
AtCUL1

GAAGATACGCTTGAGAAGGTTGTCAAATTGCTTGCATACATAAGTGACAAGGATCTTTTCGCTGAGTTCTACAGGAAGAAGCTGGCCCGT 2970
CTTCTATGCGAACTCTTCCAACAGTTTAACGAACGTATGTATTCACTGTTCCTAGAAAAGCGACTCAAGATGTCCTTCTTCGACCGGGCA
410 415 420 425 430 435
Glu Asp Thr Leu Glu Lys Val Val Lys Leu Leu Ala Tyr Ile Ser Asp Lys Asp Leu Phe Ala Glu Phe Tyr Arg Lys Lys Leu Ala Arg
AtCUL1

AGGCTCTTATTTGATCGCAGTGCTAATGATGATCATGAGAGAAGTATCCTGACAAAGCTCAAGCAACAATGTGGTGGACAGTTTACTTCT 3060
TCCGAGAATAAACTAGCGTCACGATTACTACTAGTACTCTCTTCATAGGACTGTTTCGAGTTCGTTGTTACACCACCTGTCAAATGAAGA
440 445 450 455 460 465
Arg Leu Leu Phe Asp Arg Ser Ala Asn Asp Asp His Glu Arg Ser Ile Leu Thr Lys Leu Lys Gln Gln Cys Gly Gly Gln Phe Thr Ser
AtCUL1

AAGATGGAGGGCATGGTGACGGATTTGACACTGGCAAGAGAAAACCAAAACAGTTTCGAGGATTATCTAGGCAGTAACCCTGCTGCAAAC 3150
TTCTACCTCCCGTACCACTGCCTAAACTGTGACCGTTCTCTTTTGGTTTTGTCAAAGCTCCTAATAGATCCGTCATTGGGACGACGTTTG
470 475 480 485 490 495
Lys Met Glu Gly Met Val Thr Asp Leu Thr Leu Ala Arg Glu Asn Gln Asn Ser Phe Glu Asp Tyr Leu Gly Ser Asn Pro Ala Ala Asn
AtCUL1

CCAGGGATTGACTTGACCGTCACTGTTCTTACCACTGGTTTCTGGCCAAGTTACAAATCATTTGACATAAATCTACCCAGTGAAATGATC 3240
GGTCCCTAACTGAACTGGCAGTGACAAGAATGGTGACCAAAGACCGGTTCAATGTTTAGTAAACTGTATTTAGATGGGTCACTTTACTAG
500 505 510 515 520 525
Pro Gly Ile Asp Leu Thr Val Thr Val Leu Thr Thr Gly Phe Trp Pro Ser Tyr Lys Ser Phe Asp Ile Asn Leu Pro Ser Glu Met Ile
AtCUL1

AAGTGTGTTGAAGTCTTCAAAGGGTTTTATGAAACGAAAACGAAACACAGGAAGCTTACGTGGATCTATTCACTGGGAACTTGTCACATA 3330
TTCACACAACTTCAGAAGTTTCCCAAAATACTTTGCTTTTGCTTTGTGTCCTTCGAATGCACCTAGATAAGTGACCCTTGAACAGTGTAT
530 535 540 545 550 555
Lys Cys Val Glu Val Phe Lys Gly Phe Tyr Glu Thr Lys Thr Lys His Arg Lys Leu Thr Trp Ile Tyr Ser Leu Gly Thr Cys His Ile
AtCUL1

AACGGGAAGTTTGATCAAAAGGCCATCGAGTTAATAGTGTCTACTTACCAGGCTGCTGTGCTTCTACTCTTTAACACAACTGACAAGTTA 3420
TTGCCCTTCAAACTAGTTTTCCGGTAGCTCAATTATCACAGATGAATGGTCCGACGACACGAAGATGAGAAATTGTGTTGACTGTTCAAT
560 565 570 575 580 585
Asn Gly Lys Phe Asp Gln Lys Ala Ile Glu Leu Ile Val Ser Thr Tyr Gln Ala Ala Val Leu Leu Leu Phe Asn Thr Thr Asp Lys Leu
AtCUL1

AGTTACACTGAGATCTTGGCTCAACTGAACCTAAGCCATGAAGATCTAGTTAGGTTGCTTCATTCCTTGTCATGTGCTAAGTACAAGATA 3510
TCAATGTGACTCTAGAACCGAGTTGACTTGGATTCGGTACTTCTAGATCAATCCAACGAAGTAAGGAACAGTACACGATTCATGTTCTAT
590 595 600 605 610 615
Ser Tyr Thr Glu Ile Leu Ala Gln Leu Asn Leu Ser His Glu Asp Leu Val Arg Leu Leu His Ser Leu Ser Cys Ala Lys Tyr Lys Ile
AtCUL1

CTCCTTAAGGAGCCAAACACCAAGACTGTCTCCCAGAATGATGCCTTTGAGTTCAACTCCAAATTCACCGATAGAATGCGCAGAATCAAG 3600
GAGGAATTCCTCGGTTTGTGGTTCTGACAGAGGGTCTTACTACGGAAACTCAAGTTGAGGTTTAAGTGGCTATCTTACGCGTCTTAGTTC
620 625 630 635 640 645
Leu Leu Lys Glu Pro Asn Thr Lys Thr Val Ser Gln Asn Asp Ala Phe Glu Phe Asn Ser Lys Phe Thr Asp Arg Met Arg Arg Ile Lys
AtCUL1

ATCCCTCTTCCCCCAGTTGATGAAAGGAAGAAAGTCGTTGAAGATGTCGATAAAGACAGAAGATATGCAATTGATGCTGCCATTGTCAGG 3690
TAGGGAGAAGGGGGTCAACTACTTTCCTTCTTTCAGCAACTTCTACAGCTATTTCTGTCTTCTATACGTTAACTACGACGGTAACAGTCC
650 655 660 665 670 675
Ile Pro Leu Pro Pro Val Asp Glu Arg Lys Lys Val Val Glu Asp Val Asp Lys Asp Arg Arg Tyr Ala Ile Asp Ala Ala Ile Val Arg
AtCUL1

ATCATGAAGAGCAGGAAAGTATTGGGACATCAACAACTTGTTTCTGAGTGTGTTGAGCAACTTAGCCGAATGTTCAAGCCTGATATCAAA 3780
TAGTACTTCTCGTCCTTTCATAACCCTGTAGTTGTTGAACAAAGACTCACACAACTCGTTGAATCGGCTTACAAGTTCGGACTATAGTTT
680 685 690 695 700 705
Ile Met Lys Ser Arg Lys Val Leu Gly His Gln Gln Leu Val Ser Glu Cys Val Glu Gln Leu Ser Arg Met Phe Lys Pro Asp Ile Lys
AtCUL1

GCGATCAAGAAGCGTATGGAGGATTTAATAACCAGAGATTATTTGGAGAGGGACAAGGAGAATCCTAACATGTTTAGGTACTTGGCTggt 3870
CGCTAGTTCTTCGCATACCTCCTAAATTATTGGTCTCTAATAAACCTCTCCCTGTTCCTCTTAGGATTGTACAAATCCATGAACCGAcca
710 715 720 725 730 735
Ala Ile Lys Lys Arg Met Glu Asp Leu Ile Thr Arg Asp Tyr Leu Glu Arg Asp Lys Glu Asn Pro Asn Met Phe Arg Tyr Leu Ala Gly
AtCUL1

accctcgagtctggtaaagaaaccgctgctgcgaaatttgaacgccagcacatggactcgtctactagcgcagcttaattaacctaggct 3960
tgggagctcagaccatttctttggcgacgacgctttaaacttgcggtcgtgtacctgagcagatgatcgcgtcgaattaattggatccga
1 5 10 15
Thr Leu Glu Ser Gly Lys Glu Thr Ala Ala Ala Lys Phe Glu Arg Gln His Met Asp Ser Ser Thr Ser Ala Ala *
S
(in frame with S)

gctgccaccgctgagcaataactagcataaccccttggggcctctaaacgggtcttgaggggttttttgctgaaaggaggaactatatcc 4050
cgacggtggcgactcgttattgatcgtattggggaaccccggagatttgcccagaactccccaaaaaacgactttcctccttgatatagg
T7 terminator

ggattggcgaatgggacgcgccctgtagcggcgcattaagcgcggcgggtgtggtggttacgcgcagcgtgaccgctacacttgccagcg 4140
cctaaccgcttaccctgcgcgggacatcgccgcgtaattcgcgccgcccacaccaccaatgcgcgtcgcactggcgatgtgaacggtcgc
f1 ori

ccctagcgcccgctcctttcgctttcttcccttcctttctcgccacgttcgccggctttccccgtcaagctctaaatcgggggctccctt 4230
gggatcgcgggcgaggaaagcgaaagaagggaaggaaagagcggtgcaagcggccgaaaggggcagttcgagatttagcccccgagggaa
f1 ori

tagggttccgatttagtgctttacggcacctcgaccccaaaaaacttgattagggtgatggttcacgtagtgggccatcgccctgataga 4320
atcccaaggctaaatcacgaaatgccgtggagctggggttttttgaactaatcccactaccaagtgcatcacccggtagcgggactatct
f1 ori

cggtttttcgccctttgacgttggagtccacgttctttaatagtggactcttgttccaaactggaacaacactcaaccctatctcggtct 4410
gccaaaaagcgggaaactgcaacctcaggtgcaagaaattatcacctgagaacaaggtttgaccttgttgtgagttgggatagagccaga
f1 ori

attcttttgatttataagggattttgccgatttcggcctattggttaaaaaatgagctgatttaacaaaaatttaacgcgaattttaaca 4500
taagaaaactaaatattccctaaaacggctaaagccggataaccaatttttttactcgactaaattgtttttaaattgcgcttaaaattgt
f1 ori

aaatattaacgtttacaatttctggcggcacgatggcatgagattatcaaaaaggatcttcacctagatccttttaaattaaaaatgaag 4590
tttataattgcaaatgttaaagaccgccgtgctaccgtactctaatagtttttcctagaagtggatctaggaaaatttaatttttacttc
f1 ori

ttttaaatcaatctaaagtatatatgagtaaacttggtctgacagttaccaatgcttaatcagtgaggcacctatctcagcgatctgtct 4680
aaaatttagttagatttcatatatactcatttgaaccagactgtcaatggttacgaattagtcactccgtggatagagtcgctagacaga
285 280 275
* Trp His Lys Ile Leu Ser Ala Gly Ile Glu Ala Ile Gln Arg
AmpR

atttcgttcatccatagttgcctgactccccgtcgtgtagataactacgatacgggagggcttaccatctggccccagtgctgcaatgat 4770
taaagcaagtaggtatcaacggactgaggggcagcacatctattgatgctatgccctcccgaatggtagaccggggtcacgacgttacta
270 265 260 255 250 245
Asn Arg Glu Asp Met Thr Ala Gln Ser Gly Thr Thr Tyr Ile Val Val Ile Arg Ser Pro Lys Gly Asp Pro Gly Leu Ala Ala Ile Ile
AmpR

accgcgagacccacgctcaccggctccagatttatcagcaataaaccagccagccggaagggccgagcgcagaagtggtcctgcaacttt 4860
tggcgctctgggtgcgagtggccgaggtctaaatagtcgttatttggtcggtcggccttcccggctcgcgtcttcaccaggacgttgaaa
240 235 230 225 220 215
Gly Arg Ser Gly Arg Glu Gly Ala Gly Ser Lys Asp Ala Ile Phe Trp Gly Ala Pro Leu Ala Ser Arg Leu Leu Pro Gly Ala Val Lys
AmpR

atccgcctccatccagtctattaattgttgccgggaagctagagtaagtagttcgccagttaatagtttgcgcaacgttgttgccattgc 4950
taggcggaggtaggtcagataattaacaacggcccttcgatctcattcatcaagcggtcaattatcaaacgcgttgcaacaacggtaacg
210 205 200 195 190 185
Asp Ala Glu Met Trp Asp Ile Leu Gln Gln Arg Ser Ala Leu Thr Leu Leu Glu Gly Thr Leu Leu Lys Arg Leu Thr Thr Ala Met Ala
AmpR

tacaggcatcgtggtgtcacgctcgtcgtttggtatggcttcattcagctccggttcccaacgatcaaggcgagttacatgatcccccat 5040
atgtccgtagcaccacagtgcgagcagcaaaccataccgaagtaagtcgaggccaagggttgctagttccgctcaatgtactagggggta

180 175 170 165 160 155
Val Pro Met Thr Thr Asp Arg Glu Asp Asn Pro Ile Ala Glu Asn Leu Glu Pro Glu Trp Arg Asp Leu Arg Thr Val His Asp Gly Met
AmpR

gttgtgcaaaaaagcggttagctccttcggtcctccgatcgttgtcagaagtaagttggccgcagtgttatcactcatggttatggcagc 5130
caacacgttttttcgccaatcgaggaagccaggaggctagcaacagtcttcattcaaccggcgtcacaatagtgagtaccaataccgtcg

150 145 140 135 130 125
Asn His Leu Phe Ala Thr Leu Glu Lys Pro Gly Gly Ile Thr Thr Leu Leu Leu Asn Ala Ala Thr Asn Asp Ser Met Thr Ile Ala Ala
AmpR

actgcataattctcttactgtcatgccatccgtaagatgcttttctgtgactggtgagtactcaaccaagtcattctgagaatagtgtat 5220
tgacgtattaagagaatgacagtacggtaggcattctacgaaaagacactgaccactcatgagttggttcagtaagactcttatcacata

120 115 110 105 100 95
Ser Cys Leu Glu Arg Val Thr Met Gly Asp Thr Leu His Lys Glu Thr Val Pro Ser Tyr Glu Val Leu Asp Asn Gln Ser Tyr His Ile
AmpR

gcggcgaccgagttgctcttgcccggcgtcaatacgggataataccgcgccacatagcagaactttaaaagtgctcatcattggaaaacg 5310
cgccgctggctcaacgagaacgggccgcagttatgccctattatggcgcggtgtatcgtcttgaaattttcacgagtagtaacctttgc

90 85 80 75 70 65
Arg Arg Gly Leu Gln Glu Gln Gly Ala Asp Ile Arg Ser Leu Val Ala Gly Cys Leu Leu Val Lys Phe Thr Ser Met Met Pro Phe Arg
AmpR

ttcttcggggcgaaaactctcaaggatcttaccgctgttgagatccagttcgatgtaacccactcgtgcacccaactgatcttcagcatc 5400
aagaagccccgcttttgagagttcctagaatggcgacaactctaggtcaagctacattgggtgagcacgtgggttgactagaagtcgtag

60 55 50 45 40 35
Glu Glu Pro Arg Phe Ser Glu Leu Ile Lys Gly Ser Asn Leu Asp Leu Glu Ile Tyr Gly Val Arg Ala Gly Leu Gln Asp Glu Ala Asp
AmpR

ttttactttcaccagcgtttctgggtgagcaaaaacaggaaggcaaaatgccgcaaaaaagggaataagggcgacacggaaatgttgaat 5490
aaaatgaaagtggtcgcaaagacccactcgtttttgtccttccgttttacggcgttttttcccttattcccgctgtgcctttacaactta

30 25 20 15 10 5
Lys Val Lys Val Leu Thr Glu Pro His Ala Phe Val Pro Leu Cys Phe Ala Ala Phe Phe Pro Ile Leu Ala Val Arg Phe His Gln Ile
signal sequence
AmpR

actcatactcttcctttttcaatcatgattgaagcatttatcagggttattgtctcatgagcggatacatatttgaatgtatttagaaaa 5580
tgagtatgagaaggaaaaagttagtactaacttcgtaaatagtcccaataacagagtactcgcctatgtataaacttacataaatctttt

1
Ser Met
signal...
AmpR
AmpR promoter

ataaacaaataggtcatgaccaaaatcccttaacgtgagttttcgttccactgagcgtcagaccccgtagaaaagatcaaaggatcttct 5670
tatttgtttatccagtactggttttagggaattgcactcaaaagcaaggtgactcgcagtctggggcatcttttctagtttcctagaaga

AmpR promoter
ori

tgagatcctttttttctgcgcgtaatctgctgcttgcaaacaaaaaaaccaccgctaccagcggtggtttgtttgccggatcaagagcta 5760
actctaggaaaaaaagacgcgcattagacgacgaacgtttgtttttttggtggcgatggtcgccaccaaacaaacggcctagttctcgat

ori

ccaactctttttccgaaggtaactggcttcagcagagcgcagataccaaatactgtccttctagtgtagccgtagttaggccaccacttc 5850
ggttgagaaaaaggcttccattgaccgaagtcgtctcgcgtctatggtttatgacaggaagatcacatcggcatcaatccggtggtgaag

ori

aagaactctgtagcaccgcctacatacctcgctctgctaatcctgttaccagtggctgctgccagtggcgataagtcgtgtcttaccggg 5940
ttcttgagacatcgtggcggatgtatggagcgagacgattaggacaatggtcaccgacgacggtcaccgctattcagcacagaatggccc

ori

ttggactcaagacgatagttaccggataaggcgcagcggtcgggctgaacggggggttcgtgcacacagcccagcttggagcgaacgacc 6030
aacctgagttctgctatcaatggcctattccgcgtcgccagcccgacttgccccccaagcacgtgtgtcgggtcgaacctcgcttgctgg
ori

tacaccgaactgagatacctacagcgtgagctatgagaaagcgccacgcttcccgaagggagaaaggcggacaggtatccggtaagcggc 6120
atgtggcttgactctatggatgtcgcactcgatactctttcgcggtgcgaagggcttccctctttccgcctgtccataggccattcgccg
ori

agggtcggaacaggagagcgcacgagggagcttccagggggaaacgcctggtatctttatagtcctgtcgggtttcgccacctctgactt 6210
tcccagccttgtcctctcgcgtgctccctcgaaggtccccctttgcggaccatagaaatatcaggacagcccaaagcggtggagactgaa
ori

gagcgtcgatttttgtgatgctcgtcaggggggcggagcctatggaaaaacgccagcaacgcggcctttttacggttcctggccttttgc 6300
ctcgcagctaaaaacactacgagcagtccccccgcctcggatacctttttgcggtcgttgcgccggaaaaatgccaaggaccggaaaacg
ori

tggccttttgctcacatgttctttcctgcgttatcccctgattctgtggataaccgtattaccgcctttgagtgagctgataccgctcgc 6390
accggaaaacgagtgtacaagaaaggacgcaataggggactaagacacctattggcataatggcggaaactcactcgactatggcgagcg

cgcagccgaacgaccgagcgcagcgagtcagtgagcgaggaagcggaagagcgcctgatgcggtattttctccttacgcatctgtgcggt 6480
gcgtcggcttgctggctcgcgtcgctcagtcactcgctccttcgccttctcgcggactacgccataaaagaggaatgcgtagacacgcca
bom

atttcacaccgcatatatggtgcactctcagtacaatctgctctgatgccgcatagttaagccagtatacactccgctatcgctacgtga 6570
taaagtgtggcgtatataccacgtgagagtcatgttagacgagactacggcgtatcaattcggtcatatgtgaggcgatagcgatgcact
bom

ctgggtcatggctgcgccccgacacccgccaacacccgctgacgcgccctgacgggcttgtctgctcccggcatccgcttacagacaagc 6660
gacccagtaccgacgcggggctgtgggcggttgtgggcgactgcgcgggactgcccgaacagacgagggccgtaggcgaatgtctgttcg
bom

tgtgaccgtctccgggagctgcatgtgtcagaggttttcaccgtcatcaccgaaacgcgcgaggcagctgcggtaaagctcatcagcgtg 6750
acactggcagaggccctcgacgtacacagtctccaaaagtggcagtagtggctttgcgcgctccgtcgacgccatttcgagtagtcgcac
60 55 50 45
* Leu Asn Glu Gly Asp Asp Gly Phe Arg Ala Leu Cys Ser Arg Tyr Leu Glu Asp Ala His
rop

gtcgtgaagcgattcacagatgtctgcctgttcatccgcgtccagctcgttgagtttctccagaagcgttaatgtctggcttctgataaa 6840
cagcacttcgctaagtgtctacagacggacaagtaggcgcaggtcgagcaactcaaagaggtcttcgcaattacagaccgaagactattt
40 35 30 25 20 15
Asp His Leu Ser Glu Cys Ile Asp Ala Gln Glu Asp Ala Asp Leu Glu Asn Leu Lys Glu Leu Leu Thr Leu Thr Gln Ser Arg Ile Phe
rop

gcgggccatgttaagggcggtttttcctgtttggtcactgatgcctccgtgtaagggggatttctgttcatgggggtaatgataccgat 6930
cgcccggtacaattcccgccaaaaaggacaaaccagtgactacggaggcacattccccctaaagacaagtacccccattactatggcta
10 5 1
Arg Ala Met Asn Leu Ala Thr Lys Glu Gln Lys Thr Met
rop

gaaacgagagaggatgctcacgatacgggttactgatgatgaacatgcccggttactggaacgttgtgagggtaaacaactggcggtatg 7020
ctttgctctctcctacgagtgctatgcccaatgactactacttgtacgggccaatgaccttgcaacactcccatttgttgaccgccatac

gatgcggcgggaccagagaaaaatcactcagggtcaatgccagcgcttcgttaatacagatgtaggtgttccacagggtagccagcagca 7110
ctacgccgccctggtctctttttagtgagtcccagttacggtcgcgaagcaattatgtctacatccacaaggtgtcccatcggtcgtcgt

tcctgcgatgcagatccggaacataatggtgcagggcgctgacttccgcgtttccagactttacgaaacacggaaaccgaagaccattca 7200
aggacgctacgtctaggccttgtattaccacgtcccgcgactgaaggcgcaaaggtctgaaatgctttgtgcctttggcttctggtaagt

```
tgttgttgctcaggtcgcagacgttttgcagcagcagtcgcttcacgttcgctcgcgtatcggtgattcattctgctaaccagtaaggca  7290
acaacaacgagtccagcgtctgcaaaacgtcgtcgtcagcgaagtgcaagcgagcgcatagccactaagtaagacgattggtcattccgt

accccgccagcctagccgggtcctcaacgacaggagcacgatcatgctagtcatgccccgcgcccaccggaaggagctgactgggttgaa  7380
tggggcggtcggatcggcccaggagttgctgtcctcgtgctagtacgatcagtacggggcgcgggtggccttcctcgactgacccaactt

ggctctcaagggcatcggtcgagatcccggtgcctaatgagtgagctaacttacattaattgcgttgcgctcactgcccgctttccagtc  7470
ccgagagttcccgtagccagctctagggccacggattactcactcgattgaatgtaattaacgcaacgcgagtgacgggcgaaaggtcag
```

360 ... 355: * Gln Gly Ser Glu Leu Arg

lacI

```
gggaaacctgtcgtgccagctgcattaatgaatcggccaacgcgcggggagaggcggtttgcgtattgggcgccagggtggtttttcttt  7560
ccctttggacagcacggtcgacgtaattacttagccggttgcgcgcccctctccgccaaacgcataacccgcggtcccaccaaaaagaaa
```

350 ... 345 ... 340 ... 335 ... 330 ... 325: Ser Val Gln Arg Ala Leu Gln Met Leu Ser Asp Ala Leu Ala Arg Pro Ser Ala Thr Gln Thr Asn Pro Ala Leu Thr Thr Lys Arg Lys

lacI

```
tcaccagtgagacgggcaacagctgattgcccttcaccgcctggccctgagagagttgcagcaagcggtccacgctggtttgccccagca  7650
agtggtcactctgcccgttgtcgactaacgggaagtggcggaccgggactctctcaacgtcgttcgccaggtgcgaccaaacggggtcgt
```

320 ... 315 ... 310 ... 305 ... 300 ... 295: Val Leu Ser Val Pro Leu Leu Gln Asn Gly Lys Val Ala Gln Gly Gln Ser Leu Gln Leu Leu Arg Asp Val Ser Thr Gln Gly Leu Leu

lacI

```
ggcgaaaatcctgtttgatggtggttaacggcgggatataacatgagctgtcttcggtatcgtcgtatcccactaccgagatgtccgcac  7740
ccgcttttaggacaaactaccaccaattgccgccctatattgtactcgacagaagccatagcagcatagggtgatggctctacaggcgtg
```

290 ... 285 ... 280 ... 275 ... 270 ... 265: Arg Phe Asp Gln Lys Ile Thr Thr Leu Pro Pro Ile Tyr Cys Ser Ser Asp Glu Thr Asp Asp Tyr Gly Val Val Ser Ile Asp Ala Gly

lacI

```
caacgcgcagcccggactcggtaatggcgcgcattgcgcccagcgccatctgatcgttggcaaccagcatcgcagtgggaacgatgccct  7830
gttgcgcgtcgggcctgagccattaccgcgcgtaacgcgggtcgcggtagactagcaaccgttggtcgtagcgtcacccttgctacggga
```

260 ... 255 ... 250 ... 245 ... 240 ... 235: Val Arg Leu Gly Ser Glu Thr Ile Ala Arg Met Ala Gly Leu Ala Met Gln Asp Asn Ala Val Leu Met Ala Thr Pro Val Ile Gly Glu

lacI

```
cattcagcatttgcatggtttgttgaaaaccggacatggcactccagtcgccttcccgttccgctatcggctgaatttgattgcgagtga  7920
gtaagtcgtaaacgtaccaaacaacttttggcctgtaccgtgaggtcagcggaagggcaaggcgatagccgacttaaactaacgctcact
```

230 ... 225 ... 220 ... 215 ... 210 ... 205: Asn Leu Met Gln Met Thr Gln Gln Phe Gly Ser Met Ala Ser Trp Asp Gly Glu Arg Glu Ala Ile Pro Gln Ile Gln Asn Arg Thr Leu

lacI

```
gatatttatgccagccagccagacgcagacgcgccgagacagaacttaatgggcccgctaacagcgcgatttgctggtgacccaatgcga  8010
ctataaatacggtcggtcggtctgcgtctgcgcggctctgtcttgaattacccgggcgattgtcgcgctaaacgaccactgggttacgct
```

200 ... 195 ... 190 ... 185 ... 180 ... 175: Tyr Lys His Trp Gly Ala Leu Arg Leu Arg Ala Ser Val Ser Ser Leu Pro Gly Ala Leu Leu Ala Ile Gln Gln His Gly Leu Ala Val

lacI

```
ccagatgctccacgcccagtcgcgtaccgtcttcatgggagaaaataatactgttgatgggtgtctggtcagagacatcaagaaataacg  8100
ggtctacgaggtgcgggtcagcgcatggcagaagtaccctcttttattatgacaactacccacagaccagtctctgtagttctttattgc
```

170 ... 165 ... 160 ... 155 ... 150 ... 145: Leu His Glu Val Gly Leu Arg Thr Gly Asp Glu His Ser Phe Ile Ile Ser Asn Ile Pro Thr Gln Asp Ser Val Asp Leu Phe Leu Ala

lacI

```
ccggaacattagtgcaggcagcttccacagcaatggcatcctggtcatccagcggatagttaatgatcagcccactgacgcgttgcgcga  8190
ggccttgtaatcacgtccgtcgaaggtgtcgttaccgtaggaccagtaggtcgcctatcaattactagtcgggtgactgcgcaacgcgct
```

140 ... 135 ... 130 ... 125 ... 120 ... 115: Pro Val Asn Thr Cys Ala Ala Glu Val Ala Ile Ala Asp Gln Asp Asp Leu Pro Tyr Asn Ile Ile Leu Gly Ser Val Arg Gln Ala Leu

lacI

```
gaagattgtgcaccgccgctttacaggcttcgacgccgcttcgttctaccatcgacaccaccacgctggcacccagttgatcggcgcgag   8280
cttctaacacgtggcggcgaaatgtccgaagctgcggcgaagcaagatggtagctgtggtggtgcgaccgtgggtcaactagccgcgctc
            110        105        100        95         90         85
Leu Asn His Val Ala Ala Lys Cys Ala Glu Val Gly Ser Arg Glu Val Met Ser Val Val Val Ser Ala Gly Leu Gln Asp Ala Arg Ser
< lacI

atttaatcgccgcgacaatttgcgacggcgcgtgcagggccagactggaggtggcaacgccaatcagcaacgactgtttgcccgccagtt   8370
taaattagcggcgctgttaaacgctgccgcgcacgtcccggtctgacctccaccgttgcggttagtcgttgctgacaaacgggcggtcaa
            80         75         70         65         60         55
Lys Ile Ala Ala Val Ile Gln Ser Pro Ala His Leu Ala Leu Ser Ser Thr Ala Val Gly Ile Leu Leu Ser Gln Lys Gly Ala Leu Gln
< lacI

gttgtgccacgcggttgggaatgtaattcagctccgccatcgccgcttccactttttcccgcgttttcgcagaaacgtggctggcctggt   8460
caacacggtgcgccaacccttacattaagtcgaggcggtagcggcgaaggtgaaaaagggcgcaaaagcgtctttgcaccgaccggacca
            50         45         40         35         30         25
Gln Ala Val Arg Asn Pro Ile Tyr Asn Leu Glu Ala Met Ala Ala Glu Val Lys Glu Arg Thr Lys Ala Ser Val His Ser Ala Gln Asn
< lacI

tcaccacgcgggaaacggtctgataagagacaccggcatactctgcgacatcgtataacgttactggtttcacattcaccaccctgaatt   8550
agtggtgcgccctttgccagactattctctgtggccgtatgagacgctgtagcatattgcaatgaccaaagtgtaagtggtgggacttaa
            20         15         10         5          1
Val Val Arg Ser Val Thr Gln Tyr Ser Val Gly Ala Tyr Glu Ala Val Asp Tyr Leu Thr Val Pro Lys Met
< lacI                                                     < lacI promoter

gactctcttccgggcgctatcatgccataccgcgaaaggttttgcgccattcgatggtgtc   ••• 3′   8611
ctgagagaaggcccgcgatagtacggtatggcgctttccaaaacgcggtaagctaccacag       5′
< lacI promoter
```

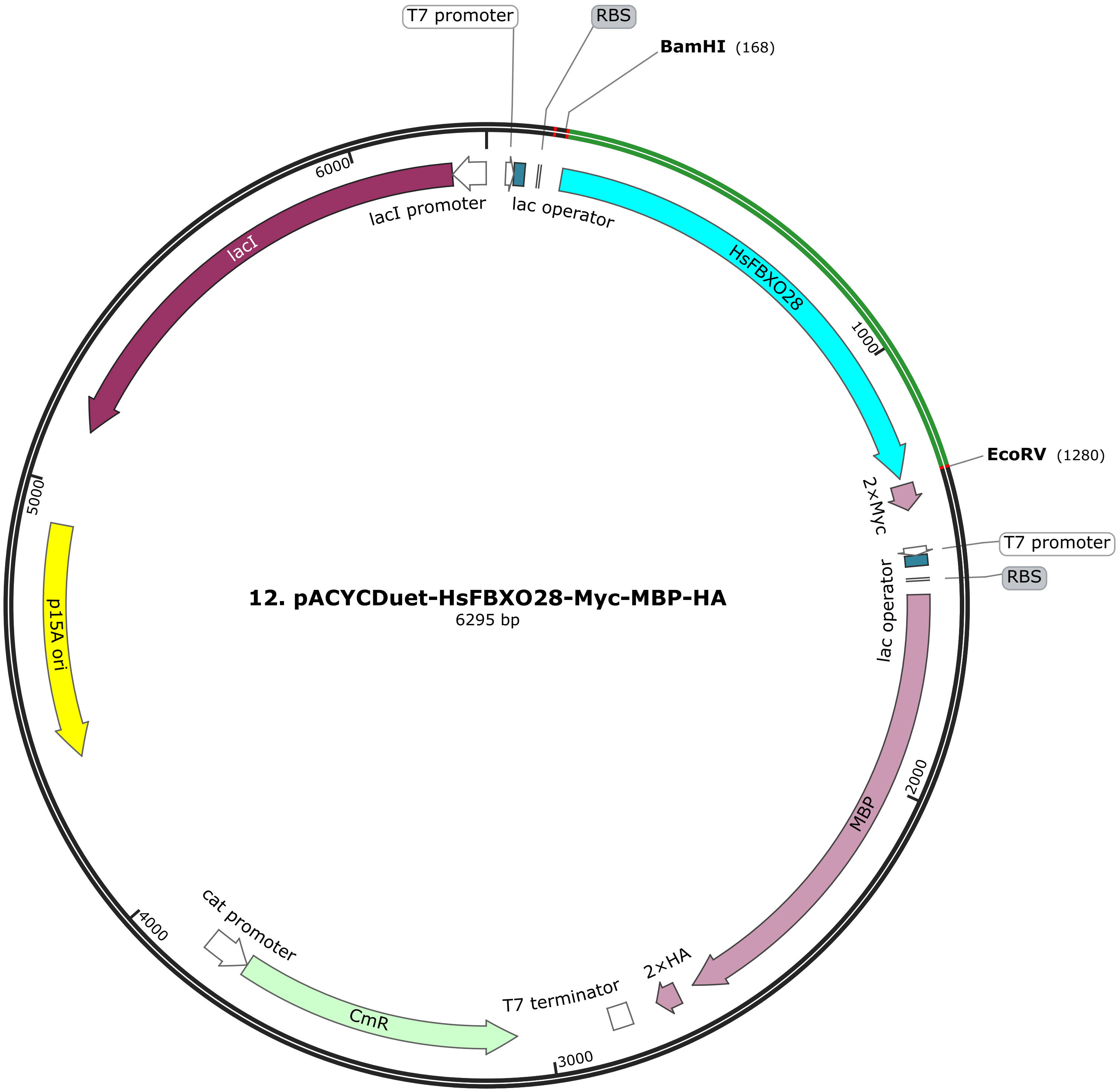
Created with SnapGene®
T7 promoter
RBS
BamHI (168)
6000
lacI promoter
lac operator
lacI
HsFBXO28
1000
EcoRV (1280)
2×Myc
T7 promoter
RBS
lac operator
5000
p15A ori
12. pACYCDuet-HsFBXO28-Myc-MBP-HA
6295 bp
MBP
2000
4000
cat promoter
2×HA
T7 terminator
CmR
3000

Sequence: 12. pACYCDuet-HsFBXO28-Myc-MBP-HA.dna (Circular / 6295 bp)
Enzymes: < Unsaved Enzyme Set > (2 of 678 total)
Features: 18 total

Unique Cutters **Bold**

5′ gggatctcgacgctctcccttatgcgactcctgcattaggaaattaatacgactcactataggggaattgtgagcggataacaattcccc 90
3′ ccctagagctgcgagagggaatacgctgaggacgtaatcctttaattatgctgagtgatatccccttaacactcgcctattgttaagggg
T7 promoter | lac operator

BamHI
tgtagaaataattttgtttaactttaataaggagatataccatgggcagcagcGcatcaccatcatcaccaagccaggGatccatggcgg 180
acatctttattaaaacaaattgaaattattcctctatatggtacccgtcgtcgCgtagtggtagtagtggttcggtccCtaggtaccgcc
RBS | HsFBXO28

cagcggcggaggagcggatggcagaggaaggaggcggcggccaaggcgacggcggttcctctttggcctccggctctacccagcgacagc 270
gtcgccgcctcctcgcctaccgtctccttcctccgccgccggttccgctgccgccaaggagaaaccggaggccgagatgggtcgctgtcg
HsFBXO28

ctccaccgcccgcgccacagcacccgcagccggggtcccaggcgctcccagcccccgcgctggctccggaccagctgcctcaaaacaaca 360
gaggtggcgggcgcggtgtcgtgggcgtcggccccagggtccgcgagggtcgggggcgcgaccgaggcctggtcgacggagttttgttgt
HsFBXO28

cgcttgtggcgctgcccatcgtagccatcgagaacatcctcagctttatgtcctacgacgaaattagccagctccgcctggtttgtaaaa 450
gcgaacaccgcgacgggtagcatcggtagctcttgtaggagtcgaaatacaggatgctgctttaatcggtcgaggcggaccaaacatttt
HsFBXO28

gaatggacttggtctgccagagaatgttgaatcagggatttctgaaagtggagaggtaccataatctatgtcagaaacaagttaaagcac 540
cttacctgaaccagacggtctcttacaacttagtccctaaagactttcacctctccatggtattagatacagtctttgttcaatttcgtg
HsFBXO28

aactcccaaggagagagtcagaaaggagaaaccattcattagctcgtcatgcagacattcttgctgctgttgaaacaaggctgtcactat 630
ttgagggttcctctctcagtctttcctctttggtaagtaatcgagcagtacgtctgtaagaacgacgacaactttgttccgacagtgata
HsFBXO28

taaatatgactttcatgaaatatgtggattccaatctctgttgcttcatcccaggaaaggtgattgatgagatttatcgtgtgttgagat 720
atttatactgaaagtactttatacacctaaggttagagacaacgaagtagggtcctttccactaactactctaaatagcacacaactcta
HsFBXO28

atgtcaattctaccagagcccctcaacgagctcatgaagtacttcaagaattaagggatatatcctctatggcaatggagtactttgatg 810
tacagttaagatggtctcggggagttgctcgagtacttcatgaagttcttaattccctatataggagataccgttacctcatgaaactac
HsFBXO28

aaaagattgttccaattttaaagaggaaattaccaggatcagatgtttctggaagactcatgggctctcctccagttccaggaccgtctg 900
ttttctaacaaggttaaaatttctcctttaatggtcctagtctacaaagaccttctgagtacccgagaggaggtcaaggtcctggcagac
HsFBXO28

cagccctaacaacaatgcagctcttctccaagcaaaatccttcaagacaagaggttaccaaactccagcagcaggttaaaacaaatggtg 990
gtcgggattgttgttacgtcgagaagaggttcgttttaggaagttctgttctccaatggtttgaggtcgtcgtccaattttgtttaccac
HsFBXO28

ctggcgtgactgttctcaggcgtgaaatttctgagcttcgcaccaaagtgcaagaacagcaaaaacagcttcaagaccaggaccagaaac 1080
gaccgcactgacaagagtccgcactttaaagactcgaagcgtggtttcacgttcttgtcgtttttgtcgaagttctggtcctggtctttg
HsFBXO28

tgctagagcagacccagatcataggtgaacaaaatgcacggttggcagagctagaacgcaaactacgagaagtaatggaaagtgctgtag 1170
acgatctcgtctgggtctagtatccacttgttttacgtgccaaccgtctcgatcttgcgtttgatgctcttcattacctttcacgacatc
HsFBXO28

gaaattcctcagggtccgggcagaatgaggagtctcctcggaaacgaaaaaggccacggaagccatagactctcttaggaaatctaaac 1260
ctttaaggagtcccaggcccgtcttactcctcagaggagcctttgctttttccggtgccttcggtatctgagagaatcctttagatttg
HsFBXO28

EcoRV
gtcttcggaatagaaagGATATCACTAGTCCTGAGCAGAAGCTCATCTCCGAGGAGGACCTCGAGCAGAAGCTCATCTCCGAGGAGGACC 1350
cagaagccttatctttcCTATAGTGATCAGGACTCGTCTTCGAGTAGAGGCTCCTCCTGGAGCTCGTCTTCGAGTAGAGGCTCCTCCTGG
1 5 10 15
Glu Gln Lys Leu Ile Ser Glu Glu Asp Leu Glu Gln Lys Leu Ile Ser Glu Glu Asp
HsFBXO28
2×Myc

TCCTGActgcaggtcgacaagcttgcggccgcataatgcttaagtcgaacagaaagtaatcgtattgtacacggccgcataatcgaaatt 1440
AGGACTgacgtccagctgttcgaacgccggcgtattacgaattcagcttgtctttcattagcataacatgtgccggcgtattagctttaa
20
Leu Leu Thr Ala Gly Arg Gln Ala Cys Gly Arg Ile Met Leu Lys Ser Asn Arg Lys *
(in frame with 2×Myc)
2×Myc
T7 promoter

aatacgactcactataggggaattgtgagcggataacaattccccatcttagtatattagttaagtataagaaggagatatacatatggc 1530
ttatgctgagtgatatccccttaacactcgcctattgttaaggggtagaatcatataatcaattcatattcttcctctatatgtataccg
T7 promoter
lac operator
RBS

agatctcaattgggaattcatgaaaatcgaagaaggtaaactggtaatctggattaacggcgataaaggctataacggtctcgctgaagt 1620
tctagagttaacccttaagtactttagcttcttccatttgaccattagacctaattgccgctatttccgatattgccagagcgacttca
1 5 10 15 20
Met Lys Ile Glu Glu Gly Lys Leu Val Ile Trp Ile Asn Gly Asp Lys Gly Tyr Asn Gly Leu Ala Glu Val
MBP

cggtaagaaattcgagaaagataccggaattaaagtcaccgttgagcatccggataaactggaagagaaattcccacaggttgcggcaac 1710
gccattctttaagctctttctatggccttaatttcagtggcaactcgtaggcctatttgaccttctctttaagggtgtccaacgccgttg
25 30 35 40 45 50
Gly Lys Lys Phe Glu Lys Asp Thr Gly Ile Lys Val Thr Val Glu His Pro Asp Lys Leu Glu Glu Lys Phe Pro Gln Val Ala Ala Thr
MBP

tggcgatggccctgacattatcttctgggcacacgaccgctttggtggctacgctcaatctggcctgttggctgaaatcaccccggacaa 1800
accgctaccgggactgtaatagaagacccgtgtgctggcgaaaccaccgatgcgagttagaccggacaaccgactttagtggggcctgtt
55 60 65 70 75 80
Gly Asp Gly Pro Asp Ile Ile Phe Trp Ala His Asp Arg Phe Gly Gly Tyr Ala Gln Ser Gly Leu Leu Ala Glu Ile Thr Pro Asp Lys
MBP

agcgttccaggacaagctgtatccgtttacctgggatgccgtacgttacaacggcaagctgattgcttacccgatcgctgttgaagcgtt 1890
tcgcaaggtcctgttcgacataggcaaatggaccctacggcatgcaatgttgccgttcgactaacgaatgggctagcgacaacttcgcaa
85 90 95 100 105 110
Ala Phe Gln Asp Lys Leu Tyr Pro Phe Thr Trp Asp Ala Val Arg Tyr Asn Gly Lys Leu Ile Ala Tyr Pro Ile Ala Val Glu Ala Leu
MBP

atcgctgatttataacaaagatctgctgccgaacccgccaaaaacctgggaagagatcccggcgctggataaagaactgaaagcgaaagg 1980
tagcgactaaatattgtttctagacgacggcttgggcggtttttggacccttctctagggccgcgacctatttcttgactttcgctttcc
115 120 125 130 135 140
Ser Leu Ile Tyr Asn Lys Asp Leu Leu Pro Asn Pro Pro Lys Thr Trp Glu Glu Ile Pro Ala Leu Asp Lys Glu Leu Lys Ala Lys Gly
MBP

taagagcgcgctgatgttcaacctgcaagaaccgtacttcacctggccgctgattgctgctgacggggggttatgcgttcaagtatgaaaa 2070
attctcgcgcgactacaagttggacgttcttggcatgaagtggaccggcgactaacgacgactgccccccaatacgcaagttcatactttt
145 150 155 160 165 170
Lys Ser Ala Leu Met Phe Asn Leu Gln Glu Pro Tyr Phe Thr Trp Pro Leu Ile Ala Ala Asp Gly Gly Tyr Ala Phe Lys Tyr Glu Asn
MBP

cggcaagtacgacattaaagacgtgggcgtggataacgctggcgcgaaagcgggtctgaccttcctggttgacctgattaaaaacaaaca 2160
gccgttcatgctgtaatttctgcacccgcacctattgcgaccgcgctttcgcccagactggaaggaccaactggactaatttttgtttgt
175 180 185 190 195 200
Gly Lys Tyr Asp Ile Lys Asp Val Gly Val Asp Asn Ala Gly Ala Lys Ala Gly Leu Thr Phe Leu Val Asp Leu Ile Lys Asn Lys His
MBP

catgaatgcagacaccgattactccatcgcagaagctgcctttaataaaggcgaaacagcgatgaccatcaacggcccgtgggcatggtc 2250
gtacttacgtctgtggctaatgaggtagcgtcttcgacggaaattatttccgctttgtcgctactggtagttgccgggcacccgtaccag
205 210 215 220 225 230
Met Asn Ala Asp Thr Asp Tyr Ser Ile Ala Glu Ala Ala Phe Asn Lys Gly Glu Thr Ala Met Thr Ile Asn Gly Pro Trp Ala Trp Ser
MBP

caacatcgacaccagcaaagtgaattatggtgtaacggtactgccgaccttcaagggtcaaccatccaaaccgttcgttggcgtgctgag 2340
gttgtagctgtggtcgtttcacttaataccacattgccatgacggctggaagttcccagttggtaggtttggcaagcaaccgcacgactc
235 240 245 250 255 260
Asn Ile Asp Thr Ser Lys Val Asn Tyr Gly Val Thr Val Leu Pro Thr Phe Lys Gly Gln Pro Ser Lys Pro Phe Val Gly Val Leu Ser
MBP

cgcaggtattaacgccgccagtccgaacaaagagctggcaaaagagttcctcgaaaactatctgctgactgatgaaggtctggaagcggt 2430
gcgtccataattgcggcggtcaggcttgtttctcgaccgttttctcaaggagcttttgatagacgactgactacttccagaccttcgcca
265 270 275 280 285 290
Ala Gly Ile Asn Ala Ala Ser Pro Asn Lys Glu Leu Ala Lys Glu Phe Leu Glu Asn Tyr Leu Leu Thr Asp Glu Gly Leu Glu Ala Val
MBP

taataaagacaaaccgctgggtgccgtagcgctgaagtcttacgaggaagagttggcgaaagatccacgtattgccgccactatggaaaa 2520
attatttctgtttggcgacccacggcatcgcgacttcagaatgctccttctcaaccgctttctaggtgcataacggcggtgatacctttt
295 300 305 310 315 320
Asn Lys Asp Lys Pro Leu Gly Ala Val Ala Leu Lys Ser Tyr Glu Glu Glu Leu Ala Lys Asp Pro Arg Ile Ala Ala Thr Met Glu Asn
MBP

cgcccagaaaggtgaaatcatgccgaacatcccgcagatgtccgctttctggtatgccgtgcgtactgcggtgatcaacgccgccagcgg 2610
gcgggtctttccactttagtacggcttgtagggcgtctacaggcgaaagaccatacggcacgcatgacgccactagttgcggcggtcgcc
325 330 335 340 345 350
Ala Gln Lys Gly Glu Ile Met Pro Asn Ile Pro Gln Met Ser Ala Phe Trp Tyr Ala Val Arg Thr Ala Val Ile Asn Ala Ala Ser Gly
MBP

tcgtcagactgtcgatgaagccctgaaagacgcgcagactgaattcgagCCCGGGAGGCCTctcggcgcgcctgcaggtTACCCATACGA 2700
agcagtctgacagcttcttcgggactttctgcgcgtctgacttaagctcGGGCCCTCCGGAgagccgcgcggacgtccaATGGGTATGCT
355 360 365 1
Arg Gln Thr Val Asp Glu Ala Leu Lys Asp Ala Gln Thr Glu Phe Glu Pro Gly Arg Pro Leu Gly Ala Pro Ala Gly Tyr Pro Tyr Asp
MBP (in frame with MBP) 2×HA

CGTACCAGATTACGCTTACCCATACGACGTACCAGATTACGCTcgacaagcttgcggccgcataatgcttaagtaattaacctaggctgc 2790
GCATGGTCTAATGCGAATGGGTATGCTGCATGGTCTAATGCGAgctgttcgaacgccggcgtattacgaattcattaattggatccgacg
5 10 15
Val Pro Asp Tyr Ala Tyr Pro Tyr Asp Val Pro Asp Tyr Ala Arg Gln Ala Cys Gly Arg Ile Met Leu Lys *
2×HA (in frame with 2×HA)

tgccaccgctgagcaataactagcataaccccttggggcctctaaacgggtcttgaggggttttttgctgaaacctcaggcatttgagaa 2880
acggtggcgactcgttattgatcgtattggggaaccccggagatttgcccagaactccccaaaaaacgactttggagtccgtaaactctt
T7 terminator

gcacacggtcacactgcttccggtagtcaataaaccggtaaaccagcaatagacataagcggctatttaacgaccctgccctgaaccgac 2970
cgtgtgccagtgtgacgaaggccatcagttatttggccatttggtcgttatctgtattcgccgataaattgctgggacgggacttggctg

gaccgggtcgaatttgctttcgaatttctgccattcatccgcttattatcacttattcaggcgtagcaccaggcgtttaagggcaccaat 3060
ctggcccagcttaaacgaaagcttaaagacggtaagtaggcgaataatagtgaataagtccgcatcgtggtccgcaaattcccgtggtta

aactgccttaaaaaaattacgccccgccctgccactcatcgcagtactgttgtaattcattaagcattctgccgacatggaagccatcac 3150
ttgacggaatttttttaatgcggggcgggacggtgagtagcgtcatgacaacattaagtaattcgtaagacggctgtaccttcggtagtg
220 215 210 205 200
* Ala Gly Gly Gln Trp Glu Asp Cys Tyr Gln Gln Leu Glu Asn Leu Met Arg Gly Val His Phe Gly Asp Cys
CmR

agacggcatgatgaacctgaatcgccagcggcatcagcaccttgtcgccttgcgtataatatttgcccatagtgaaaacgggggcgaaga 3240
tctgccgtactacttggacttagcggtcgccgtagtcgtggaacagcggaacgcatattataaacgggtatcacttttgcccccgcttct
195 190 185 180 175 170
Val Ala His His Val Gln Ile Ala Leu Pro Met Leu Val Lys Asp Gly Gln Thr Tyr Tyr Lys Gly Met Thr Phe Val Pro Ala Phe Phe
CmR

agttgtccatattggccacgtttaaatcaaaactggtgaaactcacccagggattggctgagacgaaaaacatattctcaataaaccctt 3330
tcaacaggtataaccggtgcaaatttagttttgaccactttgagtgggtccctaaccgactctgctttttgtataagagttatttgggaa
165 Asn Asp Met Asn Ala 160 Val Asn Leu Asp Phe 155 Ser Thr Phe Ser Val 150 Trp Pro Asn Ala Ser 145 Val Phe Phe Met Asn 140 Glu Ile Phe Gly Lys
CmR

tagggaaataggccaggttttcaccgtaacacgccacatcttgcgaatatatgtgtagaaactgccggaaatcgtcgtggtattcactcc 3420
atccctttatccggtccaaaagtggcattgtgcggtgtagaacgcttatatacacatctttgacggcctttagcagcaccataagtgagg
135 Pro Phe Tyr Ala Leu 130 Asn Glu Gly Tyr Cys 125 Ala Val Asp Gln Ser 120 Tyr Ile His Leu Phe 115 Gln Arg Phe Asp Asp 110 His Tyr Glu Ser Trp
CmR

agagcgatgaaaacgtttcagtttgctcatggaaaacggtgtaacaagggtgaacactatcccatatcaccagctcaccgtctttcattg 3510
tctcgctacttttgcaaagtcaaacgagtaccttttgccacattgttcccacttgtgatagggtatagtggtcgagtggcagaaagtaac
105 Leu Ser Ser Phe Thr 100 Glu Thr Gln Glu His 95 Phe Val Thr Tyr Cys 90 Pro His Val Ser Asp 85 Trp Ile Val Leu Glu 80 Gly Asp Lys Met Ala
CmR

ccatacggaactccggatgagcattcatcaggcgggcaagaatgtgaataaaggccggataaaacttgtgcttatttttctttacggtct 3600
ggtatgccttgaggcctactcgtaagtagtccgcccgttcttacacttatttccggcctattttgaacacgaataaaaagaaatgccaga
75 Met Arg Phe Glu Pro 70 His Ala Asn Met Leu 65 Arg Ala Leu Ile His 60 Ile Phe Ala Pro Tyr 55 Phe Lys His Lys Asn 50 Lys Lys Val Thr Lys
CmR

ttaaaaaggccgtaatatccagctgaacggtctggttataggtacattgagcaactgactgaaatgcctcaaaatgttctttacgatgcc 3690
aatttttccggcattataggtcgacttgccagaccaatatccatgtaactcgttgactgactttacggagttttacaagaaatgctacgg
45 Leu Phe Ala Thr Ile 40 Asp Leu Gln Val Thr 35 Gln Asn Tyr Thr Cys 30 Gln Ala Val Ser Gln 25 Phe Ala Glu Phe His 20 Glu Lys Arg His Trp
CmR

attgggatatatcaacggtggtatatccagtgatttttttctccattttagcttccttagctcctgaaaatctcgataactcaaaaaata 3780
taaccctatatagttgccaccatataggtcactaaaaaaagaggtaaaatcgaaggaatcgaggacttttagagctattgagtttttat
15 Gln Ser Ile Asp Val 10 Thr Thr Tyr Gly Thr 5 Ile Lys Lys Glu 1 Met
CmR
cat promoter

cgcccggtagtgatcttatttcattatggtgaaagttggaacctcttacgtgccgatcaacgtctcattttcgccaaaagttggcccagg 3870
gcgggccatcactagaataaagtaataccactttcaaccttggagaatgcacggctagttgcagagtaaaagcggttttcaaccgggtcc
cat promoter

gcttcccggtatcaacagggacaccaggatttatttattctgcgaagtgatcttccgtcacaggtatttattcggcgcaaagtgcgtcgg 3960
cgaagggccatagttgtccctgtggtcctaaataaataagacgcttcactagaaggcagtgtccataaataagccgcgtttcacgcagcc

gtgatgctgccaacttactgatttagtgtatgatggtgttttgaggtgctccagtggcttctgtttctatcagctgtccctcctgttca 4050
cactacgacggttgaatgactaaatcacatactaccacaaaactccacgaggtcaccgaagacaaagatagtcgacagggaggacaagt

gctactgacggggtggtgcgtaacggcaaaagcaccgccggacatcagcgctagcggagtgtatactggcttactatgttggcactgatg 4140
cgatgactgccccaccacgcattgccgttttcgtggcggcctgtagtcgcgatcgcctcacatatgaccgaatgatacaaccgtgactac

agggtgtcagtgaagtgcttcatgtggcaggagaaaaaaggctgcaccggtgcgtcagcagaatatgtgatacaggatatattccgcttc 4230
tcccacagtcacttcacgaagtacaccgtcctcttttttccgacgtggccacgcagtcgtcttatacactatgtcctatataaggcgaag

ctcgctcactgactcgctacgctcggtcgttcgactgcggcgagcggaaatggcttacgaacggggcggagatttcctggaagatgccag 4320
gagcgagtgactgagcgatgcgagccagcaagctgacgccgctcgcctttaccgaatgcttgccccgcctctaaaggaccttctacggtc

gaagatacttaacagggaagtgagagggccgcggcaaagccgtttttccataggctccgcccccctgacaagcatcacgaaatctgacgc 4410
cttctatgaattgtcccttcactctcccggcgccgtttcggcaaaaaggtatccgaggcggggggactgttcgtagtgctttagactgcg
p15A ori

tcaaatcagtggtggcgaaacccgacaggactataaagataccaggcgtttcccctggcggctccctcgtgcgctctcctgttcctgcct 4500
agtttagtcaccaccgctttgggctgtcctgatatttctatggtccgcaaaggggaccgccgagggagcacgcgagaggacaaggacgga
p15A ori

ttcggtttaccggtgtcattccgctgttatggccgcgtttgtctcattccacgcctgacactcagttccgggtaggcagttcgctccaag 4590
aagccaaatggccacagtaaggcgacaataccggcgcaaacagagtaaggtgcggactgtgagtcaaggcccatccgtcaagcgaggttc
p15A ori

ctggactgtatgcacgaaccccccgttcagtccgaccgctgcgccttatccggtaactatcgtcttgagtccaacccggaaagacatgca 4680
gacctgacatacgtgcttggggggcaagtcaggctggcgacgcggaataggccattgatagcagaactcaggttgggcctttctgtacgt
p15A ori

aaagcaccactggcagcagccactggtaattgatttagaggagttagtcttgaagtcatgcgccggttaaggctaaactgaaaggacaag 4770
tttcgtggtgaccgtcgtcggtgaccattaactaaatctcctcaatcagaacttcagtacgcggccaattccgatttgactttcctgttc
p15A ori

ttttggtgactgcgctcctccaagccagttacctcggttcaaagagttggtagctcagagaaccttcgaaaaaccgccctgcaaggcggt 4860
aaaaccactgacgcgaggaggttcggtcaatggagccaagtttctcaaccatcgagtctcttggaagctttttggcgggacgttccgcca
p15A ori

tttttcgttttcagagcaagagattacgcgcagaccaaaacgatctcaagaagatcatcttattaatcagataaaatatttctagatttc 4950
aaaaagcaaaagtctcgttctctaatgcgcgtctggttttgctagagttcttctagtagaataattagtctattttataaagatctaaag
p15A ori

agtgcaatttatctcttcaaatgtagcacctgaagtcagccccatacgatataagttgtaattctcatgttagtcatgccccgcgcccac 5040
tcacgttaaatagagaagtttacatcgtggacttcagtcggggtatgctatattcaacattaagagtacaatcagtacggggcgcgggtg

cggaaggagctgactgggttgaaggctctcaagggcatcggtcgagatcccggtgcctaatgagtgagctaacttacattaattgcgttg 5130
gccttcctcgactgacccaacttccgagagttcccgtagccagctctagggccacggattactcactcgattgaatgtaattaacgcaac

cgctcactgcccgctttccagtcgggaaacctgtcgtgccagctgcattaatgaatcggccaacgcgcggggagaggcggtttgcgtatt 5220
gcgagtgacgggcgaaaggtcagccctttggacagcacggtcgacgtaattacttagccggttgcgcgcccctctccgccaaacgcataa
360 355 350 345 340 335
* Gln Gly Ser Glu Leu Arg Ser Val Gln Arg Ala Leu Gln Met Leu Ser Asp Ala Leu Ala Arg Pro Ser Ala Thr Gln Thr Asn
lacI

gggcgccagggtggtttttcttttcaccagtgagacgggcaacagctgattgcccttcaccgcctggccctgagagagttgcagcaagcg 5310
cccgcggtcccaccaaaaagaaaagtggtcactctgcccgttgtcgactaacgggaagtggcggaccgggactctctcaacgtcgttcgc
330 325 320 315 310 305
Pro Ala Leu Thr Thr Lys Arg Lys Val Leu Ser Val Pro Leu Leu Gln Asn Gly Lys Val Ala Gln Gly Gln Ser Leu Gln Leu Leu Arg
lacI

gtccacgctggtttgccccagcaggcgaaaatcctgtttgatggtggttaacggcgggatataacatgagctgtcttcggtatcgtcgta 5400
caggtgcgaccaaacggggtcgtccgcttttaggacaaactaccaccaattgccgccctatattgtactcgacagaagccatagcagcat
300 295 290 285 280 275
Asp Val Ser Thr Gln Gly Leu Leu Arg Phe Asp Gln Lys Ile Thr Thr Leu Pro Pro Ile Tyr Cys Ser Ser Asp Glu Thr Asp Asp Tyr
lacI

tcccactaccgagatgtccgcaccaacgcgcagcccggactcggtaatggcgcgcattgcgcccagcgccatctgatcgttggcaaccag 5490
agggtgatggctctacaggcgtggttgcgcgtcgggcctgagccattaccgcgcgtaacgcgggtcgcggtagactagcaaccgttggtc
270 265 260 255 250 245
Gly Val Val Ser Ile Asp Ala Gly Val Arg Leu Gly Ser Glu Thr Ile Ala Arg Met Ala Gly Leu Ala Met Gln Asp Asn Ala Val Leu
lacI

catcgcagtgggaacgatgccctcattcagcatttgcatggtttgttgaaaaccggacatggcactccagtcgccttcccgttccgctat 5580
gtagcgtcacccttgctacgggagtaagtcgtaaacgtaccaaacaactttttggcctgtaccgtgaggtcagcggaagggcaaggcgata
240 235 230 225 220 215
Met Ala Thr Pro Val Ile Gly Glu Asn Leu Met Gln Met Thr Gln Gln Phe Gly Ser Met Ala Ser Trp Asp Gly Glu Arg Glu Ala Ile
lacI

cggctgaatttgattgcgagtgagatatttatgccagccagccagacgcagacgcgccgagacagaacttaatgggcccgctaacagcgc 5670
gccgacttaaactaacgctcactctataaatacggtcggtcggtctgcgtctgcgcggctctgtcttgaattacccgggcgattgtcgcg

210 205 200 195 190 185
Pro Gln Ile Gln Asn Arg Thr Leu Tyr Lys His Trp Gly Ala Leu Arg Leu Arg Ala Ser Val Ser Ser Leu Pro Gly Ala Leu Leu Ala
lacI

gatttgctggtgacccaatgcgaccagatgctccacgcccagtcgcgtaccgtcttcatgggagaaaataatactgttgatgggtgtctg 5760
ctaaacgaccactgggttacgctggtctacgaggtgcgggtcagcgcatggcagaagtaccctcttttattatgacaactacccacagac

180 175 170 165 160 155
Ile Gln Gln His Gly Leu Ala Val Leu His Glu Val Gly Leu Arg Thr Gly Asp Glu His Ser Phe Ile Ile Ser Asn Ile Pro Thr Gln
lacI

gtcagagacatcaagaaataacgccggaacattagtgcaggcagcttccacagcaatggcatcctggtcatccagcggatagttaatgat 5850
cagtctctgtagttctttattgcggccttgtaatcacgtccgtcgaaggtgtcgttaccgtaggaccagtaggtcgcctatcaattacta

150 145 140 135 130 125
Asp Ser Val Asp Leu Phe Leu Ala Pro Val Asn Thr Cys Ala Ala Glu Val Ala Ile Ala Asp Gln Asp Asp Leu Pro Tyr Asn Ile Ile
lacI

cagcccactgacgcgttgcgcgagaagattgtgcaccgccgctttacaggcttcgacgccgcttcgttctaccatcgacaccaccacgct 5940
gtcgggtgactgcgcaacgcgctcttctaacacgtggcggcgaaatgtccgaagctgcggcgaagcaagatggtagctgtggtggtgcga

120 115 110 105 100 95
Leu Gly Ser Val Arg Gln Ala Leu Leu Asn His Val Ala Ala Lys Cys Ala Glu Val Gly Ser Arg Glu Val Met Ser Val Val Val Ser
lacI

ggcacccagttgatcggcgcgagatttaatcgccgcgacaatttgcgacggcgcgtgcagggccagactggaggtggcaacgccaatcag 6030
ccgtgggtcaactagccgcgctctaaattagcggcgctgttaaacgctgccgcgcacgtcccggtctgacctccaccgttgcggttagtc

90 85 80 75 70 65
Ala Gly Leu Gln Asp Ala Arg Ser Lys Ile Ala Ala Val Ile Gln Ser Pro Ala His Leu Ala Leu Ser Ser Thr Ala Val Gly Ile Leu
lacI

caacgactgtttgcccgccagttgttgtgccacgcggttgggaatgtaattcagctccgccatcgccgcttccactttttcccgcgtttt 6120
gttgctgacaaacgggcggtcaacaacacggtgcgccaacccttacattaagtcgaggcggtagcggcgaaggtgaaaaagggcgcaaaa

60 55 50 45 40 35
Leu Ser Gln Lys Gly Ala Leu Gln Gln Ala Val Arg Asn Pro Ile Tyr Asn Leu Glu Ala Met Ala Ala Glu Val Lys Glu Arg Thr Lys
lacI

cgcagaaacgtggctggcctggttcaccacgcgggaaacggtctgataagagacaccggcatactctgcgacatcgtataacgttactgg 6210
gcgtctttgcaccgaccggaccaagtggtgcgccctttgccagactattctctgtggccgtatgagacgctgtagcatattgcaatgacc

30 25 20 15 10 5
Ala Ser Val His Ser Ala Gln Asn Val Val Arg Ser Val Thr Gln Tyr Ser Val Gly Ala Tyr Glu Ala Val Asp Tyr Leu Thr Val Pro
lacI

tttcacattcaccaccctgaattgactctcttccgggcgctatcatgccataccgcgaaaggttttgcgccattcgatggtgtcc 3′
••• 6295
aaagtgtaagtggtgggacttaactgagagaaggcccgcgatagtacggtatggcgctttccaaaacgcggtaagctaccacagg 5′

1
Lys Met
lacI lacI promoter

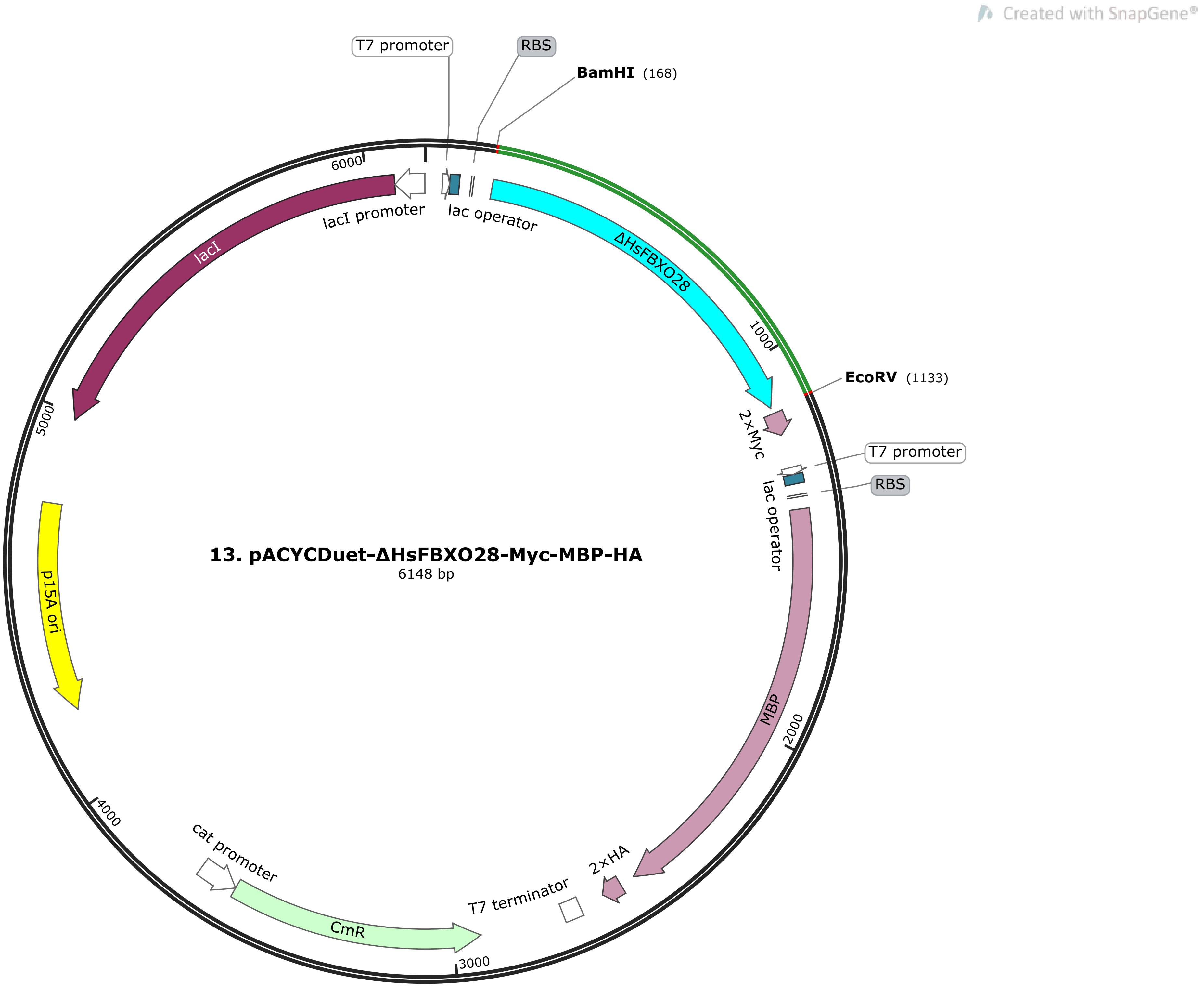

Created with SnapGene®
T7 promoter
RBS
BamHI (168)
6000
lacI promoter
lac operator
ΔHsFBXO28
1000
EcoRV (1133)
lacI
2×Myc
T7 promoter
RBS
lac operator
5000
p15A ori
13. pACYCDuet-ΔHsFBXO28-Myc-MBP-HA
6148 bp
MBP
2000
4000
cat promoter
2×HA
T7 terminator
CmR
3000

Sequence: 13. pACYCDuet-ΔHsFBXO28-Myc-MBP-HA.dna (Circular / 6148 bp)
Enzymes: < Unsaved Enzyme Set > (2 of 678 total)
Features: 18 total

Unique Cutters **Bold**

5′ gggatctcgacgctctcccttatgcgactcctgcattaggaaattaatacgactcactataggggaattgtgagcggataacaattcccc 90
3′ ccctagagctgcgagagggaatacgctgaggacgtaatcctttaattatgctgagtgatatccccttaacactcgcctattgttaaggggg

T7 promoter | lac operator

BamHI

tgtagaaataattttgtttaactttaataaggagatataccatgggcagcagcGcatcaccatcatcaccaagccaggGatccatggcgg 180
acatctttattaaaacaaattgaaattattcctctatatggtacccgtcgtcgCgtagtggtagtagtggttcggtccCtaggtaccgcc

1
Met Ala

RBS | ΔHsFBXO28

cagcggcggaggagcggatggcagaggaaggaggcggcggccaaggcgacggcggttcctctttggcctccggctctacccagcgacagc 270
gtcgccgcctcctcgcctaccgtctccttcctccgccgccggttccgctgccgccaaggagaaaccggaggccgagatgggtcgctgtcg

5 10 15 20 25 30
Ala Ala Ala Glu Glu Arg Met Ala Glu Glu Gly Gly Gly Gly Gln Gly Asp Gly Gly Ser Ser Leu Ala Ser Gly Ser Thr Gln Arg Gln

ΔHsFBXO28

ctccaccgcccgcgccacagcacccgcagccggggtcccaggcgctcccagcccccgcgctggctccggaccagctgcctcaagagaggt 360
gaggtggcgggcgcggtgtcgtgggcgtcggccccagggtccgcgagggtcgggggcgcgaccgaggcctggtcgacggagttctctcca

35 40 45 50 55 60
Pro Pro Pro Pro Ala Pro Gln His Pro Gln Pro Gly Ser Gln Ala Leu Pro Ala Pro Ala Leu Ala Pro Asp Gln Leu Pro Gln Glu Arg

ΔHsFBXO28

accataatctatgtcagaaacaagttaaagcacaactcccaaggagagagtcagaaaggagaaaccattcattagctcgtcatgcagaca 450
tggtattagatacagtctttgttcaatttcgtgttgagggttcctctctcagtctttcctctttggtaagtaatcgagcagtacgtctgt

65 70 75 80 85 90
Tyr His Asn Leu Cys Gln Lys Gln Val Lys Ala Gln Leu Pro Arg Arg Glu Ser Glu Arg Arg Asn His Ser Leu Ala Arg His Ala Asp

ΔHsFBXO28

ttcttgctgctgttgaaacaaggctgtcactattaaatatgactttcatgaaatatgtggattccaatctctgttgcttcatcccaggaa 540
aagaacgacgacaactttgttccgacagtgataatttatactgaaagtactttatacacctaaggttagagacaacgaagtagggtcctt

95 100 105 110 115 120
Ile Leu Ala Ala Val Glu Thr Arg Leu Ser Leu Leu Asn Met Thr Phe Met Lys Tyr Val Asp Ser Asn Leu Cys Cys Phe Ile Pro Gly

ΔHsFBXO28

aggtgattgatgagatttatcgtgtgttgagatatgtcaattctaccagagcccctcaacgagctcatgaagtacttcaagaattaaggg 630
tccactaactactctaaatagcacacaactctatacagttaagatggtctcggggagttgctcgagtacttcatgaagttcttaattccc

125 130 135 140 145 150
Lys Val Ile Asp Glu Ile Tyr Arg Val Leu Arg Tyr Val Asn Ser Thr Arg Ala Pro Gln Arg Ala His Glu Val Leu Gln Glu Leu Arg

ΔHsFBXO28

atatatcctctatggcaatggagtactttgatgaaaagattgttccaattttaaagaggaaattaccaggatcagatgtttctggaagac 720
tatataggagataccgttacctcatgaaactacttttctaacaaggttaaaatttctcctttaatggtcctagtctacaaagaccttctg

155 160 165 170 175 180
Asp Ile Ser Ser Met Ala Met Glu Tyr Phe Asp Glu Lys Ile Val Pro Ile Leu Lys Arg Lys Leu Pro Gly Ser Asp Val Ser Gly Arg

ΔHsFBXO28

tcatgggctctcctccagttccaggaccgtctgcagccctaacaacaatgcagctcttctccaagcaaaatccttcaagacaagaggtta 810
agtacccgagaggaggtcaaggtcctggcagacgtcgggattgttgttacgtcgagaagaggttcgttttaggaagttctgttctccaat

185 190 195 200 205 210
Leu Met Gly Ser Pro Pro Val Pro Gly Pro Ser Ala Ala Leu Thr Thr Met Gln Leu Phe Ser Lys Gln Asn Pro Ser Arg Gln Glu Val

ΔHsFBXO28

ccaaactccagcagcaggttaaaacaaatggtgctggcgtgactgttctcaggcgtgaaatttctgagcttcgcaccaaagtgcaagaac 900
ggtttgaggtcgtcgtccaattttgtttaccacgaccgcactgacaagagtccgcactttaaagactcgaagcgtggtttcacgttcttg

215 220 225 230 235 240
Thr Lys Leu Gln Gln Gln Val Lys Thr Asn Gly Ala Gly Val Thr Val Leu Arg Arg Glu Ile Ser Glu Leu Arg Thr Lys Val Gln Glu

ΔHsFBXO28

```
agcaaaaacagcttcaagaccaggaccagaaactgctagagcagacccagatcataggtgaacaaaatgcacggttggcagagctagaac   990
tcgtttttgtcgaagttctggtcctggtctttgacgatctcgtctgggtctagtatccacttgttttacgtgccaaccgtctcgatcttg
        245             250             255             260             265             270
Gln Gln Lys Gln Leu Gln Asp Gln Asp Gln Lys Leu Leu Glu Gln Thr Gln Ile Ile Gly Glu Gln Asn Ala Arg Leu Ala Glu Leu Glu
ΔHsFBXO28

gcaaactacgagaagtaatggaaagtgctgtaggaaattcctcagggtccgggcagaatgaggagtctcctcggaaacgaaaaaaggcca   1080
cgtttgatgctcttcattacctttcacgacatcctttaaggagtcccaggcccgtcttactcctcagaggagcctttgctttttttccggt
        275             280             285             290             295             300
Arg Lys Leu Arg Glu Val Met Glu Ser Ala Val Gly Asn Ser Ser Gly Ser Gly Gln Asn Glu Glu Ser Pro Arg Lys Arg Lys Lys Ala
ΔHsFBXO28

                                                  EcoRV
cggaagccatagactctcttaggaaatctaaacgtcttcggaatagaaagGATATCACTAGTCCTGAGCAGAAGCTCATCTCCGAGGAGG   1170
gccttcggtatctgagagaatcctttagatttgcagaagccttatctttcCTATAGTGATCAGGACTCGTCTTCGAGTAGAGGCTCCTCC
        305             310             315                                     1               5
Thr Glu Ala Ile Asp Ser Leu Arg Lys Ser Lys Arg Leu Arg Asn Arg Lys Asp Ile Thr Ser Pro Glu Gln Lys Leu Ile Ser Glu Glu
ΔHsFBXO28                                                                         2×Myc

ACCTCGAGCAGAAGCTCATCTCCGAGGAGGACCTCCTGActgcaggtcgacaagcttgcggccgcataatgcttaagtcgaacagaaagt   1260
TGGAGCTCGTCTTCGAGTAGAGGCTCCTCCTGGAGGACTgacgtccagctgttcgaacgccggcgtattacgaattcagcttgtctttca
    10              15              20
Asp Leu Glu Gln Lys Leu Ile Ser Glu Glu Asp Leu Leu Thr Ala Gly Arg Gln Ala Cys Gly Arg Ile Met Leu Lys Ser Asn Arg Lys
2×Myc                                   (in frame with 2×Myc)

aatcgtattgtacacggccgcataatcgaaattaatacgactcactataggggaattgtgagcggataacaattccccatcttagtatat   1350
ttagcataacatgtgccggcgtattagctttaattatgctgagtgatatccccttaacactcgcctattgttaaggggtagaatcatata
*
                                        T7 promoter         lac operator

tagttaagtataagaaggagatatacatatggcagatctcaattgggaattcatgaaaatcgaagaaggtaaactggtaatctggattaa   1440
atcaattcatattcttcctctatatgtataccgtctagagttaacccttaagtactttagcttcttccatttgaccattagacctaatt
                                                            1               5               10
                                                            Met Lys Ile Glu Glu Gly Lys Leu Val Ile Trp Ile Asn
                RBS                                         MBP

cggcgataaaggctataacggtctcgctgaagtcggtaagaaattcgagaaagataccggaattaaagtcaccgttgagcatccggataa   1530
gccgctatttccgatattgccagagcgacttcagccattctttaagctctttctatggccttaatttcagtggcaactcgtaggcctatt
        15              20              25              30              35              40
Gly Asp Lys Gly Tyr Asn Gly Leu Ala Glu Val Gly Lys Lys Phe Glu Lys Asp Thr Gly Ile Lys Val Thr Val Glu His Pro Asp Lys
MBP

actggaagagaaattcccacaggttgcggcaactggcgatggccctgacattatcttctgggcacacgaccgctttggtggctacgctca   1620
tgaccttctctttaagggtgtccaacgccgttgaccgctaccgggactgtaatagaagacccgtgtgctggcgaaaccaccgatgcgagt
        45              50              55              60              65              70
Leu Glu Glu Lys Phe Pro Gln Val Ala Ala Thr Gly Asp Gly Pro Asp Ile Ile Phe Trp Ala His Asp Arg Phe Gly Gly Tyr Ala Gln
MBP

atctggcctgttggctgaaatcaccccggacaaagcgttccaggacaagctgtatccgtttacctgggatgccgtacgttacaacggcaa   1710
tagaccggacaaccgactttagtggggcctgtttcgcaaggtcctgttcgacataggcaaatggaccctacggcatgcaatgttgccgtt
        75              80              85              90              95              100
Ser Gly Leu Leu Ala Glu Ile Thr Pro Asp Lys Ala Phe Gln Asp Lys Leu Tyr Pro Phe Thr Trp Asp Ala Val Arg Tyr Asn Gly Lys
MBP

gctgattgcttacccgatcgctgttgaagcgttatcgctgatttataacaaagatctgctgccgaacccgccaaaaacctgggaagagat   1800
cgactaacgaatgggctagcgacaacttcgcaatagcgactaaatattgtttctagacgacggcttgggcggtttttggacccttctcta
        105             110             115             120             125             130
Leu Ile Ala Tyr Pro Ile Ala Val Glu Ala Leu Ser Leu Ile Tyr Asn Lys Asp Leu Leu Pro Asn Pro Pro Lys Thr Trp Glu Glu Ile
MBP
```

```
cccggcgctggataaagaactgaaagcgaaaggtaagagcgcgctgatgttcaacctgcaagaaccgtacttcacctggccgctgattgc   1890
gggccgcgacctatttcttgactttcgctttccattctcgcgcgactacaagttggacgttcttggcatgaagtggaccggcgactaacg
     135       140       145       150       155       160
Pro Ala Leu Asp Lys Glu Leu Lys Ala Lys Gly Lys Ser Ala Leu Met Phe Asn Leu Gln Glu Pro Tyr Phe Thr Trp Pro Leu Ile Ala
MBP

tgctgacggggggttatgcgttcaagtatgaaaacggcaagtacgacattaaagacgtgggcgtggataacgctggcgcgaaagcgggtct   1980
acgactgccccccaatacgcaagttcatacttttgccgttcatgctgtaatttctgcacccgcacctattgcgaccgcgctttcgcccaga
     165       170       175       180       185       190
Ala Asp Gly Gly Tyr Ala Phe Lys Tyr Glu Asn Gly Lys Tyr Asp Ile Lys Asp Val Gly Val Asp Asn Ala Gly Ala Lys Ala Gly Leu
MBP

gaccttcctggttgacctgattaaaaacaaacacatgaatgcagacaccgattactccatcgcagaagctgcctttaataaaggcgaaac   2070
ctggaaggaccaactggactaatttttgtttgtgtacttacgtctgtggctaatgaggtagcgtcttcgacggaaattatttccgctttg
     195       200       205       210       215       220
Thr Phe Leu Val Asp Leu Ile Lys Asn Lys His Met Asn Ala Asp Thr Asp Tyr Ser Ile Ala Glu Ala Ala Phe Asn Lys Gly Glu Thr
MBP

agcgatgaccatcaacggcccgtgggcatggtccaacatcgacaccagcaaagtgaattatggtgtaacggtactgccgaccttcaaggg   2160
tcgctactggtagttgccgggcacccgtaccaggttgtagctgtggtcgtttcacttaataccacattgccatgacggctggaagttccc
     225       230       235       240       245       250
Ala Met Thr Ile Asn Gly Pro Trp Ala Trp Ser Asn Ile Asp Thr Ser Lys Val Asn Tyr Gly Val Thr Val Leu Pro Thr Phe Lys Gly
MBP

tcaaccatccaaaccgttcgttggcgtgctgagcgcaggtattaacgccgccagtccgaacaaagagctggcaaaagagttcctcgaaaa   2250
agttggtaggtttggcaagcaaccgcacgactcgcgtccataattgcggcggtcaggcttgtttctcgaccgttttctcaaggagctttt
     255       260       265       270       275       280
Gln Pro Ser Lys Pro Phe Val Gly Val Leu Ser Ala Gly Ile Asn Ala Ala Ser Pro Asn Lys Glu Leu Ala Lys Glu Phe Leu Glu Asn
MBP

ctatctgctgactgatgaaggtctggaagcggttaataaagacaaaccgctgggtgccgtagcgctgaagtcttacgaggaagagttggc   2340
gatagacgactgactacttccagaccttcgccaattatttctgtttggcgacccacggcatcgcgacttcagaatgctccttctcaaccg
     285       290       295       300       305       310
Tyr Leu Leu Thr Asp Glu Gly Leu Glu Ala Val Asn Lys Asp Lys Pro Leu Gly Ala Val Ala Leu Lys Ser Tyr Glu Glu Glu Leu Ala
MBP

gaaagatccacgtattgccgccactatggaaaacgcccagaaaggtgaaatcatgccgaacatcccgcagatgtccgctttctggtatgc   2430
ctttctaggtgcataacggcggtgataccttttgcgggtctttccactttagtacggcttgtagggcgtctacaggcgaaagaccatacg
     315       320       325       330       335       340
Lys Asp Pro Arg Ile Ala Ala Thr Met Glu Asn Ala Gln Lys Gly Glu Ile Met Pro Asn Ile Pro Gln Met Ser Ala Phe Trp Tyr Ala
MBP

cgtgcgtactgcggtgatcaacgccgccagcggtcgtcagactgtcgatgaagccctgaaagacgcgcagactgaattcgagCCCGGGAG   2520
gcacgcatgacgccactagttgcggcggtcgccagcagtctgacagctacttcgggactttctgcgcgtctgacttaagctcGGGCCCTC
     345       350       355       360       365
Val Arg Thr Ala Val Ile Asn Ala Ala Ser Gly Arg Gln Thr Val Asp Glu Ala Leu Lys Asp Ala Gln Thr Glu Phe Glu Pro Gly Arg
MBP                                                                           (in frame with MBP)

GCCTctcggcgcgcctgcaggtTACCCATACGACGTACCAGATTACGCTTACCCATACGACGTACCAGATTACGCTcgacaagcttgcgg   2610
CGGAgagccgcgcggacgtccaATGGGTATGCTGCATGGTCTAATGCGAATGGGTATGCTGCATGGTCTAATGCGAgctgttcgaacgcc
                         1         5         10        15
Pro Leu Gly Ala Pro Ala Gly Tyr Pro Tyr Asp Val Pro Asp Tyr Ala Tyr Pro Tyr Asp Val Pro Asp Tyr Ala Arg Gln Ala Cys Gly
(in frame with MBP)         2×HA

ccgcataatgcttaagtaattaacctaggctgctgccaccgctgagcaataactagcataaccccttggggcctctaaacgggtcttgag   2700
ggcgtattacgaattcattaattggatccgacgacggtggcgactcgttattgatcgtattggggaaccccggagatttgcccagaactc
Arg Ile Met Leu Lys *
(in frame with 2×HA)                                  T7 terminator

gggttttttgctgaaacctcaggcatttgagaagcacacggtcacactgcttccggtagtcaataaaccggtaaaccagcaatagacata   2790
cccaaaaaacgactttggagtccgtaaactcttcgtgtgccagtgtgacgaaggccatcagttatttggccatttggtcgttatctgtat
T7 terminator
```

agcggctatttaacgaccctgccctgaaccgacgaccgggtcgaatttgctttcgaatttctgccattcatccgcttattatcacttatt 2880
tcgccgataaattgctgggacgggacttggctgctggcccagcttaaacgaaagcttaaagacggtaagtaggcgaataatagtgaataa

caggcgtagcaccaggcgtttaagggcaccaataactgccttaaaaaaattacgccccgccctgccactcatcgcagtactgttgtaatt 2970
gtccgcatcgtggtccgcaaattcccgtggttattgacggaatttttttaatgcggggcgggacggtgagtagcgtcatgacaacattaa
220 * Ala Gly Gly Gln 215 Trp Glu Asp Cys Tyr 210 Gln Gln Leu Glu
CmR

cattaagcattctgccgacatggaagccatcacagacggcatgatgaacctgaatcgccagcggcatcagcaccttgtcgccttgcgtat 3060
gtaattcgtaagacggctgtaccttcggtagtgtctgccgtactacttggacttagcggtcgccgtagtcgtggaacagcggaacgcata
Asn 205 Leu Met Arg Gly Val 200 His Phe Gly Asp Cys 195 Val Ala His His Val 190 Gln Ile Ala Leu Pro 185 Met Leu Val Lys Asp 180 Gly Gln Thr Tyr
CmR

aatatttgcccatagtgaaaacgggggcgaagaagttgtccatattggccacgtttaaatcaaaactggtgaaactcacccagggattgg 3150
ttataaacgggtatcacttttgcccccgcttcttcaacaggtataaccggtgcaaatttagttttgaccactttgagtgggtccctaacc
Tyr 175 Lys Gly Met Thr Phe 170 Val Pro Ala Phe Phe 165 Asn Asp Met Asn Ala 160 Val Asn Leu Asp Phe 155 Ser Thr Phe Ser Val 150 Trp Pro Asn Ala
CmR

ctgagacgaaaaacatattctcaataaaccctttagggaaataggccaggttttcaccgtaacacgccacatcttgcgaatatatgtgta 3240
gactctgctttttgtataagagttatttgggaaatccctttatccggtccaaaagtggcattgtgcggtgtagaacgcttatatacacat
Ser 145 Val Phe Phe Met Asn 140 Glu Ile Phe Gly Lys 135 Pro Phe Tyr Ala Leu 130 Asn Glu Gly Tyr Cys 125 Ala Val Asp Gln Ser 120 Tyr Ile His Leu
CmR

gaaactgccggaaatcgtcgtggtattcactccagagcgatgaaaacgtttcagtttgctcatggaaaacggtgtaacaagggtgaacac 3330
ctttgacggcctttagcagcaccataagtgaggtctcgctacttttgcaaagtcaaacgagtaccttttgccacattgttcccacttgtg
Phe 115 Gln Arg Phe Asp Asp 110 His Tyr Glu Ser Trp 105 Leu Ser Ser Phe Thr 100 Glu Thr Gln Glu His 95 Phe Val Thr Tyr Cys 90 Pro His Val Ser
CmR

tatcccatatcaccagctcaccgtctttcattgccatacggaactccggatgagcattcatcaggcgggcaagaatgtgaataaaggccg 3420
atagggtatagtggtcgagtggcagaaagtaacggtatgccttgaggcctactcgtaagtagtccgcccgttcttacacttatttccggc
Asp 85 Trp Ile Val Leu Glu 80 Gly Asp Lys Met Ala 75 Met Arg Phe Glu Pro 70 His Ala Asn Met Leu 65 Arg Ala Leu Ile His 60 Ile Phe Ala Pro
CmR

gataaaacttgtgcttatttttctttacggtctttaaaaaggccgtaatatccagctgaacggtctggttataggtacattgagcaactg 3510
ctattttgaacacgaataaaaagaaatgccagaaatttttccggcattataggtcgacttgccagaccaatatccatgtaactcgttgac
Tyr 55 Phe Lys His Lys Asn 50 Lys Lys Val Thr Lys 45 Leu Phe Ala Thr Ile 40 Asp Leu Gln Val Thr 35 Gln Asn Tyr Thr Cys 30 Gln Ala Val Ser
CmR

actgaaatgcctcaaaatgttctttacgatgccattgggatatatcaacggtggtatatccagtgatttttttctccattttagcttcct 3600
tgactttacggagttttacaagaaatgctacggtaaccctatatagttgccaccatataggtcactaaaaaaagaggtaaaatcgaagga
Gln 25 Phe Ala Glu Phe His 20 Glu Lys Arg His Trp 15 Gln Ser Ile Asp Val 10 Thr Thr Tyr Gly Thr 5 Ile Lys Lys Glu 1 Met
CmR
cat promoter

tagctcctgaaaatctcgataactcaaaaaatacgcccggtagtgatcttatttcattatggtgaaagttggaacctcttacgtgccgat 3690
atcgaggacttttagagctattgagttttttatgcgggccatcactagaataaagtaataccactttcaaccttggagaatgcacggcta
cat promoter

caacgtctcattttcgccaaaagttggcccagggcttcccggtatcaacagggacaccaggatttatttattctgcgaagtgatcttccg 3780
gttgcagagtaaaagcggttttcaaccgggtcccgaagggccatagttgtccctgtggtcctaaataaataagacgcttcactagaaggc
cat promoter

tcacaggtatttattcggcgcaaagtgcgtcgggtgatgctgccaacttactgatttagtgtatgatggtgtttttgaggtgctccagtg 3870
agtgtccataaataagccgcgtttcacgcagcccactacgacggttgaatgactaaatcacatactaccacaaaaactccacgaggtcac

```
gcttctgtttctatcagctgtccctcctgttcagctactgacggggtggtgcgtaacggcaaaagcaccgccggacatcagcgctagcgg
                                                                                          3960
cgaagacaaagatagtcgacagggaggacaagtcgatgactgccccaccacgcattgccgttttcgtggcggcctgtagtcgcgatcgcc

agtgtatactggcttactatgttggcactgatgagggtgtcagtgaagtgcttcatgtggcaggagaaaaaaggctgcaccggtgcgtca
                                                                                          4050
tcacatatgaccgaatgatacaaccgtgactactcccacagtcacttcacgaagtacaccgtcctcttttttccgacgtggccacgcagt

gcagaatatgtgatacaggatatattccgcttcctcgctcactgactcgctacgctcggtcgttcgactgcggcgagcggaaatggctta
                                                                                          4140
cgtcttatacactatgtcctatataaggcgaaggagcgagtgactgagcgatgcgagccagcaagctgacgccgctcgcctttaccgaat

cgaacggggcggagatttcctggaagatgccaggaagatacttaacagggaagtgagagggccgcggcaaagccgtttttccataggctc
                                                                                          4230
gcttgccccgcctctaaaggaccttctacggtccttctatgaattgtcccttcactctcccggcgccgtttcggcaaaaaggtatccgag
                                                                                  p15A ori

cgcccccctgacaagcatcacgaaatctgacgctcaaatcagtggtggcgaaacccgacaggactataaagataccaggcgtttcccctg
                                                                                          4320
gcggggggactgttcgtagtgctttagactgcgagtttagtcaccaccgctttgggctgtcctgatatttctatggtccgcaaaggggac
                                        p15A ori

gcggctccctcgtgcgctctcctgttcctgcctttcggtttaccggtgtcattccgctgttatggccgcgtttgtctcattccacgcctg
                                                                                          4410
cgccgagggagcacgcgagaggacaaggacggaaagccaaatggccacagtaaggcgacaataccggcgcaaacagagtaaggtgcggac
                                        p15A ori

acactcagttccgggtaggcagttcgctccaagctggactgtatgcacgaaccccccgttcagtccgaccgctgcgccttatccggtaac
                                                                                          4500
tgtgagtcaaggcccatccgtcaagcgaggttcgacctgacatacgtgcttggggggcaagtcaggctggcgacgcggaataggccattg
                                        p15A ori

tatcgtcttgagtccaacccggaaagacatgcaaaagcaccactggcagcagccactggtaattgatttagaggagttagtcttgaagtc
                                                                                          4590
atagcagaactcaggttgggcctttctgtacgttttcgtggtgaccgtcgtcggtgaccattaactaaatctcctcaatcagaacttcag
                                        p15A ori

atgcgccggttaaggctaaactgaaaggacaagttttggtgactgcgctcctccaagccagttacctcggttcaaagagttggtagctca
                                                                                          4680
tacgcggccaattccgatttgactttcctgttcaaaaccactgacgcgaggaggttcggtcaatggagccaagtttctcaaccatcgagt
                                        p15A ori

gagaaccttcgaaaaaccgccctgcaaggcggttttttcgttttcagagcaagagattacgcgcagaccaaaacgatctcaagaagatca
                                                                                          4770
ctcttggaagctttttggcgggacgttccgccaaaaaagcaaaagtctcgttctctaatgcgcgtctggttttgctagagttcttctagt
                                    p15A ori

tcttattaatcagataaaatatttctagatttcagtgcaatttatctcttcaaatgtagcacctgaagtcagccccatacgatataagtt
                                                                                          4860
agaataattagtctattttataaagatctaaagtcacgttaaatagagaagtttacatcgtggacttcagtcggggtatgctatattcaa

gtaattctcatgttagtcatgccccgcgcccaccggaaggagctgactgggttgaaggctctcaagggcatcggtcgagatcccggtgcc
                                                                                          4950
cattaagagtacaatcagtacggggcgcgggtggccttcctcgactgacccaacttccgagagttcccgtagccagctctagggccacgg

taatgagtgagctaacttacattaattgcgttgcgctcactgcccgctttccagtcgggaaacctgtcgtgccagctgcattaatgaatc
                                                                                          5040
attactcactcgattgaatgtaattaacgcaacgcgagtgacgggcgaaaggtcagccctttggacagcacggtcgacgtaattacttag
                     360             355             350             345
                  *  Gln Gly Ser Glu Leu Arg Ser Val Gln Arg Ala Leu Gln Met Leu Ser Asp
                                       lacI

ggccaacgcgcggggagaggcggtttgcgtattgggcgccagggtggtttttcttttcaccagtgagacgggcaacagctgattgccctt
                                                                                          5130
ccggttgcgcgcccctctccgccaaacgcataacccgcggtcccaccaaaaagaaaagtggtcactctgcccgttgtcgactaacgggaa
         340             335             330             325             320             315
Ala Leu Ala Arg Pro Ser Ala Thr Gln Thr Asn Pro Ala Leu Thr Thr Lys Arg Lys Val Leu Ser Val Pro Leu Leu Gln Asn Gly Lys
                                        lacI
```

caccgcctggccctgagagagttgcagcaagcggtccacgctggtttgccccagcaggcgaaaatcctgtttgatggtggttaacggcgg 5220
gtggcggaccgggactctctcaacgtcgttcgccaggtgcgaccaaacggggtcgtccgcttttaggacaaactaccaccaattgccgcc

310 305 300 295 290 285
Val Ala Gln Gly Gln Ser Leu Gln Leu Leu Arg Asp Val Ser Thr Gln Gly Leu Leu Arg Phe Asp Gln Lys Ile Thr Thr Leu Pro Pro
lacI

gatataacatgagctgtcttcggtatcgtcgtatcccactaccgagatgtccgcaccaacgcgcagcccggactcggtaatggcgcgcat 5310
ctatattgtactcgacagaagccatagcagcatagggtgatggctctacaggcgtggttgcgcgtcgggcctgagccattaccgcgcgta

280 275 270 265 260 255
Ile Tyr Cys Ser Ser Asp Glu Thr Asp Asp Tyr Gly Val Val Ser Ile Asp Ala Gly Val Arg Leu Gly Ser Glu Thr Ile Ala Arg Met
lacI

tgcgcccagcgccatctgatcgttggcaaccagcatcgcagtgggaacgatgccctcattcagcatttgcatggtttgttgaaaaccgga 5400
acgcgggtcgcggtagactagcaaccgttggtcgtagcgtcacccttgctacgggagtaagtcgtaaacgtaccaaacaacttttggcct

250 245 240 235 230 225
Ala Gly Leu Ala Met Gln Asp Asn Ala Val Leu Met Ala Thr Pro Val Ile Gly Glu Asn Leu Met Gln Met Thr Gln Gln Phe Gly Ser
lacI

catggcactccagtcgccttcccgttccgctatcggctgaatttgattgcgagtgagatatttatgccagccagccagacgcagacgcgc 5490
gtaccgtgaggtcagcggaagggcaaggcgatagccgacttaaactaacgctcactctataaatacggtcggtcggtctgcgtctgcgcg

220 215 210 205 200 195
Met Ala Ser Trp Asp Gly Glu Arg Glu Ala Ile Pro Gln Ile Gln Asn Arg Thr Leu Tyr Lys His Trp Gly Ala Leu Arg Leu Arg Ala
lacI

cgagacagaacttaatgggcccgctaacagcgcgatttgctggtgacccaatgcgaccagatgctccacgcccagtcgcgtaccgtcttc 5580
gctctgtcttgaattacccgggcgattgtcgcgctaaacgaccactgggttacgctggtctacgaggtgcgggtcagcgcatggcagaag

190 185 180 175 170 165
Ser Val Ser Ser Leu Pro Gly Ala Leu Leu Ala Ile Gln Gln His Gly Leu Ala Val Leu His Glu Val Gly Leu Arg Thr Gly Asp Glu
lacI

atgggagaaaataatactgttgatgggtgtctggtcagagacatcaagaaataacgccggaacattagtgcaggcagcttccacagcaat 5670
taccctcttttattatgacaactacccacagaccagtctctgtagttctttattgcggccttgtaatcacgtccgtcgaaggtgtcgtta

160 155 150 145 140 135
His Ser Phe Ile Ile Ser Asn Ile Pro Thr Gln Asp Ser Val Asp Leu Phe Leu Ala Pro Val Asn Thr Cys Ala Ala Glu Val Ala Ile
lacI

ggcatcctggtcatccagcggatagttaatgatcagcccactgacgcgttgcgcgagaagattgtgcaccgccgctttacaggcttcgac 5760
ccgtaggaccagtaggtcgcctatcaattactagtcgggtgactgcgcaacgcgctcttctaacacgtggcggcgaaatgtccgaagctg

130 125 120 115 110 105
Ala Asp Gln Asp Asp Leu Pro Tyr Asn Ile Ile Leu Gly Ser Val Arg Gln Ala Leu Leu Asn His Val Ala Ala Lys Cys Ala Glu Val
lacI

gccgcttcgttctaccatcgacaccaccacgctggcacccagttgatcggcgcgagatttaatcgccgcgacaatttgcgacggcgcgtg 5850
cggcgaagcaagatggtagctgtggtggtgcgaccgtgggtcaactagccgcgctctaaattagcggcgctgttaaacgctgccgcgcac

100 95 90 85 80 75
Gly Ser Arg Glu Val Met Ser Val Val Val Ser Ala Gly Leu Gln Asp Ala Arg Ser Lys Ile Ala Ala Val Ile Gln Ser Pro Ala His
lacI

cagggccagactggaggtggcaacgccaatcagcaacgactgtttgcccgccagttgttgtgccacgcggttgggaatgtaattcagctc 5940
gtcccggtctgacctccaccgttgcggttagtcgttgctgacaaacgggcggtcaacaacacggtgcgccaacccttacattaagtcgag

70 65 60 55 50 45
Leu Ala Leu Ser Ser Thr Ala Val Gly Ile Leu Leu Ser Gln Lys Gly Ala Leu Gln Gln Ala Val Arg Asn Pro Ile Tyr Asn Leu Glu
lacI

cgccatcgccgcttccactttttcccgcgttttcgcagaaacgtggctggcctggttcaccacgcgggaaacggtctgataagagacacc 6030
gcggtagcggcgaaggtgaaaaagggcgcaaaagcgtctttgcaccgaccggaccaagtggtgcgccctttgccagactattctctgtgg

40 35 30 25 20 15
Ala Met Ala Ala Glu Val Lys Glu Arg Thr Lys Ala Ser Val His Ser Ala Gln Asn Val Val Arg Ser Val Thr Gln Tyr Ser Val Gly
lacI

ggcatactctgcgacatcgtataacgttactggtttcacattcaccaccctgaattgactctcttccgggcgctatcatgccataccgcg 6120
ccgtatgagacgctgtagcatattgcaatgaccaaagtgtaagtggtgggacttaactgagagaaggcccgcgatagtacggtatggcgc

10 5 1
Ala Tyr Glu Ala Val Asp Tyr Leu Thr Val Pro Lys Met
lacI
lacI promoter

```
aaaggttttgcgccattcgatggtgtcc     3′
                                 6148
tttccaaaacgcggtaagctaccacagg     5′
```

lacI promoter
