# Supplementary material for: A Bacterial Platform for Studying Ubiquitination Cascades Anchored by SCF-Type E3 Ubiquitin Ligases
Source: Biomolecules. 2024 Sep 25;14(10):1209. doi: 10.3390/biom14101209 (PMC11505812; doi:10.3390/biom14101209)
Supplement: Supplementary file 1 [file biomolecules-14-01209-s001.zip › Figure S1-S4.pdf]

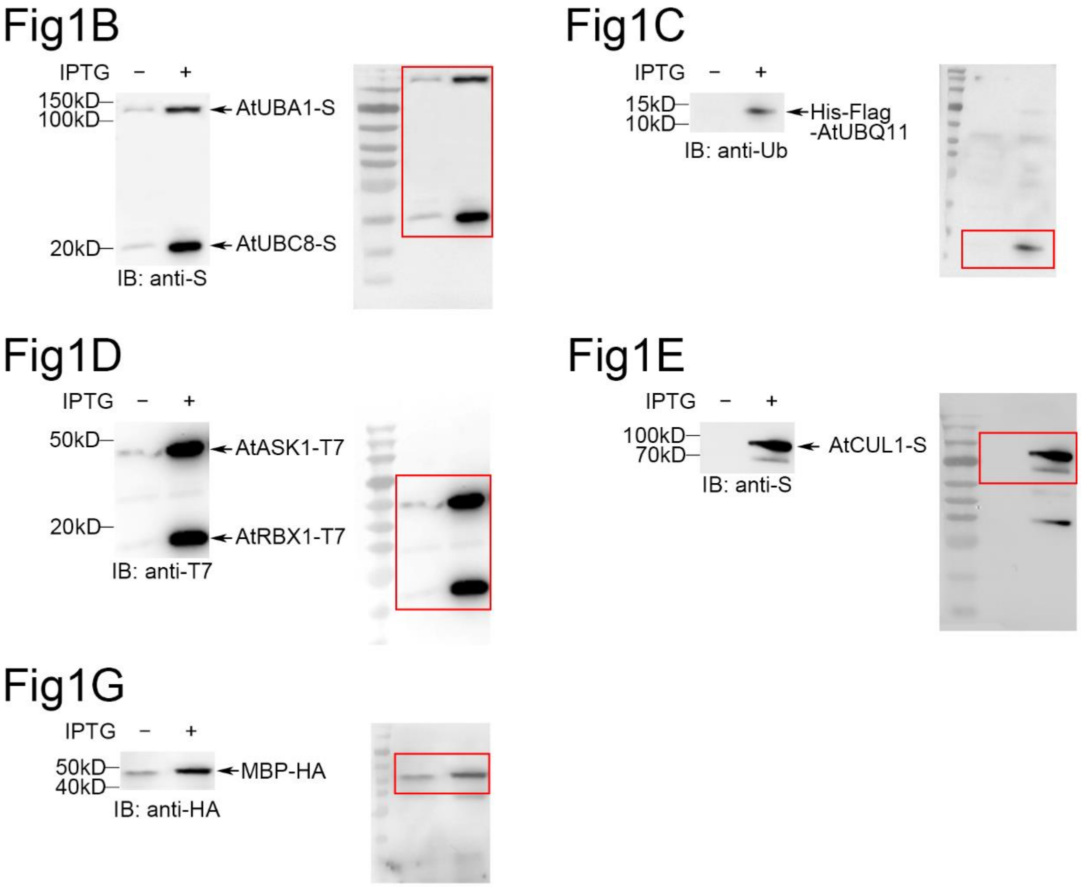

Figure S1. Uncropped Western-blots images corresponding to Figure 1B-E and G.

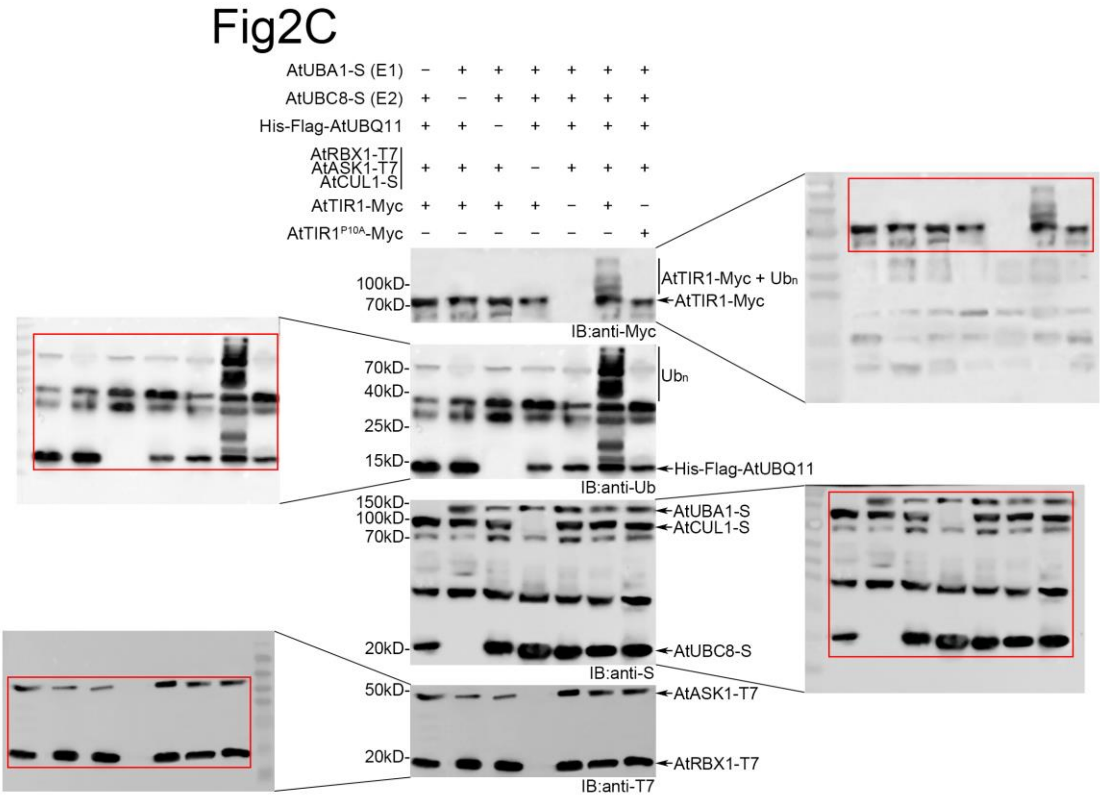

Figure S2. Uncropped Western-blots images corresponding to Figure 2C.

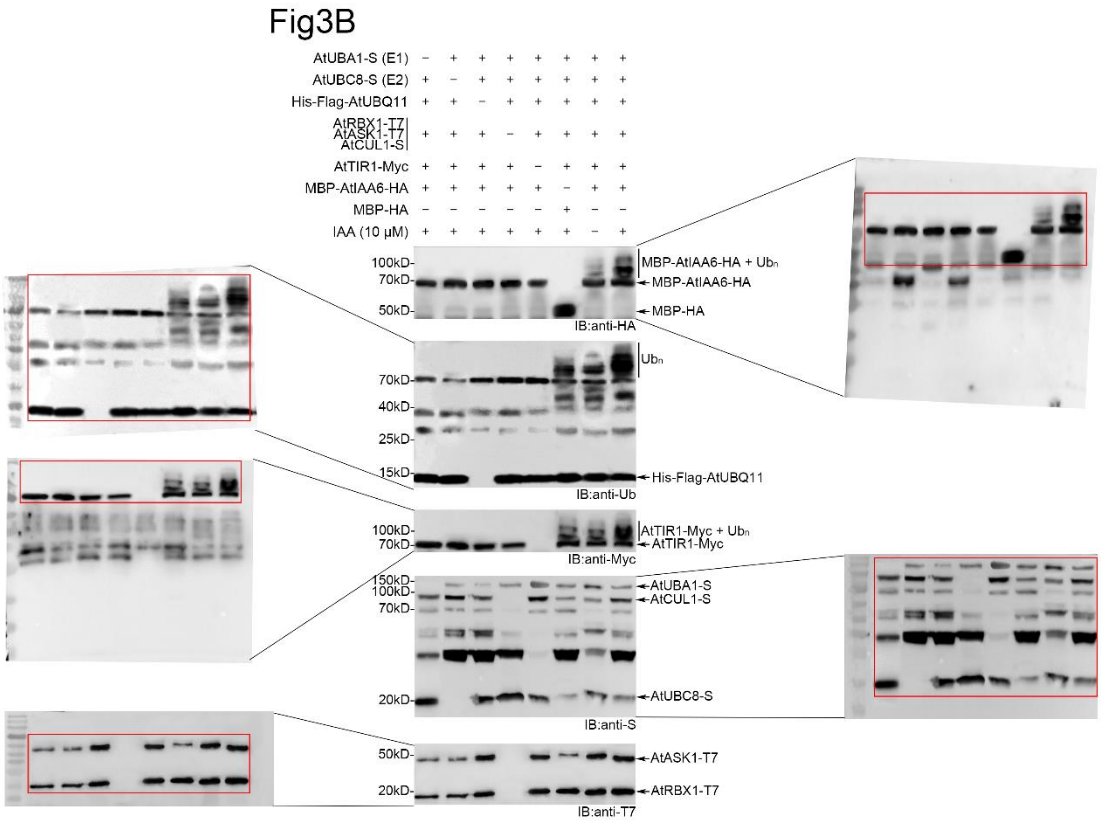

Figure S3. Uncropped Western-blots images corresponding to Figure 3B.

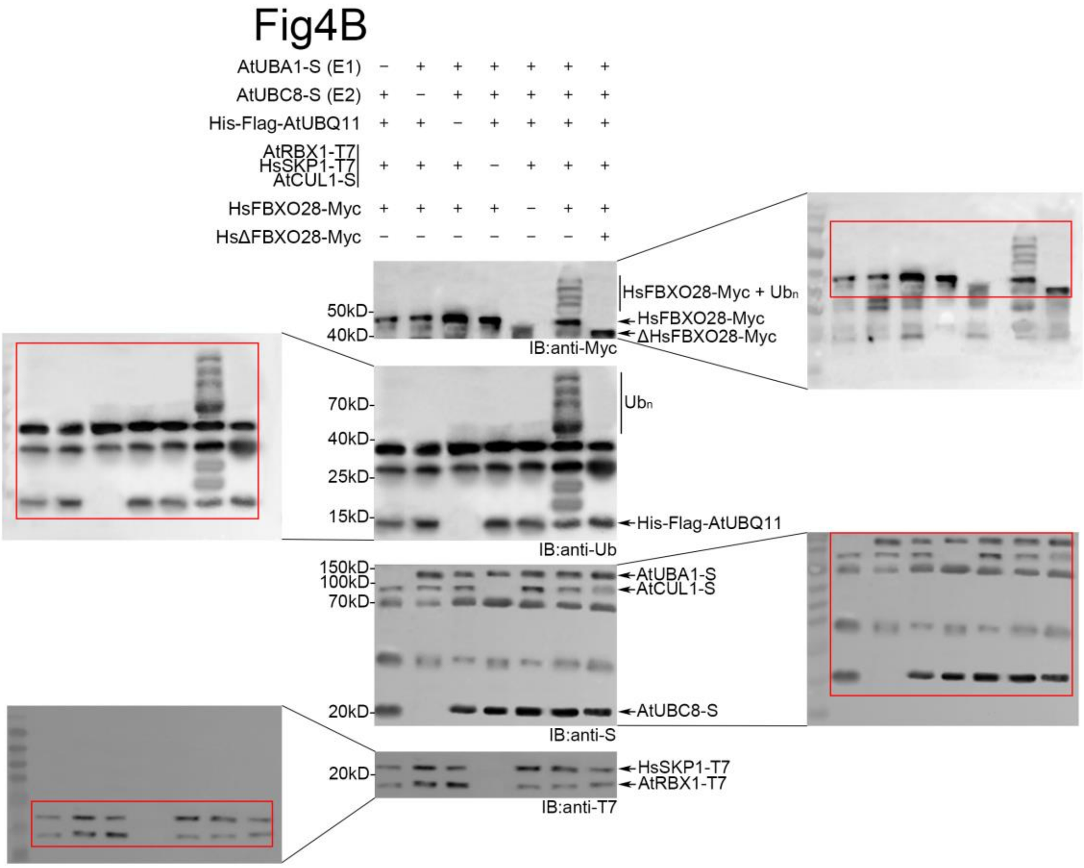

Figure S4. Uncropped Western-blots images corresponding to Figure 4B.
