# Supplementary material for: A Bacterial Platform for Studying Ubiquitination Cascades Anchored by SCF-Type E3 Ubiquitin Ligases
Source: Biomolecules. 2024 Sep 25;14(10):1209. doi: 10.3390/biom14101209 (PMC11505812; doi:10.3390/biom14101209)
Supplement: Supplementary file 1 [file biomolecules-14-01209-s001.zip › Table S1.pdf]

Table S1. Primers used in this study.

Name	5'-3' sequence
UBA1 F	TCTCAATTGGATATCGATGCTTCACAAGCGAGCTAGTGAA
UBA1 R	AGACTCGAGGGTACCCCTGAAGTAGATAGAGACGAGAGGA
UBC8-S F	CCAGGGATCCGAATTCATGGCTTCGAAACGGATCTTGAA
UBC8-S R	CGCCGAGCTCGAATTCTTAAGCTGCGCTAGTAGACGAGT
CUL1 F	CGCTGACGTCGGTACCATGGAGCGCAAGACTATTGACT
CUL1 R	CAGACTCGAGGGTACCAGCCAAGTACCTAAACATGTTAG
HIS-FLAG-UBQ F	AGGAGATATACCATGGGCAGCAGC
HIS-FLAG-UBQ R	CAGACTCGAGGGTACCTTAACCACCACGGAGCCTGAGGACCA
TIR1 F	CATCACCAAGCCAGGGATCCATGCAGAAGCGAATAGCCTTGTCG
TIR1 R	TATCGAGCTCGGATCCTAATCCGTTAGTAGTAATGATTTGCCTGG
IAA6 F	GAATTCGAGCCCGGGAGGCCTATGGCAAAGGAAGGTCTAG
IAA6 R	CAGGCGCGCCGAGAGGCCTATCTTGCTGGAGACCAAAACC
